# Investigation of Direct and Retro Chromone-2-Carboxamides Based Analogs of *Pseudomonas aeruginosa* Quorum Sensing Signal as New Anti-Biofilm Agents

**DOI:** 10.3390/ph15040417

**Published:** 2022-03-29

**Authors:** Jeanne Trognon, Gonzalo Vera, Maya Rima, Jean-Luc Stigliani, Laurent Amielet, Salomé El Hage, Barbora Lajoie, Christine Roques, Fatima El Garah

**Affiliations:** 1 Laboratoire de Génie Chimique, Université de Toulouse, CNRS, INPT, UPS, 31062 Toulouse, France; jeanne.trognon@univ-tlse3.fr (J.T.); gonzalo.vera-namuncura@univ-tlse3.fr (G.V.); maya.rima@univ-tlse3.fr (M.R.); laurent.amielet@univ-tlse3.fr (L.A.); salome.elhage@univ-tlse3.fr (S.E.H.); barbora.lajoie@univ-tlse3.fr (B.L.); christine.roques@univ-tlse3.fr (C.R.); 2 Laboratoire de Chimie de Coordination, Université de Toulouse, CNRS, INPT, UPS, 31062 Toulouse, France; jean-luc.stigliani@lcc-toulouse.fr

**Keywords:** *Pseudomonas aeruginosa*, Quorum Sensing inhibition, chromone carboxamides, PqsR, *Pseudomonas* Quinolone Signal (PQS), biofilms, molecular docking

## Abstract

Biofilm formation is considered a major cause of therapeutic failure because bacteria in biofilms have higher protection against antimicrobials. Thus, biofilm-related infections are extremely challenging to treat and pose major concerns for public health, along with huge economic impacts. *Pseudomonas aeruginosa*, in particular, is a “critical priority” pathogen, responsible for severe infections, especially in cystic fibrosis patients because of its capacity to form resistant biofilms. Therefore, new therapeutic approaches are needed to complete the pipeline of molecules offering new targets and modes of action. Biofilm formation is mainly controlled by Quorum Sensing (QS), a communication system based on signaling molecules. In the present study, we employed a molecular docking approach (Autodock Vina) to assess two series of chromones-based compounds as possible ligands for PqsR, a LuxR-type receptor. Most compounds showed good predicted affinities for PqsR, higher than the PQS native ligand. Encouraged by these docking results, we synthesized a library of 34 direct and 25 retro chromone carboxamides using two optimized routes from 2-chromone carboxylic acid as starting material for both series. We evaluated the synthesized carboxamides for their ability to inhibit the biofilm formation of *P. aeruginosa* in vitro. Overall, results showed several chromone 2-carboxamides of the retro series are potent inhibitors of the formation of *P. aeruginosa* biofilms (16/25 compound with % inhibition ≥ 50% at 50 μM), without cytotoxicity on Vero cells (IC_50_ > 1.0 mM). The 2,4-dinitro-N-(4-oxo-4H-chromen-2-yl) benzamide (**6n**) was the most promising antibiofilm compound, with potential for hit to lead optimization.

## 1. Introduction

Many ESKAPE pathogens, including the Gram-negative *Pseudomonas aeruginosa*, grow in biofilm as a survival strategy to environmental stresses. Biofilms are aggregated bacteria attached to surfaces and embedded in a self-produced matrix of extracellular polymeric substances (EPS) composed of polysaccharides, DNA and proteins [1]. Bacteria in biofilms are more protected against antimicrobial treatments, and are up to 1500 times more resistant to antibiotics and biocides than planktonic bacteria [2]. The local environment within a biofilm also offers protection to persister cells from the immune system and increases the likelihood of genetic mutations [3]. Furthermore, bacterial biofilms can form on any surface (medical implants, mucosa, water distribution systems…), thus becoming a reservoir for cross-contaminations and infections, especially in hospitals [4]. Bacteria, especially in the biofilm state, can be extremely difficult to eradicate. This alarming situation led the WHO to urge for an efficient strategy against bacterial biofilm-associated infections [5]. Among ESKAPE biofilm producers, *P. aeruginosa* is one of the most critical opportunistic pathogens, responsible for severe and often fatal infections, especially in immune-depressed and cystic fibrosis (CF) patients. 

Biofilm initiation, formation, and maintenance are mainly regulated by Quorum Sensing (QS), a communication system where bacteria produce, detect, and collectively respond to small signal molecules called auto-inducers (AI). As a result of their key role in biofilm formation and bacterial virulence, QS mechanisms are a promising target for new anti-infective therapies; several reports have validated the capacity of anti-QS molecules to reduce the expression of virulence factors of *P. aeruginosa* and increase the susceptibility of bacterial biofilms to antibiotics, both in vitro and in vivo [6,7,8,9,10]. 

*P. aeruginosa* QS systems (*las*, *rhl*, *iqs*, and *pqs*) are well known, with some auto-inducers like acyl-homoserine lactones (AHLs) and alkylquinolones (AQs), being shared with other gram-negative species [11]. In particular, the *P. aeruginosa pqs* system is based on 2-alkyl-4-quinolones as signal molecules, namely, 2-heptyl-3-hydroxy-4-quinolone (PQS for *Pseudomonas* Quinolone Signal), and its immediate precursor 2-heptyl-4-hydroxyquinoline (HHQ) [12]. PQS binds to, and activates, the transcriptional regulator PqsR, also known as MvfR. The PqsR/PQS complex triggers the transcription of the pqsABCDE operon, coding for the enzymes required for the synthesis of HHQ (PqsA to PqsE), which is, in turn, oxidized to PQS by the PqsH monooxygenase. HHQ and PQS act by generating an autoinductive feedback loop that accelerates their own synthesis. Thus, when PQS activates PqsR, its concentration rises exponentially. PqsR-deficient strains showed reduced pathogenicity in several in vivo infection models, proving its central role during the infection process [13]. The *pqs* system also regulates the formation of biofilms and the production of EPS matrix by external DNA release [14]. Therefore, PqsR is a potential drug target to attenuate *P. aeruginosa* virulence and inhibit biofilm formation, without affecting bacterial viability to reduce the risk of emerging resistances. 

**Figure 1:**
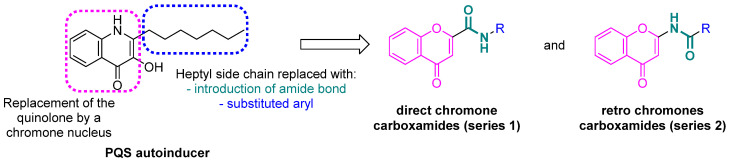
Rational design of chromone carboxamides as PQS analogs.

In the present study, we proposed the synthesis of two new series of structural analogs of the native PQS auto-inducer as potential *P. aeruginosa* anti-biofilm agents. The rational design was mainly based on the replacement of the quinolone scaffold of the native PQS molecule by its bioisostere, the chromone [(4H)-1-benzopyran-4-one] nucleus, while the heptyl side chain was replaced by an amide function linked to a substituted aromatic moiety (Figure 1). 

**Figure 2:**
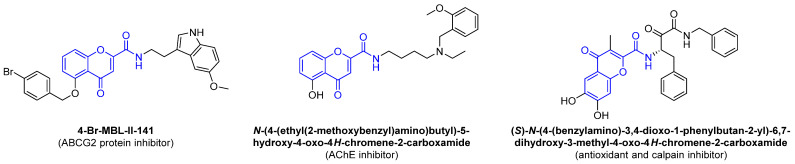
Structure of some bioactive chromone carboxamide based compounds [16,17,18].

The chromone scaffold constitutes the basic nucleus of flavones and is well known as a pharmacophore of a large amount of natural and synthetic bioactive molecules [15]. Within this wide family, it has been found that chromone carboxamide derivatives can present interesting bio-applications, such as ABCG2 protein inhibition [16], AChE inhibition [17], calpain inhibition, and antioxidant properties [18] (Figure 2). In addition to the excellent biological profile shown by the chromone nucleus, it is worth highlighting its well described functionalization, that allows interesting chemical diversity and the obtaining of derivatives on a large scale and in a cost-effective fashion [19,20].

In an effort to gain knowledge on the structure–activity relationship (SAR) of chromone carboxamides as potential anti-PqsR agents, we designed a series of direct chromone-CO-NH-R amides and retro chromone-NH-CO-R amides (series 1 and 2, Figure 1), bearing various substituents in terms of electronic effects, size, and physicochemical properties. The strategy of exchanging an amide for a retro-amide has been successfully used in the field of antimicrobial research with the identification of retro-amides with increased activity against *C. albicans* [21] and *M. tuberculosis* [22]. However, despite the large amount of literature and data on chromone carboxamides, their retro-amide counterparts have not been extensively explored [19]. 

We employed docking calculations to identify the best ligands for the PqsR receptor.

Based on the docking results, we worked on synthetic routes and applied two different strategies, both using 2-chromone carboxylic acid as starting material, to obtain the desired compounds in good yields and purity (Table 1). Thus, two series of chromone-based carboxamides, bearing electron-donating and electron-withdrawing groups (Table 2 and Table 3). were synthetized and fully characterized. Among the retro carboxamide series, 24 compounds are newly described in the present study. The synthetized compounds were then evaluated in their potency to inhibit *P. aeruginosa* biofilms using an in vitro cellular assay developed to promote the growth of adherent cells [23]. The structure–activity relationships of the two series are discussed, in light of predicted binding interactions with the PqsR active site. We also evaluated the cytotoxic activity on Vero cells of the most interesting biofilm inhibitors.

## 2. Results and Discussion 

### 2.1. Docking of Chromone 2-Carboxamides with PqsR 

**Figure 3:**
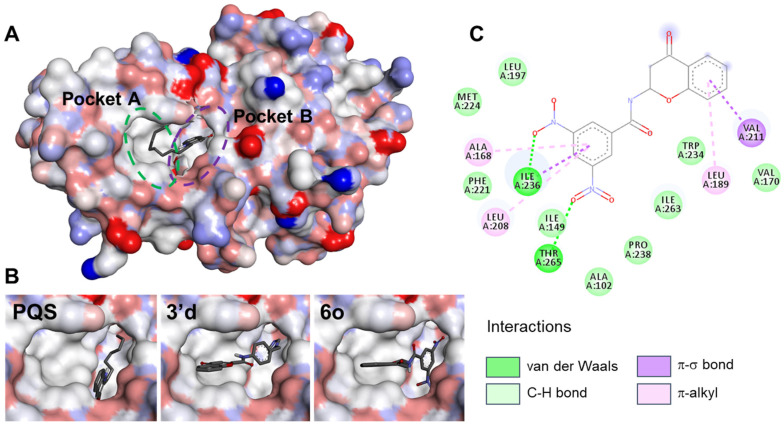
(**A**) PqsR-CBD structure (PDB code 4JVC) binding with NHQ [25]. PqsR solvent surface colored by atom charge (blue = positive charge, red = negative charge). (**B**) PqsR ligand binding site with PQS, compounds **3′d** and **6o**. (**C**) 2D interactions diagram of compound **3′d**.

We used the AutoDock Vina software to predict the affinity of target compounds for the PqsR protein [24]. PqsR, also named MvfR (Multiple Virulence Factor Regulator), is a global activator controlling the expression of many virulence factors in *P. aeruginosa* [13,25,26]. A monomeric unit of PqsR is composed of two subdomains connected by a β-sheet region β4 [25]. The ligand-binding domain is a hydrophobic cavity inserted between helix α4 and sheets β4-5-7. The active site is composed of the two pockets A and B (Figure 3A), mostly formed by aliphatic amino acid residues [25,27]. The superficial A pocket is located between sheets β5-7 while the deep B pocket is buried in the hinge region of PqsR, between the two subdomains. Ilangovan et al. identified Ile149, Phe221, Tyr258, Ile263, and to a lesser extent, Ile186, Leu207, and Ile236 residues as essential amino acids in the interaction between PqsR and the 2-nonyl-4-hydroxy-quinoline (NHQ) ligand, a PQS homolog with a C9 side chain [25].

Docking simulations were carried out with the apo form of the PqsR-CBD (PDB code: 4JVC [25]) by keeping all protein residues rigid, except for Ile149, Leu189, Ile236, Tyr258, and Thr265 [25,28]. The docking search space was larger than the A and B pockets of the PqsR-CBD. We considered the binding free energy (E_bind_, kcal·mol^−1^, Table 1) and inhibition constant (Ki, µM, Table S1) of the best ranked conformations as the main parameters (score) for the analysis of AutoDock results. Compounds with the highest affinity are those with the lowest binding energy. We also considered the protonated forms at pH = 7.2 for amine-containing structures. Docking results are reported in Table 2 and Table 3. Docking of PQS gave a predicted binding energy (E_bind_) of −8.1 kcal·mol^−1^ for the best pose, in which PQS is deeply bonded in the B pocket (Figure 3B), through hydrophobic interactions with the residues Ala102, Ile149, Ala168, and Pro238, contrary to NHQ which occupies the two pockets (Figure 3A). As expected, PQS interactions were mainly hydrophobic [25,29,30]. Our docking results showed PQS forms several H-bonds between its carbonyl group and Gln194 and Ser196, and between its 3-hydroxyl group and Leu197. 

For the design of chromone 2-carboxamides, the major modification done to the PQS structure was the replacement of the 3-hydroxyquinolone by a non-substituted chromone to conserve the steric requirements for optimal binding [25]. Preliminary docking calculations of AQs ligands (PQS and NHQ), bearing a chromone nucleus in place of the quinolone part, gave interesting scores, with predicted binding energies very close to their quinolone counterparts (data not shown). This allowed us to speculate that the single replacement of the quinolone by its chromone bioisostere would give good PqsR agonists rather than antagonists. Thus, we considered other structural modifications and the PQS heptyl side chain was replaced by an amide linkage, bearing either a phenyl group, substituted by electron-donating or electron-withdrawing groups (EDG and EWG), or an aliphatic side chain. We studied 25 pairs of direct chromone-CO-NH-R amides (series 1, compounds **3a**–**y**) and retro chromone-NH-CO-R amides (series 2, compounds **6a**–**y**). To widen the panel of structures, we also considered direct chromone carboxamides bearing various oxygen- and nitrogen-containing aromatic and aliphatic side chains (compounds **3′a–i**). 

Docking results showed most of the compounds fit by occupying the binding site pockets. In a similar manner to NHQ, the chromone nucleus resides inside the B pocket and the alkyl or substituted phenyl side chain occupies the A pocket. In accordance with the litterature, interactions were mainly hydrophobic, based on π-σ and π-alkyl interactions, and the most frequently involved amino acids were Ile149, Ile236, Phe221, and Tyr258 [25]. However, some compounds, such as **3′d** and **6o**, revealed flipped poses with the chromone part being in the A pocket (Figure 3B,C). Overall, all compounds showed good predicted affinities for the PqsR binding domain, with several compounds exhibiting lower binding energies than the PQS ligand itself (E_bind_ ≤ −8.1 kcal·mol^−1^).

The first compounds to be considered were aromatic amides with the N-phenyl ring substituted by various donor and acceptor groups. Unsubstituted phenyl **3a** showed a predicted affinity very similar to that of PQS (E_bind_ = −8.0 kcal·mol^−1^), while its retro counterpart **6a** turned out to be a better ligand (E_bind_ = −9.1 kcal·mol^−1^). Introduction of an alkyl group on the para (4′) position of the phenyl ring, with increasing length up to C3, had a slightly beneficial impact on the predicted affinity, with binding energies ranging from −8.0 kcal·mol^−1^ for **3a** to −8.7 kcal·mol^−1^ for **3d**. Interestingly, the position of the side chain on the phenyl ring also had an impact, as observed with the ethyl group; since comparison of compounds **3c** and **3′a** indicated the meta position (3′) is slightly more favorable than the para position for the ethyl group, with E_bind_ = −8.6 and −9.2 kcal·mol^−1^, respectively. 

Introduction of a halogen atom at ortho (2′) and para (4′) positions (**3e**–**j**) does not change the predicted affinity compared to the 4′-CH_3_ compound (**3b**) and all docking poses are perfectly superimposable. Except for the fluorinated compounds, changing the position of the halogen from 4′ to 2′ slightly improves the affinity for the retro amide series (**6**e–**j**), while the introduction of two halogens (compounds **3k**/**6k** and **3l**/**6l**) does not significantly impact the docking results. 

Concerning the aliphatic sub-series, compounds bearing a propyl side chain (**3w** and **6w**) showed the lowest predicted affinity for the PqsR receptor (E_bind_ = −7.7 and −7.8 kcal·mol^−1^, respectively), certainly because of their inability to reach far enough inside the A pocket. Introduction of a cyclohexyl ring (**3x** and **6x**) did improve the predicted binding affinity to match the scores observed for the phenyl series, but the best docking score of the aliphatic sub-series was obtained with the introduction of an adamantyl group (**3y** and **6y**) with E_bind_ = −9.5 and −9.4 kcal·mol^−1^, respectively. This bulky cycle showed the formation of several beneficial interactions with the Val170, Val211, Ile236, Tyr258, and Ile 263 residues. 

Compounds of the sub-series of direct carboxamides **3′a–i** showed calculated binding energies ranging from −7.5 to −9.5 kcal/mol, with affinity mostly based on hydrophobic interactions. Suprisingly, no H-bonds were observed with this series, despite the presence of H-donor and acceptor groups in their structure (Figure 3 and Table 1). The most interesting compound of this series, in terms of docking score, was compound **3′d** bearing an (imidazol-1-yl) methylphenyl moety (E_bind_ = −9.5 kcal·mol^−1^). 

Overall, it emerges from this docking study that a high predicted affinity is reached when the ligand’s structure is long enough to occupy both pockets of the binding site, which translated in numerous hydrophobic interactions, especially with aliphatic residues. Comparison of the two series showed that retro-amides **6a**–**y** presented similar docking scores to their direct amide counterparts **3a**–**y**, i.e., with a difference in binding energies ΔE_bind_ ≤ 1.1 kcal·mol^−1^ lower than the standard error defined for Autodock Vina [31], suggesting the orientation of the amide bond (-CO-NH- vs. -NH-CO-) does not play a major role in their binding within the PqsR receptor. Regarding their drug-likeness properties, all the chromone derivatives possess suitable logP values and no violations of the Lipinski’s rule were found (Table S1). Thus, all compounds were synthesized and evaluated for their ability to inhibit the growth of *P. aeruginosa* biofilms in vitro in view of the identification of new anti-biofilm hit compounds.

### 2.2. Synthesis

#### 2.2.1. Synthesis of Direct Chromone Carboxamides Series 1

**Scheme 1:**
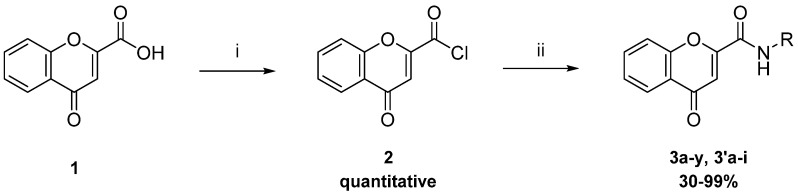
Synthesis of compounds **3a**–**3′i**. Reaction conditions: (i) PCl_5_/dry cyclohexane, 1.5 h; (ii) R-NH_2_/TEA/dry CH_2_Cl_2_, 12 h.

The synthesis of the direct carboxamide derivatives corresponding to Series 1 was carried out using the two-step method developed in our laboratory [32], starting from the commercially available 2-chromone carboxylic acid **1**, which, in a first step, was conveniently transformed in the respective chromone acyl chloride **2** using PCl_5_ in dry cyclohexane. In a second step, the intermediate **2** was condensed with aliphatic and aromatic amines in presence of triethylamine to give amides **3a**–**y** and **3′a–i** (Scheme 1). We observed that under these mild conditions, formation of the carboxamides occurs smoothly, being complete after 12 h of stirring at room temperature. One of the main advantages of this procedure is the use of the acyl chloride **2** as coupling partner, which can be synthetized quantitatively in high scale and also proved to be exceptionally stable at low temperatures, facilitating its storage compared with other activated forms of the acid **1**, which must be generated in situ [33,34]. Another important aspect to consider is the easy work-up used that provides, in most of cases, the analytically pure carboxamides by simple recrystallization in ethanol or extraction in dichloromethane. Thus, these derivatives were obtained in a range of 30–99% global yield, which is, to the best of our knowledge, the highest average reported to date for this family of carboxamides [33,34,35].

#### 2.2.2. Synthesis of Retro Chromone Carboxamides Series 2

Synthesis of the retro carboxamide derivatives corresponding to Series 2 represented a big synthetic challenge, due to the scarcity of references in literature, where only a few examples of this sub-class of compounds are described [36,37,38,39]. A logical disconnection of the amide function allows proposal of obtaining 2-nitrogen chromone derivatives by peptidic coupling between 2-aminochromone and different activated carboxylic acids. For this purpose, 2-aminochromone **4** was synthetized according to the procedure described by Ghosh et al. [40], and the commercially available 4-bromobenzoic acid **5** was used as a model carboxylic acid (Scheme in Table 1). Studies commenced by employing the conditions described by Reis et al. [36]. When the reaction was carried out in one pot using phosphoryl chloride as an activating agent (Table 1, entry 1), only traces of the desired amide **6** were isolated. Improvements were observed when the coupling was performed in two separated steps that involved the conversion of **5** in its respective acid chloride and posterior addition of **4** in basic media (entry 2) [37,38], affording a 32% yield of the carboxamide **6**. Attempts at optimizing this two-step procedure led to the use of CDI [41] and PyBOP [34] as coupling reagents (entries 3 and 4, respectively), However, the yield of the expected product **6** did not exceed 25% for these assays. Inspired by the work of Reddy et al. [42], we facilitated reaction between the acid chloride obtained from **5** with aminochromone **4** using aluminum powder, which can act as a Lewis acid in order to increase the electrophilic nature of the carbonyl function [43]. Notably, under these conditions (entry 5), the reaction afforded a considerable amount of product where **6** was not identified. These unsatisfying results allow us to hypothesize that 2-aminochromone **4** is not convenient to be used as a nucleophile in a standard peptidic coupling procedure, due to the extremely low electron density over its nitrogen atom. This fact made us turn our attention to peptidic coupling methods that involve the use of poorly nucleophilic amines. In this context, the recent protocol described by Otsuka et al. [41] is highlighted; where amidation of neutral amines was performed, producing excellent yield, using dimethylacetamide (DMAC) as both solvent and basic catalyst. However, applying this procedure using **4** only produced a 31% yield of the desired amide **6** (entry 6). Interestingly, when the same reaction was performed using 2 equivalents of TEA (entry 7), the yield of **6** decreased and appearance of the unexpected product **7** was observed, which can be related to a base-induced regioselective radical arylation reaction [44]. 

Alternatively, the Pd-catalized Buchwald-Hartwig reaction between non-nucleophilic amines and activated esters have been successfully described for obtaining of amides [45,46]. Emulating this cross-coupling condition, using the phenyl ester of **5** as intermediate, we were able to obtain chromone carboxamide **6** in a satisfactory 70% yield (entry 8). 

**Scheme 2:**
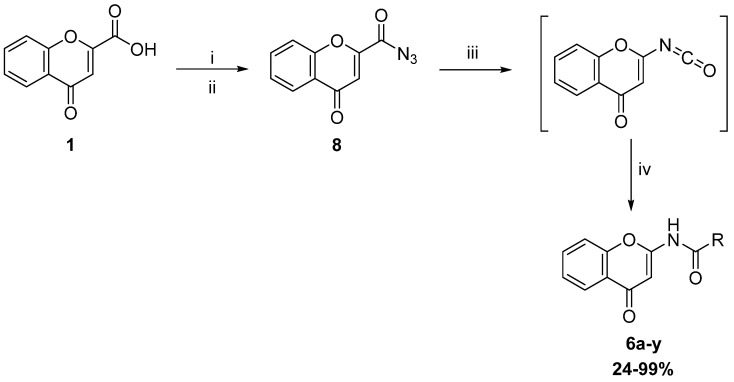
Synthesis of compounds **6a-y**. Reaction conditions: (i) PCl_5_/dry cyclohexane, 1.5 h; (ii) NaN_3_/dry cyclohexane, 1.5 h; (iii) 80 °C, 1.5 to 2 h; (iv) R-COOH/dry cyclohexane/reflux 12 h.

Finally, encouraged by the method described by Payard [39], we decided to attempt the synthesis of amide **6** using 2-chromone carboxylic acid **1** as a precursor, by its transformation in the respective carbonyl azide **8**. This multistep reaction involves conversion of azide **8** into isocyanate intermediate, which can be attacked by unactivated carboxylic acids to generate the desired amides by a Curtius rearrangement (Scheme 2) [39,47]. Using the previously reported conditions, a considerable amount of insoluble side product was found alongside amide **6** in low yield. Further exploration of the reaction parameters led us to notice that the formation of the isocyanate intermediary is indicated by a change of colour of the solution from white to intense orange, while overheating generates an insoluble brown suspension that contains several impurities. To our delight, when the temperature and time were carefully controlled, the retro carboxamide **6** was obtained with an excellent 90% yield (entry 9), which represents the best output in the performed screening. Besides the considerable yield improvement, a second important advantage of this method is the possibility to use the 2-chromone carboxylic acid **1** as a precursor, which is also the starting material in the synthesis of the direct carboxamides of Series 1. It is worth mentioning that when the same reaction was tried out in one pot, i.e., without isolation of the carbonyl azide intermediate **8** [47], only traces of compound **6** were detected (entry 10). With the optimized conditions in hand, 25 derivatives of Series 2 were synthetized in a range of 24–99% global yield.

### 2.3. Anti-Biofilm Activity against P. aeruginosa

Kinetic studies using *pqsR* mutants proved that the *pqs* system is involved in early steps of biofilm formation and supported the fact that it represents an interesting target for new anti-virulence and antibiofilm compounds against *P. aeruginosa* pathogenicity [27,29,30,48,49]. Regarding the crucial role of the *pqs* system in the adaptability of *P. aeruginosa*, including biofilm formation, virulence regulation and some remarkable secondary effects, like iron acquisition or cytotoxicity [26,50,51], we chose an anti-biofilm assay based on detection of adherent cell population, rather than the classically used crystal violet method, suitable for the evaluation of total biomass but nonspecific enough [52]. 

The effect of all compounds was evaluated on PAO1 biofilms, grown in 24-well microplates. The antibiofilm assay used in this study is based on a low nutritive culture medium, a rather weak inoculum (10^2^ CFU/mL), and a regular renewal of the culture medium (at 2, 4, 6, 20, and 24 h), in order to promote the growth of adhered cells as biofilm, instead of the growth and/or sedimentation of planktonic cells [23]. Since the QS systems are involved in early steps of biofilm formation, compounds were added at t0. After incubation, viable cells were quantified by CFU plate counts. This quantification method has been shown to exhibit the best responsiveness to different levels of efficacy and the best reproducibility with respect to responsiveness (Slope/SR = 1.02), making it a highly reliable method for the assessment of treatment efficacy [53]. 

The rather low concentration of 50 µM used was set up according to detected concentrations of QS molecules in CF patients’ lungs [54]. The minimal inhibitory (MIC) and minimal bactericidal concentrations (MBC) of all compounds were determined according to Eucast recommendations to make sure their activity was not due to classical antibacterial mechanisms and to ensure they do not affect bacterial growth at the tested concentration. For all compounds, MICs/MBCs were higher than 500 µM. Results of biofilm (adherent population) inhibition are reported in Table 2 and Table 3. To be considered active, a threshold of 50% inhibition, relative to the untreated control, was applied. 

Among the thirty-four direct chromone carboxamides evaluated (series 1, **3a-3′i**), six compounds showed interesting activity with % inhibition higher than 50%, seven compounds were totally inactive (% inhibition < 10%) and the remaining 21 showed low to moderate activity (Table 2 and Table 3, Figure S1). The best compound of Series 1 was the 4-fluorophenyl substituted compound **3e** (73.3 ± 11.0%). The other halogenated analogs **3f-l** (4-Cl, 4-Br, 2-F, 2-Cl, 2-Br, 2-Cl-4-F, 3-Cl-4-F) showed poor biofilm inhibition, despite good docking scores, close to those of PQS and **3e**. The 3-ethylphenyl compound **3′a** also showed promising anti-biofilm activity (70.5 ± 5.6%) with a value that matched its predicted binding energy (−9.2 kcal·mol^−1^). 

We also observed some surprising results for the series of direct carboxamides. For example, the **3′d** compound, bearing an (imidazol-1-yl-methyl) phenyl moiety, showed high predicted affinity for the PqsR active site in docking results (E_bind_ = −9.5 kcal·mol^−1^), but turned out to be completely inactive as a biofilm inhibitor. The same observations can be made for compounds **3t**, **3y**, and **3′i**, bearing a 3,5-dimethoxyphenyl, an adamatyl, and a 2-oxo-2-(pyrrolidin-1-yl-ethyl) piperazine-1-carbonyl group, respectively. 

**Figure 4:**
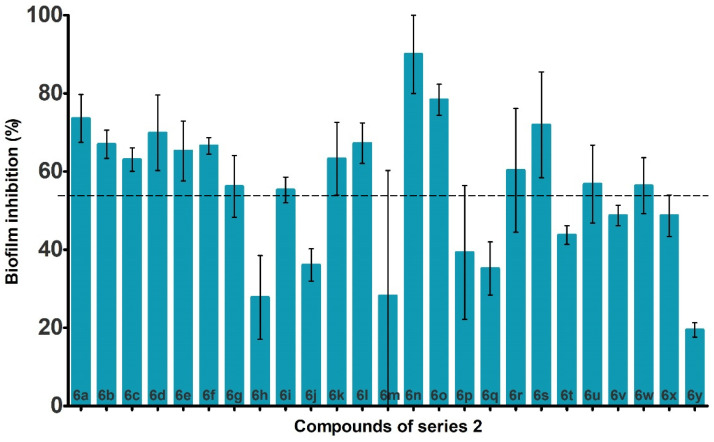
Anti-biofilm activity against *P. aerugiosa* PAO1 of retro chromone carboxamides (**6a-y**) at 50 µM, expressed as inhibition % (GraphPad 5.0).

The most potent antibiofilm compounds were obtained from Series 2, of which 16 of the 25 retro carboxamides evaluated showed inhibition rates greater than 50% (Figure 4). The most remarkable compound was 2,4-dinitro-N-(4-oxo-4H-chromen-2-yl-benzamide (**6n**), with a biofilm inhibition of 90.9 ± 9.9% at 50 µM. The 3,5-dinitrophenyl analogue **6o** also showed good activity (78.3 ± 4.0%), while the mono 4-nitrophenyl substituted compound **6m** was inactive. Nevertheless, these results are consistent with previous reports which demonstrated that the nitro electron-withdrawing group is critical for antagonists to selectively recognize PqsR over other possible targets such as PqsBC [48,49]. Among other potent retro carboxamides, the 3,4-dimethoxyphenyl derivative **6s** also showed promising antibiofilm activity with a % inhibition of 71.9 ± 13.5% at 50 µM.

Although the high level of similarity of the selected structures did not allow us to properly discriminate in prediction of affinity, the overall good docking scores allow us to hypothesize that active compounds, as evaluated in the anti-biofilm assay, are genuine inhibitors of PqsR; although we cannot exclude an antagonistic effect on other enzymes involved in the biosynthesis of PQS or on other targets. The inability for some compounds (**3y/6y**, **3′d** or **3′i**) with good predicted affinity to target the *pqs* system and inhibit *P. aeruginosa* biofilm formation may be explained by lack of internalization, due to permeability issues and/or the presence of efflux pumps on the bacterial cell membrane [28]. Modification, or inactivation, by cellular metabolism may also be responsible for lack of activity on the whole cell assay, as experienced by Lu et al. [50]; whose research showed antagonists selected on an *E. coli* reporter assay became agonists on a *P. aeruginosa* assay, supporting our choice of an anti-biofilm assay. 

### 2.4. Evaluation of the Cytotoxic Effect on Vero Cells

**Figure 5:**
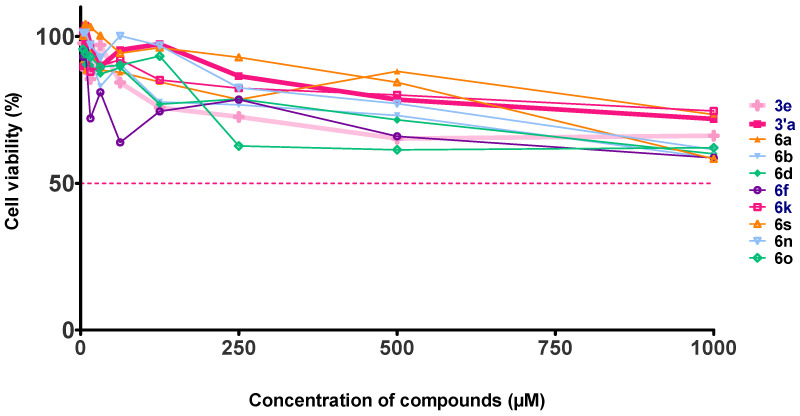
Cytotoxic activity on Vero cells of the 10 most active chromone carboxamides, expressed as viability % (GraphPad 5.0).

Finally, we evaluated the potential cytotoxicity of the 10 most active antibiofilm compounds (i.e., % inhibition ≥ 63%) within the two series of chromone carboxamides against Vero cells. Cells were incubated with different concentrations of compounds **3e**, **3′a**, **6a**, **6b**, **6d**, **6f**, **6k**, **6s**, **6n**, and **6o,** varying from 3.9 µM to 1.0 mM. The relative cell viabilities were determined after 24 h using the MTT assay [55] and compared to the untreated control (100% viability). A 5% Tween solution was used as positive control. Figure 5 reports the viability % for all evaluated concentrations. 

For all evaluated compounds, dose-response curves showed cell viability is greater than 50% at all tested concentrations, including the highest one. Thus, we can conclude all evaluated compounds, including the most active biofilm inhibitor (compound **6n**), exhibit IC_50_ values higher than 1.0 mM. This result is promising for future studies on chromone carboxamides as safe anti-biofilm agents.

## 3. Materials and Methods

### 3.1. Molecular Docking

#### 3.1.1. Protein Structure File and Ligand Preparation 

The X-ray crystal structure of the *P. aeruginosa* PqsR protein was downloaded from the Protein Data Bank website (PDB code: 4JVC, 203 amino acid residues, resolution: 2.5 Å, completeness: 99.2%) and used for structure-based virtual screening [28]. Avogadro software [v1.2.0] was used for the geometric optimization of chemical structures through the UFF force field minimization field in 10, 000 steps with a conjugate gradient algorithm. A conformational analysis was performed in order to find the lowest energy conformation, using the MMFF94 force field of MarvinSketch [v14.10.6.0]. The minimized ligand structures were then used for docking calculations.

#### 3.1.2. Structure-Based Virtual Screening

Discovery Studio Visualizer (DSV) [v17.2.0.16349] software was used for protein visualization and preparation. MolProbity software (Manchester, UK) was used to assign the position of hydrogen atoms and the protonation state of histidine residues. Water molecules were removed before docking calculations.

The docking calculations were performed with the open-source structure-based virtual screening docking program AutoDock Vina v1.1, implemented with the Lamarckian Genetic search algorithm (exhaustiveness of 20). The optimized chemical structures of the ligands and the graphical interface AutoDockTools were used for the preparation of proteins and ligands into their lowest energy 3D conformations [24]. The search space was included in a cubic grid of 24 × 24 × 24 Å, framing both ligand and flexible residues. Flexible torsions of ligands were assigned with AutoTors, an auxiliary module of AutoDockTools. The receptor grid was designed around the following flexible residues: Ile149, Leu189, Ile236, Tyr258, and Thr265 [25]. Ligands were docked in their major microspecies structure at pH = 7.2 (neutral or protonated). For each calculation, ten poses were ranked according to the scoring-function of Autodock Vina. For each ligand, the first pose, i.e., with the lowest energy, was selected [31,56].

### 3.2. Chemistry

#### 3.2.1. General

Melting points were determined on Köfler melting point apparatus (Wagner & Munz GmbH, München, Germany) or Buchi melting point apparatus, model B-545. ^1^H NMR and ^13^C NMR spectra were obtained in CDCl_3_ or DMSO-d_6_ on a AV300 spectrometer (Brüker Biosciences, Billerica, MA, USA), and peak positions are given as s (singlet), d (doublet), t (triplet), q (quadruplet) or m (multiplet). Chemical shift (δ) values were given in ppm, and coupling constants (J) were recorded in Hz. The spectra were analyzed using MestReNova 14.0.1 software. High resolution mass spectrometry (HRMS) was performed by DCI/CH_4_ using a Waters GCT-Premier mass spectrometer in positive mode, signals were given as m/z. Elemental analyses were carried out at the Laboratoire de Chimie de Coordination (Toulouse) with a Perkin Elmer 2400 series II analyzer. Reactions were monitored by thin-layer chromatography (TLC) using pre-coated silica gel plates 60 F-254 (Fluka). Visualization was performed with ultraviolet light (254 nm). Purifications were done by recrystallization in EtOH or DFM/H_2_O or by Flash chromatography using a CombiFlash NextGen 300 (Teledyne Isco) with RediSep silica prepacked cartridges (12 g, 35–70 μm).

All yields were calculated for analytically pure materials. All solvents and commercially available reagents were purchased from Merck-Sigma (St. Quentin Fallavier, France), ThermoFisher Scientific, (Illkirch, France) or VWR-Avantor (Rosny-sous-Bois, France), and were used without further purification. All chemical structures were confirmed by ^1^H NMR (300 MHz), ^13^C NMR (75 MHz) for all compounds, and by HRMS for the newly reported ones. NMR spectra are given as Supplementary Materials. The physico-chemical properties of the previously described compounds are in agreement with the literature data (e.g., **3g**, **3′b** and **3′h** [57]; and **2**, **3a**, **3f**, **3g**, **3y**, **3′a**, **3′c**, **3′g** and **3′i** [32])). 

#### 3.2.2. 4-Oxo-4H-chromene-2-carbonyl chloride (**2**)

4-oxo-4H-chromene-2-carbonyl chloride **2** was prepared according to a previously described method [32,58]. In a round bottom flask were placed 4-oxo-4H-chromene-2-carboxylic acid (10.0 g, 52 mmol), phosphorus pentachloride (12.0 g, 57 mmol) and dry cyclohexane (200 mL). The system was isolated from the exterior by a solvent blast guard filled with dry cyclohexane and then refluxed for 2 h. The hot resultant solution was filtered off and the filtrate was allowed to reach room temperature. The solid obtained was then filtered off, washed with cold cyclohexane and dried under vacuum to afford 10.84 g of the expected acid chloride as white needles, which was used without further purification. Yield: quantitative. Mp 106 °C. ^1^H NMR (300 MHz, DMSO-d_6_): δ 8.06 (dd, J = 7.9, 1.7 Hz, 1H), 7.88 (ddd, J = 8.7, 7.1, 1.7 Hz, 1H), 7.74 (dd, J = 8.6, 1.1 Hz, 1H), 7.55 (ddd, J = 8.0, 7.1, 1.1 Hz, 1H), 6.92 (s, 1H). ^13^C NMR (75 MHz, DMSO-d_6_) δ 178.1, 161.8, 155.9, 153.7, 135.7, 126.6, 125.4, 124.2, 119.4, 114.0.

#### 3.2.3. General Procedure for the Synthesis of Direct Chromone Carboxamides (Series 1, **3a-y** and **3′a-g**)

In a round bottom flask were placed the suitable amine, triethylamine (1.2 equiv.) and anhydrous dichlorometane (10.0 mL). The system was kept at 0 °C using an ice bath, then 4-oxo-4H-chromene-2-carbonyl chloride **2** (1 equiv.) was added in small portions over a 30 min period and the solution was allowed to stir for 12 h at room temperature. After this time, the solid was filtered off, washed with 5 mL of HCl 5%. and dried in an oven at 90 °C. The crude product was recrystallized in EtOH to give the final compound.

4-Oxo-N-phenyl-4H-chromene-2-carboxamide (**3a**) 

Starting from aniline (0.22 mL, 2.4 mmol) and intermediate **2** (0.5 g, 2.4 mmol) and following the general procedure, compound **3a** was obtained as white sparkle solid. Yield: 96%. Mp 229 °C. ^1^H NMR (300 MHz, DMSO-d_6_) δ 10.76 (s, 1H), 8.10 (ddd, J = 8.0, 1.7, 0.5 Hz, 1H), 7.95 (m, 1H), 7.87–7.79 (m, 3H), 7.61–7.55 (m, 1H), 7.49–7.40 (m, 2H), 7.26–7.18 (m, 1H), 7.00 (s, 1H). ^13^C NMR (75 MHz, DMSO-d_6_) δ 177.8, 158.2, 156.2, 155.7, 138.0, 135.6, 129.3 (2C), 126.6, 125.4 (2C), 124.2, 121.6 (2C), 119.5, 111.6. *m/z* calcd. for C_16_H_11_NO_3_ [M + H]^+^ 266.0812. Found: 266.0811.

4-Oxo-N-(p-tolyl)-4H-chromene-2-carboxamide (**3b**)

White solid. Yield: 80%. Mp 229 °C. ^1^H NMR (300 MHz, DMSO-d_6_) δ 10.68 (s, 1H), 8.10 (dd, J = 7.9, 1.6 Hz, 1H), 7.94 (ddd, J = 8.6, 7.0, 1.7 Hz, 1H), 7.86 (dd, J = 8.5, 1.2 Hz, 1H), 7.74–7.66 (m, 2H), 7.58 (ddd, J = 8.1, 7.0, 1.3 Hz, 1H), 7.28–7.17 (m, 2H), 6.98 (s, 1H), 2.32 (s, 3H). ^13^C NMR (75 MHz, DMSO-d_6_) δ 177.8, 158.0, 156.3, 155.7, 135.5 (2C), 134.6, 129.7 (2C), 126.6, 125.4, 124.2, 121.5 (2C), 119.5, 111.5, 21.0. *m/z* calcd. for C_17_H_13_NO_3_ [M + H]^+^ 280.0968. Found: 280.0966.

N-(4-Ethylphenyl)-4-oxo-4H-chromene-2-carboxamide (**3c**)

White solid. Yield: 88%. Mp 179 °C. ^1^H NMR (300 MHz, DMSO-d_6_) δ 10.70 (s, 1H), 8.19–8.05 (m, 1H), 7.98–7.91 (m, 1H), 7.90–7.83 (m, 1H), 7.77–7.68 (m, 2H), 7.58 (ddd, J = 8.1, 6.9, 1.3 Hz, 1H), 7.31–7.22 (m, 2H), 6.99 (s, 1H), 2.62 (q, J = 7.6 Hz, 2H), 1.21 (t, J = 7.6 Hz, 3H). ^13^C NMR (75 MHz, DMSO-d_6_) δ 177.8, 158.0, 156.3, 155.6, 141.0, 135.7, 135.5, 128.5 (2C), 126.6, 125.4, 124.2, 121.6 (2C), 119.5, 111.5, 28.2, 16.1. *m/z* calcd. for C_18_H_15_NO_3_ [M + H]^+^ 294.1125. Found: 294.1125.

4-Oxo-N-(4-propylphenyl)-4H-chromene-2-carboxamide (**3d**)

Light yellow solid. Yield: 99%. Mp 173 °C. ^1^H NMR (300 MHz, DMSO-d_6_) δ 10.70 (s, 1H), 8.10 (dd, J = 8.0, 1.6 Hz, 1H), 7.94 (ddd, J = 8.6, 7.0, 1.7 Hz, 1H), 7.86 (m, 1H), 7.75–7.69 (m, 2H), 7.58 (ddd, J = 8.1, 6.9, 1.3 Hz, 1H), 7.28–7.21 (m, 2H), 6.98 (s, 1H), 2.57 (t, J = 7.5 Hz, 2H), 1.68–1.54 (m, 2H), 0.91 (t, J = 7.3 Hz, 3H). ^13^C NMR (75 MHz, DMSO-d_6_) δ 177.8, 158.0, 156.3, 155.6, 139.3, 135.7, 135.5, 129.1 (2C), 126.6, 125.4, 124.2, 121.5 (2C), 119.5, 111.5, 37.2, 24.5, 14.0. *m/z* calcd. for C_19_H_17_NO_3_ [M + H]^+^ 308.1281. Found: 308.1279.

N-(4-Fluorophenyl)-4-oxo-4H-chromene-2-carboxamide (**3e**)

White solid. Yield: 66%. Mp 211 °C. ^1^H NMR (300 MHz, DMSO-d_6_) δ 10.80 (s, 1H), 8.10 (ddd, J = 7.9, 1.7, 0.5 Hz, 1H), 7.94 (ddd, J = 8.7, 7.0, 1.7 Hz, 1H), 7.88–7.78 (m, 3H), 7.58 (ddd, J = 8.1, 7.0, 1.2 Hz, 1H), 7.33–7.21 (m, 2H), 6.99 (s, 1H). ^13^C NMR (75 MHz, DMSO-d_6_) δ 177.7, 159.5 (d, J = 242.1 Hz), 158.2, 156.1, 155.6, 135.5, 134.4 (d, J = 2.7 Hz), 126.6, 125.4, 124.2, 123.6 (2C, d, J = 8.1 Hz), 119.5, 116.0 (2C, d, J = 22.4 Hz), 111.6. *m/z* calcd. for C_16_H_10_FNO_3_ [M + H]^+^ 284.0717. Found: 284.0715.

N-(4-Chlorophenyl)-4-oxo-4H-chromene-2-carboxamide (**3f**) 

White solid. Yield: 93%. Mp 265 °C. ^1^H NMR (300 MHz, DMSO-d_6_) δ 10.85 (s, 1H), 8.10 (dd, J = 7.9, 1.6 Hz, 1H), 7.95 (ddd, J = 8.7, 7.0, 1.7 Hz, 1H), 7.89–7.81 (m, 3H), 7.58 (ddd, J = 8.1, 7.1, 1.2 Hz, 1H), 7.53–7.46 (m, 2H), 7.00 (s, 1H). ^13^C NMR (75 MHz, DMSO-d_6_) δ 177.7, 158.3, 155.9, 155.6, 137.0, 135.6, 129.3 (2C), 129.2, 126.63, 125.4, 124.2, 123.1 (2C), 119.5, 111.7.

N-(4-Bromophenyl)-4-oxo-4H-chromene-2-carboxamide (**3g**) 

White solid. Yield: 97%. Mp 279.4 °C. ^1^H NMR (300 MHz, DMSO-d_6_) δ 10.87 (s, 1H), 8.10 (dd, J = 8.0, 1.6 Hz, 1H), 7.95 (ddd, J = 8.6, 7.0, 1.7 Hz, 1H), 7.90–7.76 (m, 3H), 7.70–7.55 (m, 3H), 7.00 (s, 1H). ^13^C NMR (75 MHz, DMSO-d_6_) δ 177.8, 158.3, 155.9, 155.6, 137.5, 135.6, 132.2 (2C), 126.7, 125.4, 124.2, 123.5 (2C), 119.5, 117.3, 111.7.

N-(2-Fluorophenyl)-4-oxo-4H-chromene-2-carboxamide (**3h**)

White solid. Yield: 93%. Mp 225 °C. ^1^H NMR (300 MHz, DMSO-d_6_) δ 10.77 (s, 1H), 8.11 (dd, J = 8.0, 1.7 Hz, 1H), 7.95 (ddd, J = 8.7, 7.0, 1.7 Hz, 1H), 7.83 (dd, J = 8.5, 1.2 Hz, 1H), 7.62 (dddd, J = 18.1, 8.1, 7.0, 1.2 Hz, 2H), 7.43–7.34 (m, 2H), 7.30 (ddd, J = 7.7, 5.4, 3.9 Hz, 1H), 6.98 (s, 1H). ^13^C NMR (75 MHz, DMSO-d_6_) δ 177.7, 158.6, 156.4 (d, J = 247.9 Hz), 155.6 (2C), 135.6, 128.6 (d, J = 7.7 Hz), 127.9, 126.7, 125.5, 125.1 (d, J = 3.7 Hz), 124.6 (d, J = 12.4 Hz), 124.2, 119.4, 116.6 (d, J = 19.5 Hz), 111.7. *m/z* calcd. for C_16_H_10_FNO_3_ [M + H]^+^ 284.0717. Found: 284.0717.

N-(2-Chlorophenyl)-4-oxo-4H-chromene-2-carboxamide (**3i**)

White solid. Yield: 98%. Mp 181 °C. ^1^H NMR (300 MHz, DMSO-d_6_) δ 10.72 (s, 1H), 8.11 (dd, J = 8.0, 1.6 Hz, 1H), 7.95 (ddd, J = 8.7, 7.1, 1.7 Hz, 1H), 7.83 (dd, J = 8.6, 1.2 Hz, 1H), 7.73–7.55 (m, 3H), 7.43 (dtd, J = 22.8, 7.5, 1.7 Hz, 2H), 6.99 (s, 1H). ^13^C NMR (75 MHz, DMSO-d_6_) δ 177.7, 158.6, 155.6 (2C), 135.7, 134.0, 130.3, 129.9, 128.9, 128.6, 128.3, 126.7, 125.5, 124.2, 119.4, 111.7. *m/z* calcd. for C_16_H_10_ClNO_3_ [M + H]^+^ 300.0422. Found: 300.0424.

N-(2-Bromophenyl)-4-oxo-4H-chromene-2-carboxamide (**3j**)

White solid. Yield: 82%. Mp 195 °C. ^1^H NMR (300 MHz, DMSO-d_6_) δ 10.69 (s, 1H), 8.10 (dd, J = 8.0, 1.7 Hz, 1H), 7.94 (ddd, J = 8.7, 7.1, 1.7 Hz, 1H), 7.82 (dd, J = 8.5, 1.1 Hz, 1H), 7.79 (dd, J = 8.1, 1.4 Hz, 1H), 7.67 (dd, J = 7.9, 1.6 Hz, 1H), 7.58 (ddd, J = 8.1, 7.1, 1.1 Hz, 1H), 7.50 (td, J = 7.6, 1.4 Hz, 1H), 7.31 (ddd, J = 8.0, 7.5, 1.7 Hz, 1H), 6.98 (s, 1H). ^13^C NMR (75 MHz, DMSO-d_6_) δ 177.7, 158.5, 155.6, 155.6, 135.7, 135.6, 133.4, 129.2, 128.9, 128.8, 126.7, 125.5, 124.2, 120.6, 119.4, 111.7. *m/z* calcd. for C_16_H_10_BrNO_3_ [M + H]^+^ 343.9917. Found: 343.9914.

N-(2-Chloro-4-fluorophenyl)-4-oxo-4H-chromene-2-carboxamide (**3k**)

White solid. Yield: 94%. Mp 229 °C. ^1^H NMR (300 MHz, DMSO-d_6_) δ 10.78 (s, 1H), 8.11 (dd, J = 7.9, 1.7 Hz, 1H), 7.95 (ddd, J = 8.7, 7.1, 1.7 Hz, 1H), 7.82 (dd, J = 8.5, 1.2 Hz, 1H), 7.73–7.63 (m, 2H), 7.59 (ddd, J = 8.1, 7.1, 1.2 Hz, 1H), 7.36 (td, J = 8.5, 2.9 Hz, 1H), 6.98 (s, 1H). ^13^C NMR (126 MHz, DMSO-d_6_) δ 177.74 160.7 (d, J = 247.4 Hz), 158.8, 155.6 (2C), 135.7, 131.5 (d, J = 11.1 Hz), 130.8 (d, J = 15 Hz), 130.5 (d, J = 9.3 Hz), 126.7, 125.5, 124.1, 119.4, 117.5 (d, J = 26.0 Hz), 115.4 (d, J = 22.2 Hz), 111.7. *m/z* calcd. for C_16_H_9_BrClNO_3_ [M + H]^+^ 318.0328. Found: 318.0324; [M+Na]^+^ 340.0147. Found: 340.0144.

N-(3-Chloro-4-fluorophenyl)-4-oxo-4H-chromene-2-carboxamide (**3l**)

White solid. Yield: 90%. Mp 291 °C. ^1^H NMR (300 MHz, DMSO-d_6_) δ 10.92 (s, 1H), 8.11–8.08 (m, 2H), 7.95 (ddd, J = 8.7, 7.1, 1.7 Hz, 1H), 7.85 (dd, J = 8.5, 0.6 Hz, 1H), 7.80 (m, 1H), 7.59 (bt, J = 9.1 Hz, 1H), 7.51 (bt, J = 9.1 Hz, 1H), 7.00 (s, 1H). ^13^C NMR (75 MHz, DMSO-d_6_) δ 177.7, 158.5, 155.7, 155.6, 153.6, 135.7, 135.3, 126.7, 125.5, 124.2, 123.2, 122.1, 119.8, 119.4, 117.6, 111.8. *m/z* calcd. For C_16_H_9_ClFNO_3_ [M + H]^+^ 318.0328. Found: 318.0341. Anal. Calcd.: C, 60.49; H, 2.86; N, 4.41%. Found: C, 60.15; H, 2.51; N, 4.34%.

N-(4-Nitrophenyl)-4-oxo-4H-chromene-2-carboxamide (**3m**)

White solid. Yield: 88%. Mp 307 °C. ^1^H NMR (300 MHz, DMSO-d_6_) δ 11.20 (s, 1H), 8.34 (d, J = 9.3 Hz, 2H), 8.16–8.06 (m, 3H), 7.96 (ddd, J = 8.6, 7.0, 1.7 Hz, 1H), 7.86 (dd, J = 8.7, 1.2 Hz, 1H), 7.59 (ddd, J = 8.1, 7.0, 1.2 Hz, 1H), 7.05 (s, 1H). ^13^C NMR (75 MHz, DMSO-d_6_) δ 177.7, 159.0, 155.6, 155.5, 144.3, 143.9, 135.7, 126.7, 125.5, 125.3 (2C), 124.2, 121.3 (2C), 119.5, 112.1.

N-(2,4-Dinitrophenyl)-4-oxo-4H-chromene-2-carboxamide (**3n**)

Yellow solid. Yield: 92%. Mp 330 °C. ^1^H NMR (300 MHz, DMSO-d_6_) δ 11.85 (s, 1H), 8.82 (d, J = 2.7 Hz, 1H), 8.19 (dd, J = 9.4, 2.7 Hz, 1H), 8.10 (bd, J = 7.8 Hz, 1H), 7.92 (m, 1H), 7.77 (bd, J = 8.3 Hz, 1H), 7.57 (m, 1H), 7.13 (d, J = 9.4 Hz, 1H), 7.02 (s, 1H). ^13^C NMR (75 MHz, DMSO-d_6_) δ 173.9, 159.7, 155.9, 154.5, 148.9, 148.5, 137.2, 135.2, 130.1, 129.3, 127.1, 124.5, 123.4, 120.1, 116.3, 95.3. *m/z* calcd. for C_16_H_9_N_3_O_7_ [M + H]^+^ 356.0513. Found: 356.0513. Anal. Calcd.: C, 54.09; H, 2.55; N, 11.83%. Found: C, 53.26; H, 2.03; N,11.91%.

N-(3,5-Dinitrophenyl)-4-oxo-4H-chromene-2-carboxamide (**3o**)

Light yellow solid. Yield: 88%. Mp 312 °C. ^1^H NMR (300 MHz, DMSO-d_6_) δ 10.68 (s, 1H), 11.50 (s, 1H), 9.18 (d, J = 2.1 Hz, 2H), 8.66 (s, 1H), 8.11 (d, J = 7.9 Hz, 1H), 7.99 (t, J = 7.8 Hz, 1H), 7.86 (d, J = 8.4 Hz, 1H), 7.61 (t, J = 7.5 Hz, 1H), 7.08 (s, 1H). ^13^C NMR (75 MHz, DMSO-d_6_) δ 177.7, 159.33, 155.5, 155.0, 148.7 (2C), 140.3, 135.8, 126.8, 125.5, 124.2, 120.8 (2C), 119.3, 114.4, 112.2. *m/z* calcd. for C_16_H_9_N_3_O_7_ [M + H]^+^ 356.0513. Found: 356.0522.

N-(4-Methoxyphenyl)-4-oxo-4H-chromene-2-carboxamide (**3p**)

Yellow solid. Yield: 86%. Mp 219 °C. ^1^H NMR (300 MHz, DMSO-d_6_) δ 10.68 (s, 1H), 8.10 (ddd, J = 8.0, 1.7, 0.5 Hz, 1H), 7.95 (ddd, J = 8.6, 7.0, 1.7 Hz, 1H), 7.86 (ddd, J = 8.5, 1.2, 0.5 Hz, 1H), 7.76–7.69 (m, 2H), 7.58 (ddd, J = 8.1, 7.0, 1.2 Hz, 1H), 7.05–6.99 (m, 2H), 6.97 (s, 1H), 3.79 (s, 3H). ^13^C NMR (75 MHz, DMSO-d_6_) δ 177.8, 157.8, 156.9, 156.3, 155.6, 135.5, 131.0, 126.6, 125.4, 124.2, 123.2 (2C), 119.5, 114.4 (2C), 111.4, 55.7. *m/z* calcd. for C_17_H_13_NO_4_ [M + H]^+^ 296.0917. Found: 296.09122; [M+Na]^+^ 318.0737. Found: 318.0732.

N-(2-Methoxyphenyl)-4-oxo-4H-chromene-2-carboxamide (**3q**)

Light yellow solid. Yield: 99%. Mp 199 °C. ^1^H NMR (300 MHz, DMSO-d_6_) 10.04 (s, 1H), 8.09 (dd, J = 7.9, 1.6 Hz, 1H), 7.93 (ddd, J = 8.6, 7.1, 1.7 Hz, 1H), 7.84 (dd, J = 7.8, 1.5 Hz, 2H), 7.58 (ddd, J = 8.1, 7.1, 1.1 Hz, 1H), 7.27 (ddd, J = 8.2, 7.5, 1.7 Hz, 1H), 7.17 (dd, J = 8.3, 1.3 Hz, 1H), 7.03 (td, J = 7.6, 1.3 Hz, 1H), 6.95 (s, 1H), 3.91 (s, 3H). ^13^C NMR (75 MHz, DMSO-d_6_) δ 177.8, 157.9, 155.9, 155.5, 151.8, 135.6, 127.3, 126.7, 125.8, 125.5, 124.3, 124.1, 120.9, 119.5, 112.1, 111.4, 56.4. *m/z* calcd. for C_17_H_13_NO_4_ [M + H]^+^ 296.0917. Found: 296.0916.

N-(2-(Methylthio)phenyl)-4-oxo-4H-chromene-2-carboxamide (**3r**)

Light yellow solid. Yield: 90%. Mp 163 °C. ^1^H NMR (300 MHz, DMSO-d_6_) δ 10.59 (s, 1H), 8.11 (dd, J = 7.9, 1.7 Hz, 1H), 7.95 (ddd, J = 8.7, 7.1, 1.7 Hz, 1H), 7.82 (d, J = 8.4 Hz, 1H), 7.63–7.44 (m, 3H), 7.34 (dtd, J = 23.3, 7.4, 1.6 Hz, 2H), 6.97 (s, 1H), 3.35 (s, 3H). ^13^C NMR (75 MHz, DMSO-d_6_) δ 177.7, 158.5, 155.8, 155.6, 135.7, 135.4, 134.4, 128.1, 127.7, 127.0, 126.7, 126.2, 125.5, 124.2, 119.4, 111.6, 15.7. *m/z* calcd. for C_17_H_13_NO_3_S [M + H]^+^ 312.0689. Found: 312.0684; [M + Na]^+^ 334.0508. Found: 334.0505.

N-(3,4-Dimethoxyphenyl)-4-oxo-4H-chromene-2-carboxamide (**3s**)

Yellow solid. Yield: 83%. Mp 229 °C. ^1^H NMR (300 MHz, DMSO-d_6_) δ 10.64 (s, 1H), 8.10 (ddd, J = 8.0, 1.7, 0.5 Hz, 1H), 7.95 (ddd, J = 8.6, 7.0, 1.7 Hz, 1H), 7.86 (ddd, J = 8.5, 1.3, 0.5 Hz, 1H), 7.58 (ddd, J = 8.1, 7.0, 1.2 Hz, 1H), 7.49 (d, J = 2.4 Hz, 1H), 7.41 (dd, J = 8.7, 2.4 Hz, 1H), 7.01 (d, J = 8.8 Hz, 1H), 6.98 (s, 1H), 3.80 (s, 3H), 3.78 (s, 3H). ^13^C NMR (75 MHz, DMSO-d_6_) δ 177.8, 157.7, 156.3, 155.6, 149.0, 146.5, 135.5, 131.4, 126.6, 125.4, 124.2, 119.5, 113.7, 112.2, 111.4, 106.4, 56.1, 55.9. *m/z* calcd. for C_18_H_15_NO_5_ [M + H]^+^ 326.1023. Found: 326.1027.

N-(3,5-Dimethoxyphenyl)-4-oxo-4H-chromene-2-carboxamide (**3t**)

Light yellow solid. Yield: 90%. Mp 211 °C. ^1^H NMR (300 MHz, DMSO-d_6_) δ 10.63 (s, 1H), 8.10 (ddd, J = 7.9, 1.7, 0.5 Hz, 1H), 7.95 (ddd, J = 8.6, 7.0, 1.7 Hz, 1H), 7.87 (ddd, J = 8.5, 1.3, 0.5 Hz, 1H), 7.58 (ddd, J = 8.1, 7.0, 1.3 Hz, 1H), 7.11 (d, J = 2.3 Hz, 2H), 6.99 (s, 1H), 6.38 (t, J = 2.3 Hz, 1H), 3.78 (s, 6H). ^13^C NMR (75 MHz, DMSO-d_6_) δ 177.8, 161.0 (2C), 158.2, 156.0, 155.6, 139.7, 135.5, 126.6, 125.4, 124.2, 119.5, 111.6, 99.7 (2C), 97.4, 55.7. *m/z* calcd. for C_18_H_15_NO_5_ [M + H]^+^ 326.1023. Found: 326.1032.

N-(4-Morpholinophenyl)-4-oxo-4H-chromene-2-carboxamide (**3u**)

Orange solid. Yield: 95%. Mp 288 °C. ^1^H NMR (300 MHz, DMSO-d_6_) δ 10.75 (s, 1H), 8.09 (dd, J = 7.9, 1.6 Hz, 1H), 8.00–7.83 (m, 2H), 7.83–7.72 (m, 2H), 7.58 (ddd, J = 8.1, 6.8, 1.4 Hz, 1H), 7.23 (d, J = 8.6 Hz, 2H), 6.98 (s, 1H), 3.85 (dd, J = 6.1, 3.4 Hz, 4H), 3.25 (t, J = 4.7 Hz, 4H). ^13^C NMR (75 MHz, DMSO-d_6_) δ 177.8, 157.8, 156.3, 155.6, 146.7, 135.5, 131.8, 126.6, 125.4, 124.1, 122.6 (2C), 119.5, 117.0 (2C), 111.4, 66.0 (2C), 50.2 (2C). *m/z* calcd. for C_20_H_18_N_2_O_4_ [M + H]^+^ 351.1339. Found: 351.1329.

4-Oxo-N-(pyrimidin-2-yl)-4H-chromene-2-carboxamide (**3v**)

White solid. Yield: 97%. Mp 189 °C. ^1^H NMR (300 MHz, DMSO-d_6_) δ 11.48 (s, 1H), 8.80 (d, J = 4.9 Hz, 2H), 8.07 (ddd, J = 8.0, 1.7, 0.5 Hz, 1H), 7.90 (ddd, J = 8.6, 7.0, 1.7 Hz, 1H), 7.84 (ddd, J = 8.5, 1.3, 0.5 Hz, 1H), 7.55 (ddd, J = 8.1, 6.9, 1.3 Hz, 1H), 7.35 (t, J = 4.8 Hz, 1H), 6.95 (s, 1H). ^13^C NMR (75 MHz, DMSO-d_6_) δ 177.7, 159.1 (2C), 158.6, 157.5, 156.0, 155.6, 135.5, 126.6, 125.3, 124.2, 119.6, 118.7, 111.9. *m/z* calcd. for C_14_H_9_N_3_O_3_ [M + H]^+^ 268.0717. Found: 268.0715. 

4-Oxo-N-propyl-4H-chromene-2-carboxamide (**3w**) 

White cristals. Yield: 23%. Mp 166 °C. ^1^H NMR (300 MHz, CDCl_3_) δ 8.26 (ddd, J = 8.0, 1.8, 0.5 Hz, 1H), 7.77 (ddd, J = 8.7, 7.2, 1.7 Hz, 1H), 7.59–7.53 (m, 1H), 7.49 (ddd, J = 8.1, 7.1, 1.1 Hz, 1H), 7.21 (s, 1H), 6.96 (s, 1H), 3.51 (ddd, J = 8.0, 7.2, 6.1 Hz, 2H), 1.83–1.64 (m, 2H), 1.05 (t, J = 7.4 Hz, 3H). ^13^C NMR (75 MHz, DMSO-d_6_) δ 178.2, 159.2, 155.3, 154.8, 134.5, 126.2, 126.0, 124.4, 118.0, 112.1, 41.7, 22.8, 11.4. *m/z* calcd. for C_13_H_13_NO_3_ [M + H]^+^ 232.0968. Found: 232.0975.

N-Cyclohexyl-4-oxo-4H-chromene-2-carboxamide (**3x**)

Light Yellow solid. Yield: 99%. Mp 179 °C. ^1^H NMR (300 MHz, DMSO-d_6_) δ (ppm) 8.84 (d, J = 8.1 Hz, 1H), 8.05 (dd, J = 8.0, 1.7 Hz, 1H), 7.90 (ddd, J = 8.7, 7.1, 1.7 Hz, 1H), 7.78 (dd, J = 8.5, 1.1 Hz, 1H), 7.54 (ddd, J = 8.1, 7.1, 1.1 Hz, 1H), 6.83 (s, 1H), 3.78 (tdt, J = 11.5, 8.1, 3.9 Hz, 1H), 1.89–1.81 (m, 2H), 1.76 (dt, J = 13.3, 3.5 Hz, 2H), 1.63 (dtd, J = 12.2, 3.3, 1.6 Hz, 1H), 1.46–1.25 (m, 4H), 1.15 (dddd, J = 16.1, 12.5, 8.1, 3.6 Hz, 1H). ^13^C NMR (75 MHz, DMSO-d_6_) δ 177.8, 158.5, 156.4, 155.6, 135.4, 126.4, 125.4, 124.1, 119.4, 111.0, 49.2, 32.5 (2C), 25.6, 25.3 (2C). *m/z* calcd. for C_16_H_17_NO_3_ [M + H]^+^ 272.1281. Found: 272.1277.

N-((3S,5S,7S)-Adamantan-1-yl)-4-oxo-4H-chromene-2-carboxamide (**3y**)

White solid. Yield: 90%. Mp 201 °C. ^1^H NMR (300 MHz, DMSO-d_6_) δ 8.14 (s, 1H), 8.06 (dd, J = 8.0, 1.6 Hz, 1H), 7.90 (ddd, J = 8.6, 6.9, 1.7 Hz, 1H), 7.82 (dd, J = 8.5, 1.3 Hz, 1H), 7.55 (ddd, J = 8.1, 6.9, 1.3 Hz, 1H), 6.80 (s, 1H), 2.11 (s, 9H), 1.69 (s, 6H).; ^13^C NMR (75 MHz, DMSO-d_6_) δ 177.8, 158.6, 156.9, 155.6, 135.3, 126.4, 125.3, 124.1, 119.6, 110.7, 52.8, 41.0 (3C), 36.4 (3C), 29.3 (3C). *m/z* calcd. for C_20_H_21_NO_3_ [M + H]^+^ 324.1594. Found: 324.1592. 

N-(3-Ethylphenyl)-4-oxo-4H-chromene-2-carboxamide (**3′a**)

White solid. Yield: 71%. Mp 168 °C.^1^H NMR (300 MHz, DMSO-d_6_) δ 10.69 (s, 1H), 8.10 (dd, J = 8.0, 1.6 Hz, 1H), 7.95 (ddd, J = 8.6, 7.0, 1.7 Hz, 1H), 7.87 (dd, J = 8.6, 1.3 Hz, 1H), 7.69–7.63 (m, 2H), 7.58 (ddd, J = 8.1, 6.9, 1.3 Hz, 1H), 7.38–7.29 (m, 1H), 7.07 (dt, J = 7.7, 1.3 Hz, 1H), 6.99 (s, 1H), 2.65 (q, J = 7.6 Hz, 2H), 1.23 (t, J = 7.6 Hz, 3H). ^13^C NMR (75 MHz, DMSO-d_6_) δ 177.8, 158.1, 156.2, 155.6, 144.9, 138.0, 135.5, 129.2, 126.6, 125.4, 125.0, 124.2, 120.9, 119.5, 119.0, 111.5, 28.7, 16.0. *m/z* calcd. for C_18_H_15_NO_3_ [M + H]^+^ 294.1125. Found: 294.1121.

N-(3-((Diethylamino)methyl)-4-hydroxyphenyl)-4-oxo-4H-chromene-2-carboxamide (**3′c**)

Yellow solid. Yield: 26%. Mp 186 °C. ^1^H NMR (300 MHz, DMSO-d_6_) δ 10.74 (s, 1H), 8.09 (dd, J = 8.0, 1.6 Hz, 1H), 8.00–7.84 (m, 2H), 7.75 (d, J = 2.5 Hz, 1H), 7.67–7.53 (m, 2H), 7.03–6.87 (m, 2H), 4.02 (s, 2H), 3.48 (s, 1H), 2.88 (d, J = 7.1 Hz, 4H), 1.19 (t, J = 7.1 Hz, 6H). ^13^C NMR (75 MHz, DMSO-d_6_) δ 177.8, 157.7, 156.4, 155.6, 154.8, 135.5, 129.4, 126.6, 125.4 (2C), 124.7, 124.2, 123.7, 119.5, 116.0, 111.3, 52.7, 46.7 (2C), 10.3 (2C).

N-(4-((1H-Imidazol-1-yl)methyl)phenyl)-4-oxo-4H-chromene-2-carboxamide (**3′d**)

White solid. Yield: 85%. Mp 261 °C. ^1^H NMR (300 MHz, DMSO-d_6_) δ 10.83 (s, 1H), 8.11–8.05 (m, 2H), 7.93 (ddd, J = 8.6, 7.0, 1.7 Hz, 1H), 7.86 (dd, J = 8.5, 1.1 Hz, 1H), 7.82–7.79 (m, 2H), 7.57 (ddd, J = 8.0, 7.0, 1.2 Hz, 1H), 7.39–7.31 (m, 3H), 7.08 (d, J = 1.3 Hz, 1H), 6.98 (s, 1H), 5.25 (s, 2H). ^13^C NMR (75 MHz, DMSO-d_6_) δ 177.8, 158.3, 156.1, 155.6, 137.7, 135.6, 137.5, 134.3, 128.7 (2C), 127.7, 126.6, 125.4, 124.2, 121.8 (2C), 120.2, 119.5, 111.6, 49.9. *m/z* calcd. for C_20_H_15_N_3_O_3_ [M + H]^+^ 346.1186. Found: 346.1173.

Tert-butyl 4-((4-oxo-4H-chromene-2-carboxamido)methyl)piperidine-1-carboxylate (**3′e**)

Light yellow solid. Yield: 54%. Mp 129 °C. ^1^H NMR (300 MHz, DMSO-d_6_) δ 9.18 (t, J = 6.0 Hz, 1H), 8.07 (ddd, J = 8.0, 1.7, 0.5 Hz, 1H), 7.91 (ddd, J = 8.7, 7.1, 1.7 Hz, 1H), 7.76 (ddd, J = 8.4, 1.1, 0.4 Hz, 1H), 7.56 (ddd, J = 8.1, 7.1, 1.1 Hz, 1H), 6.84 (s, 1H), 3.95 (d, J = 13.0 Hz, 2H), 3.22 (t, J = 6.4 Hz, 2H), 2.73 (d, J = 12.6 Hz, 2H), 1.79 (m, 1H), 1.68 (dd, J = 13.2, 3.6 Hz, 2H), 1.40 (s, 9H), 1.06 (qd, J = 12.3, 4.3 Hz, 2H). ^13^C NMR (126 MHz, DMSO-d_6_) δ177.8, 159.6, 156.2, 155.6, 154.4, 135.4, 126.5, 125.4, 124.1, 119.3, 110.9, 79.0, 45.0, 44.0, 43.3, 36.1, 30.0 (2C), 28.5 (3C). *m/z* calcd. for C_21_H_26_N_2_O_5_ [M-Boc+H]^+^ 287.1390. Found: 287.1393.

N-(3-(1H-Imidazol-1-yl)propyl)-4-oxo-4H-chromene-2-carboxamide (**3′f**)

White solid. Yield: 82%. Mp 171 °C. ^1^H NMR (300 MHz, DMSO-d_6_) δ 9.18 (t, J = 5.8 Hz, 1H), 8.07 (dd, J = 8.0, 1.6 Hz, 1H), 7.92 (ddd, J = 8.7, 7.1, 1.7 Hz, 1H), 7.80–7.67 (m, 2H), 7.56 (ddd, J = 8.1, 7.1, 1.1 Hz, 1H), 7.24 (s, 1H), 6.92 (s, 1H), 6.86 (s, 1H), 4.06 (t, J = 6.9 Hz, 2H), 3.31 (q, J = 6.6 Hz, 2H), 2.02 (p, J = 6.9 Hz, 2H). ^13^C NMR (75 MHz, DMSO-d_6_) δ 177.8, 159.6, 156.1, 155.6, 139.8, 135.5, 128.9, 126.5, 125.4, 124.1, 119.8, 119.3, 110.9, 44.2, 37.2, 30.9. *m/z* calcd. for C_16_H_15_N_3_O_3_ [M + H]^+^ 298.1186. Found: 298.1193. 

N-(5-(Diethylamino)pentan-2-yl)-4-oxo-4H-chromene-2-carboxamide (**3′g**) 

White solid. Yield: 57%. Mp 110 °C. ^1^H NMR (300 MHz, DMSO-d_6_) δ 9.03 (d, J = 8.4 Hz, 1H), 8.07 (dd, J = 7.9, 1.6 Hz, 1H), 7.92 (ddd, J = 8.5, 6.9, 1.7 Hz, 1H), 7.85 (dd, J = 8.6, 1.3 Hz, 1H), 7.56 (ddd, J = 8.1, 6.9, 1.3 Hz, 1H), 6.87 (s, 1H), 4.07 (t, J = 7.5 Hz, 1H), 3.06 (dq, J = 10.0, 5.2 Hz, 6H), 1.80–1.51 (m, 4H), 1.32–1.12 (m, 9H). ^13^C NMR (75 MHz, DMSO-d_6_) δ 177.9, 159.0, 156.3, 155.6, 135.4, 126.5, 125.4, 124.1, 119.5, 111.0, 50.8, 46.5 (2C), 45.4, 33.0, 20.9 (2C), 20.5, 8.9 (2C). *m/z* calcd. for C_19_H_26_N_2_O_3_ [M + H]^+^ 331.2016. Found: 331.2033.

2-(4-(2-Oxo-2-(pyrrolidin-1-yl)ethyl)piperazine-1-carbonyl)-4H-chromen-4-one (**3′i**) 

White solid. Yield: 55%. Mp 117 °C. ^1^H NMR (300 MHz, DMSO-d_6_) δ 8.13–8.03 (m, 1H), 7.87 (ddd, J = 8.7, 7.1, 1.7 Hz, 1H), 7.71 (dd, J = 8.6, 1.1 Hz, 1H), 7.55 (ddd, J = 8.1, 7.1, 1.1 Hz, 1H), 6.55 (s, 1H), 3.60 (dt, J = 23.9, 4.9 Hz, 4H), 3.46 (t, J = 6.7 Hz, 2H), 3.29 (t, J = 6.8 Hz, 2H), 3.19 (s, 2H), 2.65–2.54 (m, 4H), 1.93–1.70 (m, 4H). ^13^C NMR (75 MHz, DMSO-d_6_) δ 177.1, 167.6, 160.5, 158.5, 155.9, 135.3, 126.5, 125.4, 124.2, 119.1, 111.0, 60.3, 53.1, 52.3, 47.0, 46.0, 45.8, 42.2, 26.2, 24.1.

#### 3.2.4. General Procedure for the Synthesis of Retro Chromone Carboxamides 

(Series 2, **6a**–**y**)

A suspension of 4-oxo-4H-chromene-2-carbonyl chloride **2** (0.5 g, 2.4 mmol) and dry cyclohexane (4.0 mL) was cooled to 0 °C. Then, a solution of sodium azide (0.18 g, 2.7 mmol) in water (0.7 mL) was added dropwise and the resulting mixture was allowed to stir for 30 min at 0 °C and then for 1 h at room temperature. The white viscous solid was filtered off, washed with cold petroleum ether and dried under vacuum. The white powder obtained was transferred to a round bottom flask and dry cyclohexane (22 mL) was added. The system was provided with a calcium pipe and allowed to stir at 80 °C until the color of the suspension turned intense orange, then the suitable acid (2.4 mmol) was added quickly in one portion (bubbles were observed) and the system was allowed to stir at reflux overnight. After this time, the solid obtained was filtered off, washed with 10 mL of a saturated solution of NaHCO_3_ and recrystallized in DMF/H_2_O mixture (50:50) to give the final compound. 

N-(4-Oxo-4H-chromen-2-yl) benzamide (**6a**)

Light yellow needles. Yield: 87%. Mp 218 °C. ^1^H NMR (300 MHz, DMSO-d_6_): δ 11.54 (s, 1H), 8.03 (m, 3H), 7.81 (ddd, J = 8.7, 7.1, 1.8 Hz, 1H), 7.71–7.63 (m, 1H), 7.62–7.46 (m, 4H), 6.98 (s, 1H). ^13^C NMR (75 MHz, DMSO-d_6_) δ 177.6, 166.4, 157.7, 154.2, 134.4, 133.5, 133.2, 129.0 (2C), 128.8 (2C), 125.9, 125.4, 123.4, 118.0, 97.5. *m/z* calcd. for C_16_H_11_NO_3_ [M + H]^+^ 266.0811. Found: 266.0815.

4-Methyl-N-(4-oxo-4H-chromen-2-yl) benzamide (**6b**) 

Yellow needles. Yield: 31%. Mp 240 °C. ^1^H NMR (300 MHz, DMSO-d_6_) δ 11.47 (s, 1H), 8.03 (dd, J = 7.8, 1.7 Hz, 1H), 7.97–7.91 (m, 2H), 7.80 (ddd, J = 8.7, 7.2, 1.8 Hz, 1H), 7.58 (dd, J = 8.5, 1.0 Hz, 1H), 7.50 (ddd, J = 8.1, 7.1, 1.1 Hz, 1H), 7.40–7.34 (m, 2H), 6.97 (s, 1H), 2.41 (s, 3H). ^13^C NMR (100 MHz, DMSO-d_6_) δ 177.3, 161.0, 158.5, 156.5, 152.0, 125.3, 123.5, 122.2, 110.2, 109.9. *m/z* calcd. for C_17_H_13_NO_3_ [M + H]^+^ 280.0968. Found: 280.0973.

4-Ethyl-N-(4-oxo-4H-chromen-2-yl) benzamide (**6c**) 

Yellow solid. Yield: 24%. Mp 237 °C. ^1^H NMR (300 MHz, DMSO-d_6_) δ 11.44 (s, 1H), 8.03 (dd, J = 7.9, 1.7 Hz, 1H), 7.99–7.93 (m, 2H), 7.80 (ddd, J = 8.7, 7.2, 1.8 Hz, 1H), 7.58 (dd, J = 8.5, 1.0 Hz, 1H), 7.49 (ddd, J = 8.1, 7.2, 1.1 Hz, 1H), 7.42–7.36 (m, 2H), 6.98 (s, 1H), 2.70 (q, J = 7.6 Hz, 2H), 1.22 (t, J = 7.6 Hz, 3H). ^13^C NMR (75 MHz, DMSO-d_6_) δ 177.6, 166.2, 157.8, 154.2, 149.6, 134.4, 131.0, 129.0 (2C), 128.4 (2C), 125.8, 125.3, 123.4, 118.0, 97.4, 28.6, 15.7. *m/z* calcd. for C_18_H_15_NO_3_ [M + H]^+^ 294.1125. Found: 294.1133.

N-(4-Oxo-4H-chromen-2-yl)-4-propylbenzamide (**6d**)

Light yellow solid. Yield: 88%. Mp 229 °C. ^1^H NMR (300 MHz, DMSO-d_6_) δ 11.44 (s, 1H), 8.03 (dd, J = 7.9, 1.7 Hz, 1H), 7.99–7.91 (m, 2H), 7.80 (ddd, J = 8.7, 7.1, 1.7 Hz, 1H), 7.58 (dd, J = 8.5, 1.0 Hz, 1H), 7.50 (ddd, J = 8.1, 7.2, 1.1 Hz, 1H), 7.42–7.34 (m, 2H), 6.97 (s, 1H), 2.69–2.63 (t, j = 9.0 Hz, 2H), 1.71–1.57 (m, 2H), 0.92 (t, J = 7.3 Hz, 3H). ^13^C NMR (75 MHz, DMSO-d_6_) δ 177.6, 166.3, 157.9, 154.2, 148.0, 134.4, 131.1, 128.9 (2C), 128.9 (2C), 125.8, 125.3, 123.5, 118.0, 97.4, 37.6, 24.2, 14.1. *m/z* calcd. for C_19_H_17_NO_3_ [M + H]^+^ 308.1287. Found: 308.1296.

4-Fluoro-N-(4-oxo-4H-chromen-2-yl) benzamide (**6e**) 

White solid. Yield: 40%. Mp 251 °C. ^1^H NMR (300 MHz, DMSO-d_6_) δ 11.55 (s, 1H), 8.16–8.06 (m, 2H), 8.03 (dd, J = 7.9, 1.7 Hz, 1H), 7.79 (ddd, J = 8.7, 7.1, 1.8 Hz, 1H), 7.57 (d, J = 8.3 Hz, 1H), 7.49 (t, J = 7.5 Hz, 1H), 7.40 (t, J = 8.9 Hz, 2H), 6.96 (s, 1H)^. 13^C NMR (75 MHz, DMSO-d_6_) δ 177.6, 165.3, 165.2 (d, J = 251.0 Hz), 157.6, 154.1, 134.4, 131.7 (2C, d, J = 9.3 Hz), 130.0 (d, J = 2.9 Hz), 125.9, 125.3, 123.4, 118.0, 116.0 (2C, d, J = 22.0 Hz), 97.5. *m/z* calcd. for C_16_H_10_FNO_3_ [M + H]^+^ 284.0717. Found: 284.0721.

4-Chloro-N-(4-oxo-4H-chromen-2-yl) benzamide (**6f**) 

White solid. Yield: 46%. Mp 296 °C. ^1^H NMR (300 MHz, DMSO-d_6_): δ 11.63 (s, 1H), 8.08–7.99 (m, 3H), 7.81 (ddd, J = 8.7, 7.2, 1.8 Hz, 1H), 7.68–7.60 (m, 2H), 7.58 (dd, J = 8.6, 1.0 Hz, 1H), 7.50 (ddd, J = 8.1, 7.2, 1.1 Hz, 1H), 6.96 (s, 1H). ^13^C NMR (75 MHz, DMSO-d_6_) δ 177.6, 165.4, 157.6, 154.2, 138.1, 134.5, 132.3, 130.8 (2C), 129.1 (2C), 125.9, 125.4, 123.4, 118.0, 97.6. *m/z* calcd. for C_16_H_10_ClNO_3_ [M + H]^+^ 300.0422. Found: 300.0425.

4-Bromo-N-(4-oxo-4H-chromen-2-yl) benzamide (**6g**)

Light orange solid. Yield: 93%. Mp 287 °C. ^1^H NMR (300 MHz, DMSO-d_6_) δ 11.63 (s, 1H), 8.04 (dd, J = 7.9, 1.7 Hz, 1H), 7.98–7.93 (m, 2H), 7.80 (m, 3H), 7.58 (dd, J = 8.5, 1.0 Hz, 1H), 7.51 (ddd, J = 8.0, 7.1, 1.1 Hz, 1H), 6.96 (s, 1H). ^13^C NMR (75 MHz, DMSO-d_6_) δ 177.6, 165.6, 157.6, 154.2, 134.5, 132.7, 132.0 (2C), 130.9 (2C), 127.2, 125.9, 125.4, 123.4, 118.0, 97.6. *m/z* calcd. for C_16_H_10_BrNO_3_ [M + H]^+^ 343.9917. Found: 343.9933.

2-Fluoro-N-(4-oxo-4H-chromen-2-yl) benzamide (**6h**)

Pale yellow solid. Yield: 40%. Mp 194 °C. ^1^H NMR (300 MHz, DMSO-d_6_) δ 11.79 (s, 1H), 8.03 (dd, J = 7.9, 1.7 Hz, 1H), 7.78 (dtd, J = 17.6, 7.3, 1.7 Hz, 2H), 7.71–7.61 (m, 1H), 7.59–7.47 (m, 2H), 7.44–7.33 (m, 2H), 6.94 (s, 1H). ^13^C NMR (75 MHz, DMSO-d_6_) δ 177.6, 163.8, 159.6 (d, J = 250.7 Hz), 157.1, 154.1, 134.5, 134.2 (d, J = 8.6 Hz), 130.7 (d, J = 2.3 Hz), 126.0, 125.4, 125.1 (d, J = 3.5 Hz), 123.7 (d, J = 14.0 Hz), 123.4, 118.0, 116.7 (d, J = 21.5 Hz), 97.2. *m/z* calcd. for C_16_H_10_FNO_3_ [M + H]^+^ 284.0717. Found: 284.0725.

2-Chloro-N-(4-oxo-4H-chromen-2-yl) benzamide (**6i**)

Pale yellow solid. Yield: 40%. Mp 228 °C. ^1^H NMR (300 MHz, DMSO-d_6_) δ 11.94 (s, 1H), 8.03 (dd, J = 7.8, 1.7 Hz, 1H), 7.80 (ddd, J = 8.7, 7.1, 1.8 Hz, 1H), 7.68 (dd, J = 7.4, 1.7 Hz, 1H), 7.64–7.55 (m, 2H), 7.51 (m, 3H), 6.92 (s, 1H). ^13^C NMR (75 MHz, DMSO-d_6_) δ 177.5, 165.9, 157.0, 154.0, 135.6, 134.5, 132.4, 130.4, 130.2, 129.7, 127.7, 126.0, 125.4, 123.4, 118.1, 97.1. *m/z* calcd. for C_16_H_10_ClNO_3_ [M + H]^+^ 300.0422 Found: 300.0439.

2-Bromo-N-(4-oxo-4H-chromen-2-yl) benzamide (**6j**)

Orange solid. Yield: 94%. Mp 215–218 °C. ^1^H NMR (300 MHz, DMSO-d_6_) δ 11.93 (s, 1H), 8.07–7.99 (m, 1H), 7.83–7.73 (m, 2H), 7.68–7.61 (m, 1H), 7.58–7.44 (m, 4H), 6.91 (s, 1H). ^13^C NMR (75 MHz, DMSO-d_6_) δ 177.5, 166.7, 157.0, 154.0, 137.7, 134.5, 133.3, 132.5, 129.6, 128.2, 126.0, 125.4, 123.4, 119.3, 118.1, 97.1. *m/z* calcd. for C_16_H_10_BrNO_3_ [M + H]^+^ 343.9917. Found: 343.9905; [M + Na]^+^ 365.9736. Found: 365.9727.

2-Chloro-4-fluoro-N-(4-oxo-4H-chromen-2-yl) benzamide (**6k**)

Yellow solid. Yield: 43%. Mp 249 °C. ^1^H NMR (300 MHz, DMSO-d_6_) δ 11.95 (s, 1H), 8.03 (d, J = 7.8 Hz, 1H), 7.88–7.74 (m, 2H), 7.63 (dd, J = 9.0, 2.5 Hz, 1H), 7.51 (dd, J = 13.4, 7.5 Hz, 2H), 7.39 (td, J = 8.5, 2.5 Hz, 1H), 6.92 (s, 1H). ^13^C NMR (75 MHz, DMSO-d_6_) δ 177.6, 165.1, 163.2 (d, J = 251.4 Hz), 157.0, 154.0, 134.5, 132.3 (d, J = 3.8 Hz), 132.1 (d, J = 11.0 Hz), 131.8 (d, J = 9.4 Hz), 126.0, 125.4, 123.4, 118.0 (d, J = 11.0 Hz), 117.6, 115.0 (d, J = 21.5 Hz), 97.1. *m/z* calcd. for C_16_H_9_ClFNO_3_ [M + H]^+^ 318.0333. Found: 318.0334.

3-Chloro-4-fluoro-N-(4-oxo-4H-chromen-2-yl) benzamide (**6l**)

Pale yellow solid. Yield: 56%. Mp 224 °C. ^1^H NMR (300 MHz, DMSO-d_6_) δ 11.68 (s, 1H), 8.28 (dd, J = 7.1, 2.3 Hz, 1H), 8.04 (ddd, J = 8.1, 4.8, 2.1 Hz, 2H), 7.87–7.75 (m, 1H), 7.61 (q, J = 8.5, 8.1 Hz, 2H), 7.51 (t, J = 7.5 Hz, 1H), 6.95 (s, 1H). ^13^C NMR (75 MHz, DMSO-d_6_) δ 177.63, 164.33, 160.17 (d, J = 253.0 Hz), 157.74, 154.16, 134.49, 131.46, 131.38 (d, J = 3.4 Hz), 130.28 (d, J = 8.6 Hz), 125.94, 125.36, 123.40, 120.28 (d, J = 18.1 Hz), 118.03, 117.63 (d, J = 21.6 Hz), 97.56. *m/z* calcd. For C_16_H_9_ClFNO_3_ [M + H]^+^ 318.0333. Found: 318.0331.

4-Nitro-N-(4-oxo-4H-chromen-2-yl) benzamide (**6m**)

Light yellow solid. Yield: 78%. Mp 323 °C. ^1^H NMR (300 MHz, DMSO-d_6_) δ 11.96 (s, 1H), 8.44–8.34 (m, 1H), 8.28–8.19 (m, 2H), 8.04 (dd, J = 7.8, 1.6 Hz, 2H), 7.82 (ddd, J = 8.7, 7.2, 1.7 Hz, 1H), 7.60 (dd, J = 8.6, 1.0 Hz, 1H), 7.51 (ddd, J = 8.1, 7.2, 1.1 Hz, 1H), 6.98 (s, 1H). ^13^C NMR (75 MHz, DMSO-d_6_) δ 177.7, 165.2, 157.7, 154.2, 150.1, 139.4, 134.5, 130.3 (2C), 126.0, 125.4, 124.0 (2C), 123.4, 118.1, 97.8. *m/z* calcd. for C_16_H_10_N_2_O_5_ [M + H]^+^ 311.0662. Found: 311.0677.

2,4-Dinitro-N-(4-oxo-4H-chromen-2-yl) benzamide (**6n**)

Brown solid. Yield: 25%. Mp 252 °C. ^1^H NMR (300 MHz, DMSO-d_6_) δ 12.32 (s, 1H), 8.89 (d, J = 2.2 Hz, 1H), 8.74 (dd, J = 8.4, 2.2 Hz, 1H), 8.17 (d, J = 8.4 Hz, 1H), 8.04 (dd, J = 7.9, 1.7 Hz, 1H), 7.81 (ddd, J = 8.7, 7.1, 1.7 Hz, 1H), 7.60–7.47 (m, 2H), 6.90 (s, 1H). ^13^C NMR (75 MHz, DMSO-d_6_) δ 177.5, 163.8, 156.6, 154.0, 148.9, 146.5, 136.2, 134.6, 131.8, 129.4, 126.1, 125.4, 123.4, 120.3, 118.0, 97.3. *m/z* calcd. for C_16_H_9_N_3_O_7_ [M − H]^+^ 354.0362. Found: 354.0363.

3,5-Dinitro-N-(4-oxo-4H-chromen-2-yl) benzamide (**6o**) 

Brown solid. Yield: 75%. Mp 291 °C. ^1^H NMR (300 MHz, DMSO-d_6_) δ 12.21 (s, 1H), 9.18 (d, J = 2.1 Hz, 2H), 9.09 (t, J = 2.1 Hz, 1H), 8.04 (dd, J = 7.9, 1.5 Hz, 1H), 7.82 (m, 1H), 7.62 (dd, J = 8.4, 0.6 Hz, 1H), 7.52 (bt, J = 0.6 Hz, 1H), 7.00 (s, 1H). ^13^C NMR (75 MHz, DMSO-d_6_) δ 177.6, 165.3, 162.9, 154.2, 148.5 (2C), 136.8, 134.6, 129.2 (2C), 126.0, 125.4, 123.4, 122.3, 118.5, 98.0. *m/z* calcd. for C_16_H_9_N_3_O_7_ [M + H]^+^ 356.0513. Found: 356.0520. Anal. Calcd.: C, 54.09; H, 2.55; N, 11.83%. Found: C, 53.84; H, 2.11; N,11.12%.

4-Methoxy-N-(4-oxo-4H-chromen-2-yl) benzamide (**6p**)

Dark orange solid. Yield: 89%. Mp 227 °C. ^1^H NMR (300 MHz, DMSO-d_6_) δ 11.35 (s, 1H), 8.08–7.99 (m, 3H), 7.80 (ddd, J = 8.7, 7.2, 1.7 Hz, 1H), 7.58 (dd, J = 8.5, 1.0 Hz, 1H), 7.50 (ddd, J = 8.0, 7.2, 1.1 Hz, 1H), 7.14–7.05 (m, 2H), 6.97 (s, 1H), 3.87 (s, 3H). ^13^C NMR (75 MHz, DMSO-d_6_) δ 177.6, 165.5, 163.3, 157.9, 154.2, 134.4, 131.0 (2C), 125.8, 125.5, 125.3, 123.5, 118.0, 114.3 (2C), 97.3, 56.0. *m/z* calcd. for C_17_H_13_NO_4_ [M + H]^+^ 296.0917. Found: 296.0915.

2-Methoxy-N-(4-oxo-4H-chromen-2-yl) benzamide (**6q**)

Light yellow solid. Yield: 80%. Mp 260 °C. ^1^H NMR (300 MHz, DMSO-d_6_) δ 11.20 (s, 1H), 8.03 (dd, J = 7.9, 1.7 Hz, 1H), 7.79 (ddd, J = 8.7, 7.2, 1.8 Hz, 1H), 7.68 (dd, J = 7.6, 1.8 Hz, 1H), 7.63–7.44 (m, 3H), 7.23 (dd, J = 8.5, 0.9 Hz, 1H), 7.11 (td, J = 7.5, 1.0 Hz, 1H), 6.95 (s, 1H), 3.94 (s, 3H). ^13^C NMR (75 MHz, DMSO-d_6_) δ 177.6, 165.4, 157.3, 157.0, 154.0, 134.4, 133.8, 130.4, 125.9, 125.3, 123.5, 123.4, 121.1, 118.1, 112.7, 96.8, 56.6. *m/z* calcd. for C_17_H_13_NO_4_ [M + H]^+^ 296.0917. Found: 296.0916.

2-(Methylthio)-N-(4-oxo-4H-chromen-2-yl) benzamide (**6r**)

Pale yellow solid. Yield: 63%. Mp 218 °C. ^1^H NMR (300 MHz, DMSO-d_6_) δ (ppm) 11.71 (s, 1H), 8.04 (d, J = 7.8 Hz, 1H), 7.79 (t, J = 7.8 Hz, 1H), 7.65 (d, J = 7.6 Hz, 1H), 7.52 (m, 4H), 7.30 (t, J = 7.4 Hz, 1H), 6.91 (s, 1H), 2.48 (s, 3H). ^13^C NMR (75 MHz, DMSO-d_6_) δ 177.5, 167.0, 157.4, 154.1, 138.7, 134.4, 134.0, 131.9, 128.9, 126.7, 125.9, 125.4, 124.9, 123.4, 118.0, 97.0, 15.9. *m/z* calcd. for C_17_H_13_NO_3_S [M + H]^+^ 312.0689. Found 312.0693.

3,4-Dimethoxy-N-(4-oxo-4H-chromen-2-yl) benzamide (**6s**)

Pale yellow solid. Yield: 70%. Mp. 246 °C. ^1^H NMR (300 MHz, DMSO-d_6_) δ 11.35 (s, 1H), 8.11–8.02 (m, 1H), 7.80 (ddd, J = 8.7, 7.2, 1.8 Hz, 1H), 7.72 (dd, J = 8.4, 2.1 Hz, 1H), 7.67–7.56 (m, 2H), 7.52–7.44 (m, 1H), 7.12 (d, J = 8.5 Hz, 1H), 6.99 (s, 1H), 3.89 (s, 3H), 3.87 (s, 3H). ^13^C NMR (75 MHz, DMSO-d_6_) δ 177.6, 165.5, 157.9, 154.2, 153.1, 148.8, 134.3, 125.8, 125.3 (2C), 123.5, 122.8, 118.0, 111.8, 111.4, 97.3, 56.2, 56.2. *m/z* calcd. for C_18_H_15_NO_5_ [M + H]^+^ 326.1023. Found: 326.1029.

3,5-Dimethoxy-N-(4-oxo-4H-chromen-2-yl) benzamide (**6t**)

Pale Yellow solid. Yield: 88%. Mp 198 °C. ^1^H NMR (300 MHz, DMSO-d_6_) δ 11.47 (s, 1H), 8.03 (dd, J = 7.8, 1.7 Hz, 1H), 7.80 (ddd, J = 8.7, 7.2, 1.8 Hz, 1H), 7.58 (dd, J = 8.5, 1.0 Hz, 1H), 7.49 (ddd, J = 8.1, 7.3, 1.1 Hz, 1H), 7.18 (d, J = 2.3 Hz, 2H), 6.97 (s, 1H), 6.76 (t, J = 2.3 Hz, 1H), 3.84 (s, 6H). ^13^C NMR (75 MHz, DMSO-d_6_) δ 177.6, 165.8, 160.8 (2C), 157.6, 154.2, 135.4, 134.4, 125.9, 125.4, 123.4, 118.0, 106.5 (2C), 105.4, 97.6, 56.1 (2C). *m/z* calcd. for C_18_H_15_NO_5_ [M + H]^+^ 326.1023. Found: 326.1022.

4-Morpholino-N-(4-oxo-4H-chromen-2-yl) benzamide (**6u**)

Brown solid. Yield: 92%. Mp 287 °C. ^1^H NMR (300 MHz, DMSO-d_6_) δ 11.16 (s, 1H), 8.07–7.91 (m, 3H), 7.80 (ddd, J = 8.6, 7.2, 1.7 Hz, 1H), 7.58 (dd, J = 8.5, 1.1 Hz, 1H), 7.50 (ddd, J = 8.0, 7.2, 1.1 Hz, 1H), 7.10–7.00 (m, 2H), 6.97 (s, 1H), 3.76 (t, J = 4.8 Hz, 4H), 3.32 (t, J = 5.0 Hz, 4H). ^13^C NMR (75 MHz, DMSO-d_6_) δ 177.5, 165.4, 158.1, 154.4, 154.2, 134.3, 130.5 (2C), 125.8, 125.3, 123.5, 122.1, 118.0, 113.5 (2C), 97.0, 66.3 (2C), 47.3 (2C). *m/z* calcd. for C_20_H_18_N_2_O_4_ [M + H]^+^ 351.1339. Found 351.1340.

N-(4-Oxo-4H-chromen-2-yl) pyrimidine-2-carboxamide (**6v**)

Orange solid. Yield: 56%. Mp 215 °C. ^1^H NMR (300 MHz, DMSO-d_6_) δ 11.50 (s, 1H), 9.10 (s, 1H), 9.09 (s, 1H), 8.04 (ddd, J = 7.9, 1.8, 0.5 Hz, 1H), 7.86–7.78 (m, 2H), 7.60 (ddd, J = 8.4, 1.1, 0.5 Hz, 1H), 7.51 (ddd, J = 8.1, 7.2, 1.1 Hz, 1H), 6.95 (s, 1H). ^13^C NMR (75 MHz, DMSO-d_6_) δ 177.6, 161.7, 158.56 (2C), 157.3, 156.7, 154.2, 134.5, 126.0, 125.3, 124.4, 123.4, 118.2, 97.7. *m/z* calcd. for C_14_H_9_N_3_O_3_ [M + H]^+^ 268.0717. Found: 268.0716.

N-(4-Oxo-4H-chromen-2-yl) butyramide (**6w**)

White cristals. Yield: 99%. Mp 212 °C. ^1^H NMR (300 MHz, DMSO-d_6_) δ 11.22 (s, 1H), 8.00 (dd, J = 7.9, 1.7 Hz, 1H), 7.78 (ddd, J = 8.7, 7.2, 1.8 Hz, 1H), 7.57–7.42 (m, 2H), 6.83 (s, 1H), 2.43 (t, J = 7.3 Hz, 2H), 1.62 (h, J = 7.3 Hz, 2H), 0.92 (t, J = 7.4 Hz, 3H). ^13^C NMR (75 MHz, DMSO-d_6_) δ 177.5, 172.6, 157.2, 154.0, 134.3, 125.8, 125.3, 123.4, 117.9, 96.0, 38.7, 18.4, 13.9. *m/z* calcd. for C_13_H_13_NO_3_ [M + H]^+^ 232.0974. Found: 232.0969.

N-(4-Oxo-4H-chromen-2-yl)cyclohexanecarboxamide (**6x**)

Orange solid. Yield: 80%. Mp 241 °C. ^1^H NMR (300 MHz, DMSO-d_6_) δ (ppm) 11.15 (s, 1H), 7.99 (dd, J = 7.9, 1.7 Hz, 1H), 7.77 (ddd, J = 8.8, 7.2, 1.8 Hz, 1H), 7.53 (dd, J = 8.6, 1.0 Hz, 1H), 7.47 (ddd, J = 8.1, 7.2, 1.1 Hz, 1H), 6.82 (s, 1H), 2.46 (m, 1H), 1.90–1.59 (m, 5H), 1.47–1.14 (m, 5H). ^13^C NMR (75 MHz, DMSO-d_6_) δ 177.4, 175.6, 157.4, 153.9, 134.3, 125.8, 125.3, 123.4, 117.9, 96.1, 45.1, 29.2 (2C), 25.7, 25.5 (2C). *m/z* calcd. for C_16_H_17_NO_3_ [M + H]^+^ 272.1281. Found: 272.1275.

(3R,5R,7R)-N-(4-Oxo-4H-chromen-2-yl) adamantane-1-carboxamide (**6y**)

Light yellow solid. Yield: 90%. Mp 286 °C. ^1^H NMR (300 MHz, DMSO-d_6_) δ 10.51 (s, 1H), 8.00 (dd, J = 7.9, 1.7 Hz, 1H), 7.79 (ddd, J = 8.7, 7.2, 1.8 Hz, 1H), 7.55 (dd, J = 8.5, 1.0 Hz, 1H), 7.51–7.45 (m, 1H), 6.83 (s, 1H), 2.08–2.00 (m, 3H), 1.95 (d, J = 2.9 Hz, 6H), 1.72 (d, J = 3.1 Hz, 6H). ^13^C NMR (75 MHz, DMSO-d_6_) δ 177.5, 177.0, 157.8, 154.1, 134.3, 125.8, 125.3, 123.4, 117.9, 97.0, 42.4, 37.8 (3C), 36.2 (3C), 28.0 (3C). *m/z* calcd. for C_20_H_21_NO_3_ [M + H]^+^ 324.1594. Found: 324.1587.

### 3.3. Bacterial Strains and Growth Conditions

*Pseudomonas aeruginosa* PAO1 was obtained from the Institute Pasteur Collection (CIP 104116, Paris, France), frozen and kept at -80 °C in a 20% (*v*/*v*) glycerol stock solution. Before each experiment, two successive subcultures were prepared on trypticase soy agar and incubated for 24 h under aerobic conditions at 37 °C. Before each experiment, bacterial suspensions of 10^8^ CFU/mL were prepared by adjusting OD at 640 nm to 0.150.

The bacterial enumerations were done on trypticase soy agar. The minimum biofilm broth used for biofilm formation (MBB) was prepared in sterile distilled water (SDW) and was composed of MgSO_4_·7H_2_O (0.02 g/L), FeSO_4_·7H_2_O (0.5 mg/L), Na_2_HPO_4_ (1.25 g/L), KH_2_PO_4_ (0.5 g/L), (NH_4_)_2_SO_4_ (0.1 g/L), and glucose (0.05 g/L) from Merck-Sigma (France). This minimal medium has been previously demonstrated to favor *P. aeruginosa* biofilm formation rather than the growth of planktonic cells [23].

### 3.4. Anti-Biofilm Assay

The enumeration of adhered bacteria was carried-out according to Campanac et al. [59] with some modifications. *P. aeruginosa* biofilms were grown in a 24-wells microtiter plate for 48 h. The culture medium was composed of BBM 2X (1.0 mL) and 1.0 mL of a 10^−4^ M solution of tested compound dissolved in SDW (final concentration 50 µM). After inoculation with 100 µL of a 10^2^ CFU/mL bacterial suspension of *P. aeruginosa* PAO1 (final inoculum 10 CFU/well), the microtiter plate was incubated à 37 °C. After 2 h, 4 h, 6 h, 20 h and 24 h of incubation, each well was emptied, rinsed twice with SDW, and the culture medium was renewed. After 48 h of culture, the biofilm was rinsed twice with 2.0 mL SDW. The bottom of each well was scraped with a sterile spatula and suspended in 1.0 mL of SDW. The content of each well was diluted by serial dilutions in test tubes. After inclusion of 0.9 mL of each dilution in trypticase soy agar, Petri dishes were incubated for 48 h at 37 °C. The number of colony forming units (CFUs) was counted and the inhibition percentage calculated using the following formula:

$$\mathrm{Inhibition}\left(\%\right)=\frac{{\mathrm{Adhered}\;\mathrm{cells}}_{\mathrm{Control}}\left(\mathrm{CFU}/\mathrm{mL}\right)-{\mathrm{Adhered}\;\mathrm{cells}}_{\mathrm{Sample}}\left(\mathrm{CFU}/\mathrm{mL}\right)\;}{{\mathrm{Adhered}\;\mathrm{cells}}_{\mathrm{Control}}}\times 100$$

Determination of the MICs and MBCs of evaluated compounds was performed in Mueller-Hinton broth and Mueller-Hinton agar, respectively, according to EUCAST/CA-SFM guidelines (2020). 

### 3.5. Cytotoxic Activity

Evaluation of compounds’ cytotoxicity was performed using the MTT (3-(4,5-dimethylthiazol-2-yl)-2,5-diphenyltetrazolium bromide) colorimetric assay, based on the Mosmann et al. procedure [55] with some modifications. The potential cytotoxic activity of the ten most active compounds was assessed against Vero cells (ATCC^®^ CCL-81) purchased from ATCC^®^ (Manassas, VA, USA) and cultivated in RPMI medium (RPMI 1640–PAN-Biotech). Stock solutions of each compound prepared in Dulbeccos’s phosphate-buffered saline (D-PBS, Sigma, St. Quentin Fallavier, France) at a concentration of 2 mM were subjected to 2-fold serial dilutions with RPMI medium.

96-wells microtiter plates were first filled with 100 µL of a cell suspension prepared in RPMI medium (2 × 10^4^ cells/100 µL). After overnight incubation at 37 °C in a humidified 5–6.5% CO_2_ incubator, 100 µL of each product dilution were added to achieve final concentrations ranging from 1.0 mM to 3.9 µM (final volume 200 µL/well). Wells corresponding to the untreated control were supplemented with 100 µL of fresh medium. Tween 40 (5%, Sigma, St. Quentin Fallavier, France) was used as positive control for cytotoxicity. A third control was performed with a non-inoculated culture medium and compounds solution to validate the absence of interactions (OD measurements) between the assessed molecule and the reagents used in the procedure. Each concentration, for each compound, was tested in quadricates. The microplate was then incubated for 24 h at 37 °C in a humidified 5–6.5% CO_2_ incubator. The supernatant was then discarded followed by rinsing with 100 µL of D-PBS. Then 100 µL of an MTT solution, prepared in D-PBS at a concentration of 0.5 mg/mL, were added in all wells. After 60 min of incubation at 37 °C and, in order to solubilize the formazan that formed, an indicator of cell viability, 100 µL of DMSO, were added. After agitation, the OD was measured at 570 nm using a CLARIOstar Plus plate reader (BMG Labtech) and the viability percentage was calculated using the following formula. Assays were performed in duplicate with four technical replicates.

$$\mathrm{Viability}\;\left(\%\right)=\frac{{\mathrm{OD}}_{570\mathrm{nm}}\;\mathrm{of}\;\mathrm{treated}\;\mathrm{cells}}{{\mathrm{OD}}_{570\mathrm{nm}}\;\mathrm{of}\;\mathrm{control}\;\mathrm{untreated}\;\mathrm{cells}\;}\times 100$$

## 4. Conclusions

In the present study, we employed molecular docking to assess the potential of two series of chromones-based PQS analogs as PqsR ligands. Most compounds showed good predicted affinities for PqsR, validating their synthesis for biological evaluation in a whole-cell biofilm assay. The synthesis has been optimized to give 34 direct and 25 retro chromones carboxamides in good yields. 

Although we did not observe a high correlation between docking scores and the anti-biofilm activity, results showed several chromone 2-carboxamides of the retro series are potential inhibitors of the formation of *P. aeruginosa* biofilms, without bactericidal or bacteriostatic effect on planktonic cells and no cytotoxicity on Vero cells (IC_50_ > 1.0 mM) for the most active inhibitors. The 2,4-dinitro-N-(4-oxo-4H-chromen-2-yl) benzamide was selected as hit compound for further optimization studies. These results are encouraging for future development of chromone carboxamide-based compounds as new antibiofilm agents and possible adjuvants in the treatment of CF-associated *P. aeruginosa* pulmonary infections.

## Figures and Tables

**Table 1:** Optimization of the coupling conditions for obtaining of chromone carboxamide **6**.

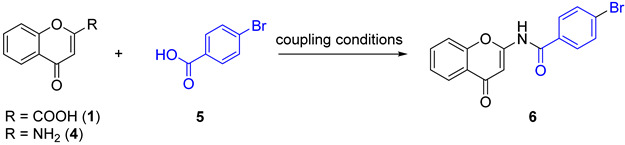 General route for the synthesis of **6** according to the coupling conditions given below			
**Entry**	**R**	**Coupling Conditions**	**Result Compound** **(Global Yield)**
1	NH_2_	POCl_3_, DIPEA, dry DMF, 18 h, r.t	**6** (traces)
2	NH_2_	(a) SOCl_2_, 0 to 100 °C (b) TEA, acetonitrile, r.t, 12 h	**6** (32%)
3	NH_2_	(a) CDI, dry THF, r.t to 60 °C, 2 h (b) Et_3_N, 60 °C, 12 h	**6** (25%)
4	NH_2_	(a) PyBOP, dry DMF, DIPEA, 0 °C, 45 min (b) r.t, overnight	**6** (23%)
5	NH_2_	(a) PCl_5_, dry cyclohexane, reflux, 2 h (b) Al powder, acetonitrile, ultrasound, 1 h	E.P.N.I
6	NH_2_	(a) SOCl_2_, dry toluene, reflux, 3 h (b) DMAC, 0 °C to r.t, 18 h	**6** (31%)
7	NH_2_	(a) SOCl_2_, dry toluene, reflux, 3 h (b) TEA, DMAC, 0 °C to r.t, 18 h	**6** (22%) + **7** (35%) 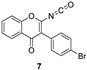
8	NH_2_	(a) CDI, dry DMF, r.t to 65 °C, 1 h (b) Phenol, DMAP, 12 h (c) Pd(PEPSI), K_2_CO_3_, DME, 110 °C, 18 h	**6** (70%)
9	COOH	(a) PCl_5_, dry cyclohexane, reflux, 2 h (b) NaN_3_, dry cyclohexane, 0 °C to r.t, 1.5 h (c) cyclohexane, reflux, 18h	**6** (90%)
10	COOH	(a) Ph_2_POCl, DBU, dry toluene, 0 °C, 5 min (b) NaN_3_, DMAP, dry DMF, 100 °C, 3 h (c) 100 °C, 12 h	**6** (traces)

All yields correspond to isolated products. E.P.N.I = expected product was not isolated.

**Table 2:** Docking scores (E_bind_, kcal·mol^−1^) and in vitro anti-biofilm activity data for the pairs of direct (**3a**–**y**) and retro (**6a**–**y**) chromone carboxamides. The anti-biofilm activity is expressed in % of inhibition as the mean ± SD of 3 independent experiments (n = 3 ∗ 2). NA: non-active (% inhibition < 10.0%).

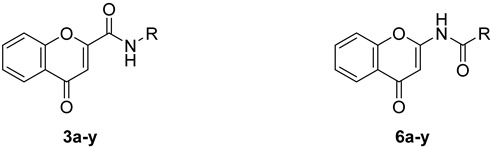						
**R**	**Cpd**	**E_bind_** **(kcal·mol^−1^)**	**Biofilm Inhibition (%)**	**Cpd**	**E_bind_** **(kcal·mol^−1^)**	**Biofilm Inhibition (%)**
PQS	**-**	−8.1	-			
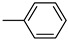	**3a**	−8.0	21.4 ± 17.0	**6a**	−9.1	73.6 ± 6.1
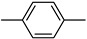	**3b**	−8.4	39.1 ± 5.4	**6b**	−8.8	67.0 ± 3.6
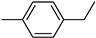	**3c**	−8.6	43.3 ± 7.2	**6c**	−8.8	63.1 ± 3.0
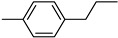	**3d**	−8.7	55.6 ± 18.3	**6d**	−9.2	69.9 ± 9.7
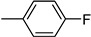	**3e**	−8.2	73.3 ± 11.0	**6e**	−8.5	65.3 ± 7.6
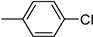	**3f**	−8.2	25.9 ± 4.3	**6f**	−8.6	66.6 ± 2.1
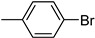	**3g**	−8.3	47.2 ± 2.8	**6g**	−8.4	56.2 ± 7.9
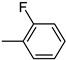	**3h**	−8.5	42.4 ± 10.4	**6h**	−8.5	27.8 ± 10.7
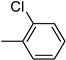	**3i**	−8.5	35.2 ± 14.8	**6i**	−9.2	59.3 ± 3.3
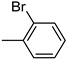	**3j**	−8.2	46.1 ± 10.2	**6j**	−9.0	36.1 ± 4.1
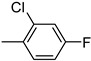	**3k**	−8.7	44.7 ± 9.4	**6k**	−8.8	63.2 ± 9.3
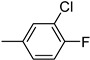	**3l**	−8.4	NA	**6l**	−8.9	67.2 ± 5.2
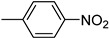	**3m**	−8.3	58.7 ± 2.5	**6m**	−8.4	NA
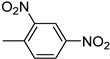	**3n**	−8.8	NA	**6n**	−8.4	90.0 ± 9.9
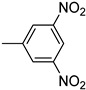	**3o**	−8.8	43.8 ± 24.2	**6o**	−9.1	78.3 ± 4.0
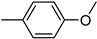	**3p**	−7.9	47.5 ± 5.3	**6p**	−8.4	39.3 ± 17.1
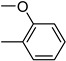	**3q**	−8.3	41.3 ± 5.7	**6q**	−8.3	35.2 ± 6.8
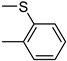	**3r**	−8.1	47.8 ± 5.7	**6r**	−8.2	60.3 ± 15.8
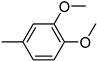	**3s**	−7.9	NA	**6s**	−8.4	71.9 ± 13.5
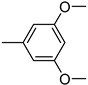	**3t**	−9.0	NA	**6t**	−8.5	43.8 ± 2.4
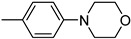	**3u**	−9.1	51.2 ± 11.7	**6u**	−9.1/−8.9	56.8 ± 10.0
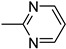	**3v**	−8.3	46.0 ± 7.3	**6v**	−8.1	48.8 ± 2.6
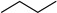	**3w**	−7.7	NA	**6w**	−7.8	56.4 ± 7.2
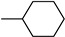	**3x**	−8.9	32.0 ± 10.3	**6x**	−8.3	48.7 ± 5.3
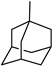	**3y**	−9.5	24.4 ± 8.8	**6y**	−9.4	19.5 ± 1.8

**Table 3:** Docking scores (E_bind_, kcal·mol^−1^) and in vitro anti-biofilm activity data for direct chromone carboxamides (**3′a–i**). The anti-biofilm activity is expressed in % of inhibition as the mean ± SD of 3 independent experiments (n = 3 ∗ 2). NA: non-active (% inhibition < 10.0%).

R	Cpd	E_bind_ (kcal·mol^−1^)	Biofilm Inhibition (%)
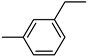	**3′a**	−9.2	70.5 ± 5.6
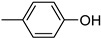	**3′b**	−8.4	35.5 ± 5.8
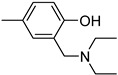	**3′c**	−8.8/−9.0	39.7 ± 4.5
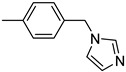	**3′d**	−9.5	NA
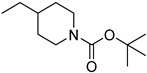	**3′e**	−9.3	36.0 ± 28.6
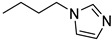	**3′f**	−8.2	NA
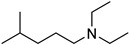	**3′g**	−7.5	14.7 ± 2.1
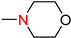	**3′h**	−8.0	55.3 ± 5.5
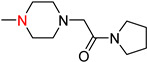	**3′i**	−9.3	10.8 ± 0.4

## Data Availability

Data is contained within the article and Supplementary Materials.

## Supplementary Materials

The following supporting information can be downloaded at https://www.mdpi.com/article/10.3390/ph15040417/s1, Table S1: Structural properties and Lipinski’s parameters of chromone carboxamides. Figure S1: Anti-biofilm activity against *P. aerugiosa* PAO1 of direct chromone carboxamides (**3a**–**y** and **3′**a–**i**) at 50 µM. Figures S2–S123: ^1^H and ^13^C NMR spectra of final compounds.
