# Supplementary material for: Investigation of Direct and Retro Chromone-2-Carboxamides Based Analogs of Pseudomonas aeruginosa Quorum Sensing Signal as New Anti-Biofilm Agents
Source: Pharmaceuticals (Basel). 2022 Mar 29;15(4):417. doi: 10.3390/ph15040417 (PMC9026348; doi:10.3390/ph15040417)
Supplement: Supplementary file 1 [file pharmaceuticals-15-00417-s001.zip › pharmaceuticals-1593335-supplementary.pdf]

Investigation of Direct and Retro Chromone-2-carboxamides based Analogs of *Pseudomonas aeruginosa* Quorum Sensing Signal as New Anti-biofilm Agents

Jeanne Trognon [1], Gonzalo Vera [1], Maya Rima [1], Jean-Luc Stigliani [2], Laurent Amielet [1], Salomé El Hage [1], Barbora Lajoie [1], Christine Roques [1] and Fatima El Garah [1,*]

Supplementary Material

Table S1: Structural properties and Lipinski's parameters of chromone carboxamides

Code	MW	LogS	LogP	pKa	non-H atomes	VdW surface area (Å²)
				**Series 1**		
**3a**	265.0	-4.6	2.73	12.11	20	338.8
**3b**	279.0	-5.1	3.24	12.19	21	370.1
**3c**	293.0	-5.6	3.69	12.2	22	400.4
**3d**	307.0	-6.1	4.13	12.19	23	431.1
**3e**	283.0	-4.9	2.87	12.18	21	345.8
**3f**	299.0	-5.3	3.34	12.09	21	355.2
**3g**	344.2	-5.6	3.50	12.09	21	359.6
**3h**	283.0	-4.9	2.87	10.9	21	345.6
**3i**	299.0	-5.3	3.34	11.41	21	354.8
**3j**	344.2	-5.6	3.50	11.55	21	358.7
**3k**	317.7	-5.6	3.48	11.51	22	361.8
**3m**	310.3	-5.3	2.67	11.58	23	376.9
**3o**	355.3	-5.9	2.61	11.32	26	413.3
**3p**	295.3	-4.6	2.57	12.24	22	386.4
**3q**	295.3	-4.6	2.57	11.27	22	385.5
**3r**	311.4	-5.3	3.36	11.67	22	392.4
**3u**	350.3	-4.9	2.62	15.21	26	472.3
**3v**	267.2	-3.9	1.49	0.69	20	324.1
**3x**	271.3	-4.4	2.51	13.97	20	386.6
**3y**	323.4	-5.6	2.81	13.56	24	447.0
**3′a**	293.0	-5.6	3.69	12.11	22	400.9
**3′b**	366.0	-4.2	2.43	9.01	21	350.2
**3′c**	281.0	-4.1	3.08	8.06	27	530.1
**3′e**	386.2	-4.4	2.1	14.03	28	579.2
**3′g**	330.0	-1.8	2.44	9.32	24	537.6
**3′i**	384.0	-3.4	0.31	5.03	28	532.9
				**Series 2**		
**6a**	265.3	-4.9	2.84	12.85	20	338.1
**6b**	279.3	-5.4	3.35	13.08	21	370.1
**6c**	293.3	-5.9	3.80	13.06	22	400.9
**6d**	307.3	-6.4	4.24	13.05	23	433.2
**6e**	283.3	-5.2	2.98	12.62	21	345.2
**6f**	300.4	-5.6	3.45	12.60	21	354.3
**6g**	344.2	-5.9	3.61	12.64	21	358.6
**6h**	283.2	-5.2	2.98	9.84	21	343.8
**6i**	300.4	-5.6	3.45	10.09	21	352.9
**6j**	344.2	-5.9	3.61	10.07	21	356.3
**6k**	317.7	-5.9	3.59	9.93	22	360.0

**6l**	317.7	-5.9	3.59	12.28	22	360.5
**6m**	310.3	-5.6	2.78	10.16	23	376.1
**6n**	357.3	-5.0	2.72	8.79	26	419.0
**6o**	357.3	-5.0	2.72	9.38	26	421.0
**6p**	295.3	-4.9	2.68	12.93	22	386.3
**6q**	296.3	-4.9	2.68	10.61	22	385.5
**6r**	312.4	-5.6	3.47	10.9	22	392.7
**6s**	327.3	-4.9	2.53	12.57	24	434.9
**6t**	325.3	-4.9	2.53	10.65	24	435.1
**6u**	350.4	-5.2	2.73	13.53	26	473.8
**6v**	267.2	-3.2	1.46	12.07	20	323.6
**6w**	231.3	-3.8	2.13	12.43	17	326.4
**6x**	271.3	-5.2	3.10	12.40	20	385.5
**6y**	323.4	-6.3	3.59	12.36	24	447.3

Figure S1. Anti-biofilm activity against *P. aeruginosa* PAO1 of direct chromone carboxamides (**3a-y** and **3′a-i**) at 50 μM, expressed as inhibition % (GraphPad 5.0)

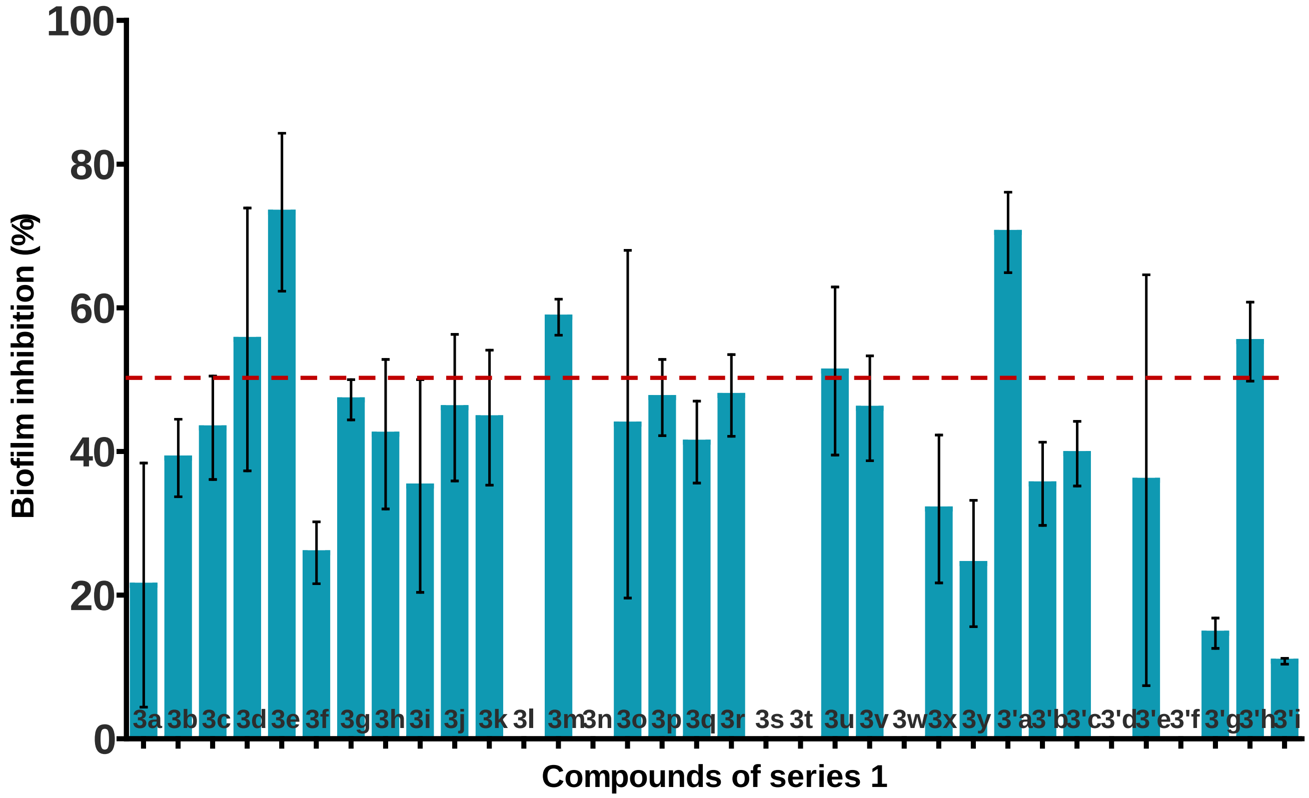

Figures S2 to S123: ^{1}H (300 MHz, DMSO-d$_6$) and ^{13}C NMR (75 MHz, DMSO-d$_6$) spectra of final compounds

4-Oxo-4*H*-chromene-2-carbonyl chloride (2)

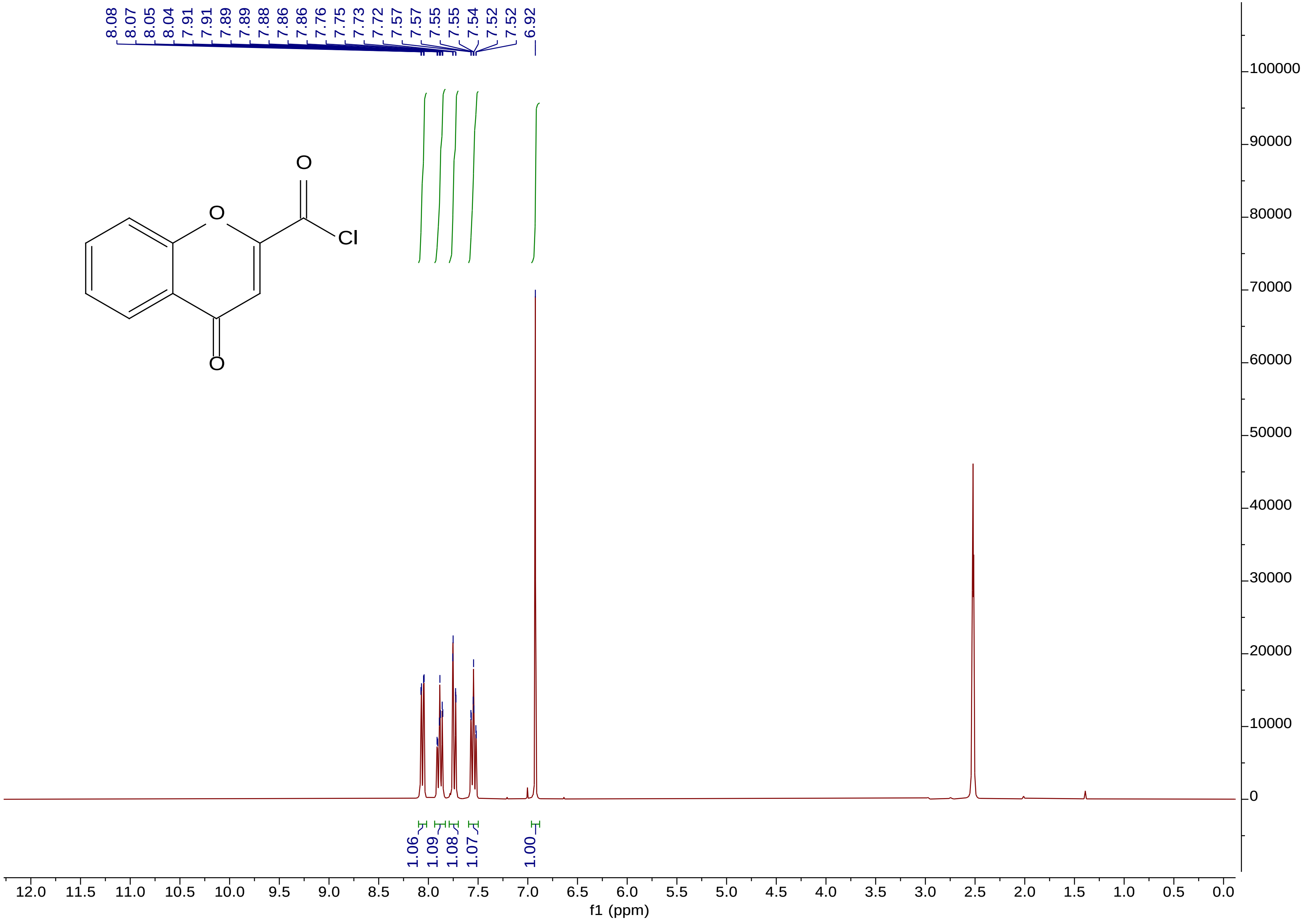

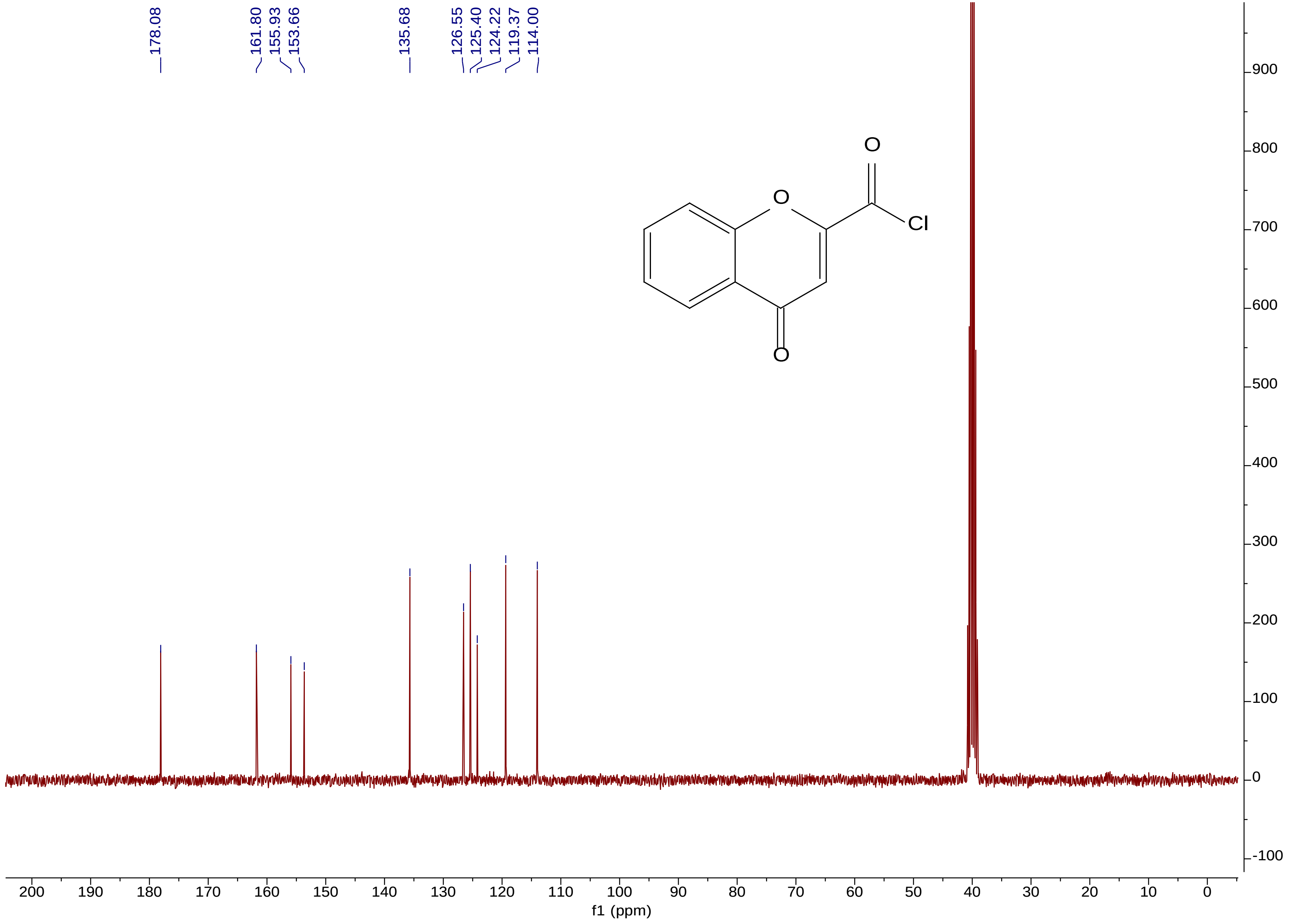

2-Amino-4*H*-chromen-4-one (4)

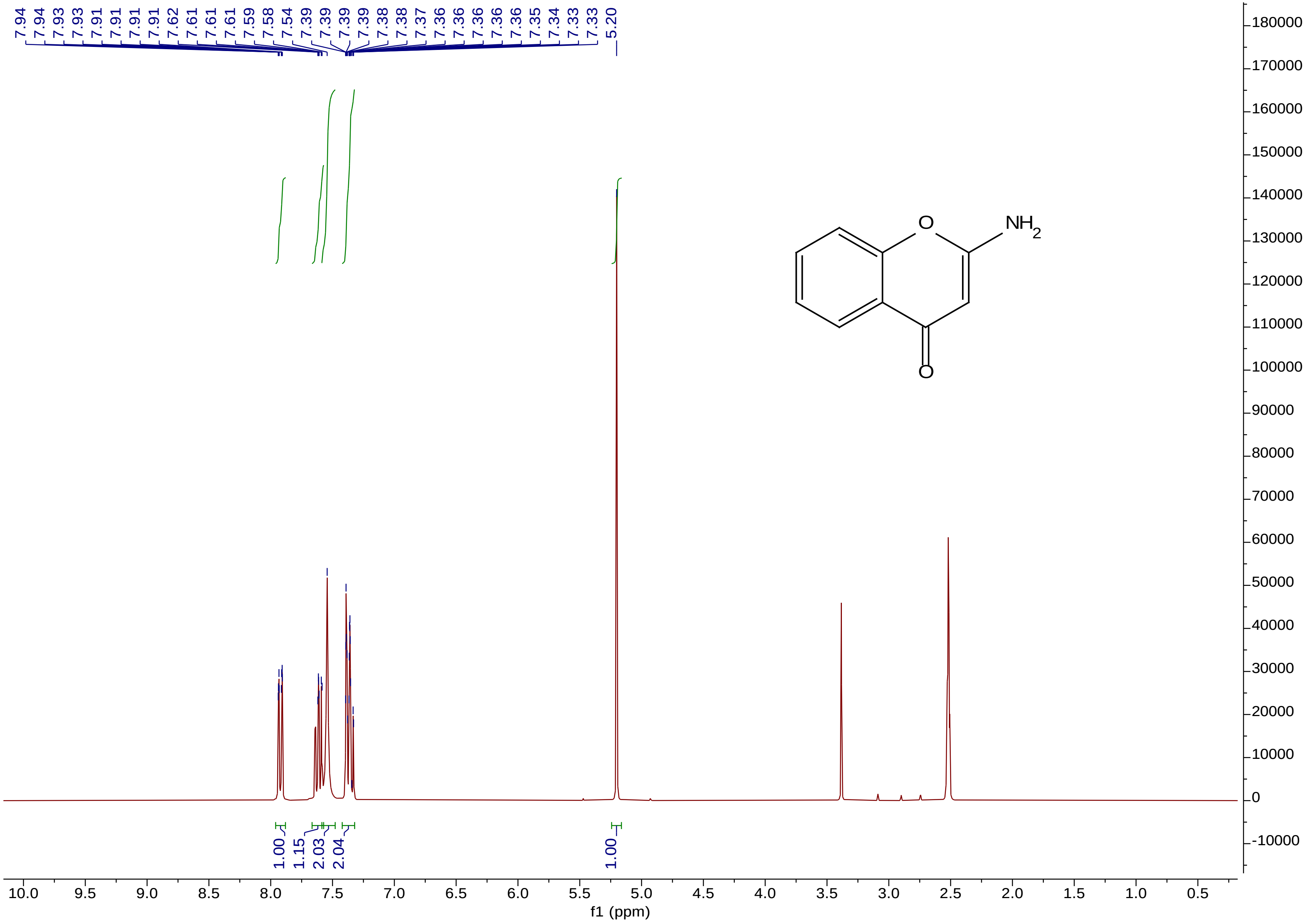
7.94 7.94 7.93 7.93 7.91 7.91 7.91 7.91 7.62 7.61 7.61 7.61 7.59 7.58 7.54 7.39 7.39 7.39 7.39 7.38 7.38 7.37 7.36 7.36 7.36 7.36 7.35 7.34 7.33 7.33
5.20
1.00
1.15
2.03
2.04
1.00
O
NH2
O
180000
170000
160000
150000
140000
130000
120000
110000
100000
90000
80000
70000
60000
50000
40000
30000
20000
10000
0
-10000
10.0 9.5 9.0 8.5 8.0 7.5 7.0 6.5 6.0 5.5 5.0 4.5 4.0 3.5 3.0 2.5 2.0 1.5 1.0 0.5
f1 (ppm)

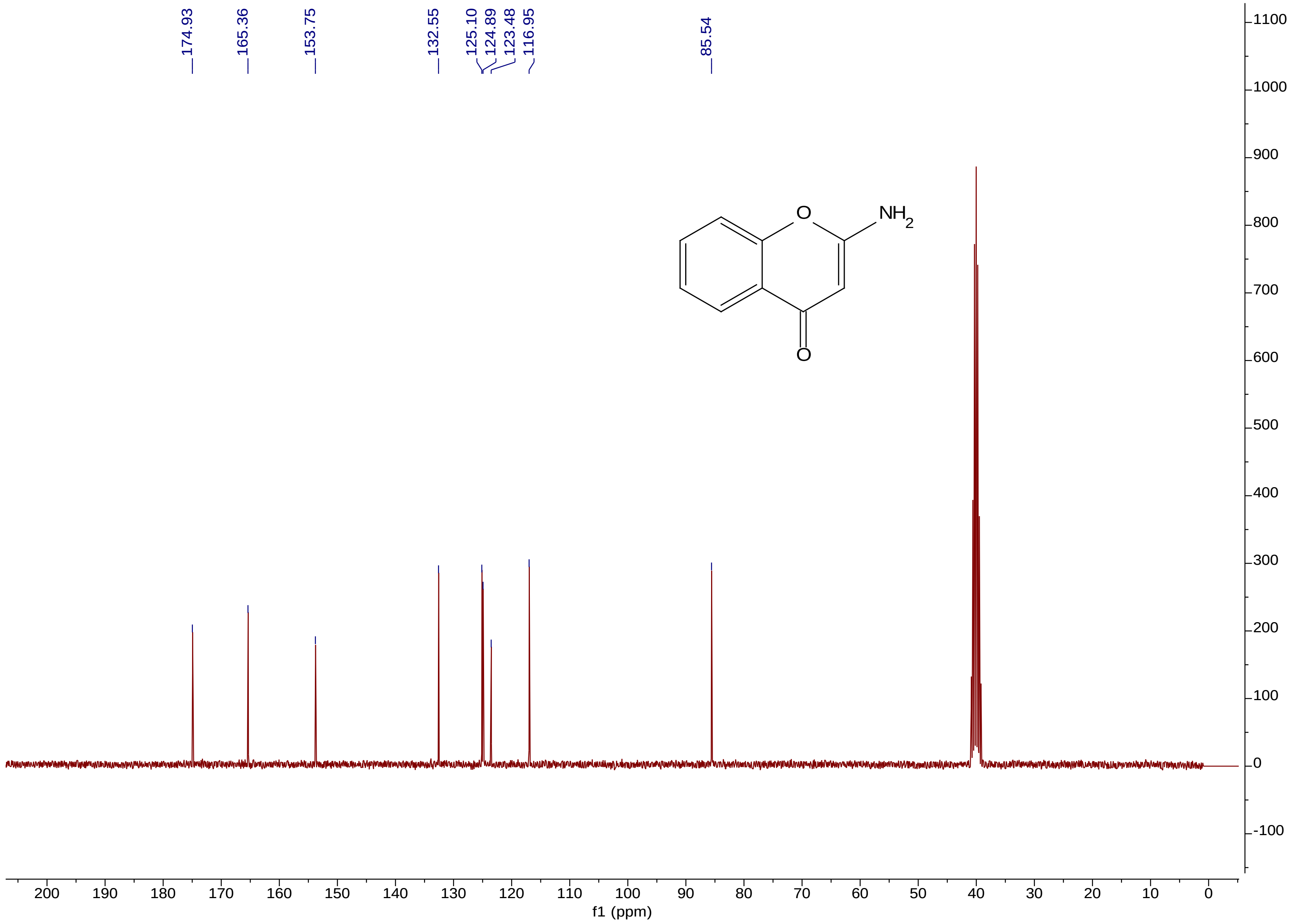

4-Oxo-*N*-phenyl-4*H*-chromene-2-carboxamide (3a)

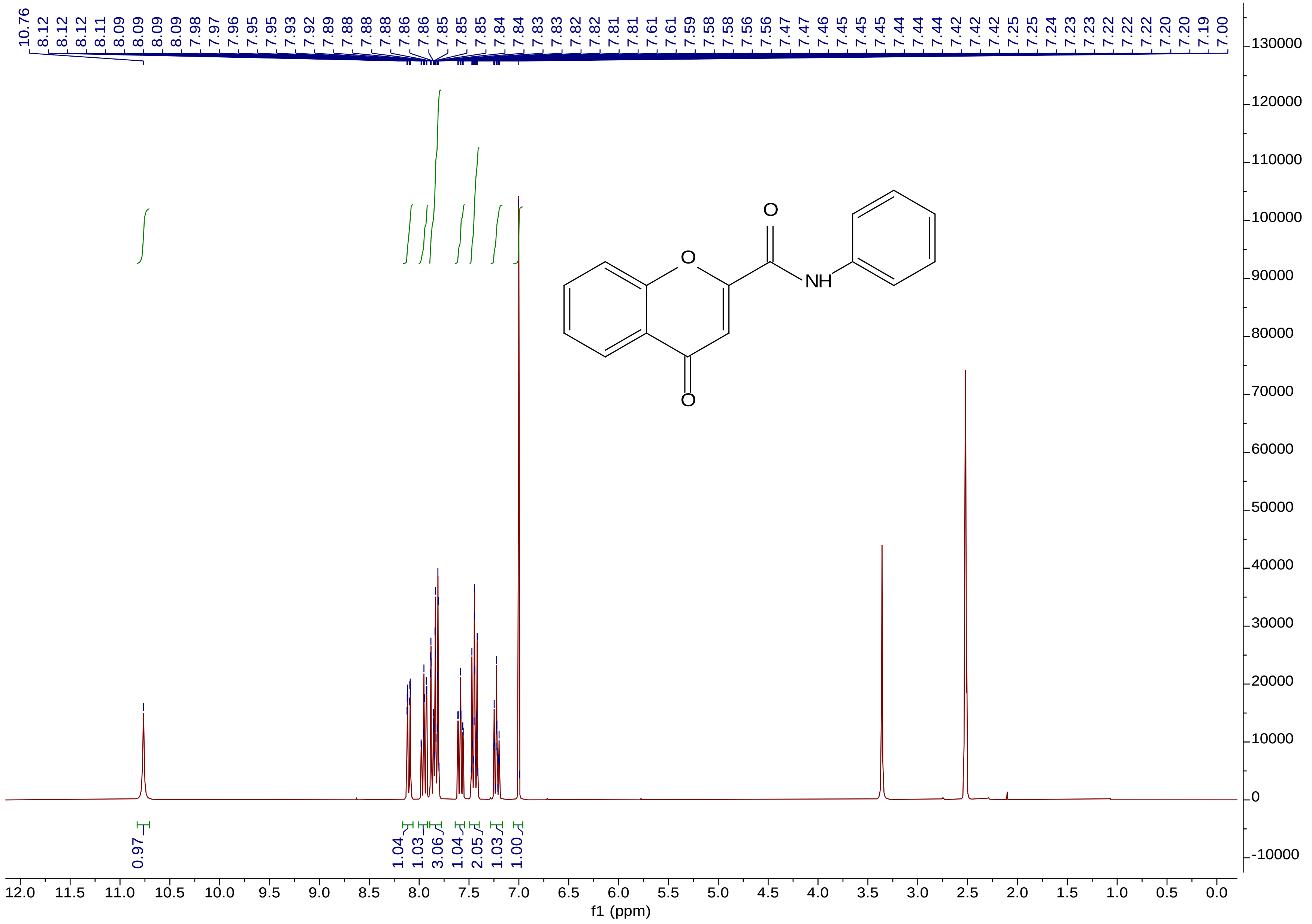

f1 (ppm)
0.97
1.04
1.03
3.06
1.04
2.05
1.03
1.00
O
NH

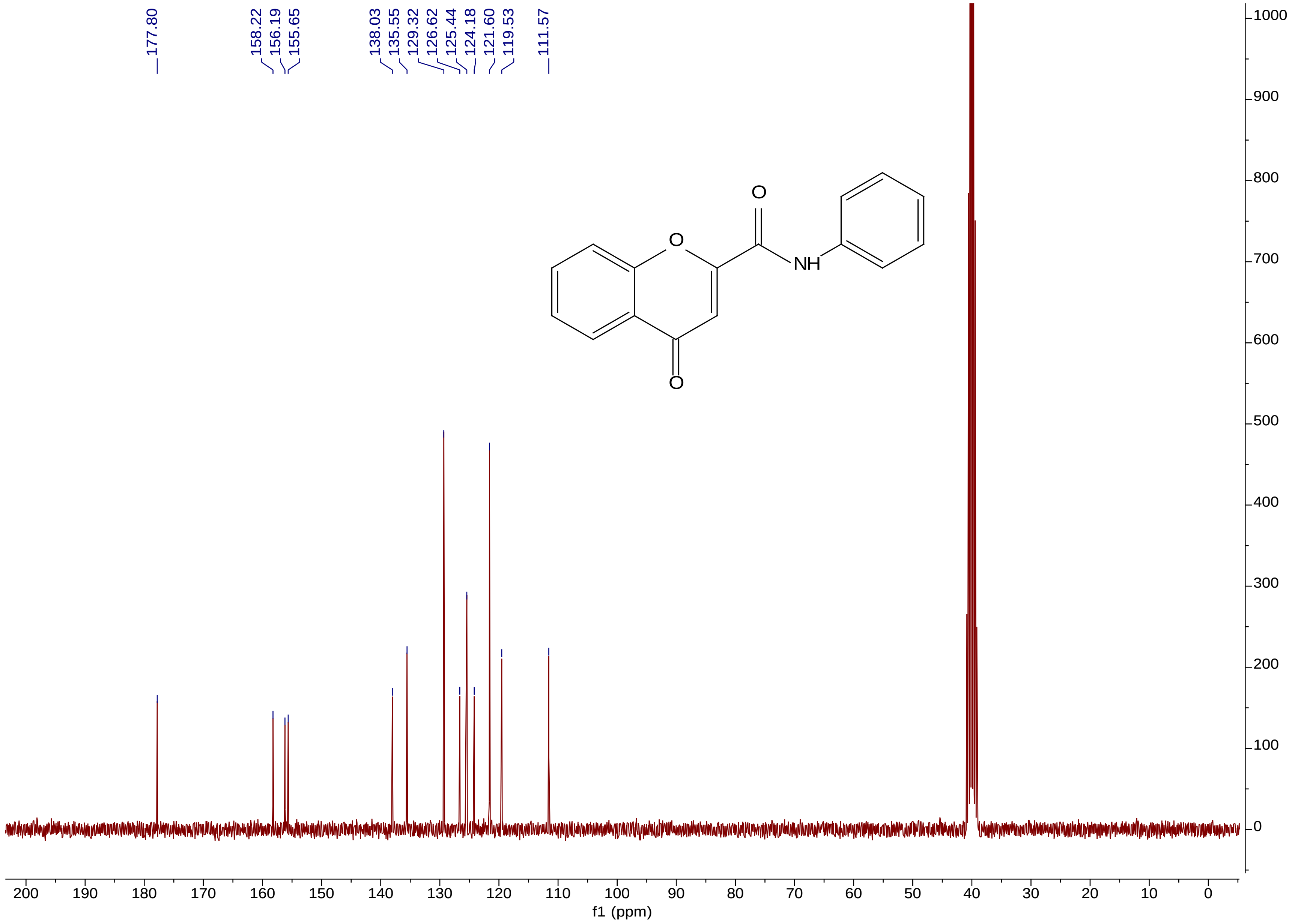
177.80
158.22
156.19
155.65
138.03
135.55
129.32
126.62
125.44
124.18
121.60
119.53
111.57
O
O
NH
O
1000
900
800
700
600
500
400
300
200
100
0
200 190 180 170 160 150 140 130 120 110 100 90 80 70 60 50 40 30 20 10 0
f1 (ppm)

4-Oxo-*N*-(p-tolyl)-4*H*-chromene-2-carboxamide (3b)

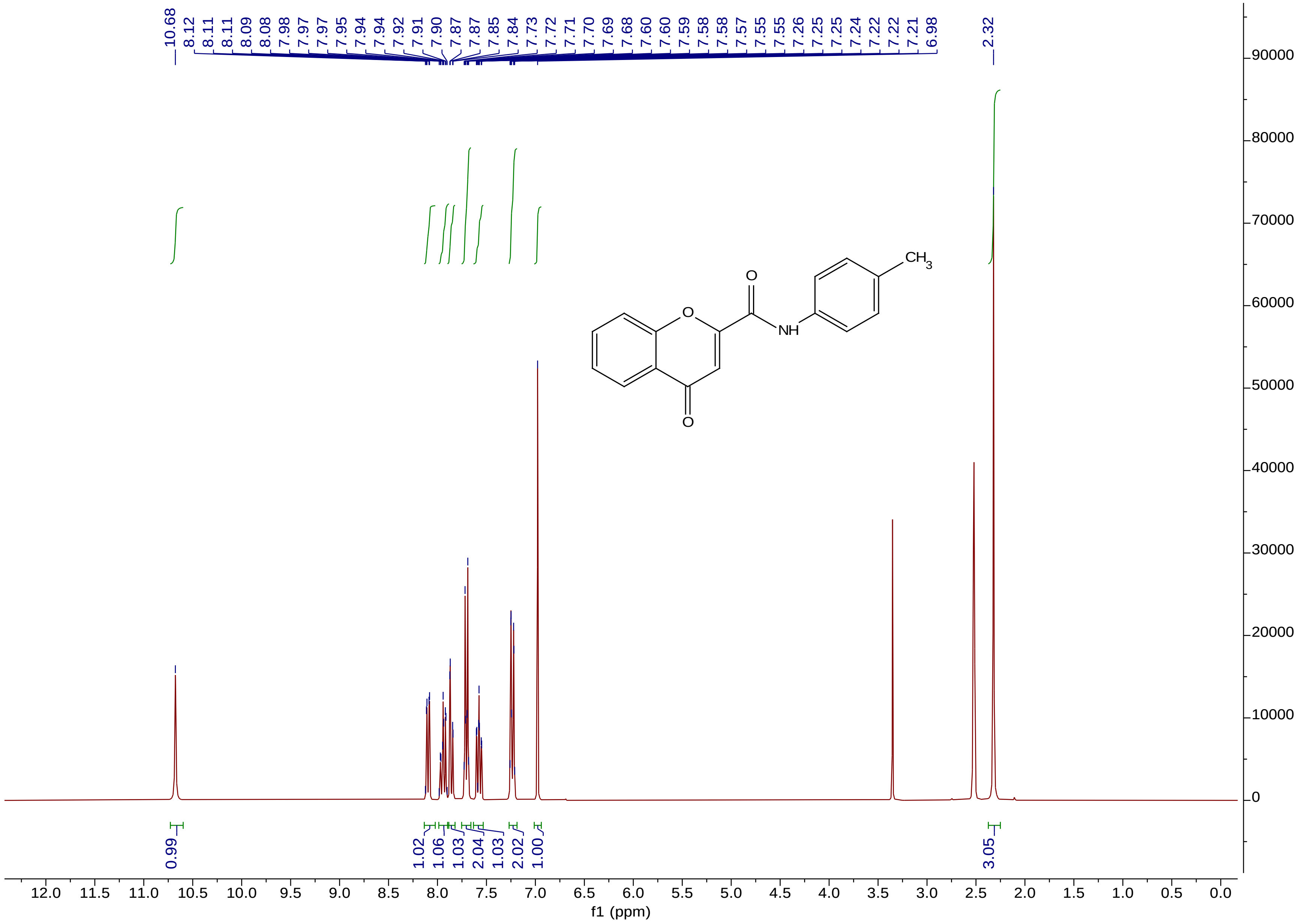

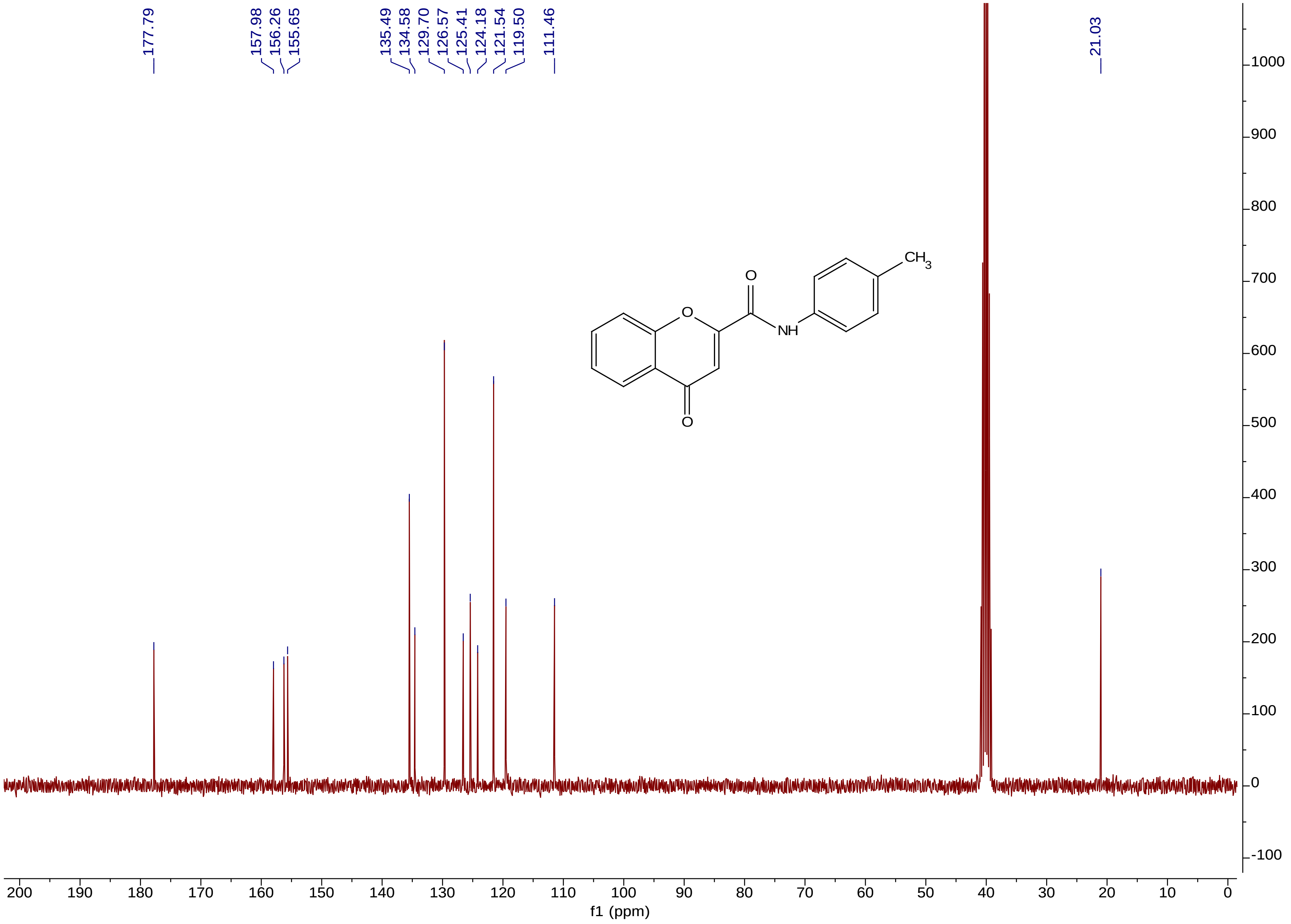

177.79
157.98
156.26
155.65
135.49
134.58
129.70
126.57
125.41
124.18
121.54
119.50
111.46
21.03
O
O
NH
O
CH3
1000
900
800
700
600
500
400
300
200
100
0
-100
200 190 180 170 160 150 140 130 120 110 100 90 80 70 60 50 40 30 20 10 0
f1 (ppm)

N-(4-Ethylphenyl)-4-oxo-4H-chromene-2-carboxamide (3c)

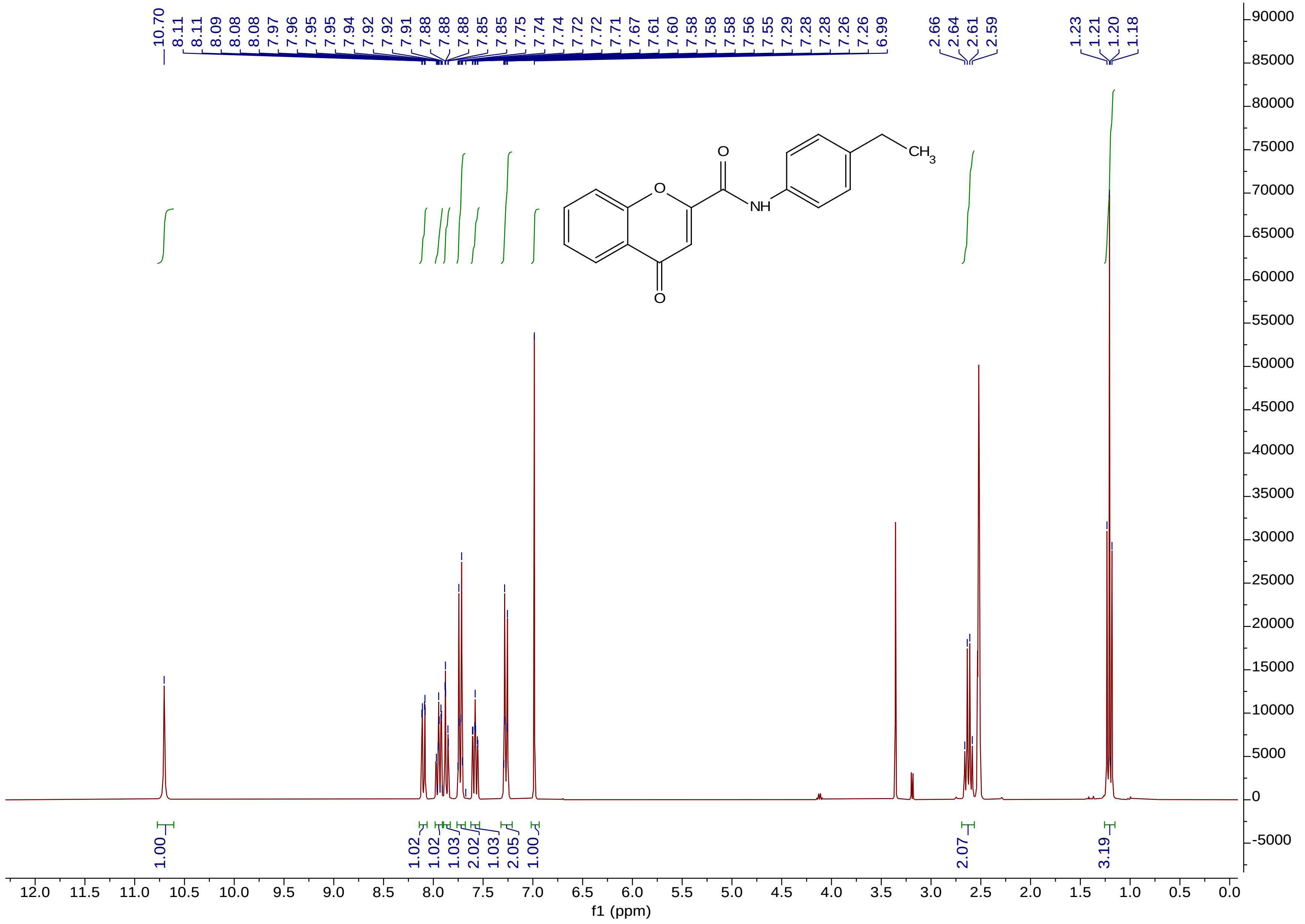

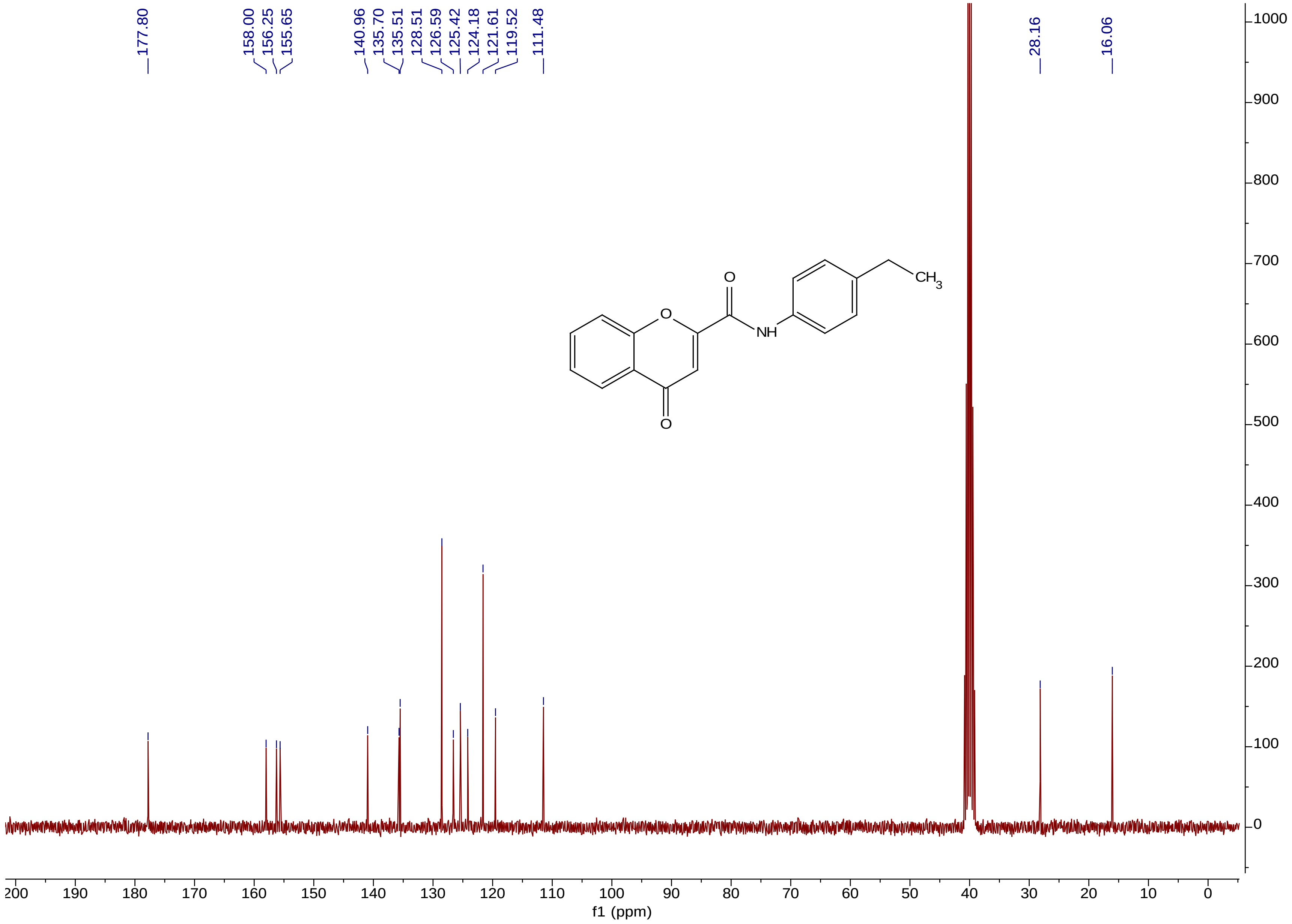
177.80
158.00
156.25
155.65
140.96
135.70
135.51
128.51
126.59
125.42
124.18
121.61
119.52
111.48
28.16
16.06
O
O
NH
CH3
O
f1 (ppm)

4-Oxo-*N*-(4-propylphenyl)-4*H*-chromene-2-carboxamide (3d)

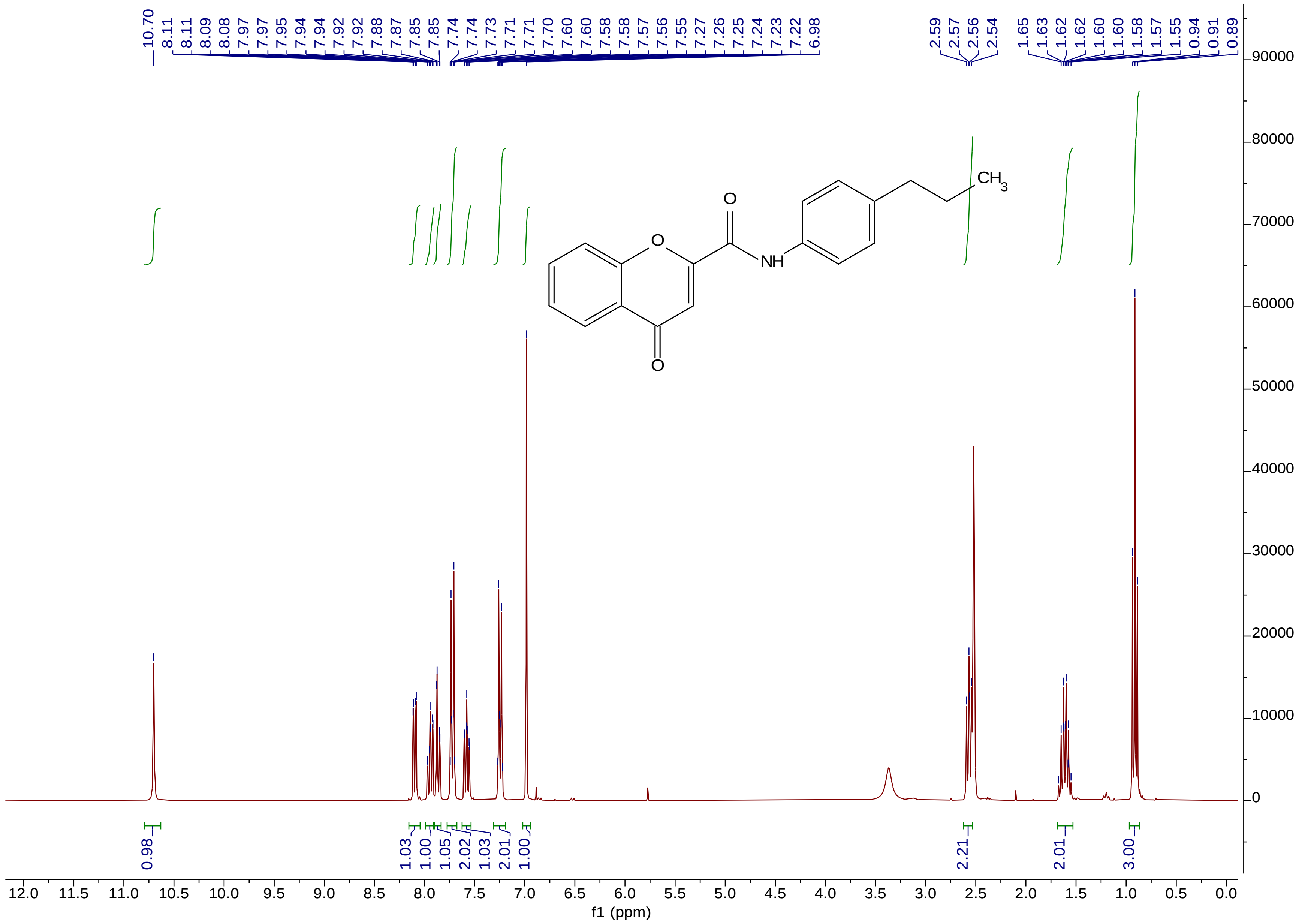

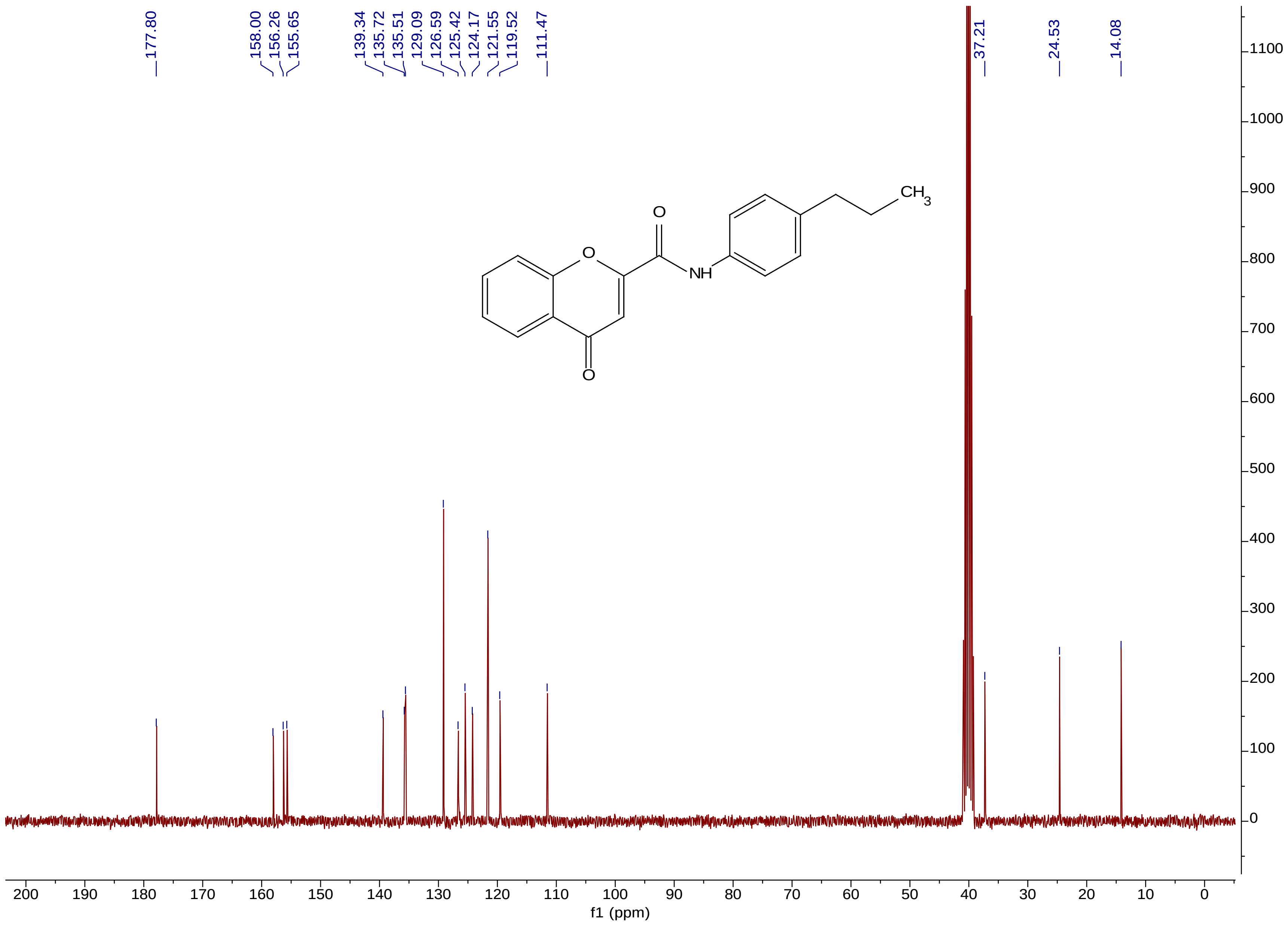

177.80
158.00
156.26
155.65
139.34
135.72
135.51
129.09
126.59
125.42
124.17
121.55
119.52
111.47
37.21
24.53
14.08
O
O
NH
O
CH3
1100
1000
900
800
700
600
500
400
300
200
100
0
200
190
180
170
160
150
140
130
120
110
100
90
80
70
60
50
40
30
20
10
0
f1 (ppm)

_N_-(4-Fluorophenyl)-4-oxo-4_H_-chromene-2-carboxamide (3e)

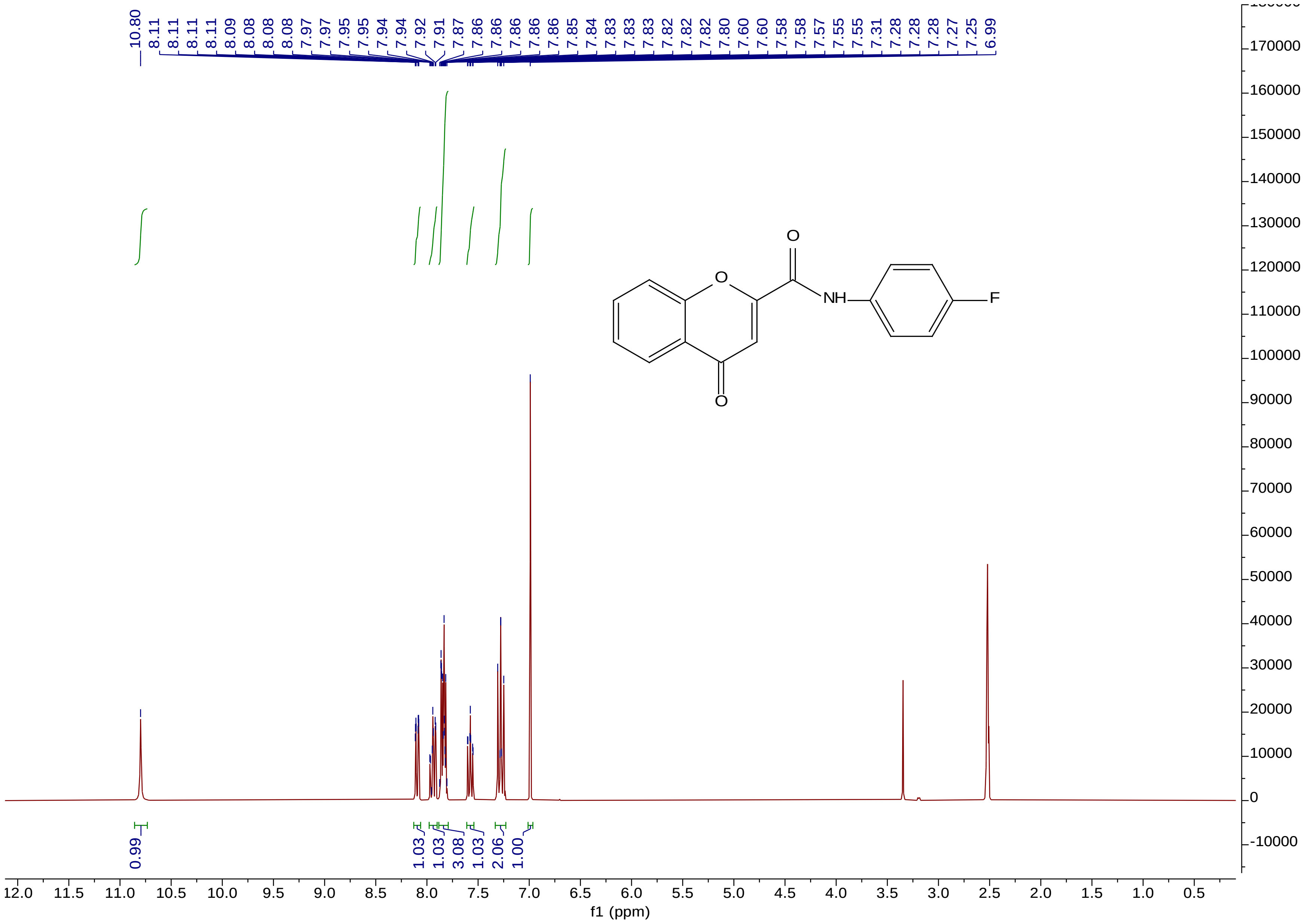

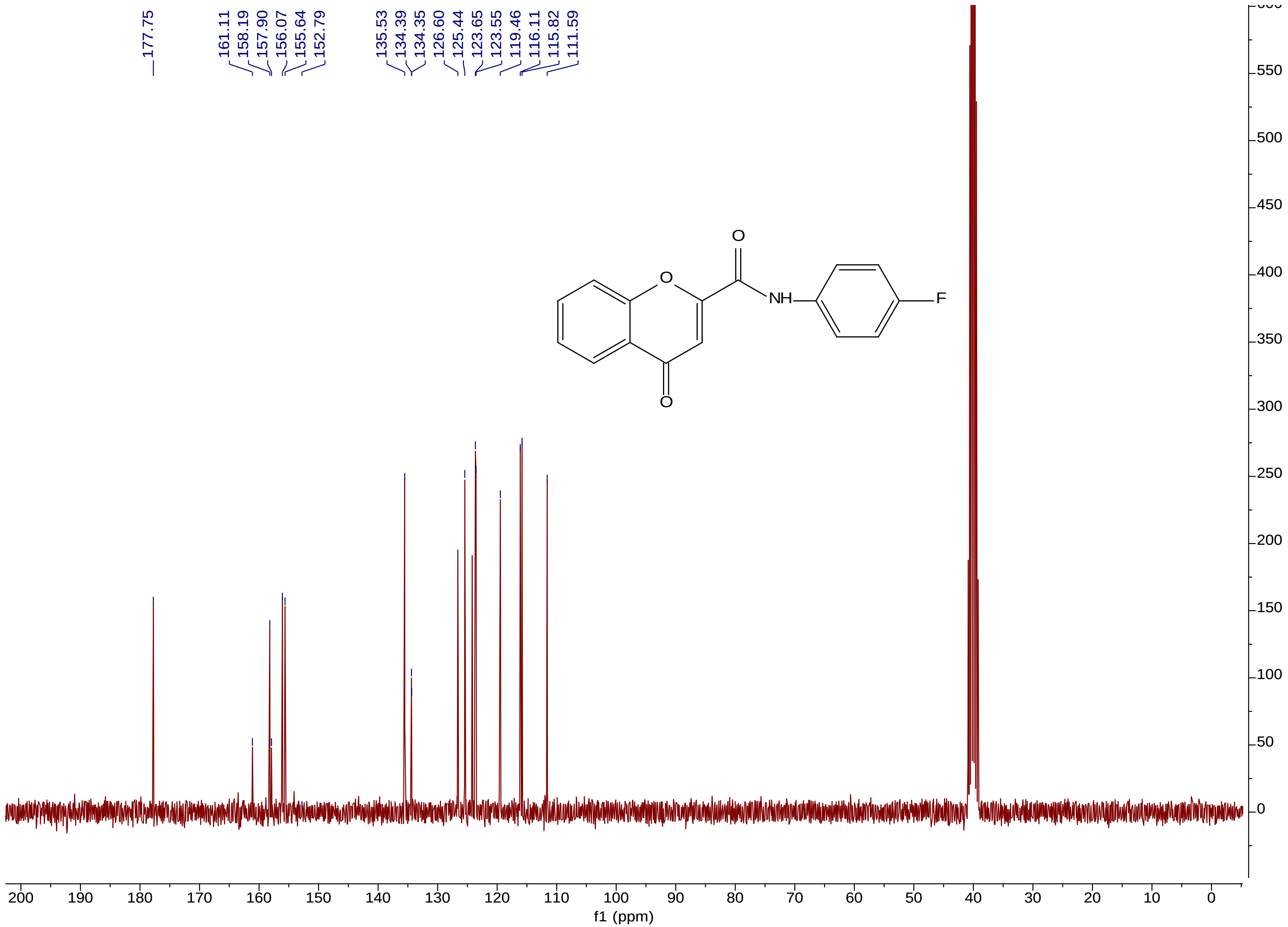
177.75
161.11
158.19
157.90
156.07
155.64
152.79
135.53
134.39
134.35
126.60
125.44
123.65
123.55
119.46
116.11
115.82
111.59
O
O
NH
F
O
f1 (ppm)

N-(4-Chlorophenyl)-4-oxo-4H-chromene-2-carboxamide (3f)

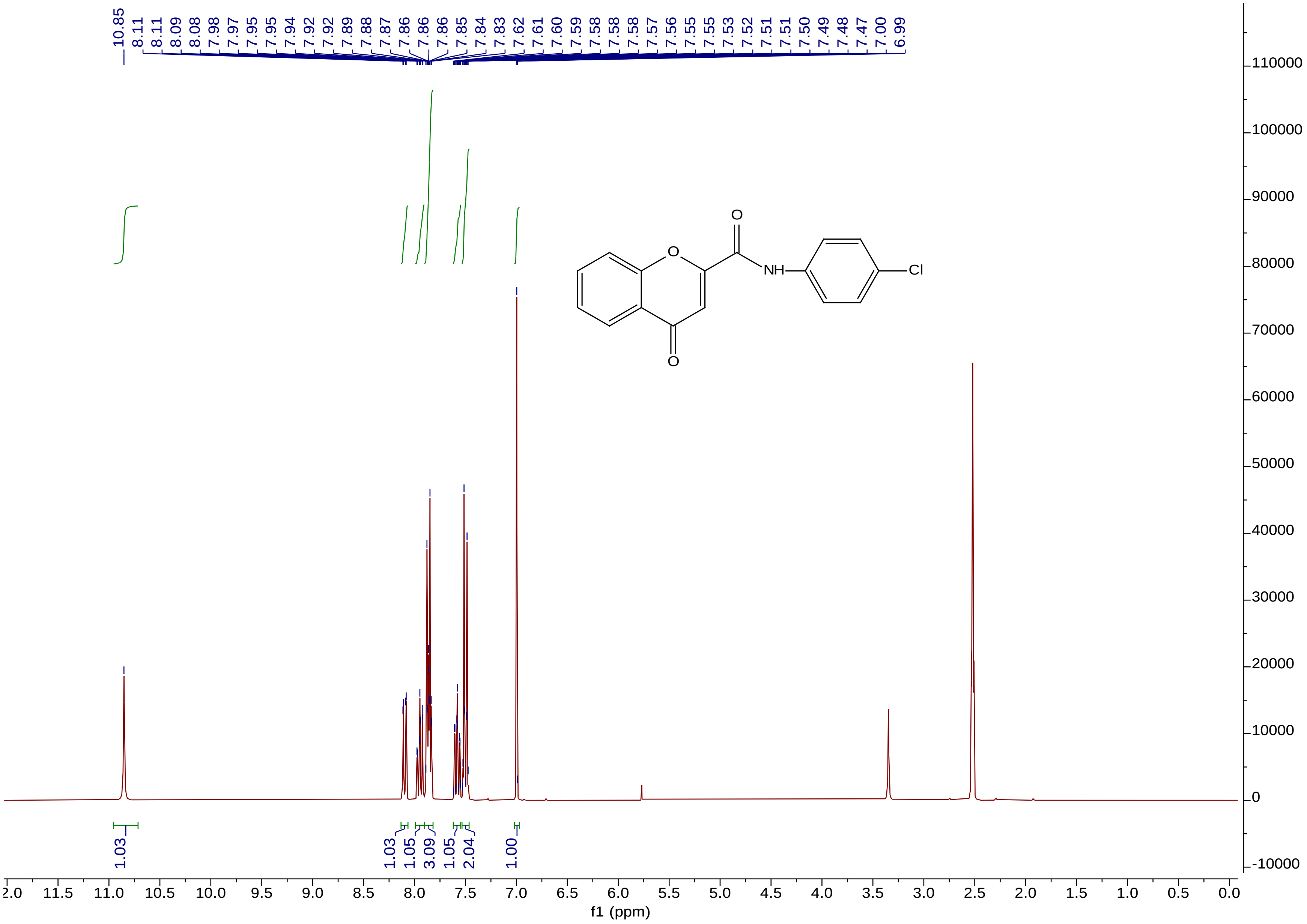

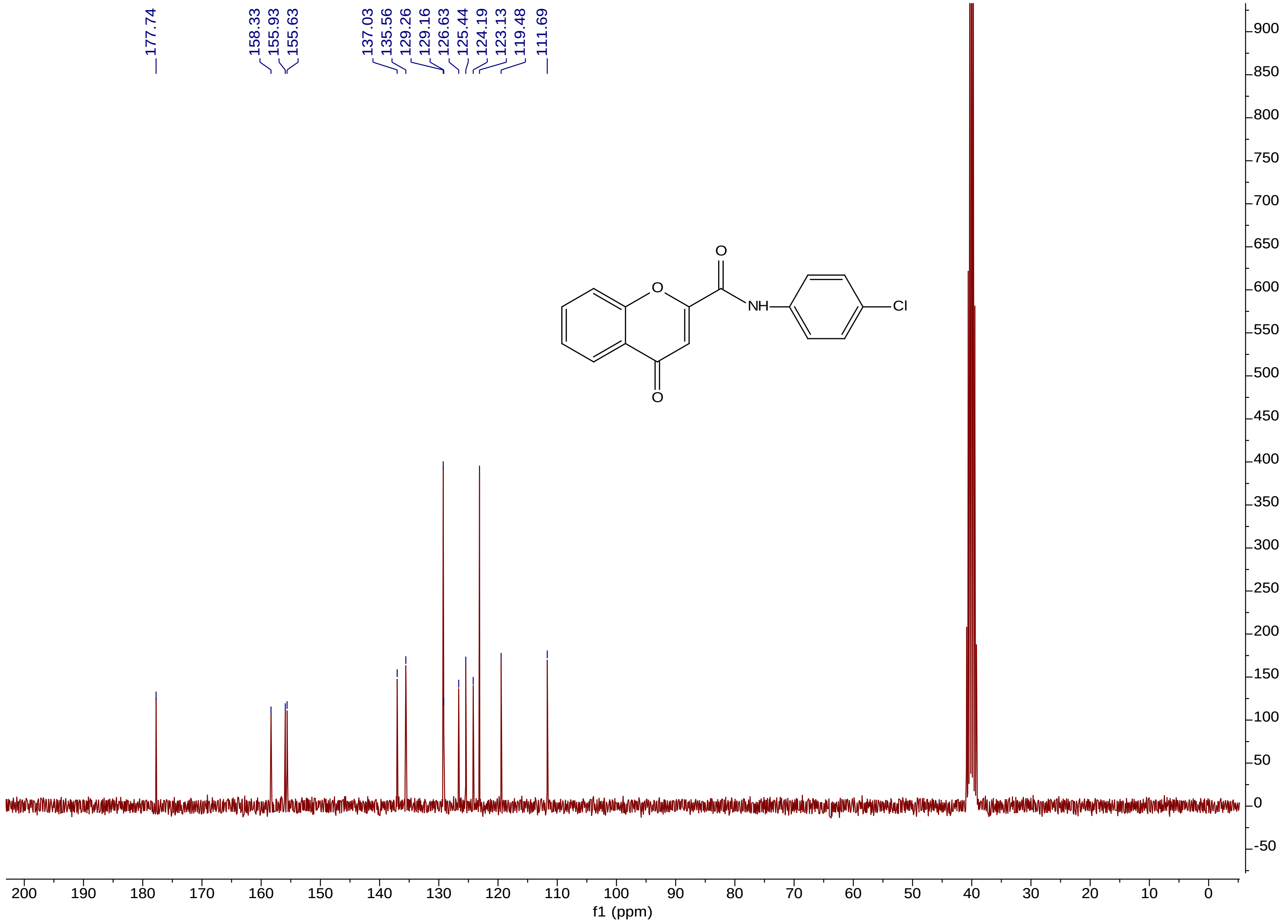
177.74
158.33
155.93
155.63
137.03
135.56
129.26
129.16
126.63
125.44
124.19
123.13
119.48
111.69
O
O
NH
Cl
O
f1 (ppm)

_N_-(4-Bromophenyl)-4-oxo-4_H_-chromene-2-carboxamide (3g)

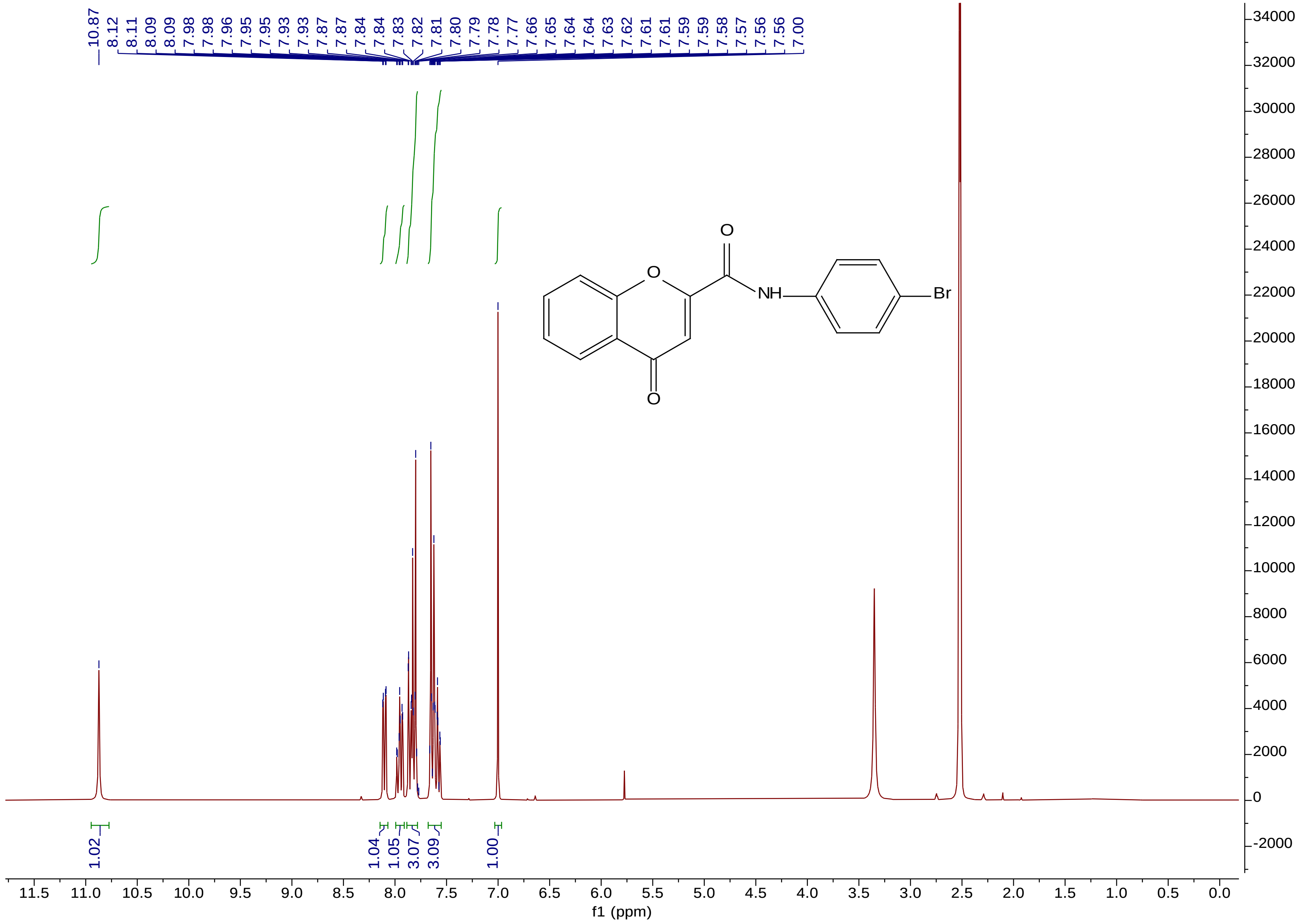

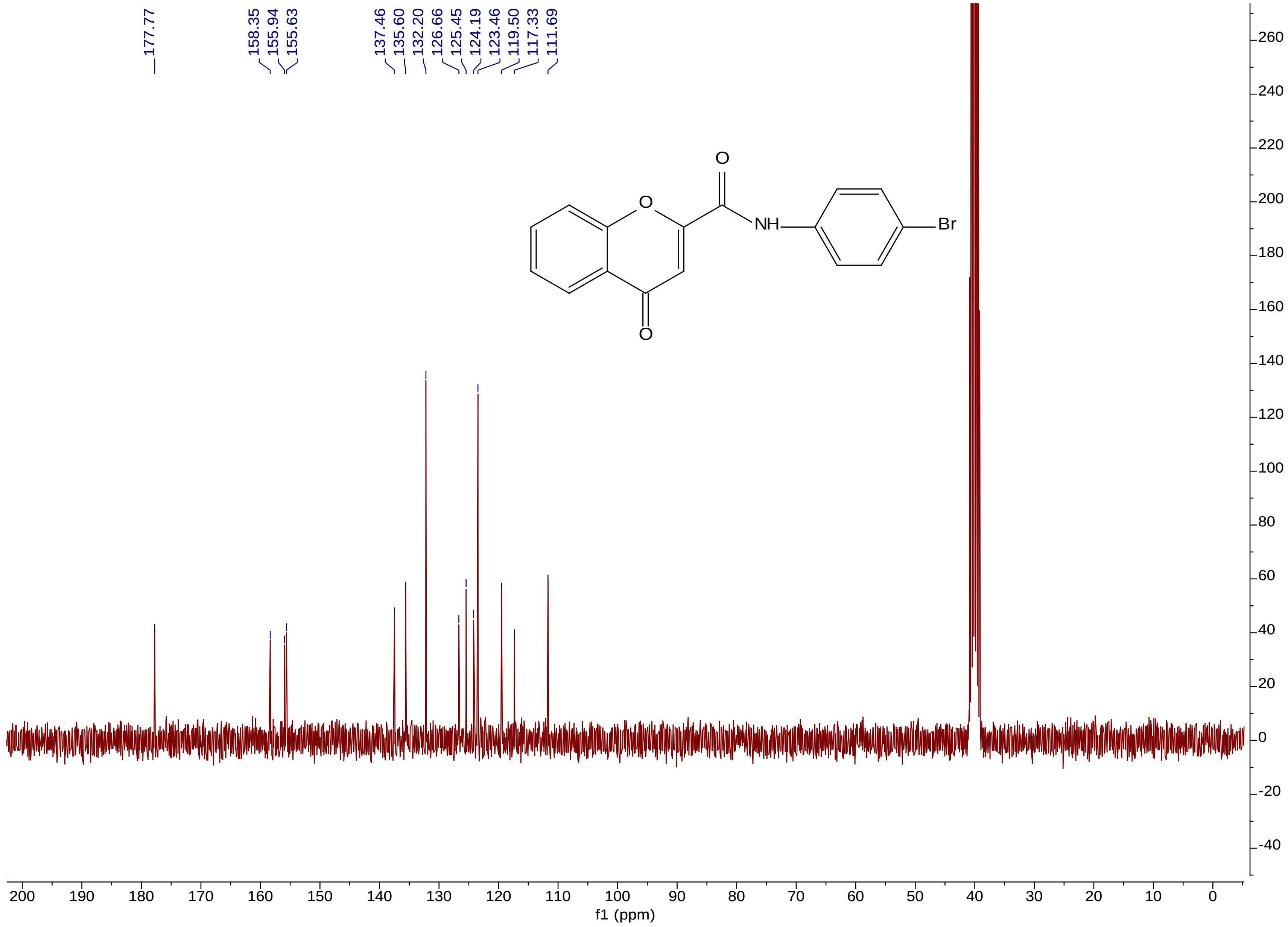
177.77
158.35
155.94
155.63
137.46
135.60
132.20
126.66
125.45
124.19
123.46
119.50
117.33
111.69
O
O
NH
Br
O
f1 (ppm)

N-(2-Fluorophenyl)-4-oxo-4*H*-chromene-2-carboxamide (3h)

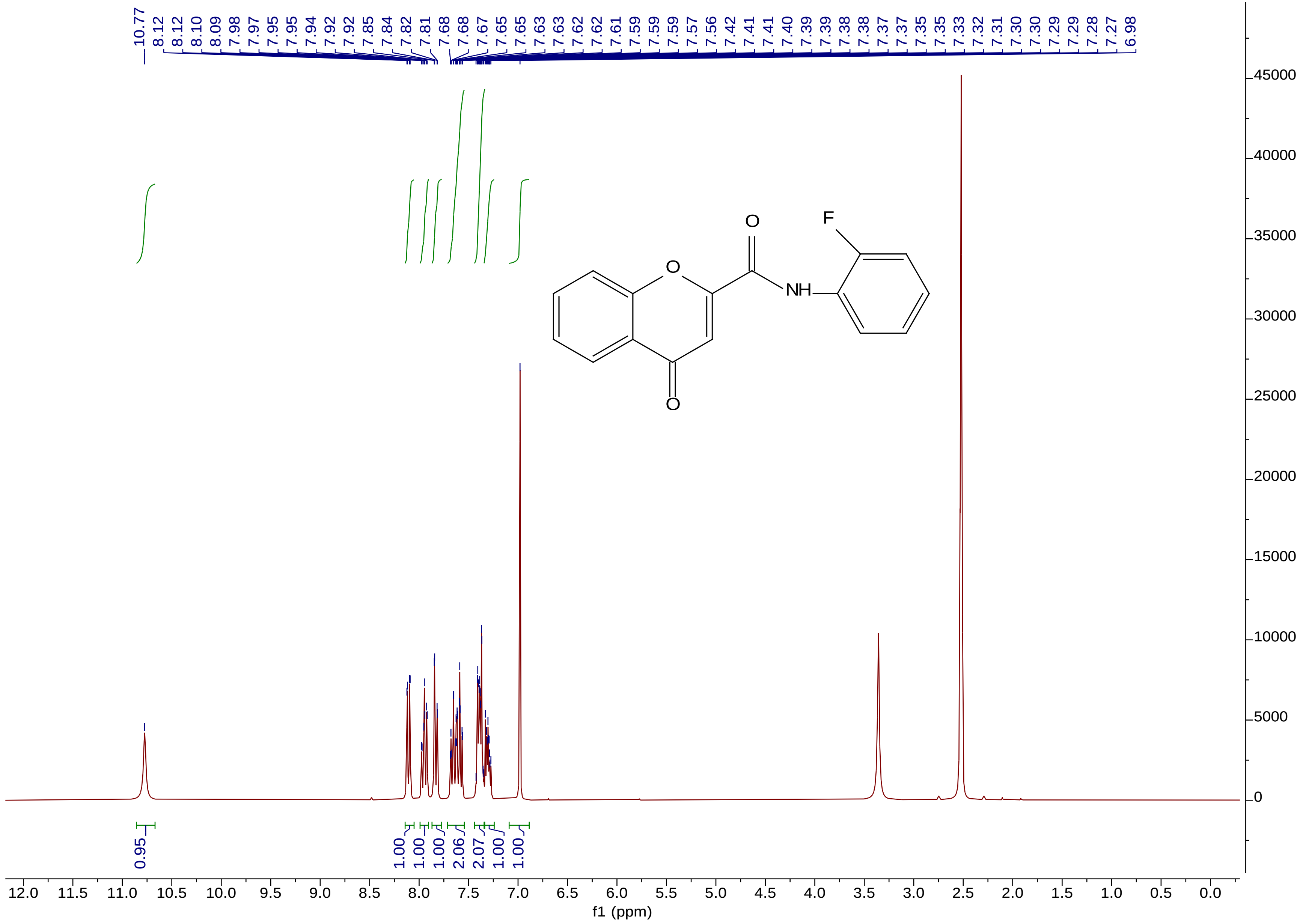

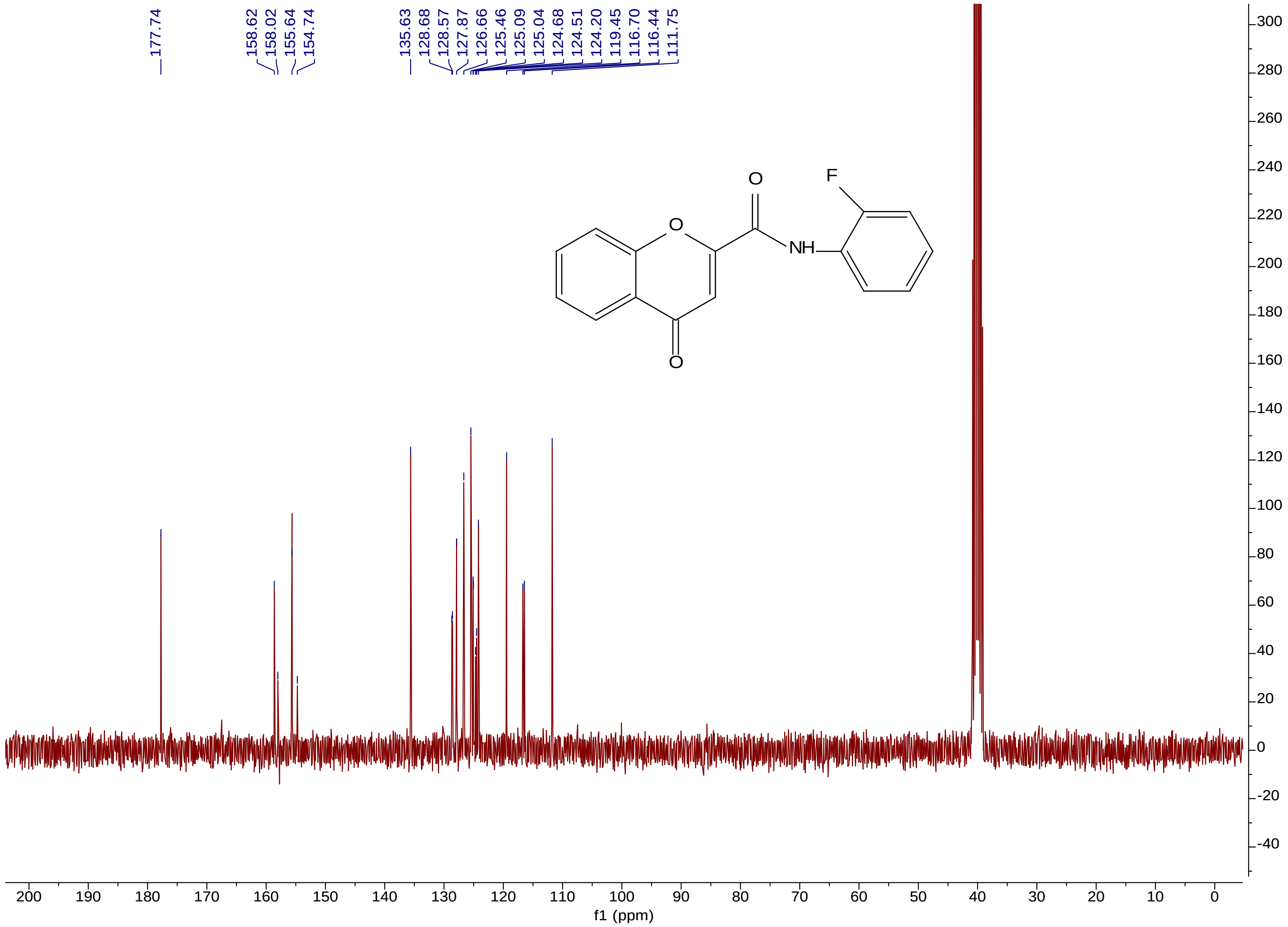
177.74
158.62
158.02
155.64
154.74
135.63
128.68
128.57
127.87
126.66
125.46
125.09
125.04
124.68
124.51
124.20
119.45
116.70
116.44
111.75
O
F
O
NH
O
f1 (ppm)

_N_-(2-Chlorophenyl)-4-oxo-4_H_-chromene-2-carboxamide (3i)

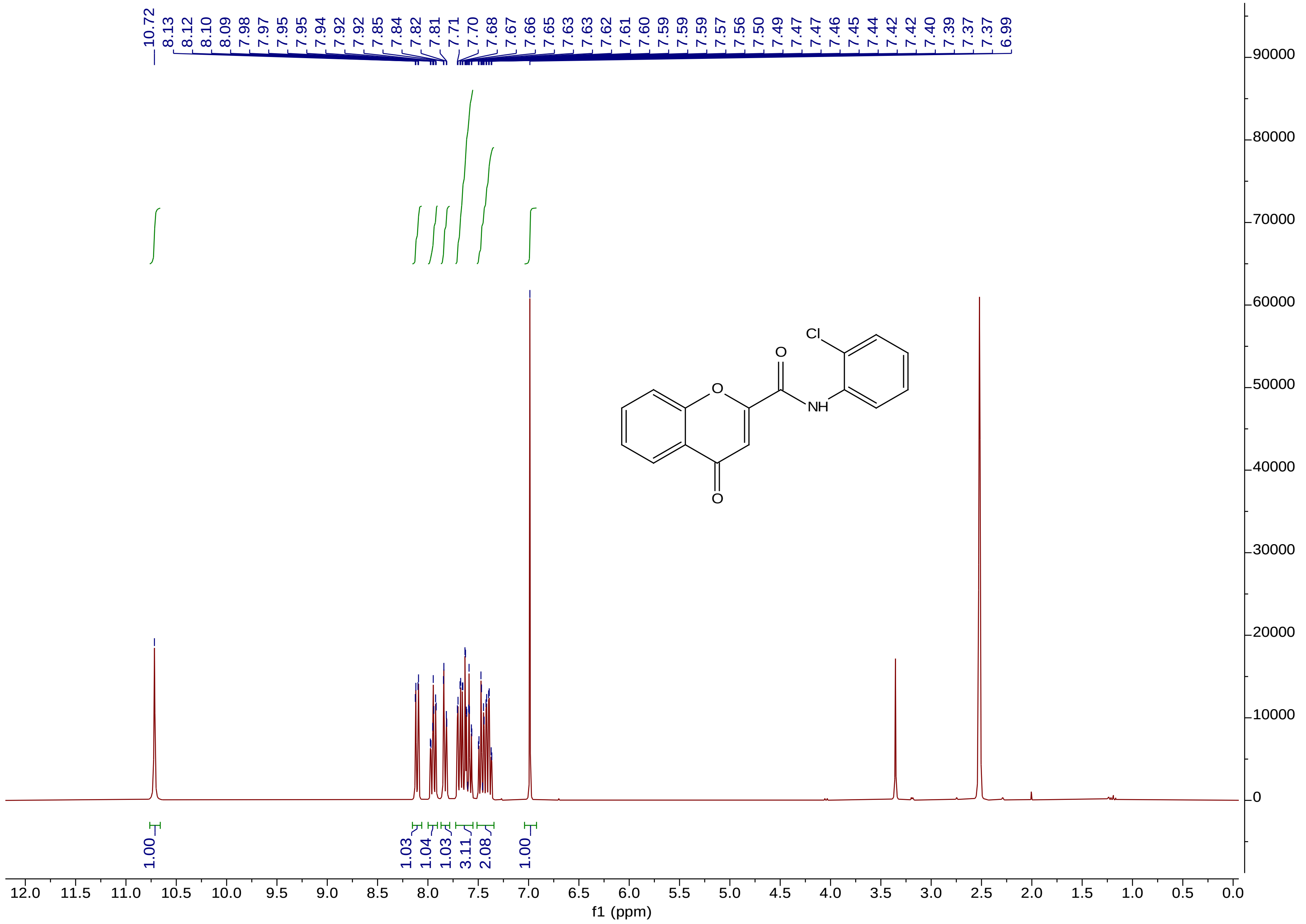

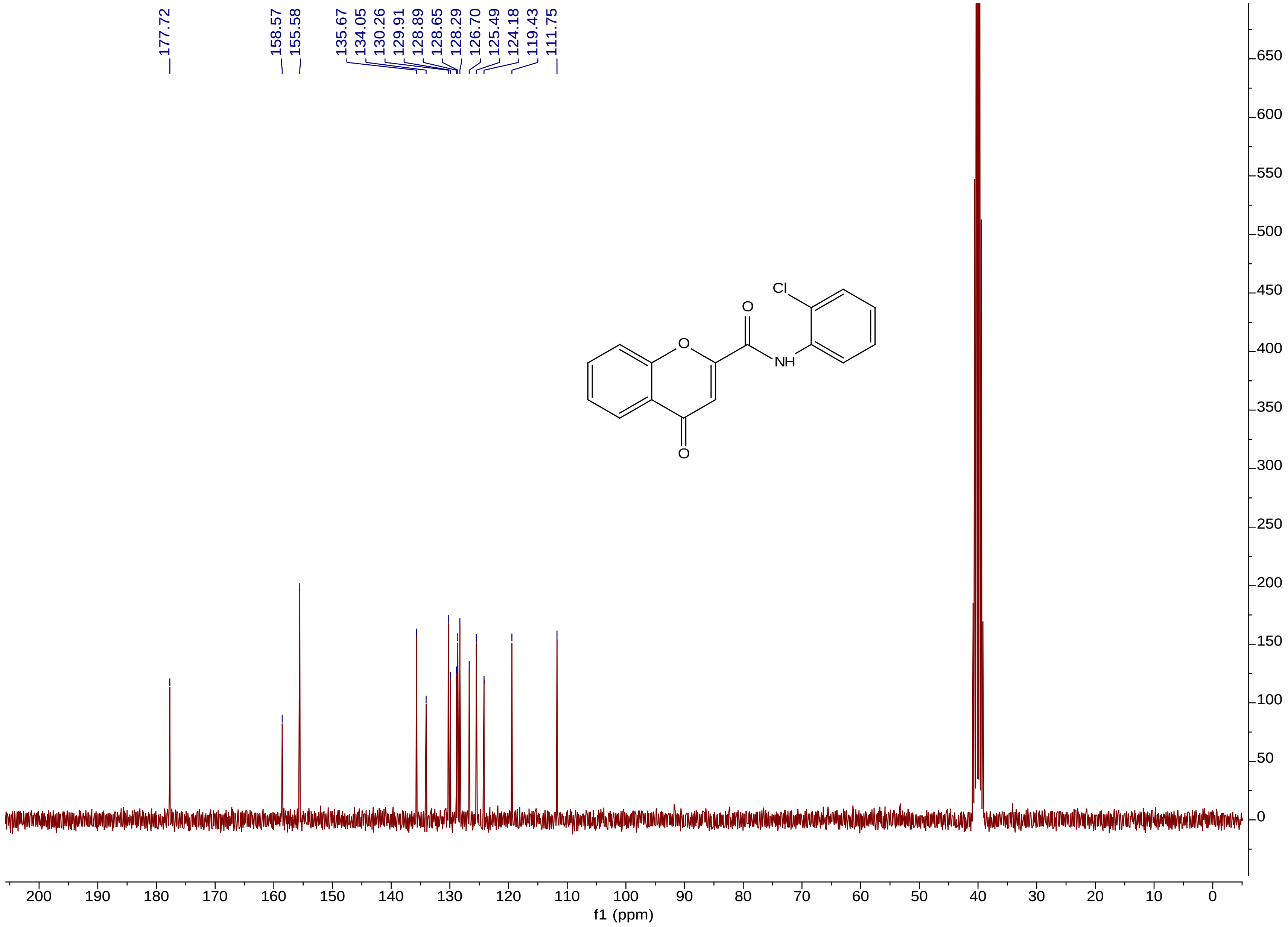
177.72
158.57
155.58
135.67
134.05
130.26
129.91
128.89
128.65
128.29
126.70
125.49
124.18
119.43
111.75
Cl
O
O
NH
O
f1 (ppm)

_N_-(2-Bromophenyl)-4-oxo-4_H_-chromene-2-carboxamide (3j)

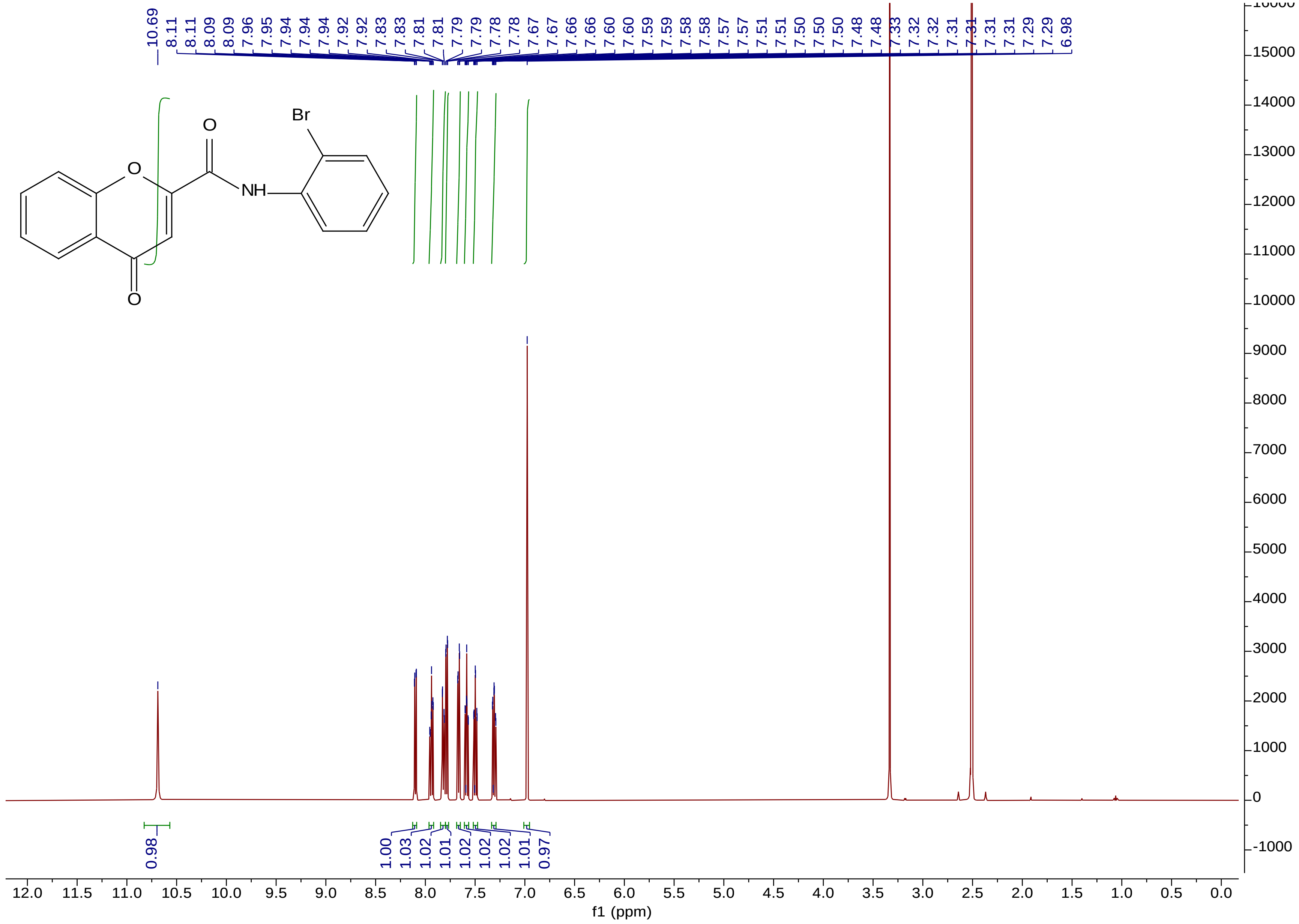

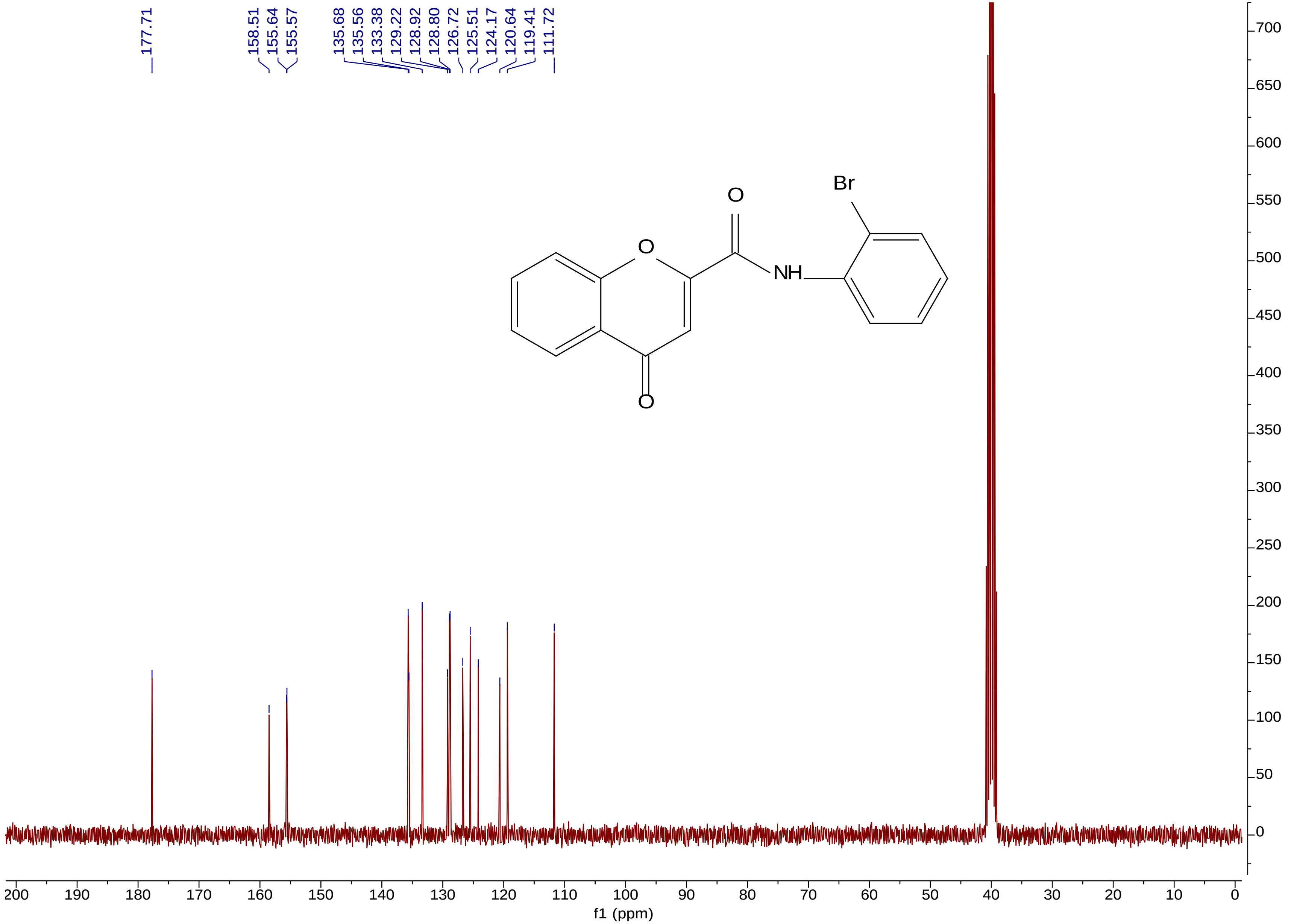
177.71
158.51
155.64
155.57
135.68
135.56
133.38
129.22
128.92
128.80
126.72
125.51
124.17
120.64
119.41
111.72
O
Br
O
NH
O
f1 (ppm)

_N_-(2-Chloro-4-fluorophenyl)-4-oxo-4_H_-chromene-2-carboxamide (3k)

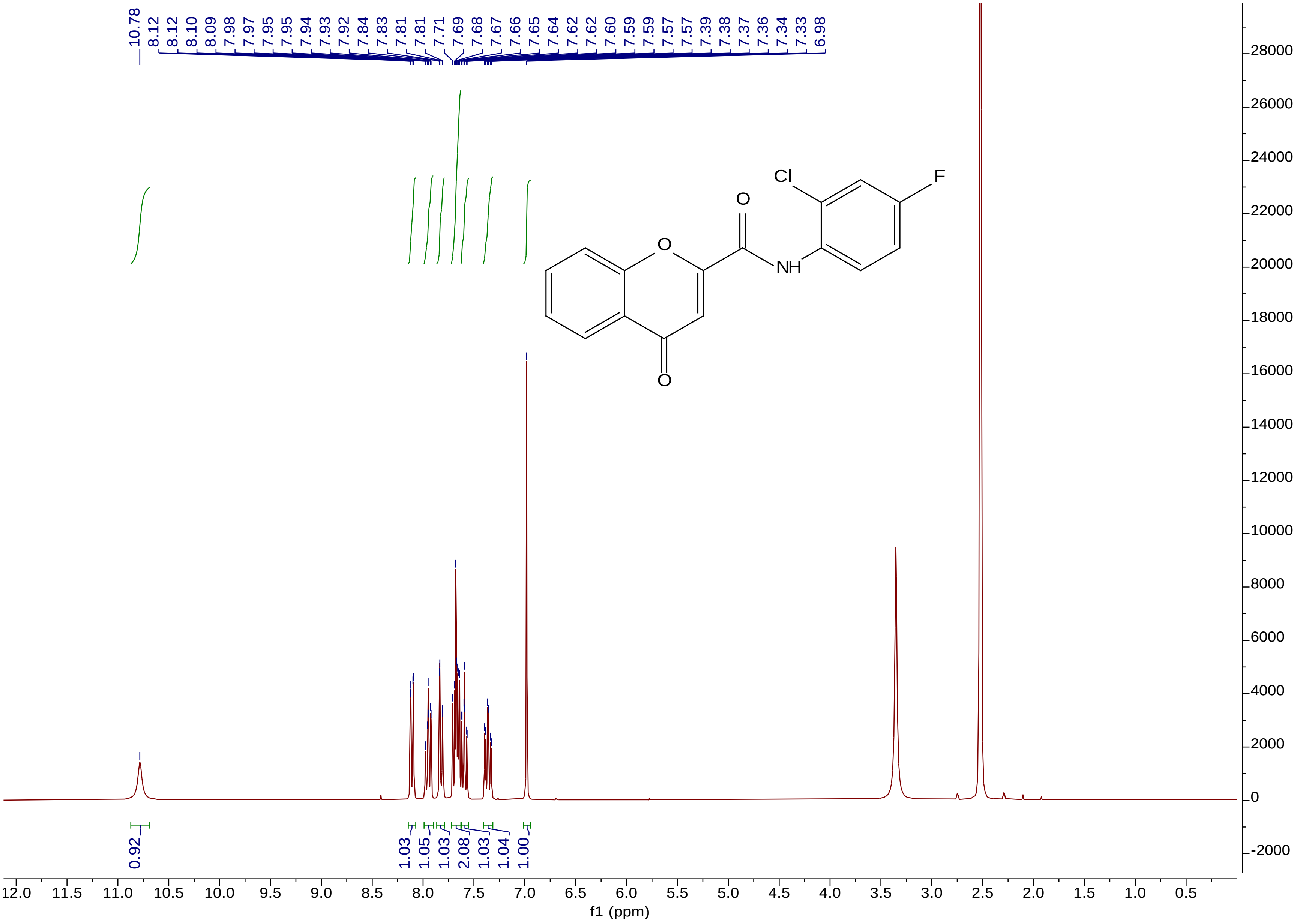

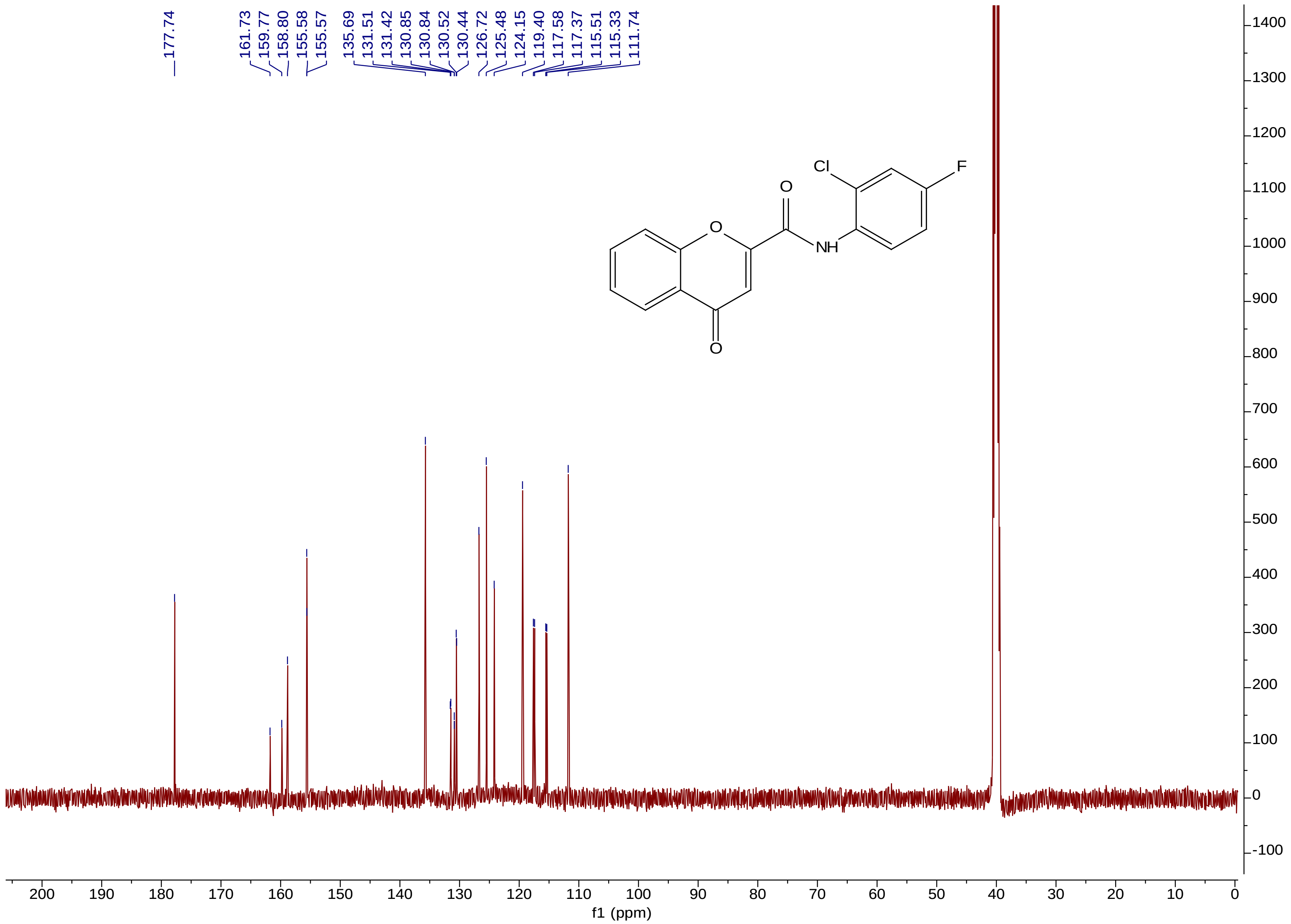
177.74
161.73
159.77
158.80
155.58
155.57
135.69
131.51
131.42
130.85
130.84
130.52
130.44
126.72
125.48
124.15
119.40
117.58
117.37
115.51
115.33
111.74
Cl
F
O
O
NH
O
f1 (ppm)

N-(3-Cchloro-4-fluorophenyl)-4-oxo-4H-chromene-2-carboxamide (3l)

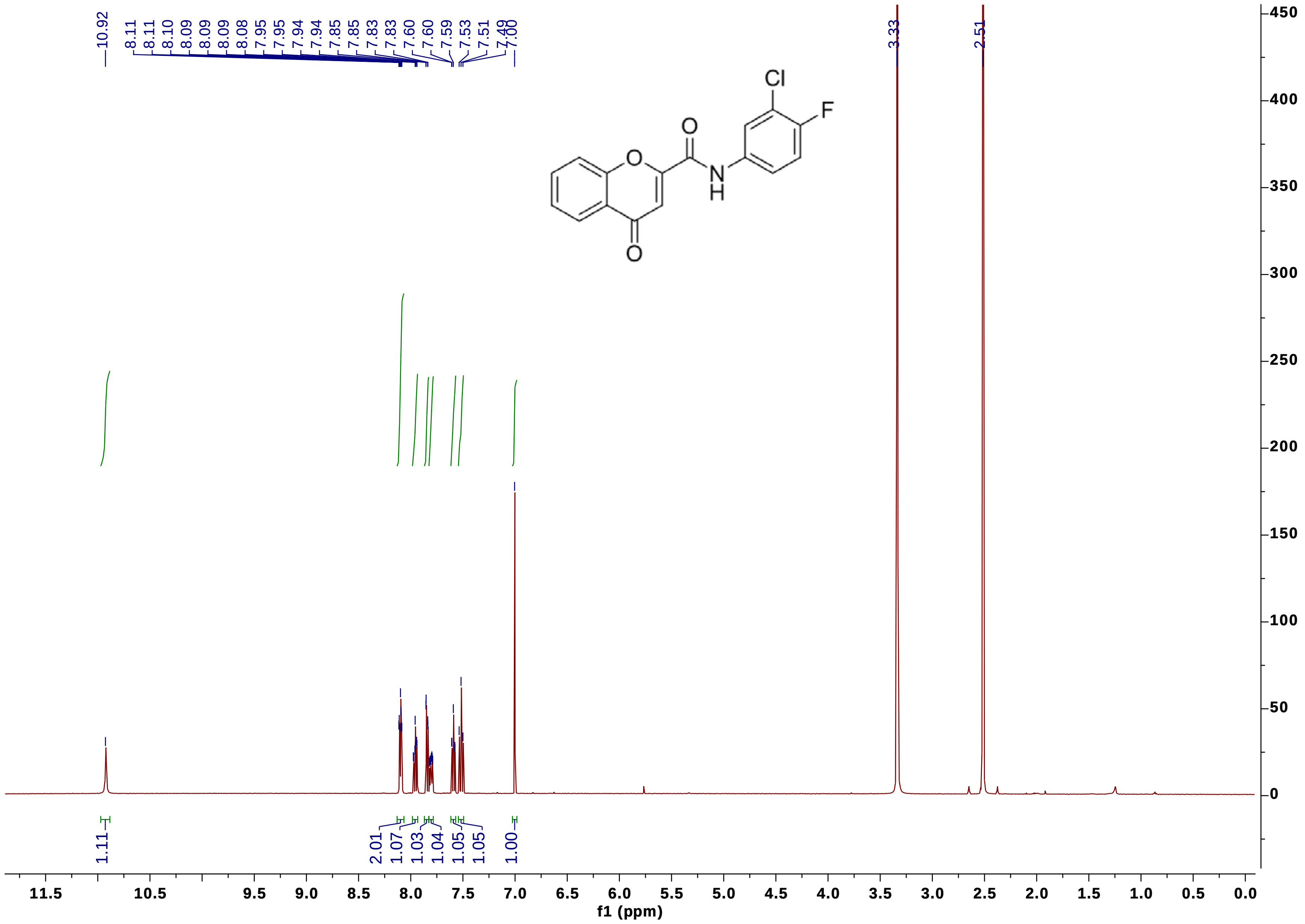

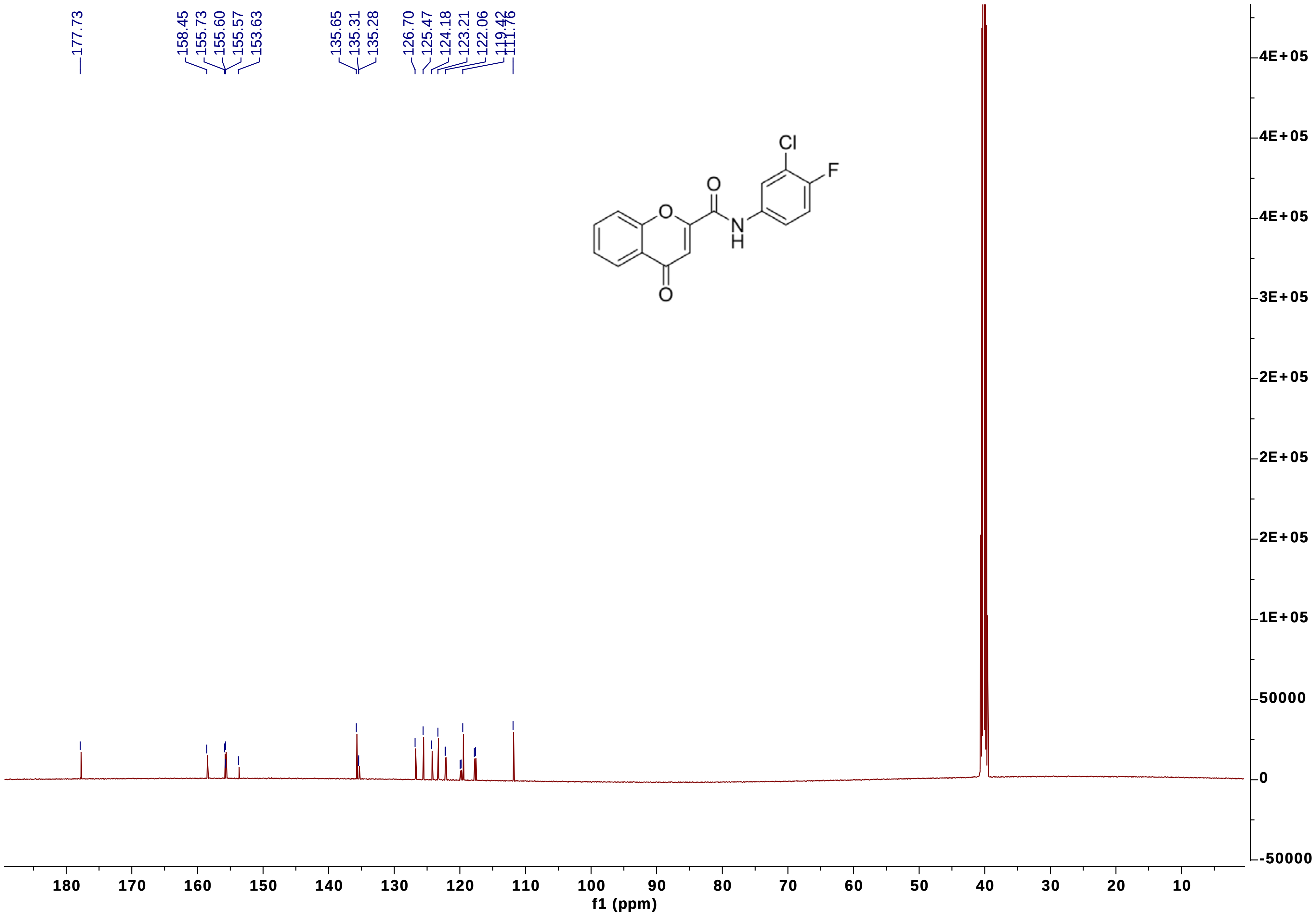
177.73
158.45
155.73
155.60
155.57
153.63
135.65
135.31
135.28
126.70
125.47
124.18
123.21
122.06
119.42
111.76
Cl
F
O
N
H
O
O
f1 (ppm)

N-(4-Nitrophenyl)-4-oxo-4H-chromene-2-carboxamide (3m)

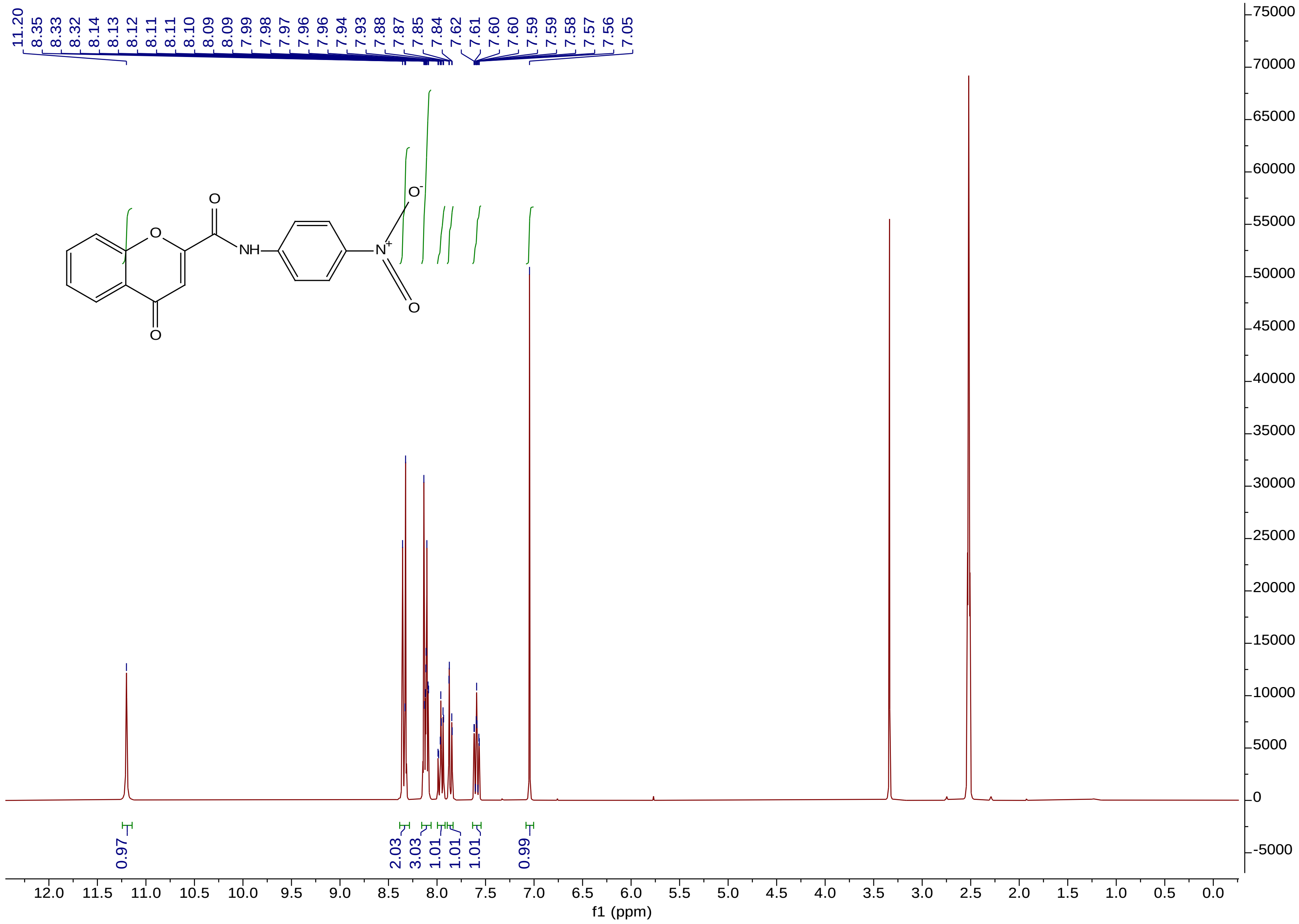

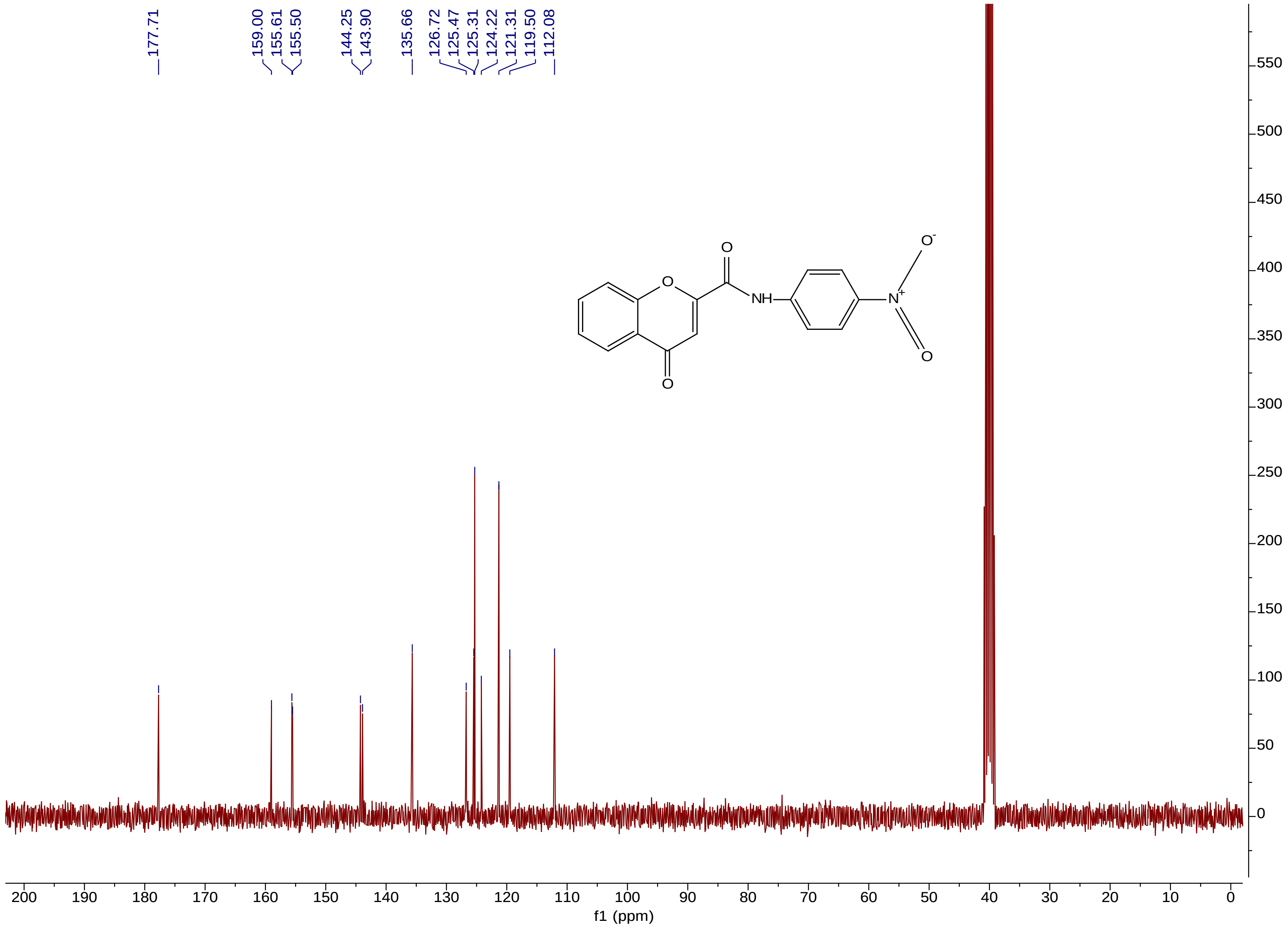
177.71
159.00
155.61
155.50
144.25
143.90
135.66
126.72
125.47
125.31
124.22
121.31
119.50
112.08
O
O
NH
N+
O-
O
O
f1 (ppm)

N-(3,5-Dinitrophenyl)-4-oxo-4*H*-chromene-2-carboxamide (3o)

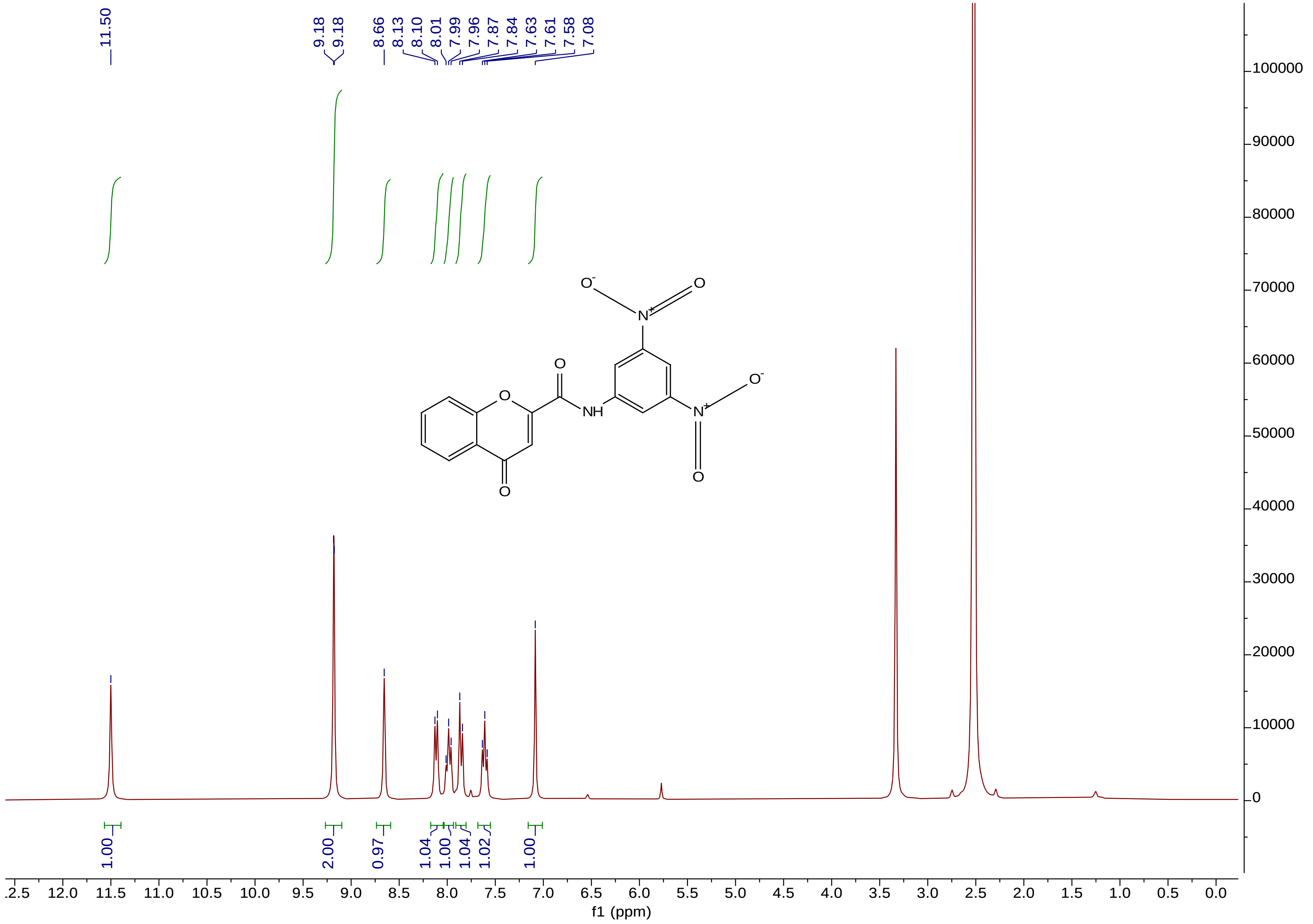

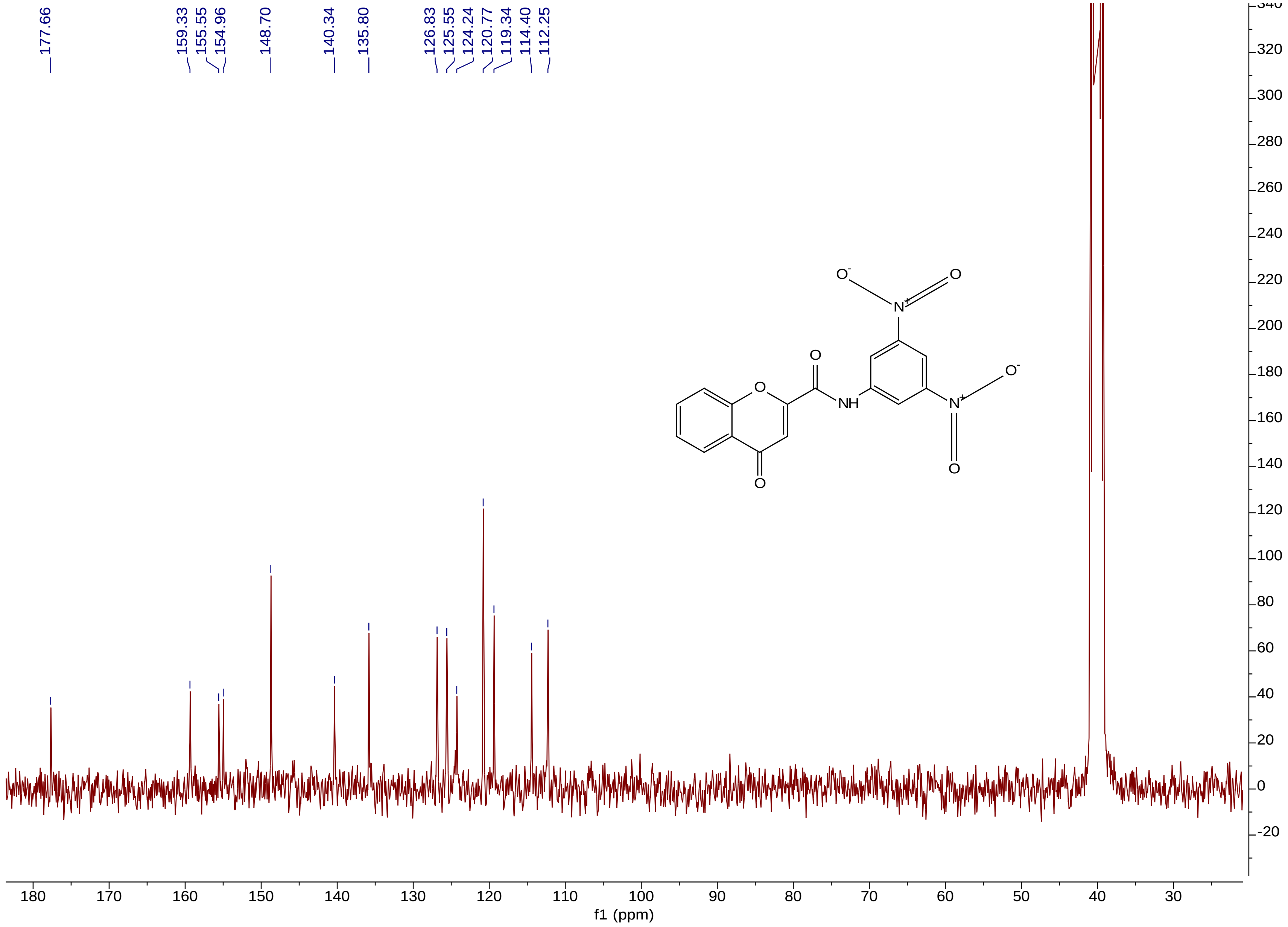
177.66
159.33
155.55
154.96
148.70
140.34
135.80
126.83
125.55
124.24
120.77
119.34
114.40
112.25
f1 (ppm)
NH
O

_N_-(4-Methoxyphenyl)-4-oxo-4_H_-chromene-2-carboxamide (3p)

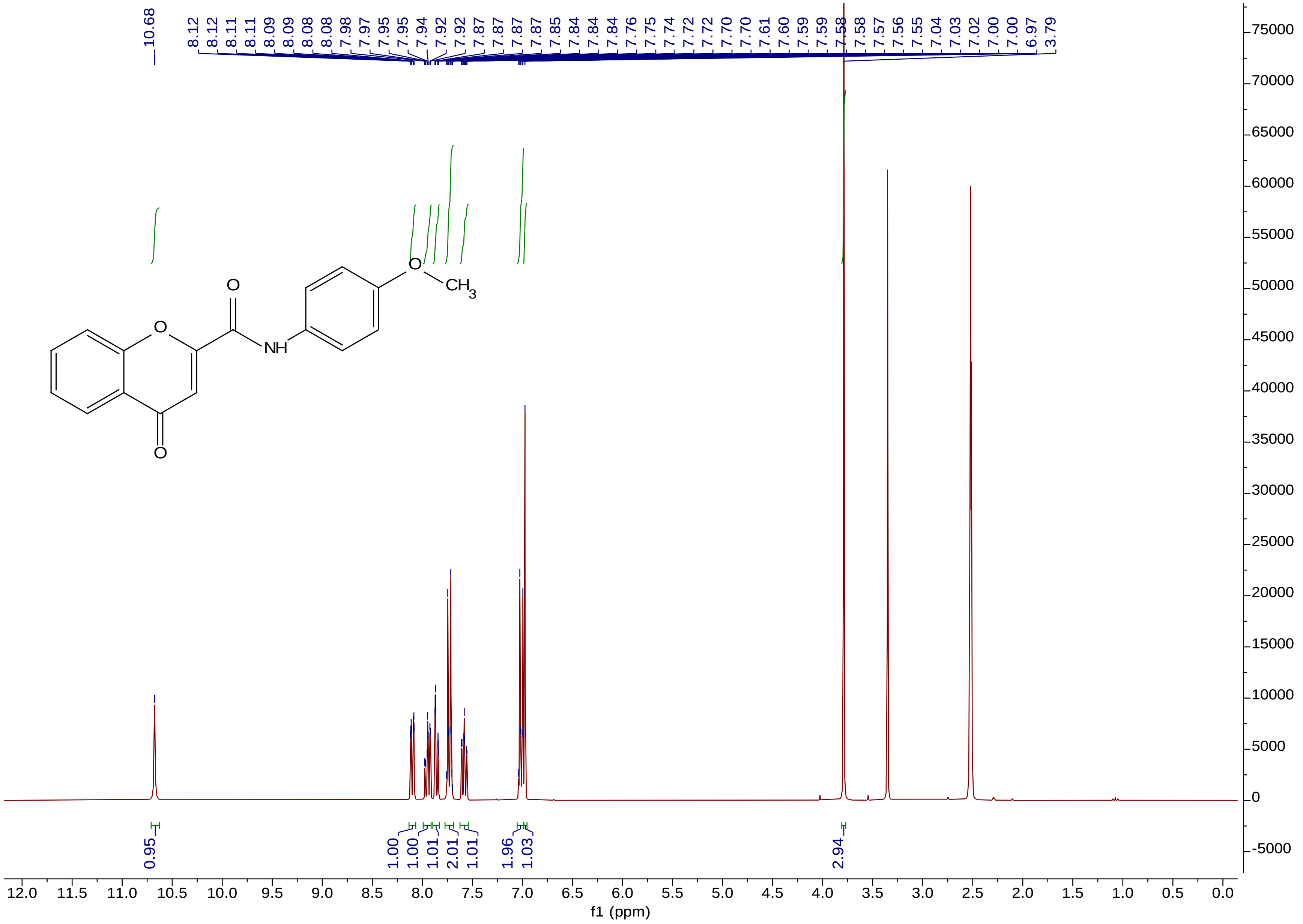

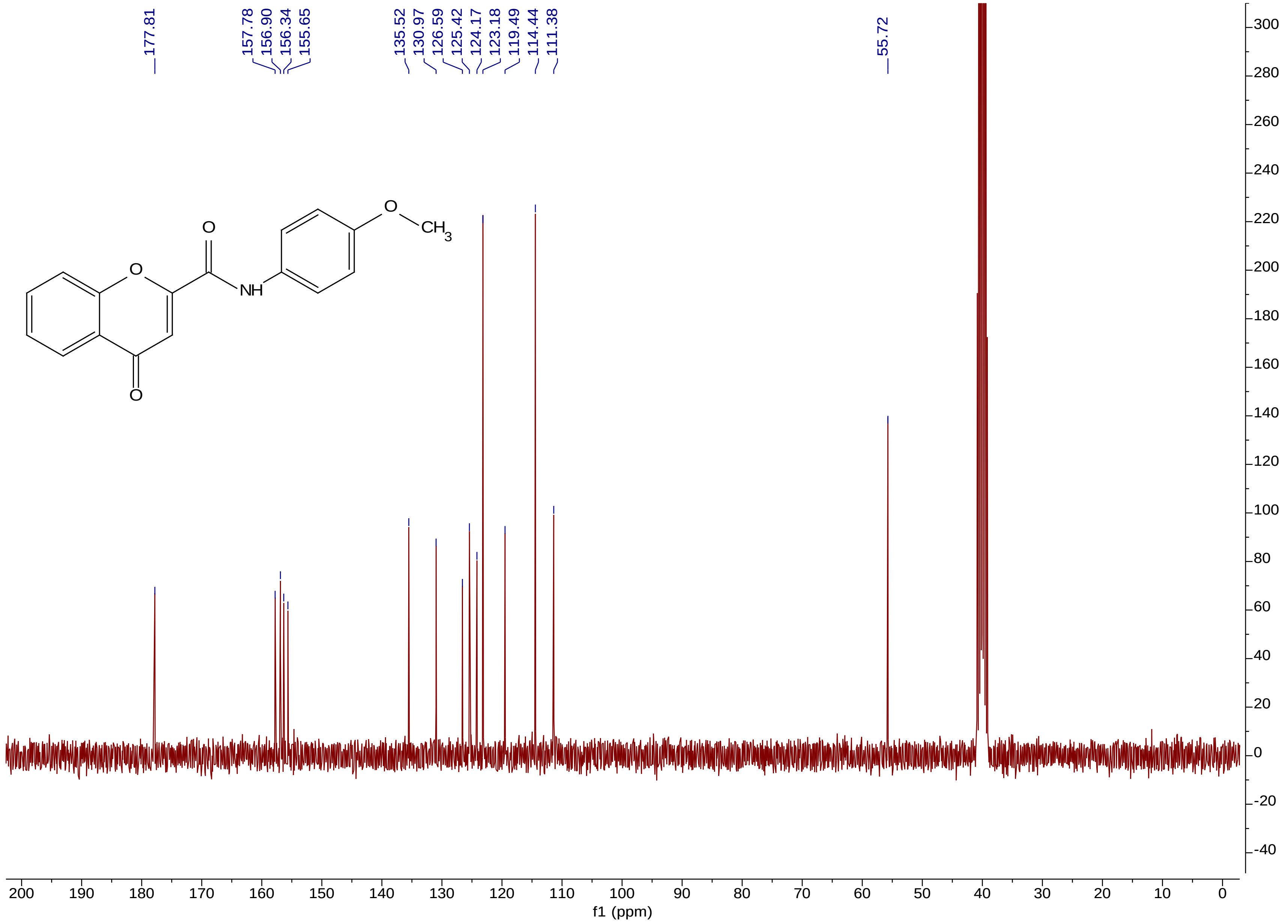
177.81
157.78
156.90
156.34
155.65
135.52
130.97
126.59
125.42
124.17
123.18
119.49
114.44
111.38
55.72
O
O
O
NH
O
CH3
f1 (ppm)

***N*-(2-Methoxyphenyl)-4-oxo-4*H*-chromene-2-carboxamide (3q)**

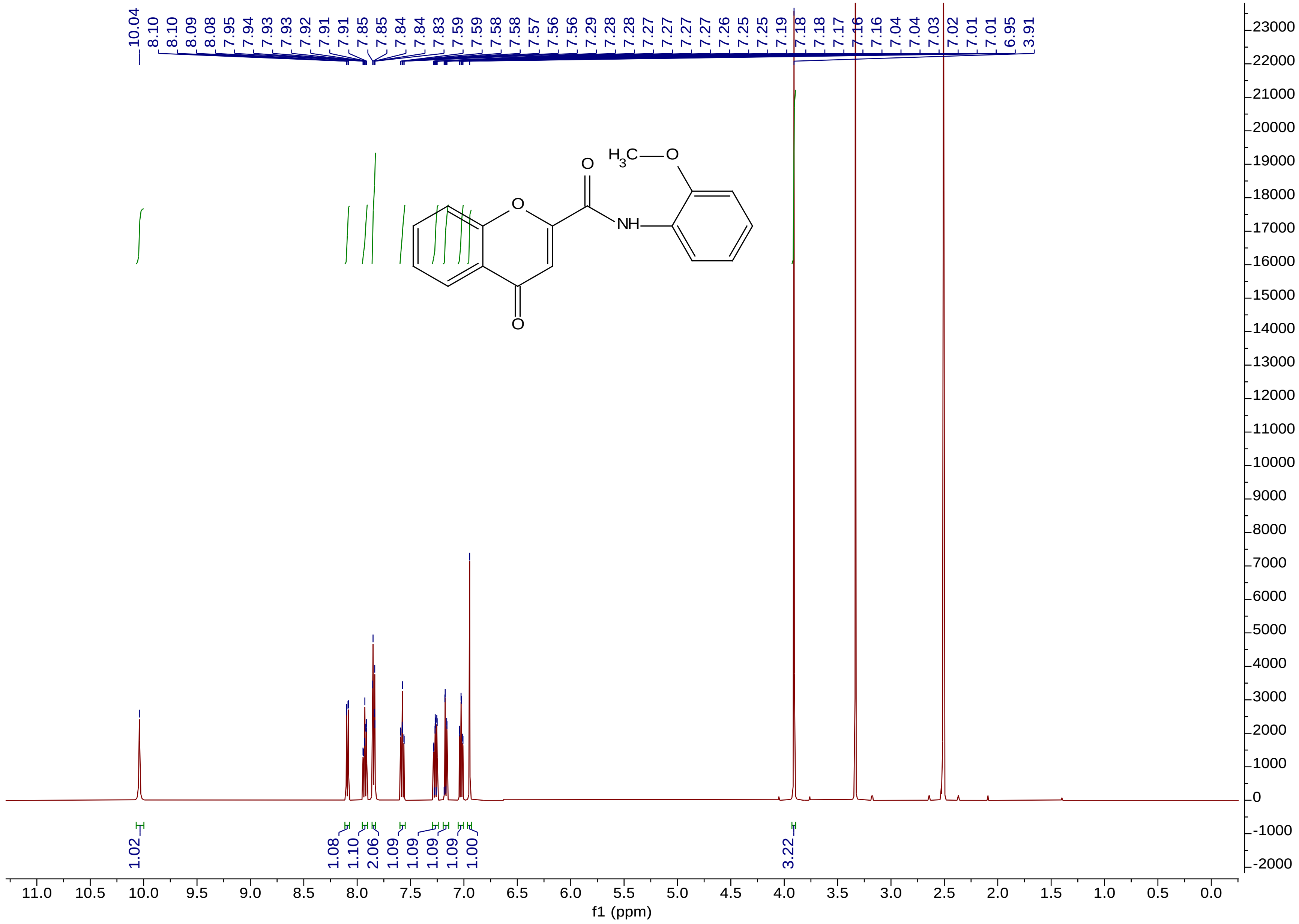

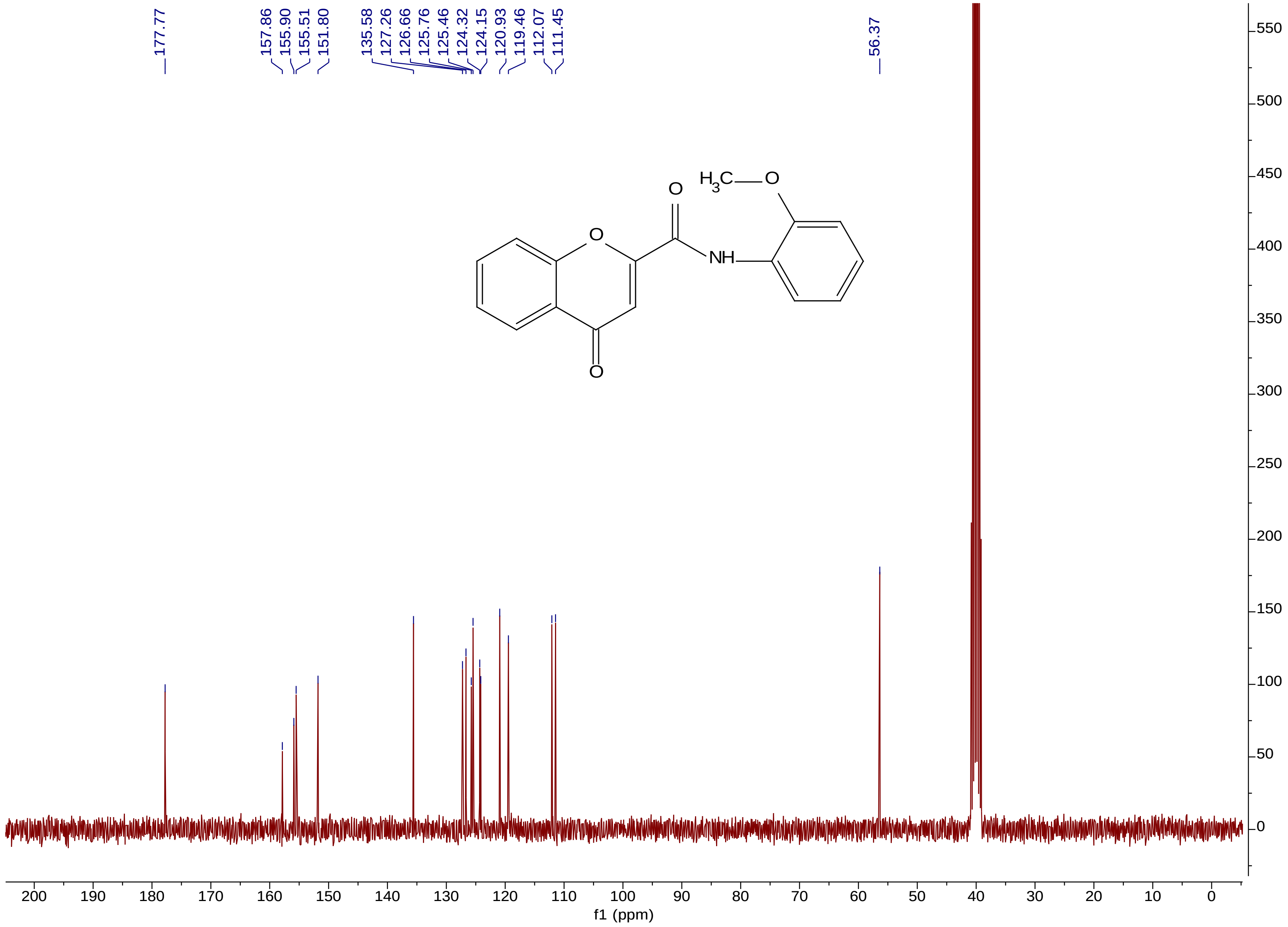
177.77
157.86
155.90
155.51
151.80
135.58
127.26
126.66
125.76
125.46
124.32
124.15
120.93
119.46
112.07
111.45
56.37
O
H3C
O
O
NH
O
f1 (ppm)

_N_-(2-(Methylthio)phenyl)-4-oxo-4_H_-chromene-2-carboxamide (3r)

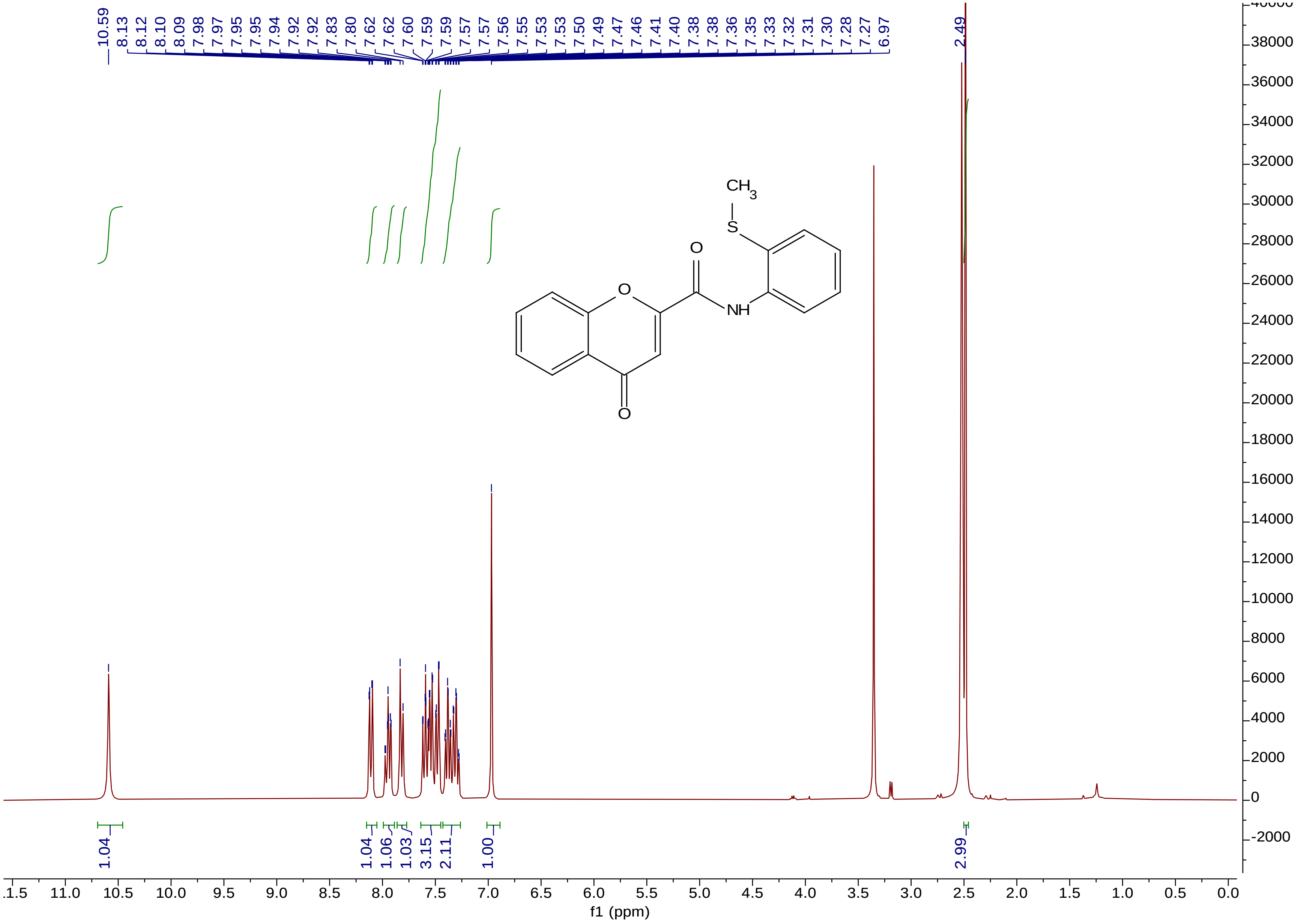

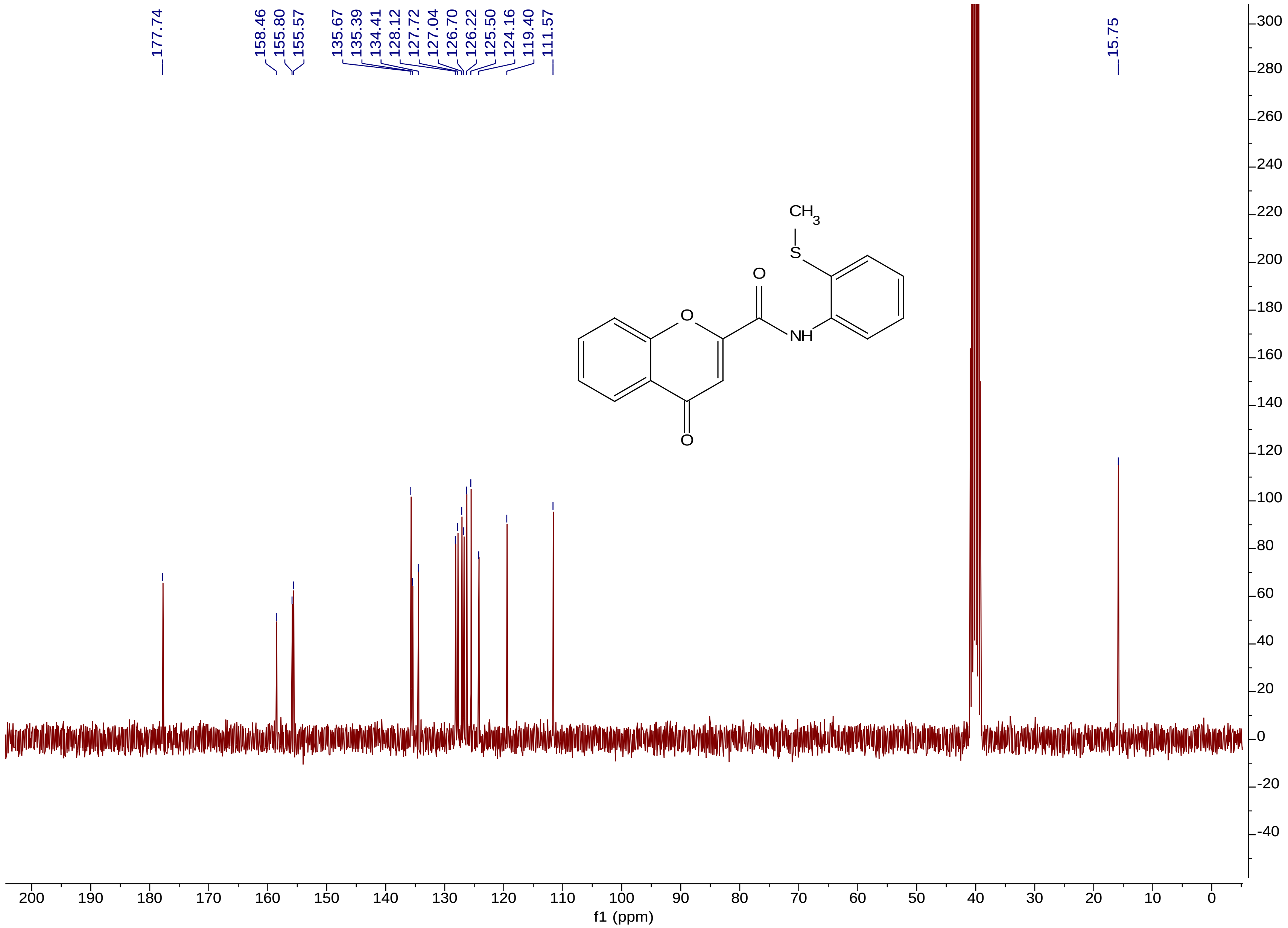
177.74
158.46
155.80
155.57
135.67
135.39
134.41
128.12
127.72
127.04
126.70
126.22
125.50
124.16
119.40
111.57
15.75
CH3
S
O
NH
O
O
f1 (ppm)

***N*-(3,4-Dimethoxyphenyl)-4-oxo-4*H*-chromene-2-carboxamide (3s)**

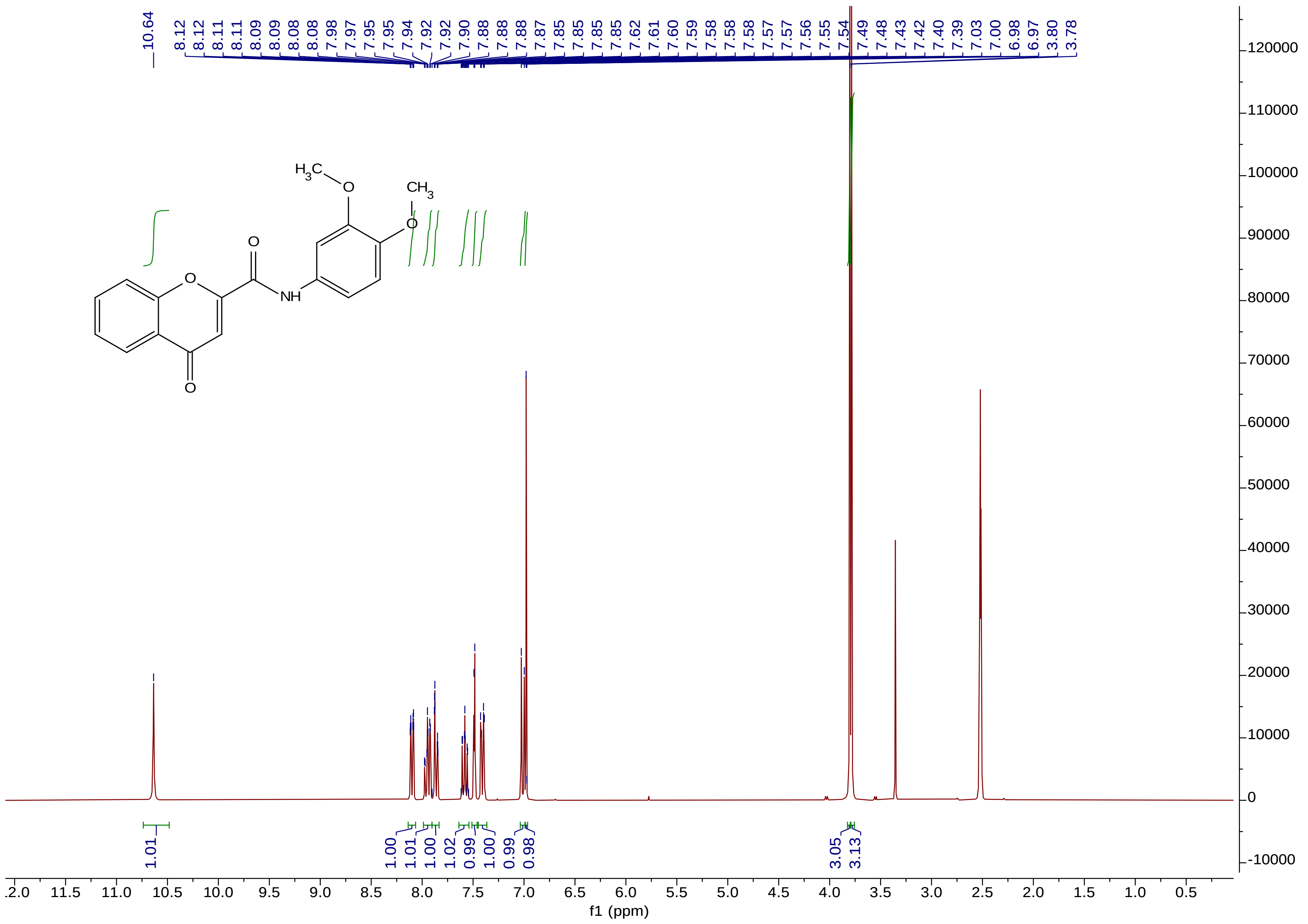

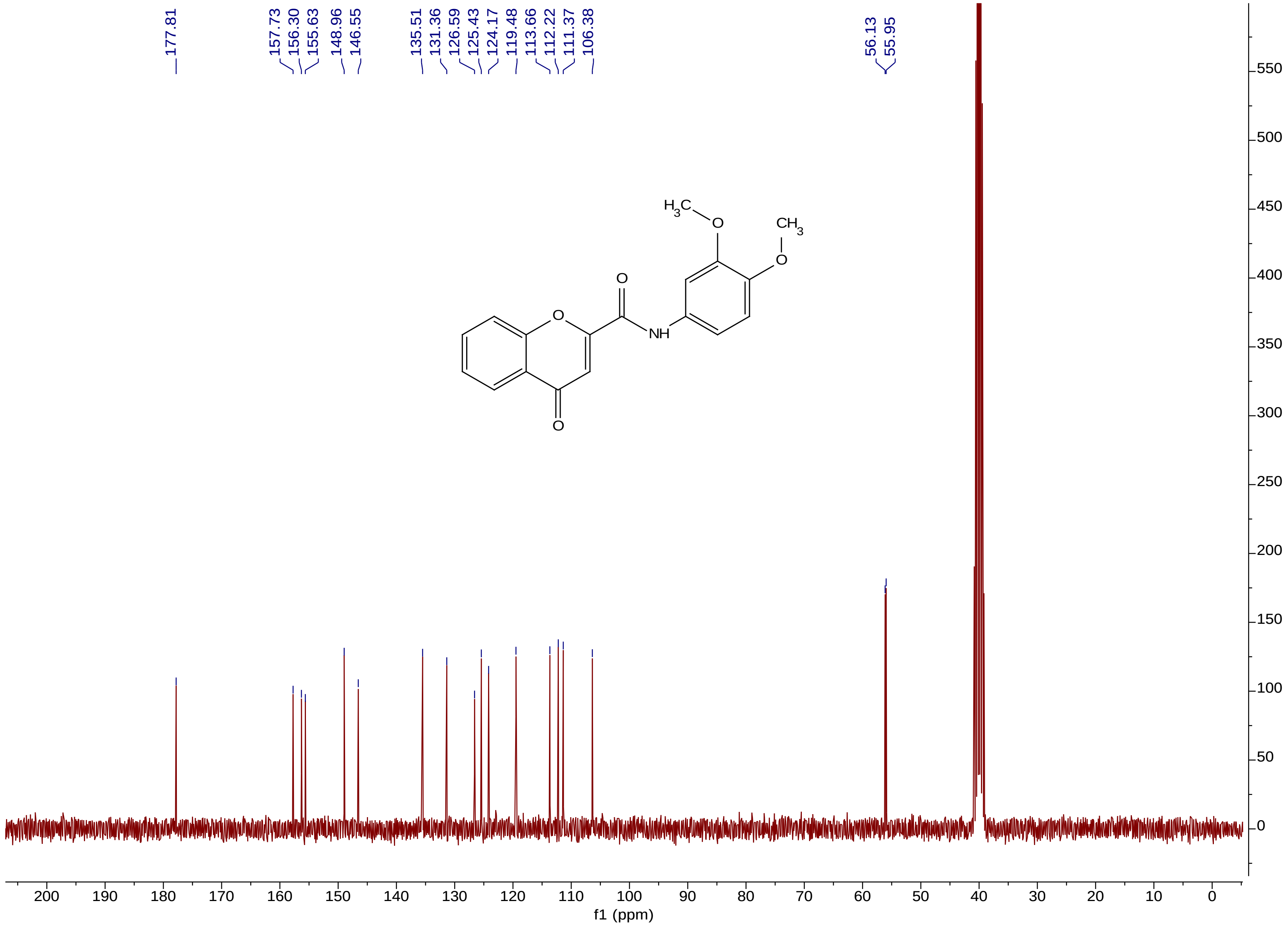
177.81
157.73
156.30
155.63
148.96
146.55
135.51
131.36
126.59
125.43
124.17
119.48
113.66
112.22
111.37
106.38
56.13
55.95
f1 (ppm)

***N*-(3,5-Dimethoxyphenyl)-4-oxo-4*H*-chromene-2-carboxamide (3t)**

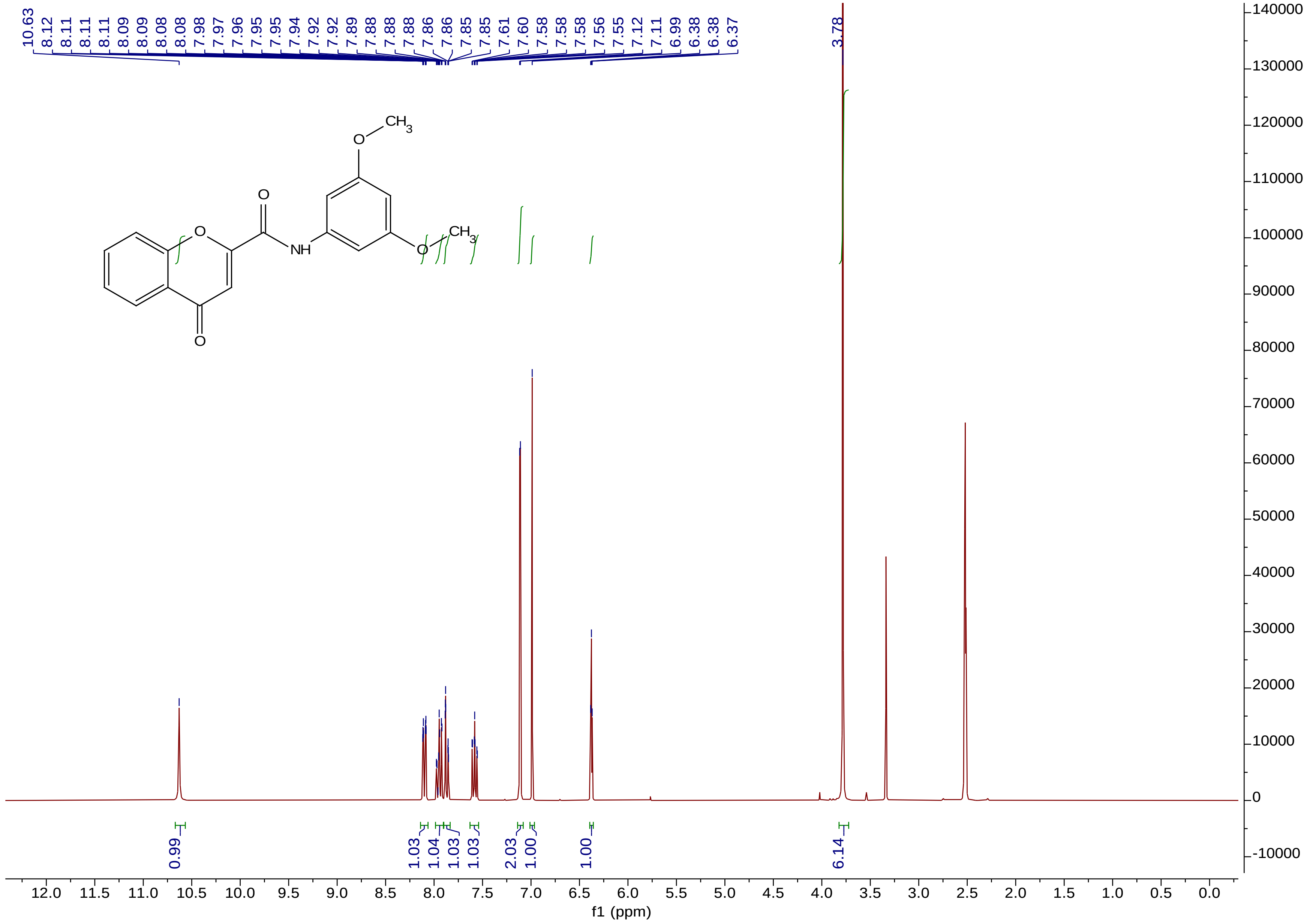

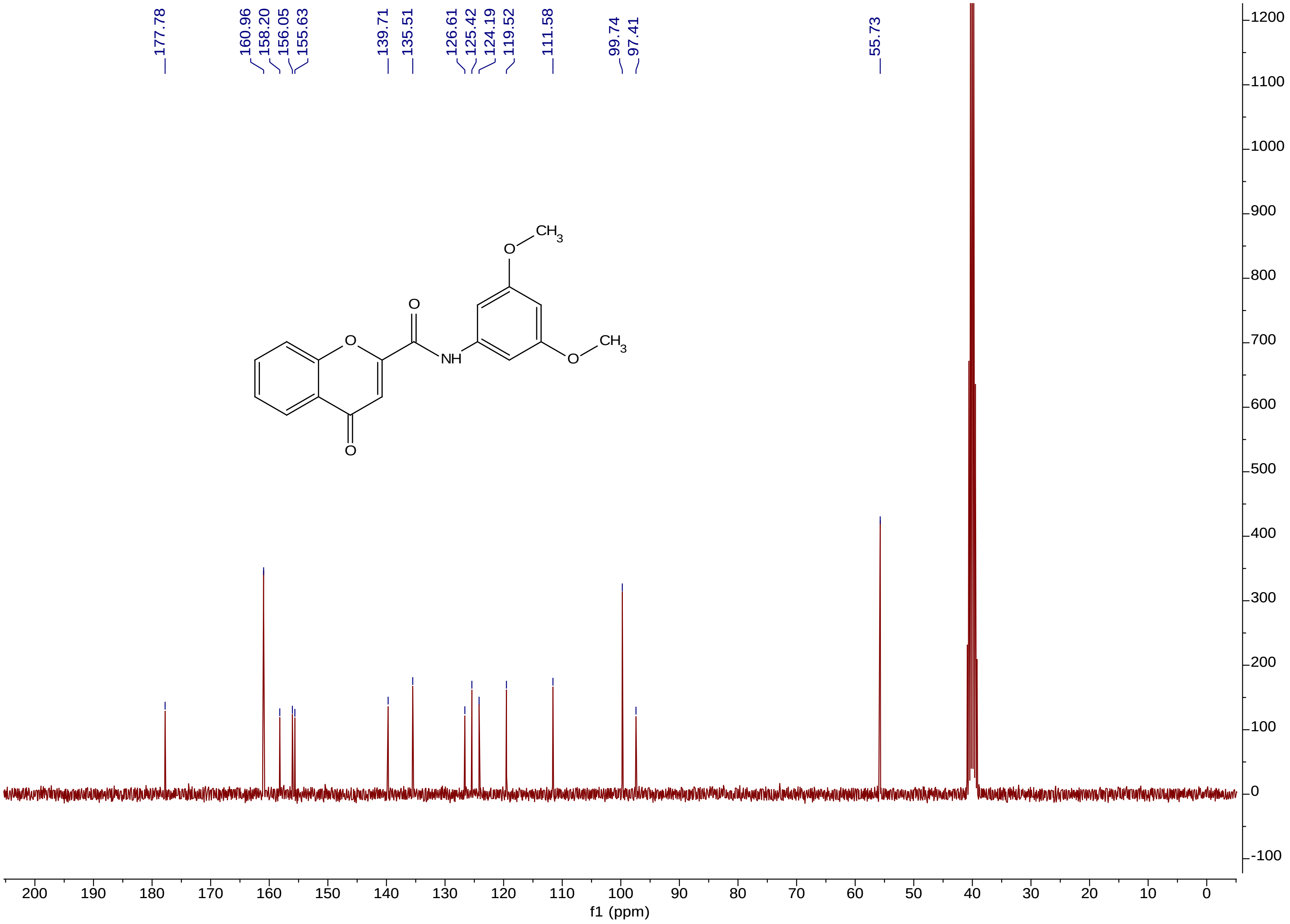

177.78
160.96
158.20
156.05
155.63
139.71
135.51
126.61
125.42
124.19
119.52
111.58
99.74
97.41
55.73
f1 (ppm)

_N_-(4-Morpholinophenyl)-4-oxo-4_H_-chromene-2-carboxamide (3u)

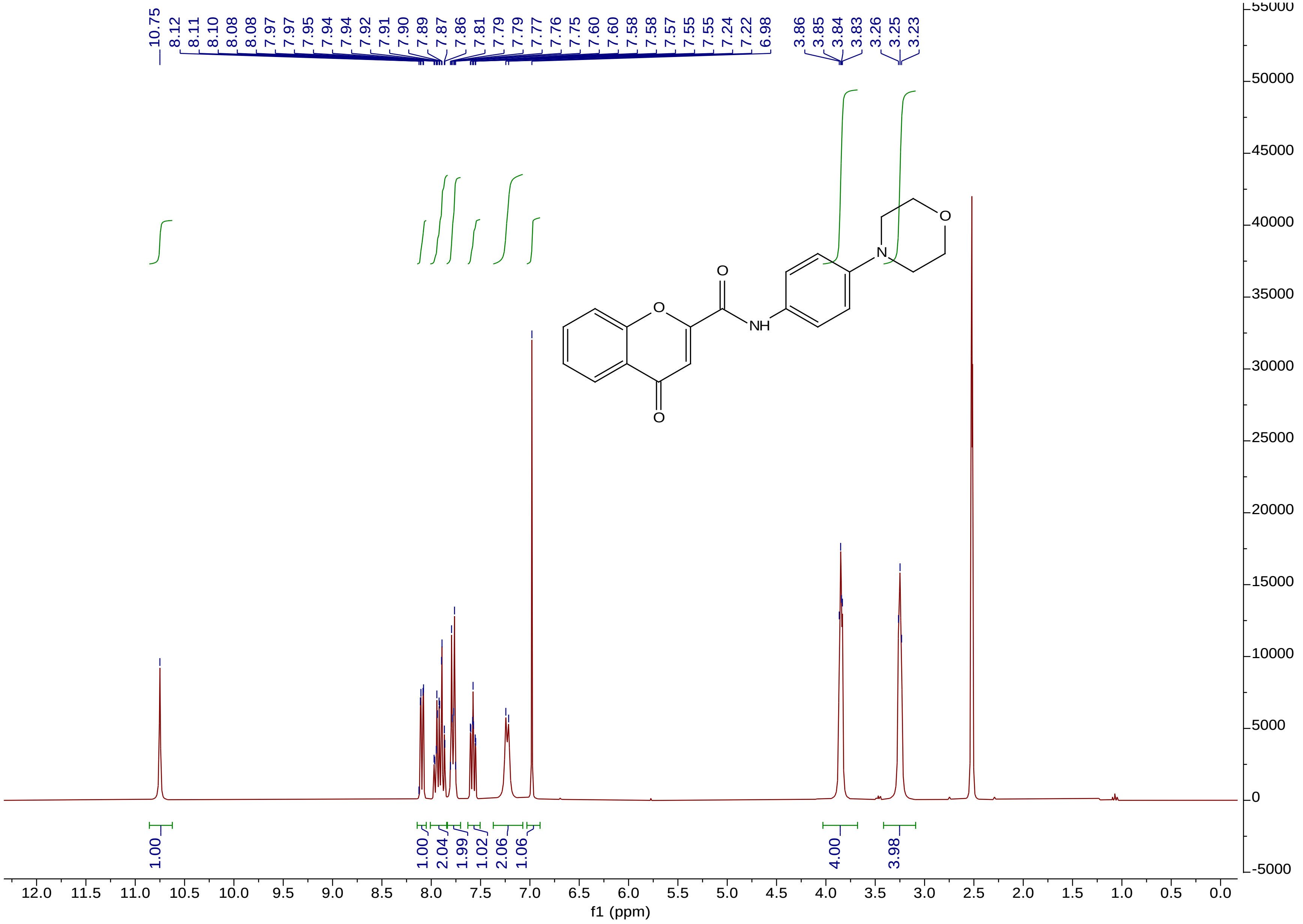

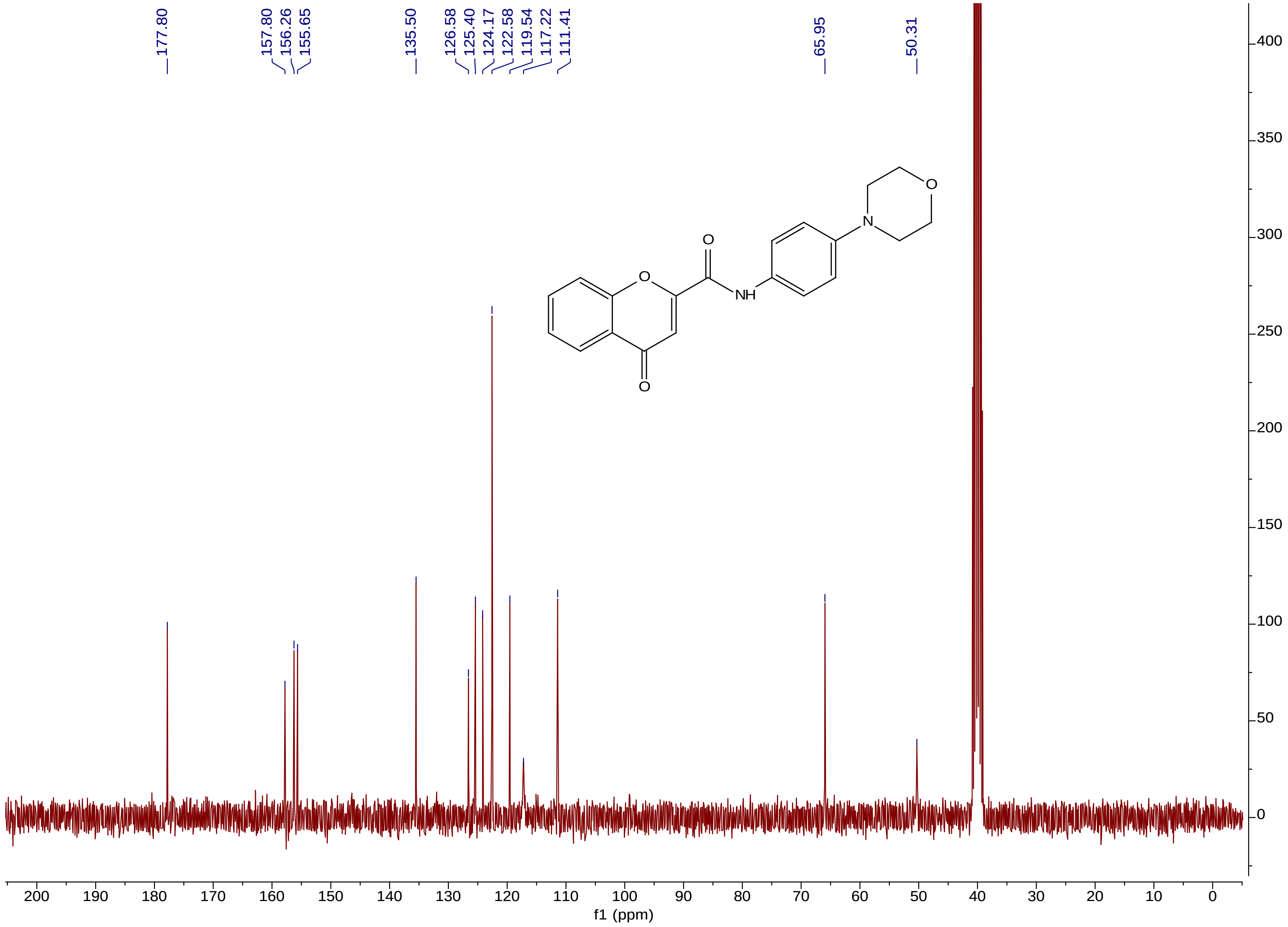

177.80
157.80
156.26
155.65
135.50
126.58
125.40
124.17
122.58
119.54
117.22
111.41
65.95
50.31
O
O
NH
O
N
O
400
350
300
250
200
150
100
50
0
200
190
180
170
160
150
140
130
120
110
100
90
80
70
60
50
40
30
20
10
0
f1 (ppm)

4-Oxo-*N*-(pyrimidin-2-yl)-4*H*-chromene-2-carboxamide (3v)

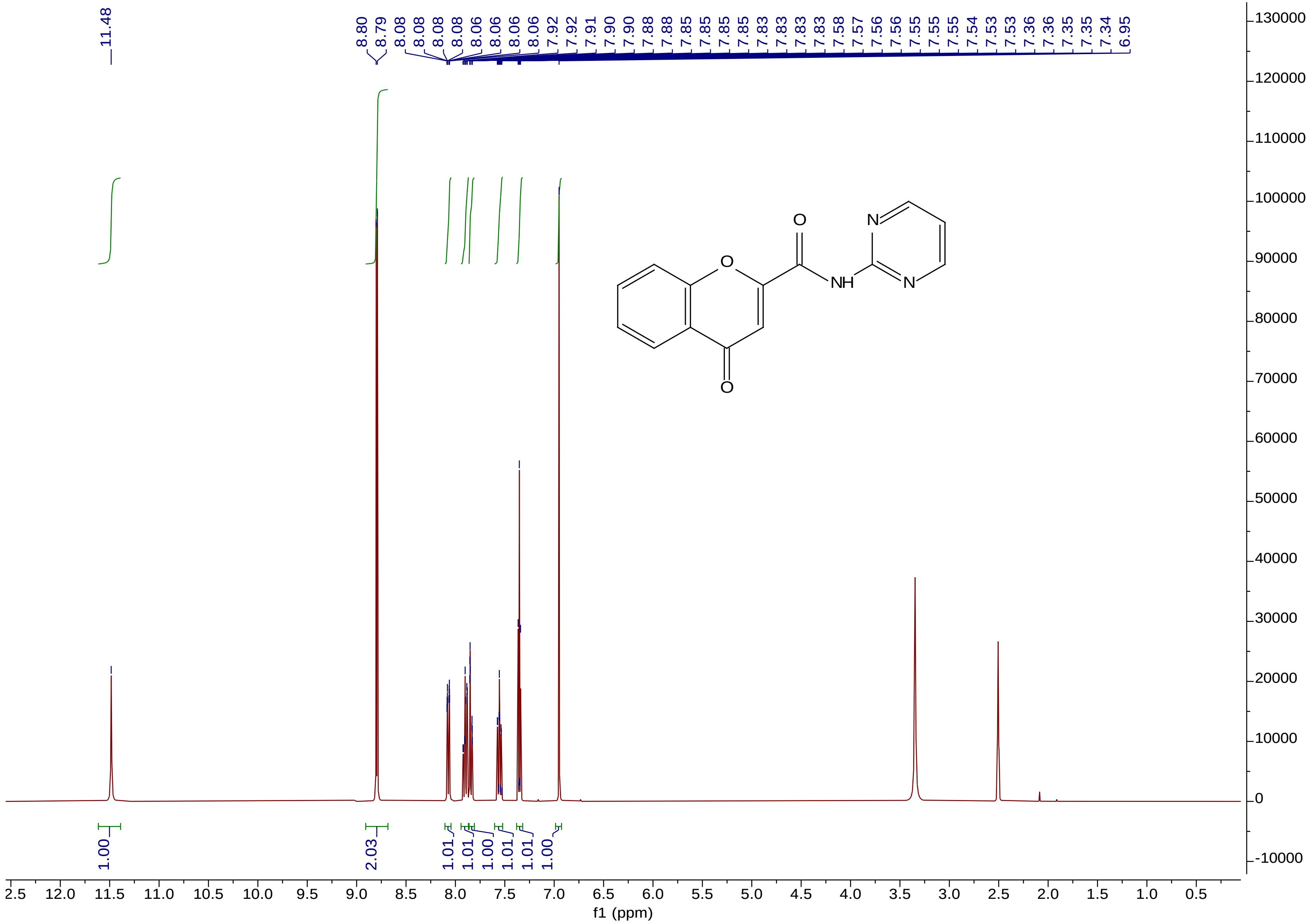

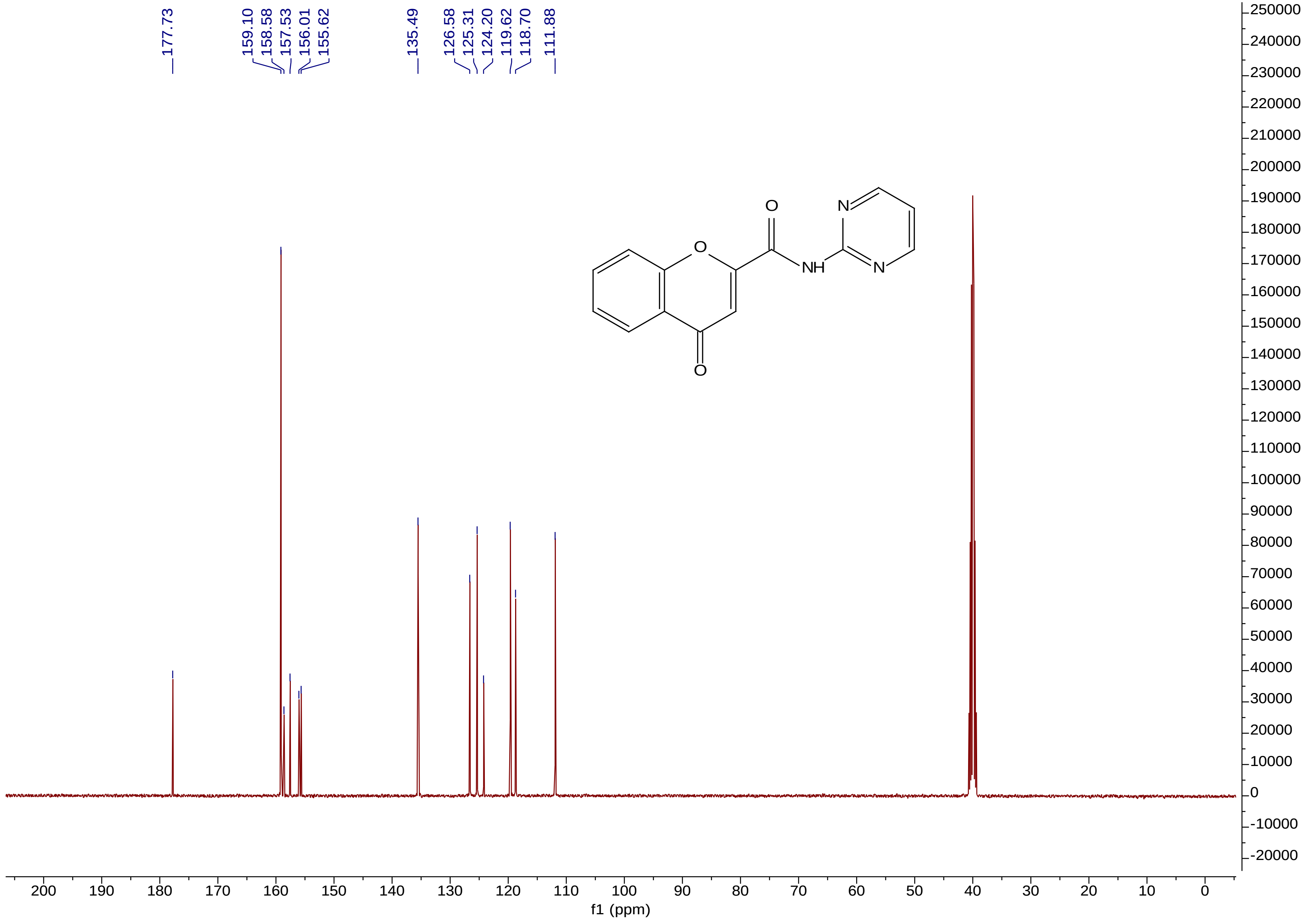
177.73
159.10
158.58
157.53
156.01
155.62
135.49
126.58
125.31
124.20
119.62
118.70
111.88
O
NH
N
f1 (ppm)

4-Oxo-*N*-propyl-4*H*-chromene-2-carboxamide (3w)

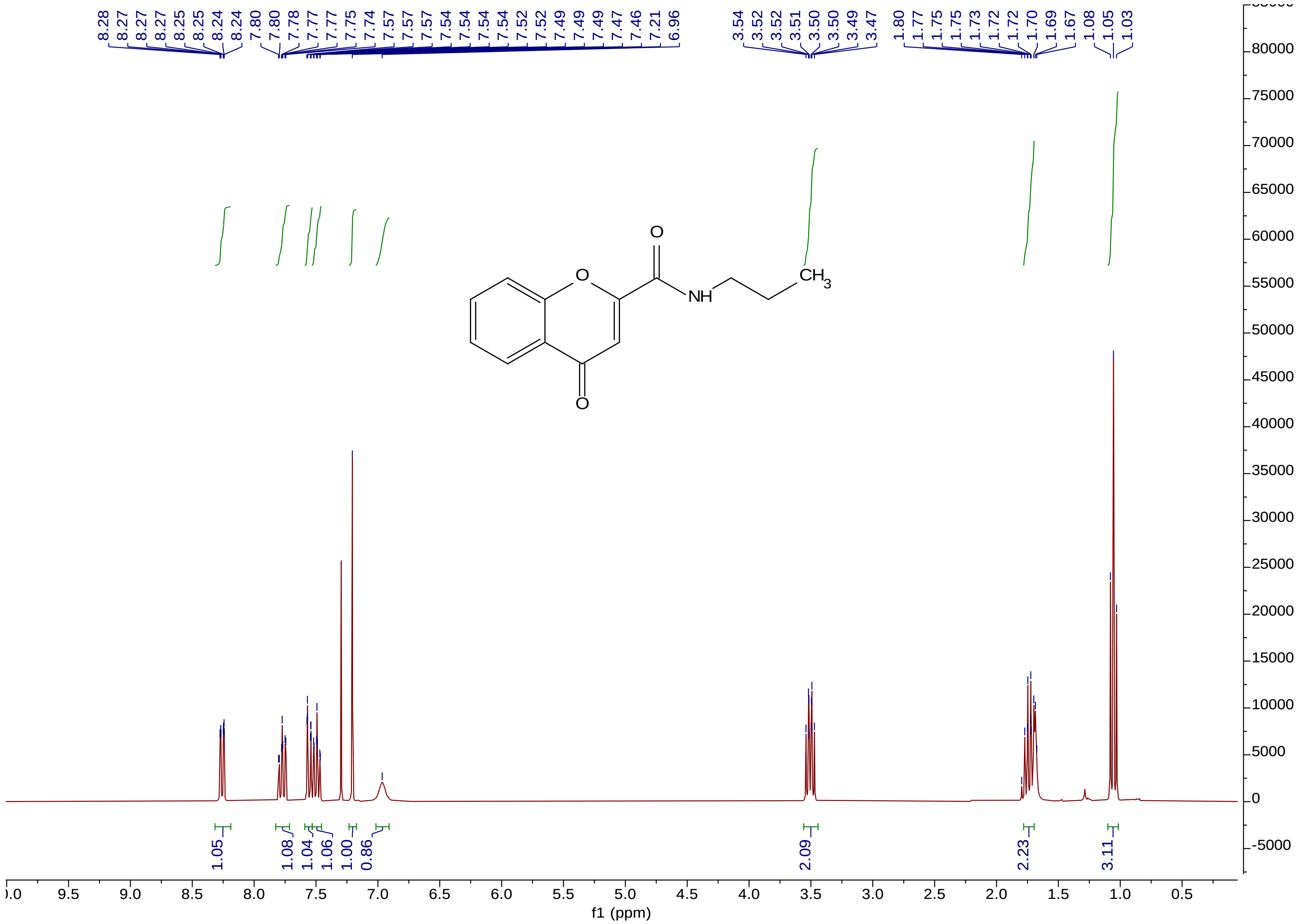

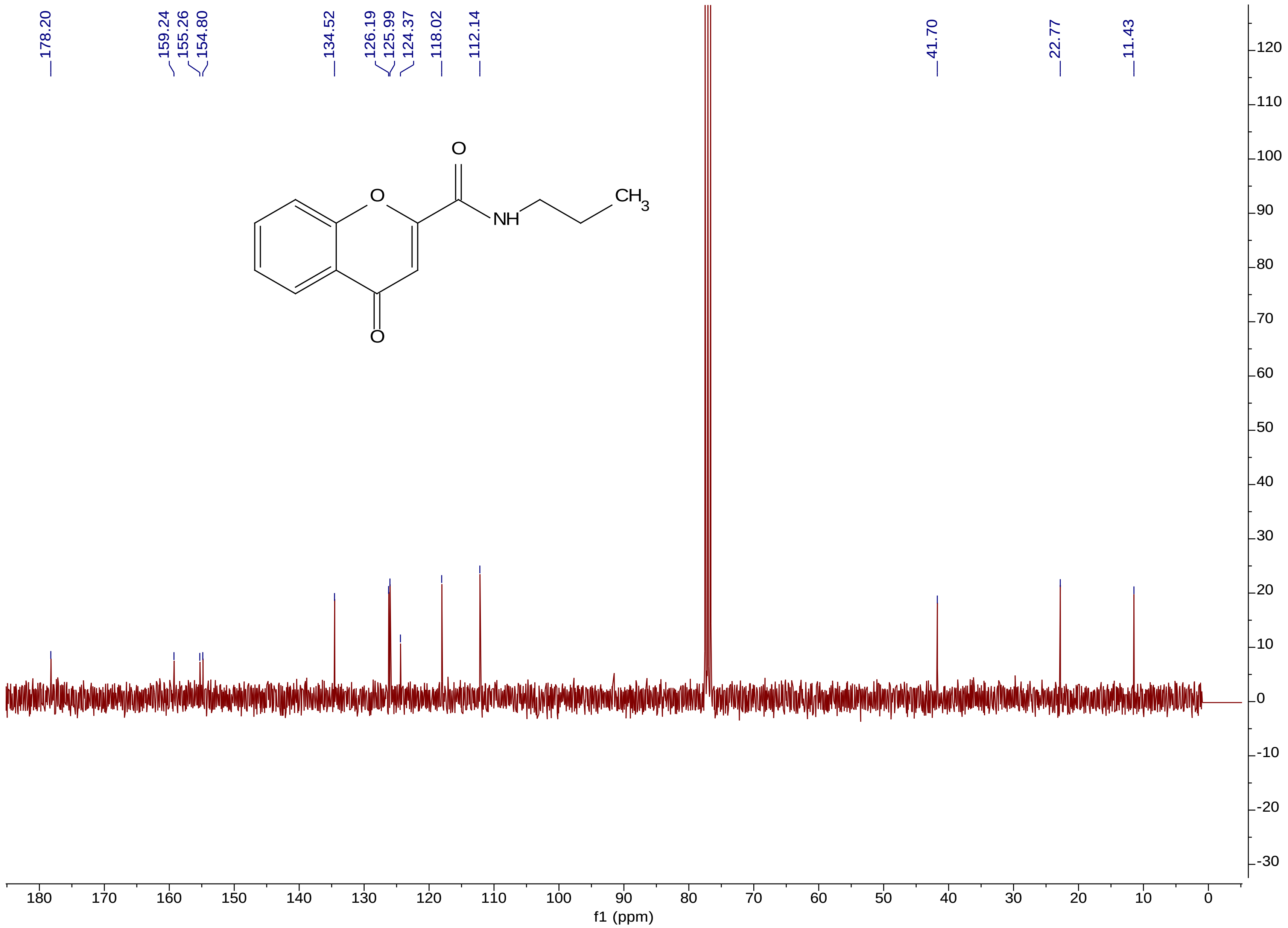
178.20
159.24
155.26
154.80
134.52
126.19
125.99
124.37
118.02
112.14
41.70
22.77
11.43
O
O
NH
CH3
O
f1 (ppm)

_N_-Cyclohexyl-4-oxo-4_H_-chromene-2-carboxamide (3x)

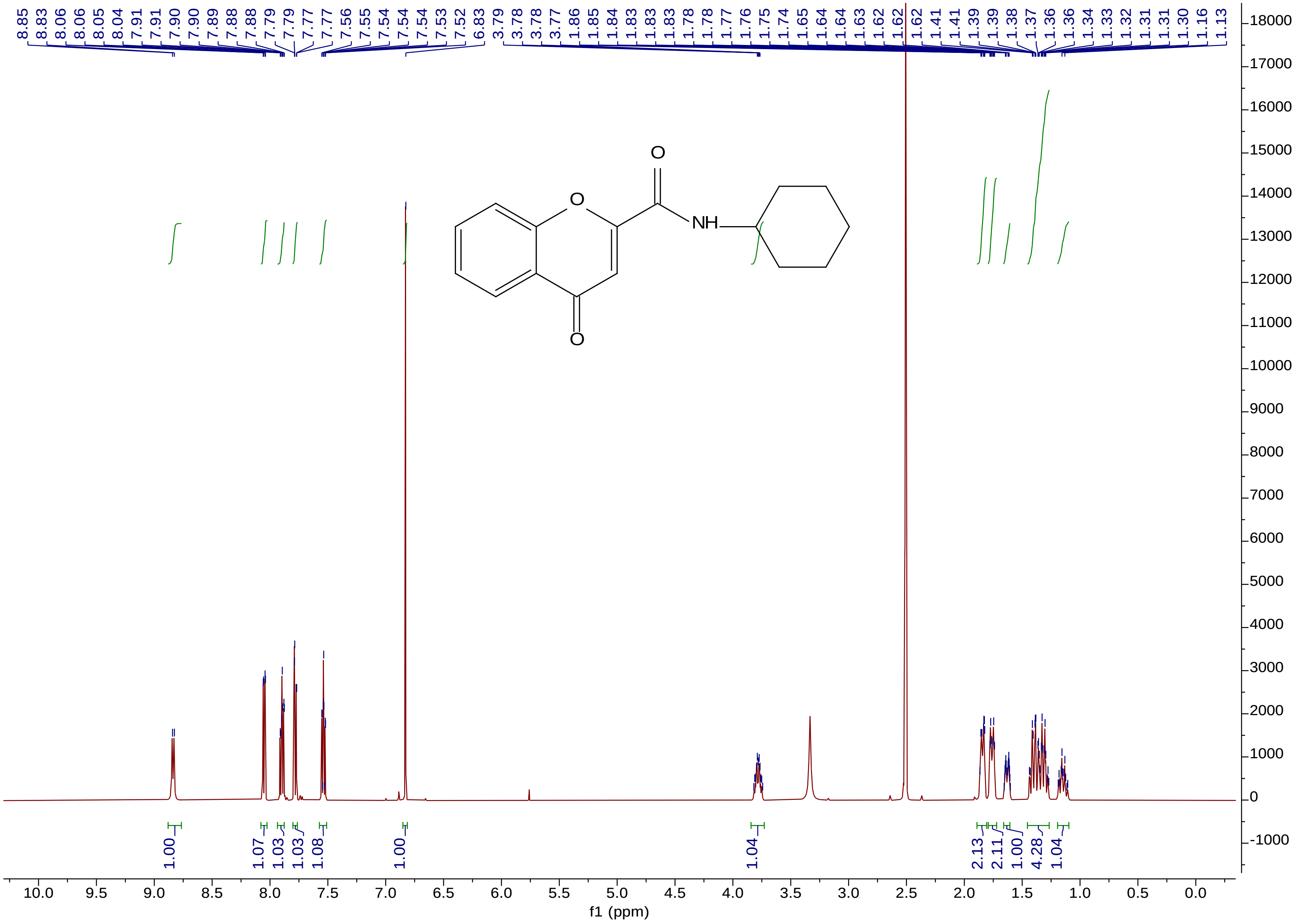

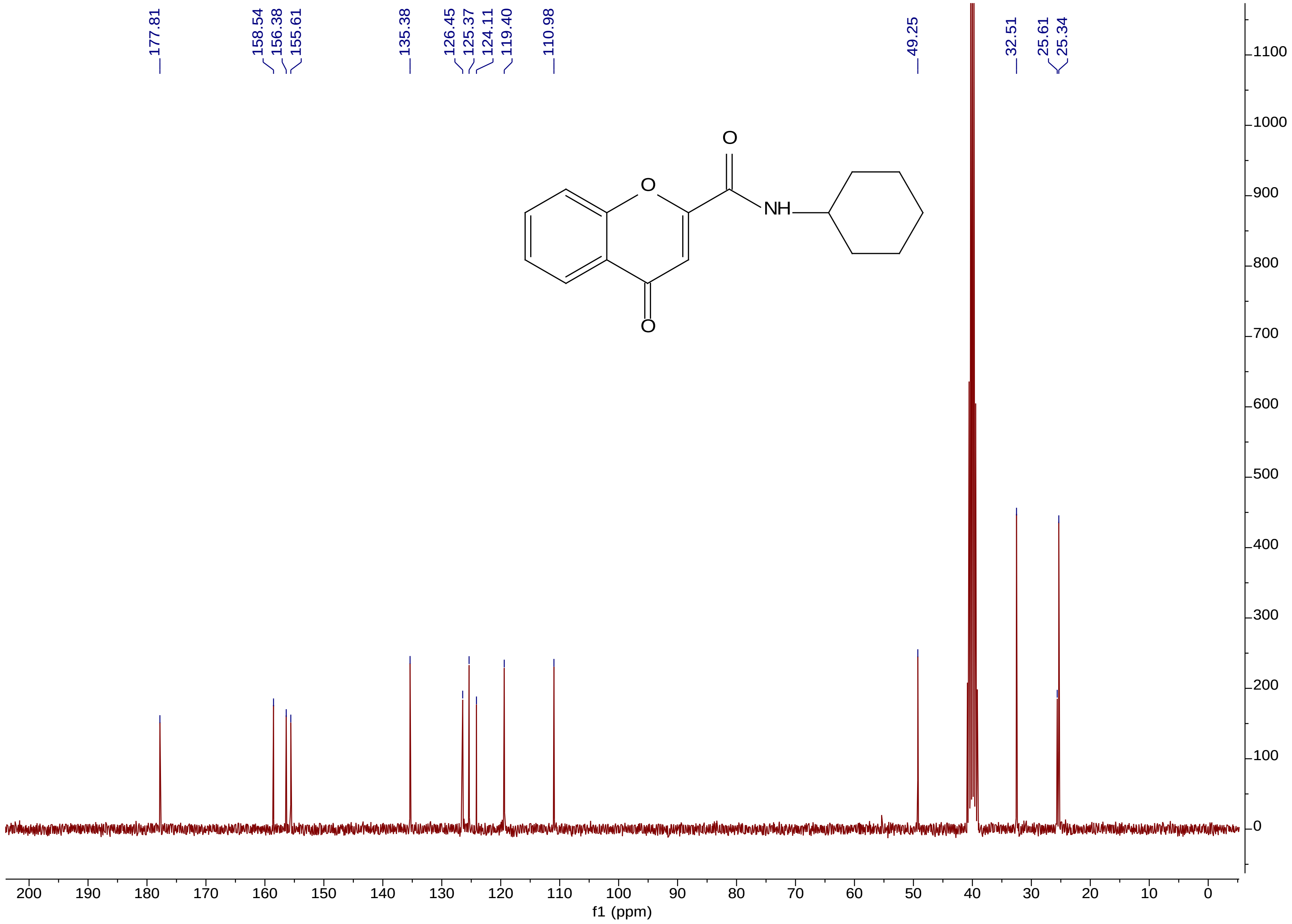
177.81
158.54
156.38
155.61
135.38
126.45
125.37
124.11
119.40
110.98
49.25
32.51
25.61
25.34
O
O
NH
O
1100
1000
900
800
700
600
500
400
300
200
100
0
200 190 180 170 160 150 140 130 120 110 100 90 80 70 60 50 40 30 20 10 0
f1 (ppm)

N-((3s,5s,7s)-Adamantan-1-yl)-4-oxo-4H-chromene-2-carboxamide (3y)

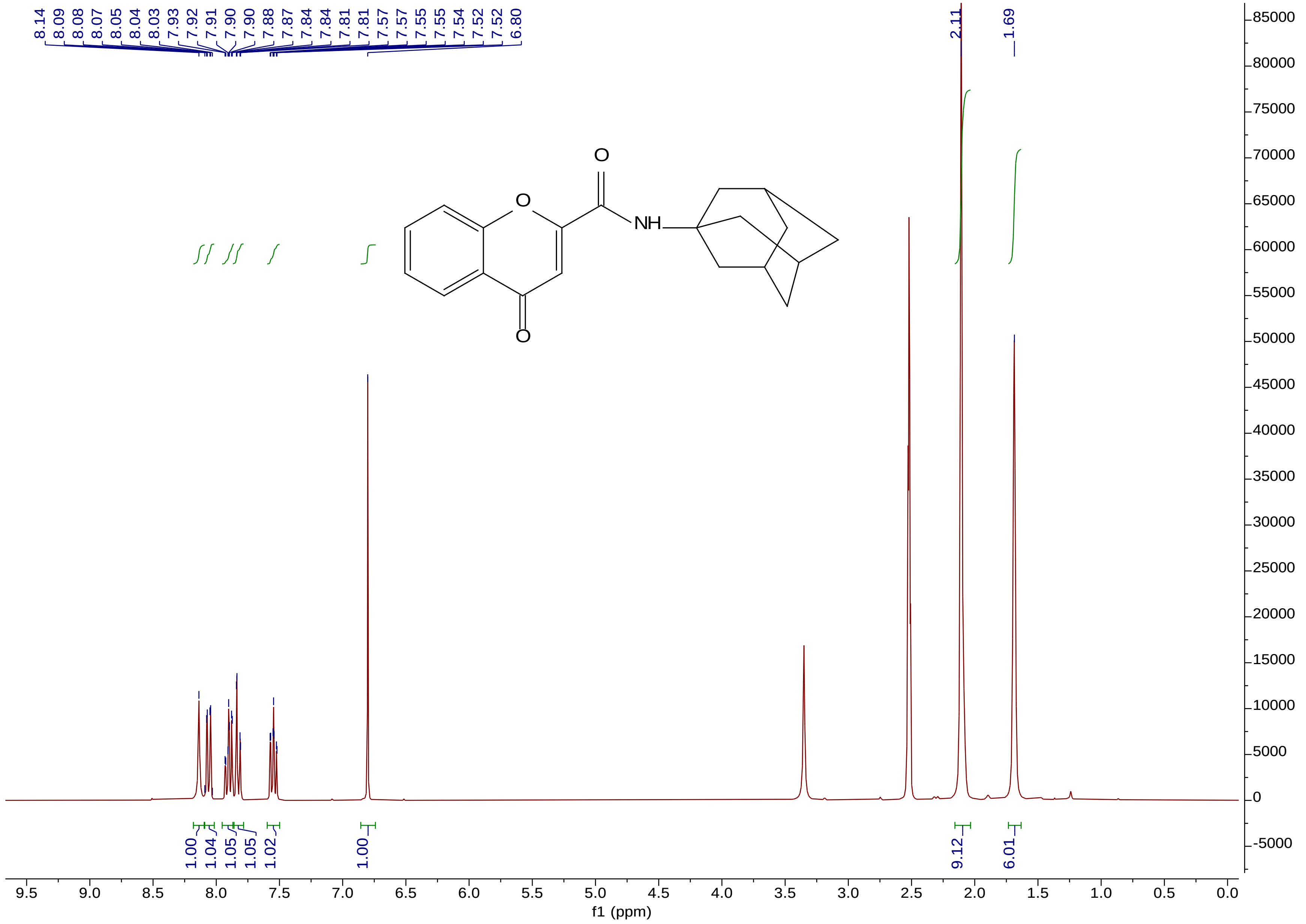

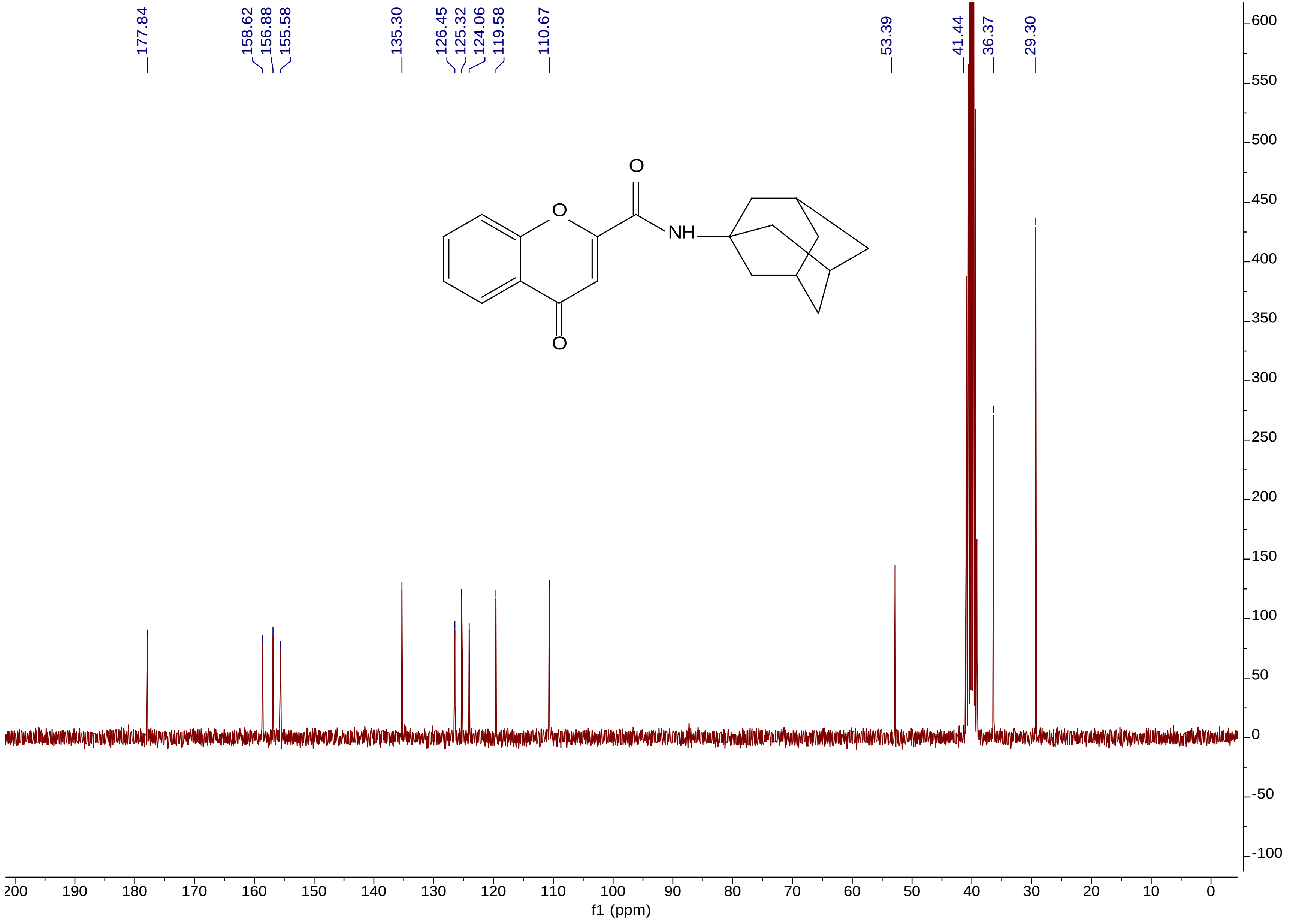

177.84
158.62
156.88
155.58
135.30
126.45
125.32
124.06
119.58
110.67
53.39
41.44
36.37
29.30
O
O
NH
O
f1 (ppm)

4-Oxo-*N*-(3-(pyrrolidin-1-yl)propyl)-4*H*-chromene-2-carboxamide (3z)

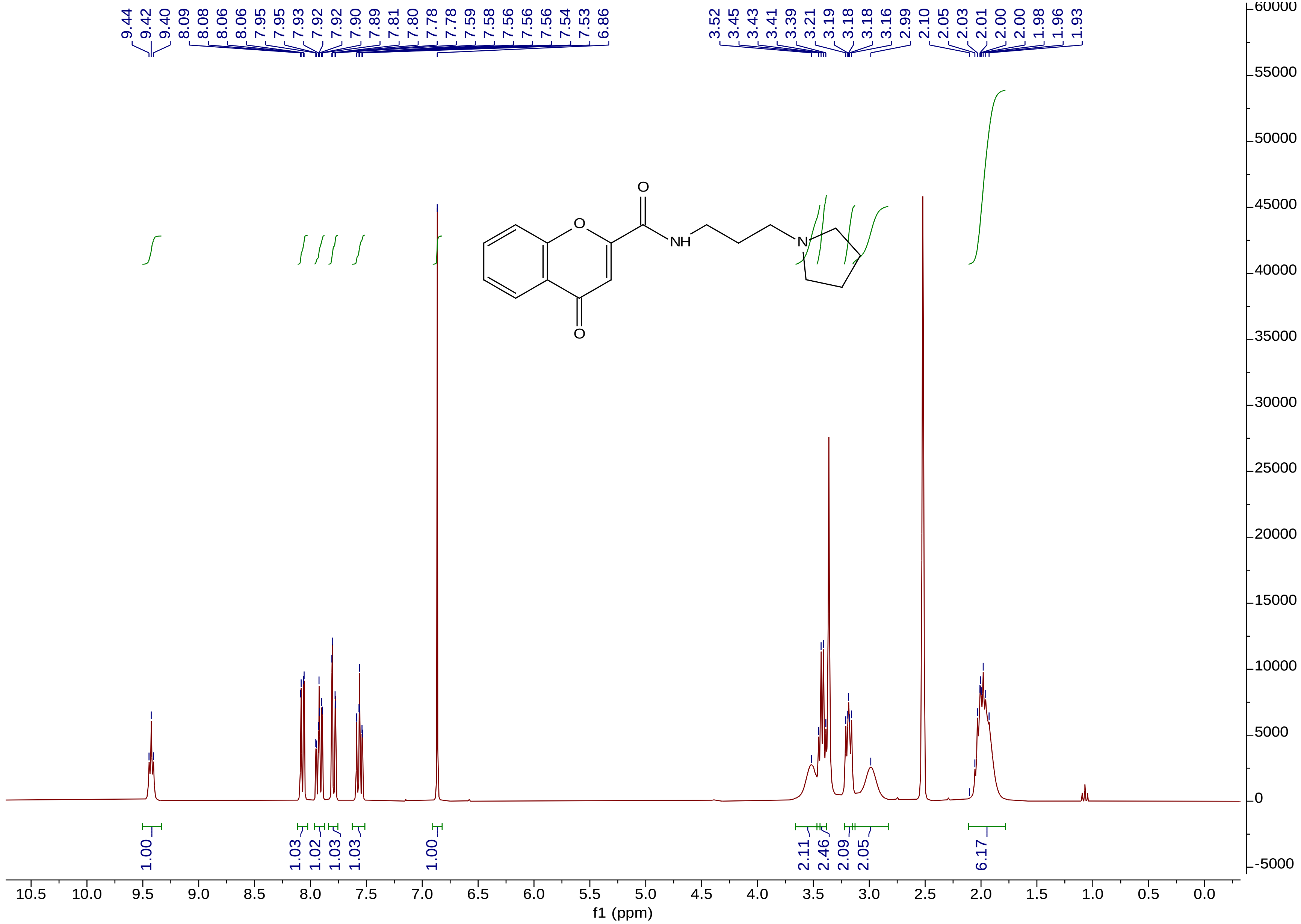

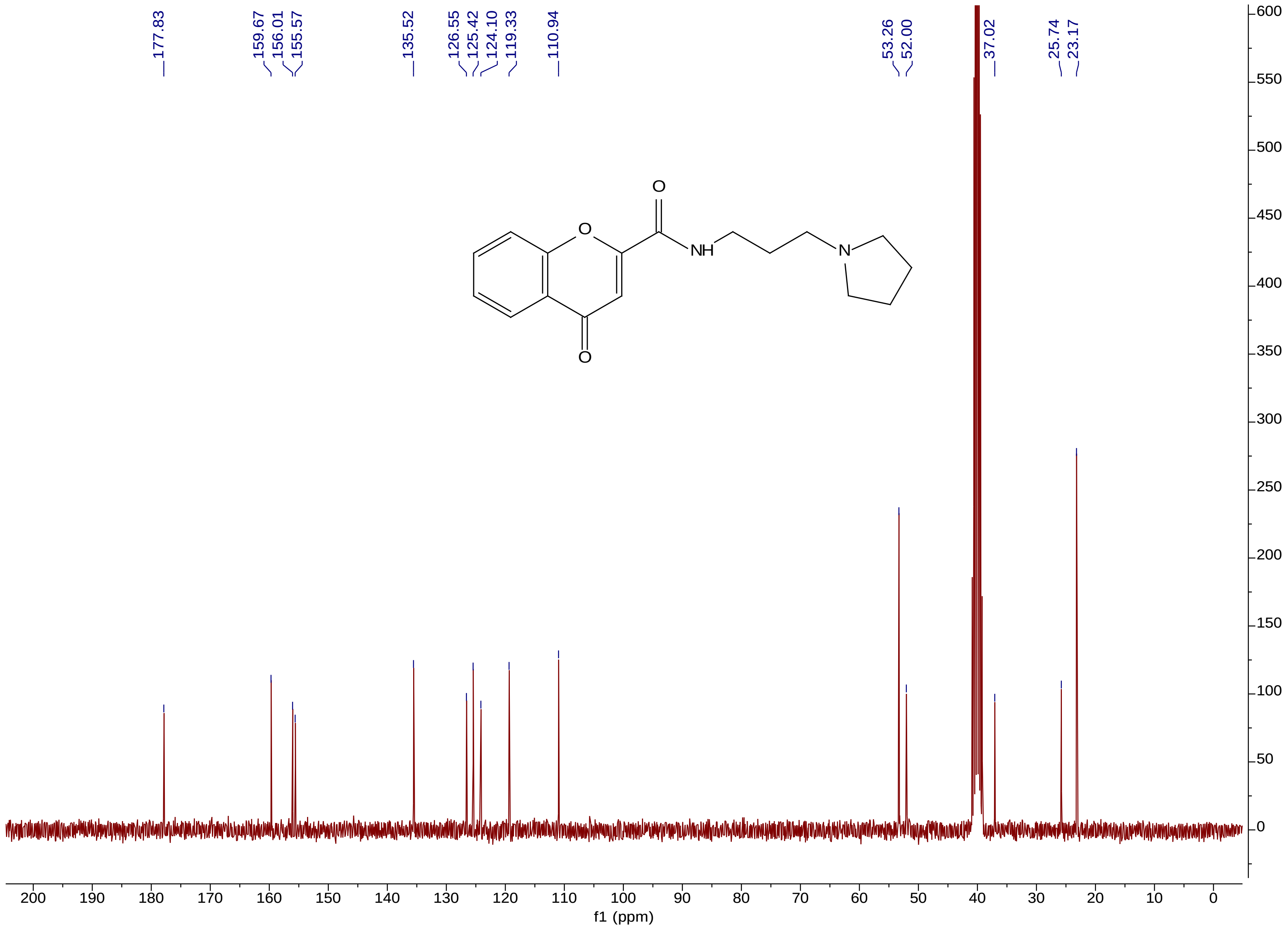

177.83
159.67
156.01
155.57
135.52
126.55
125.42
124.10
119.33
110.94
53.26
52.00
37.02
25.74
23.17
O
O
NH
N
O
600
550
500
450
400
350
300
250
200
150
100
50
0
200 190 180 170 160 150 140 130 120 110 100 90 80 70 60 50 40 30 20 10 0
f1 (ppm)

N-(3-Ethylphenyl)-4-oxo-4H-chromene-2-carboxamide (3’a)

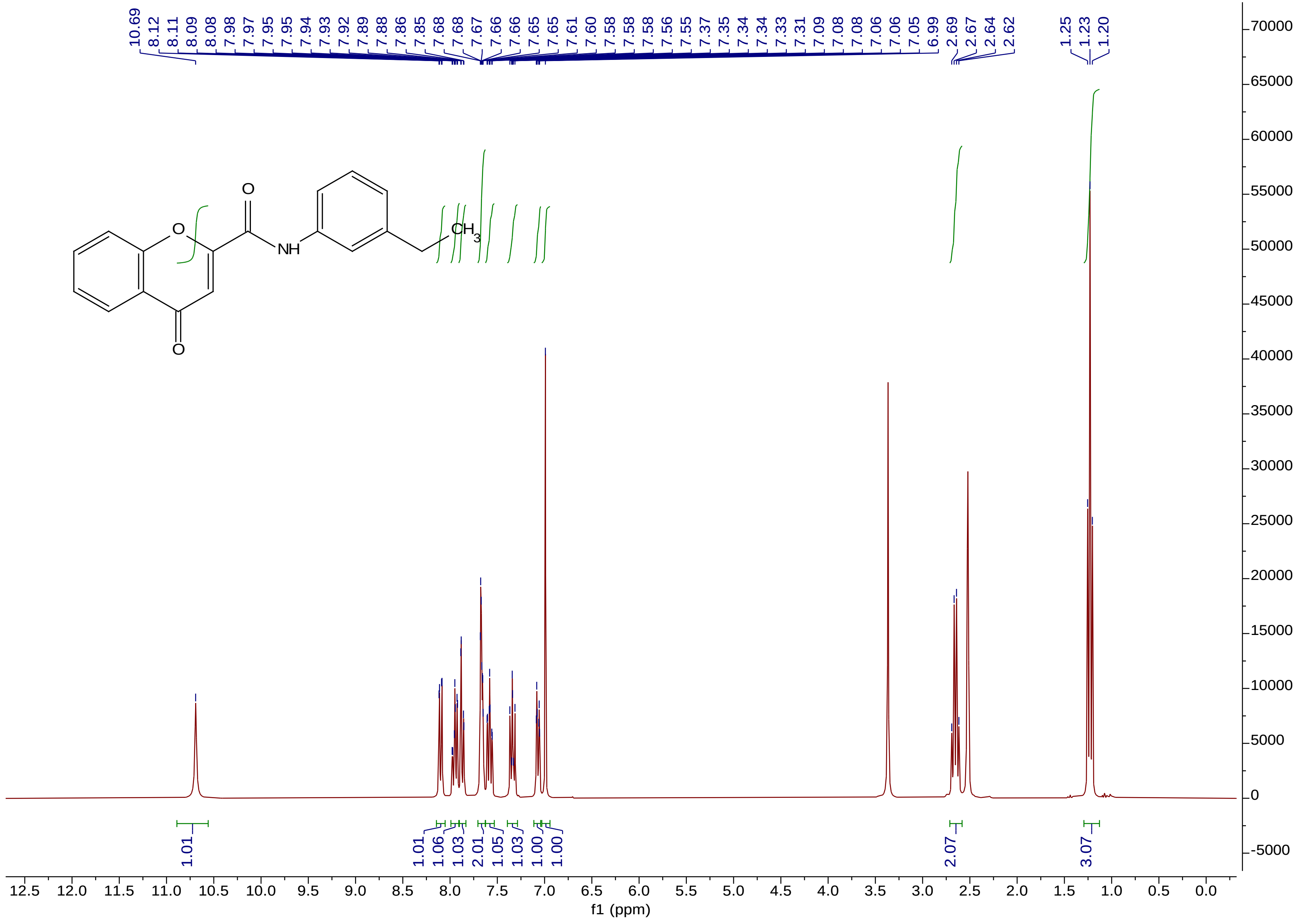

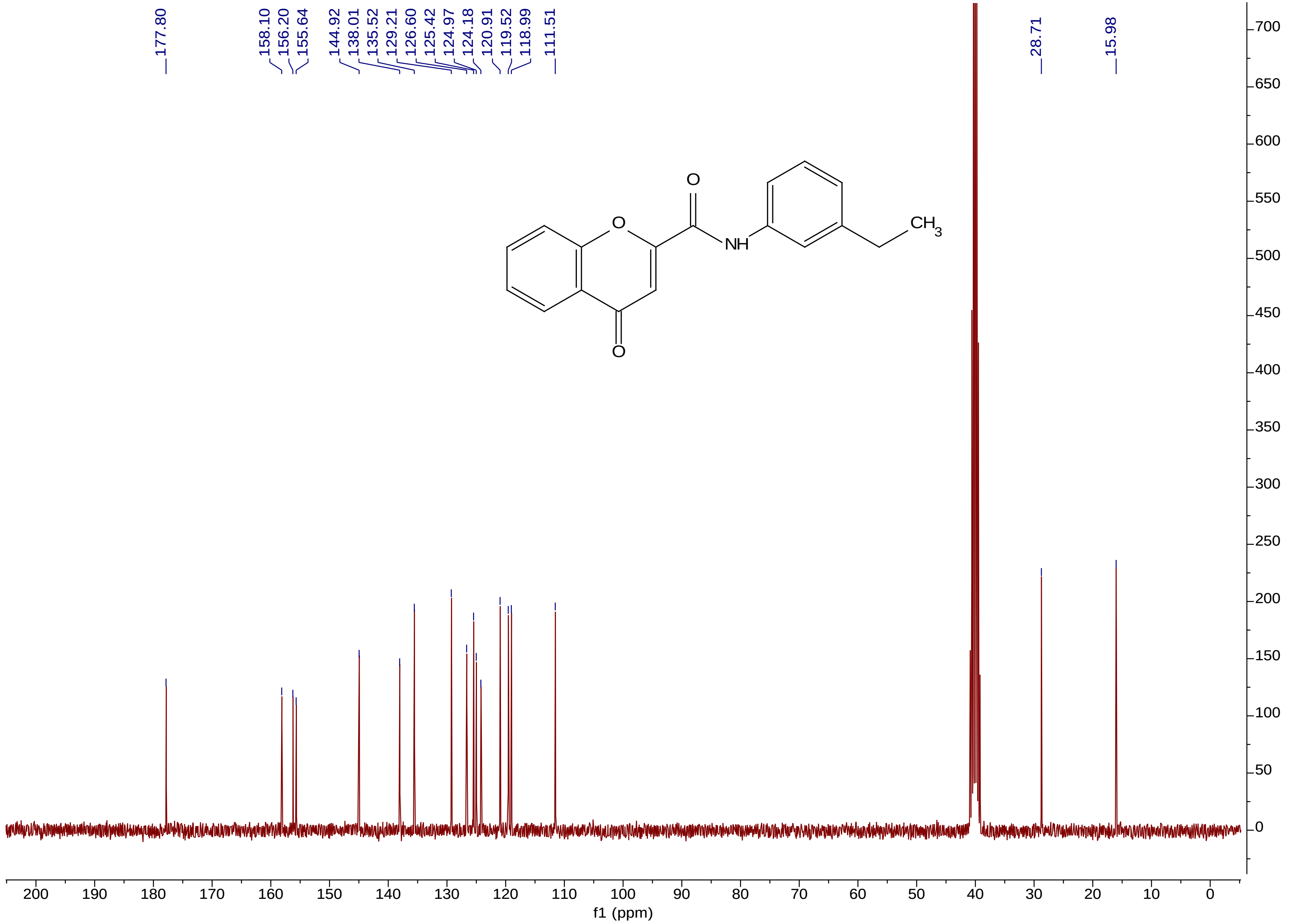
177.80
158.10
156.20
155.64
144.92
138.01
135.52
129.21
126.60
125.42
124.97
124.18
120.91
119.52
118.99
111.51
28.71
15.98
O
O
NH
CH3
O
700
650
600
550
500
450
400
350
300
250
200
150
100
50
0
200 190 180 170 160 150 140 130 120 110 100 90 80 70 60 50 40 30 20 10 0
f1 (ppm)

N-(3-((Diethylamino)methyl)-4-hydroxyphenyl)-4-oxo-4H-chromene-2-carboxamide (3'b)

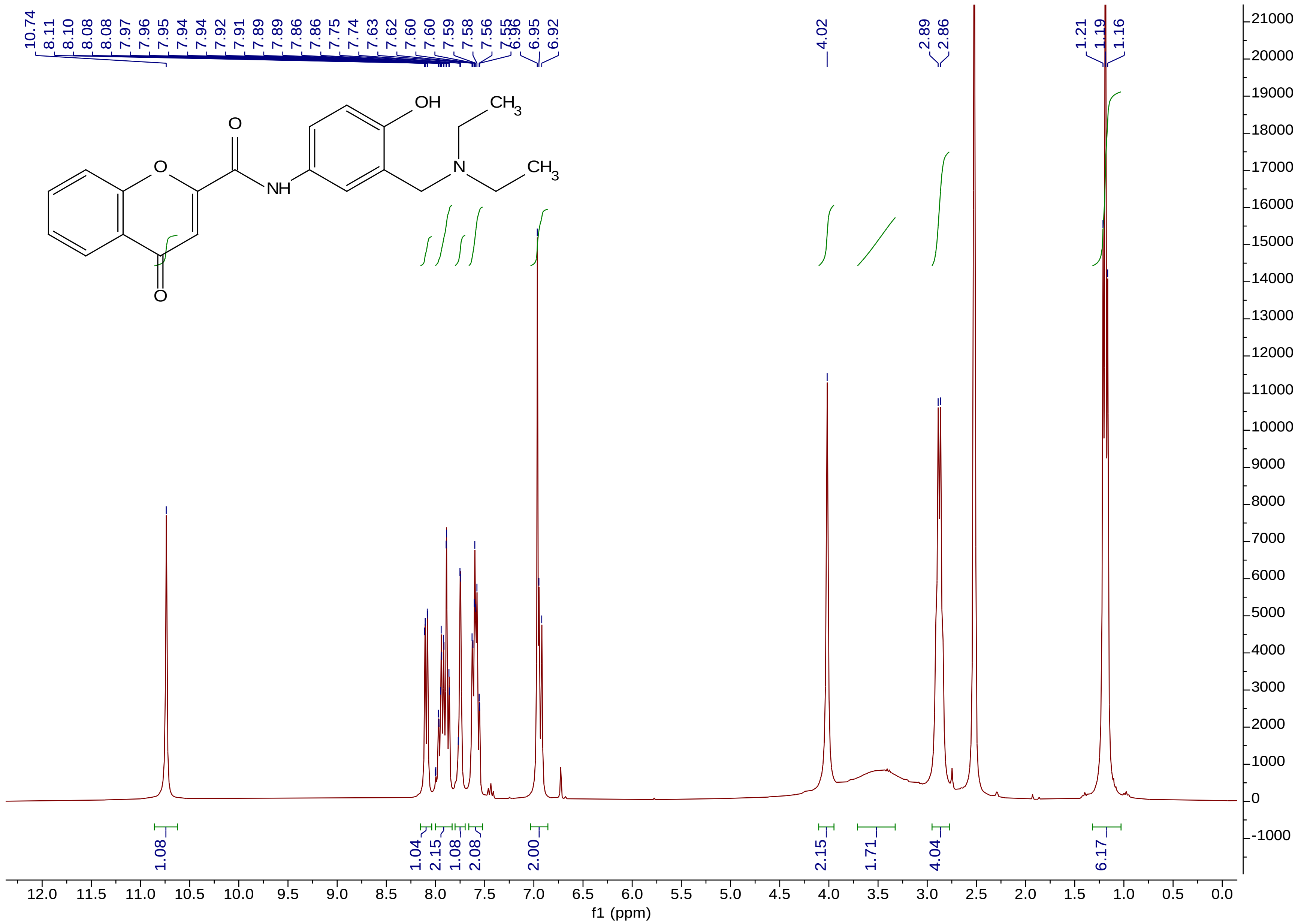

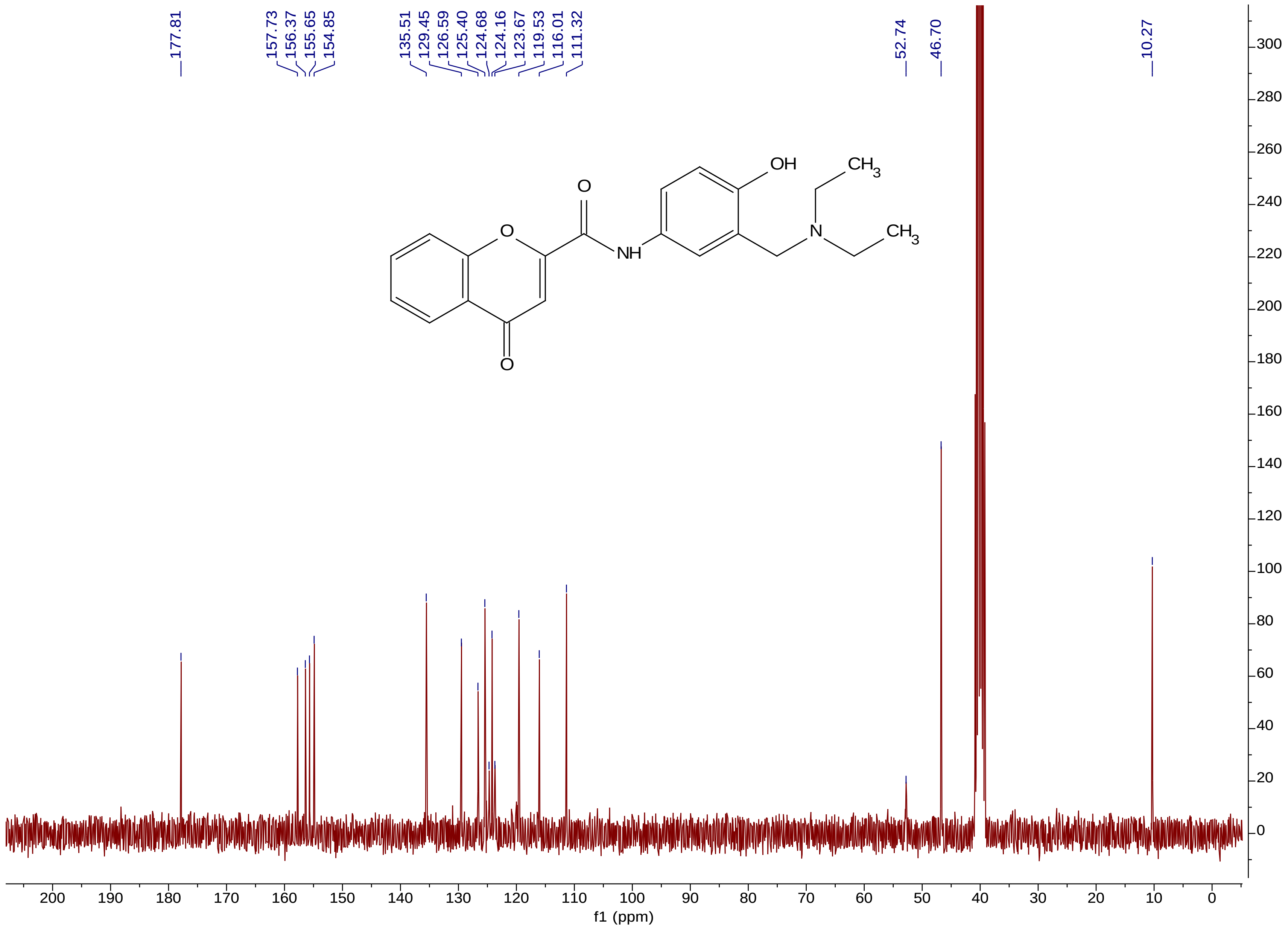
177.81
157.73
156.37
155.65
154.85
135.51
129.45
126.59
125.40
124.68
124.16
123.67
119.53
116.01
111.32
52.74
46.70
10.27
O
OH
CH3
O
N
CH3
NH
O
f1 (ppm)

_N_-(4-((1_H_-Imidazol-1-yl)methyl)phenyl)-4-oxo-4_H_-chromene-2-carboxamide (3'c)

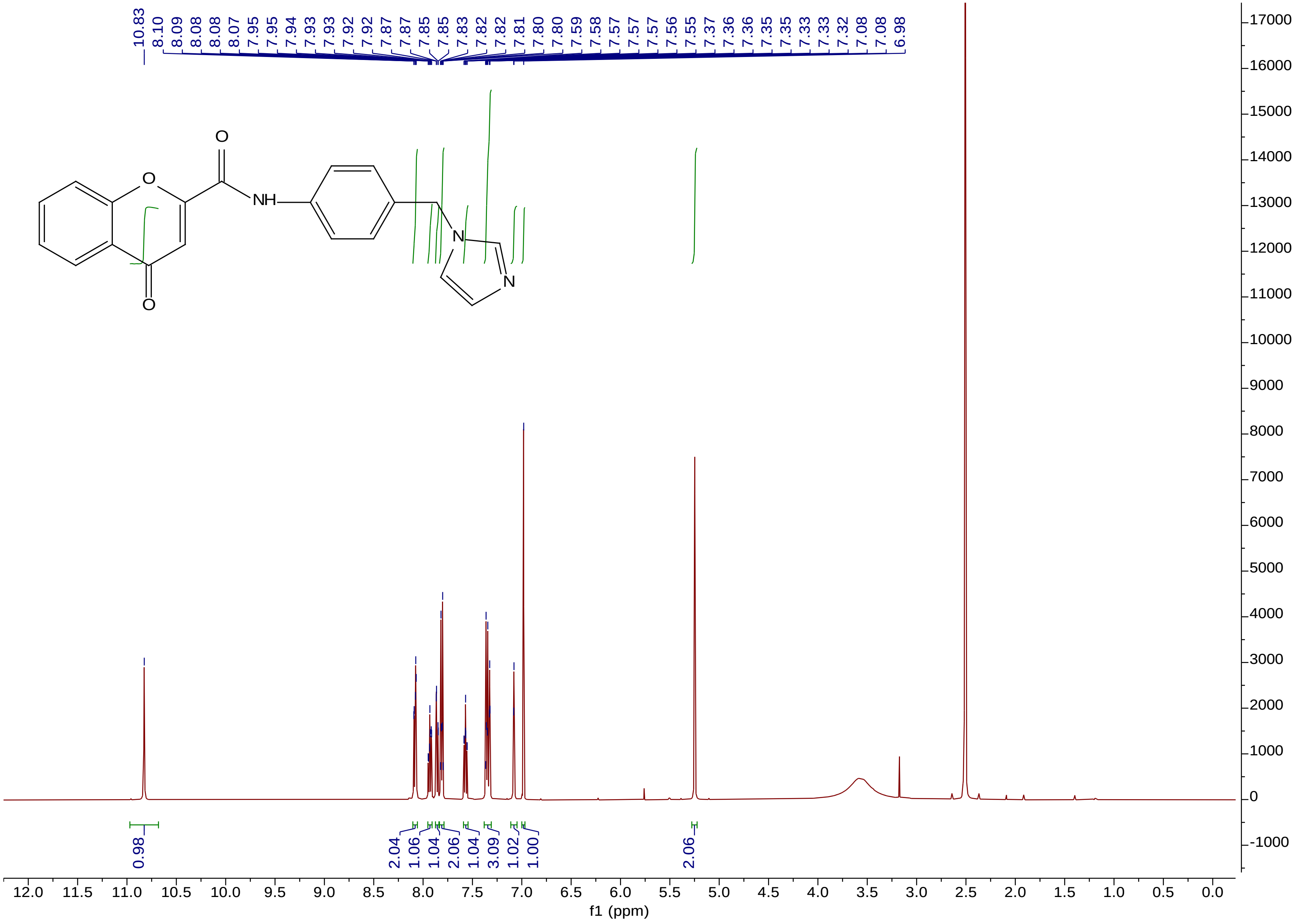

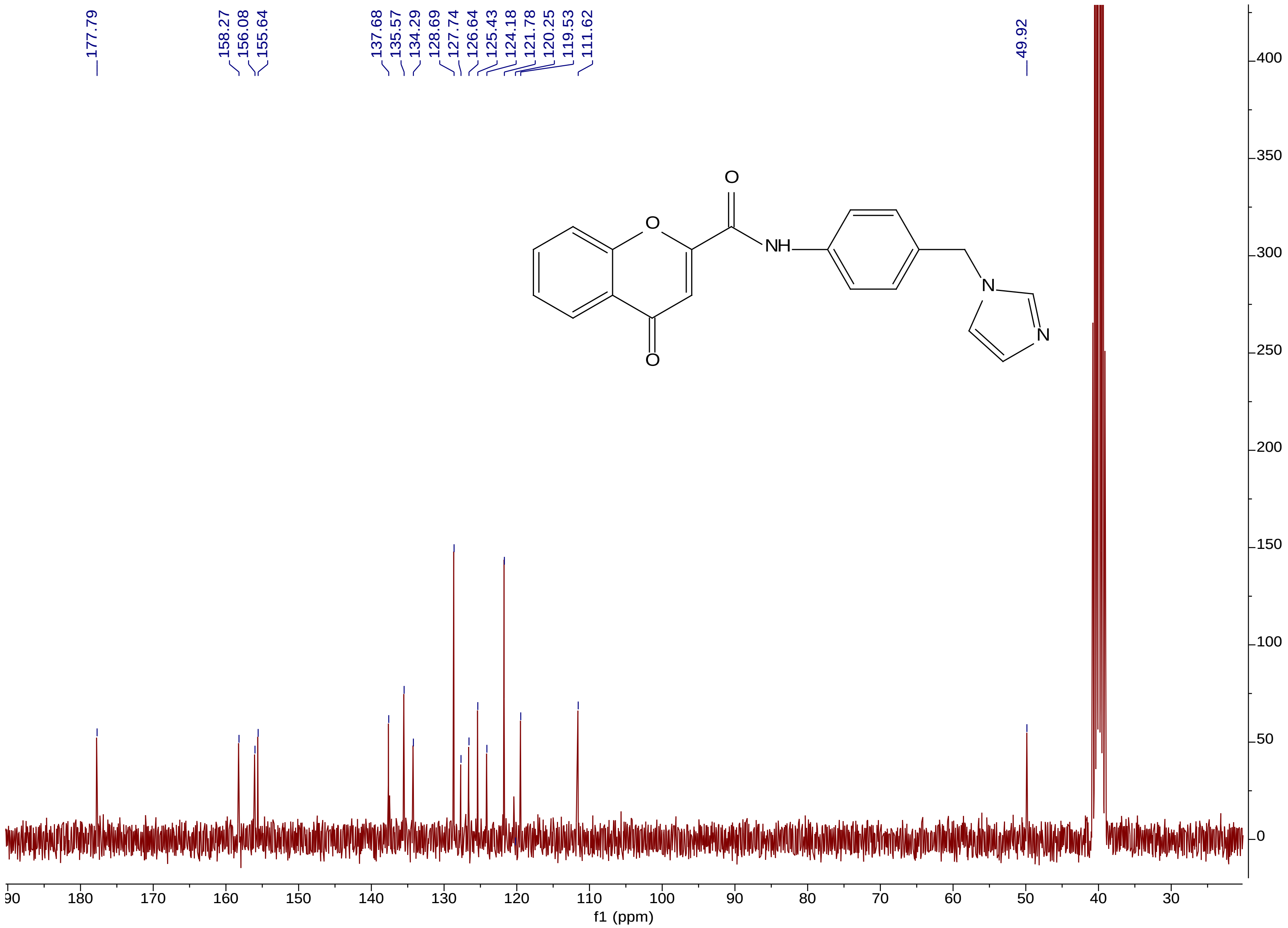
177.79
158.27
156.08
155.64
137.68
135.57
134.29
128.69
127.74
126.64
125.43
124.18
121.78
120.25
119.53
111.62
49.92
O
O
NH
N
N
O
f1 (ppm)

2-(4-(2-Oxo-2-(pyrrolidin-1-yl)ethyl)piperazine-1-carbonyl)-4*H*-chromen-4-one (3’d)

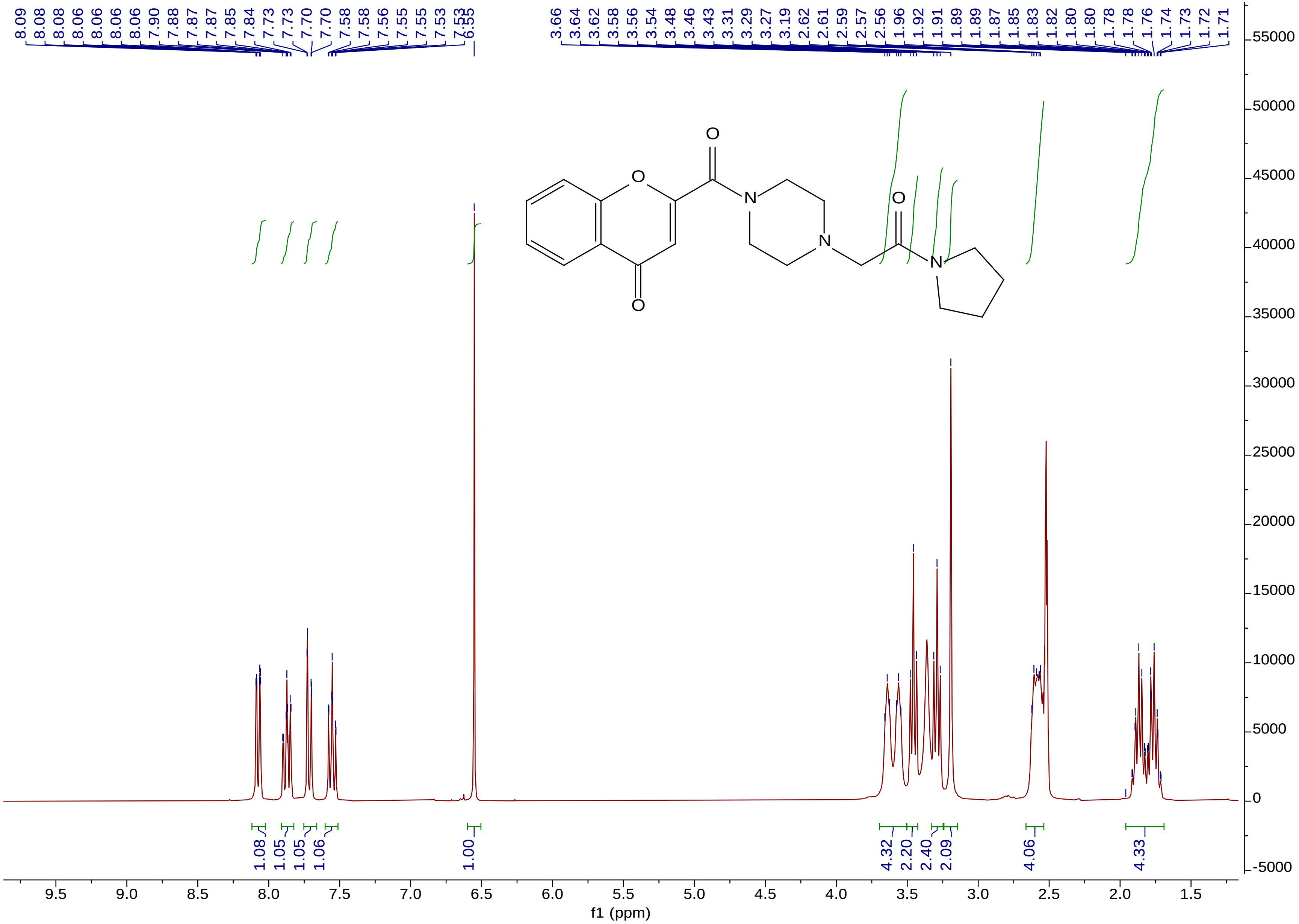

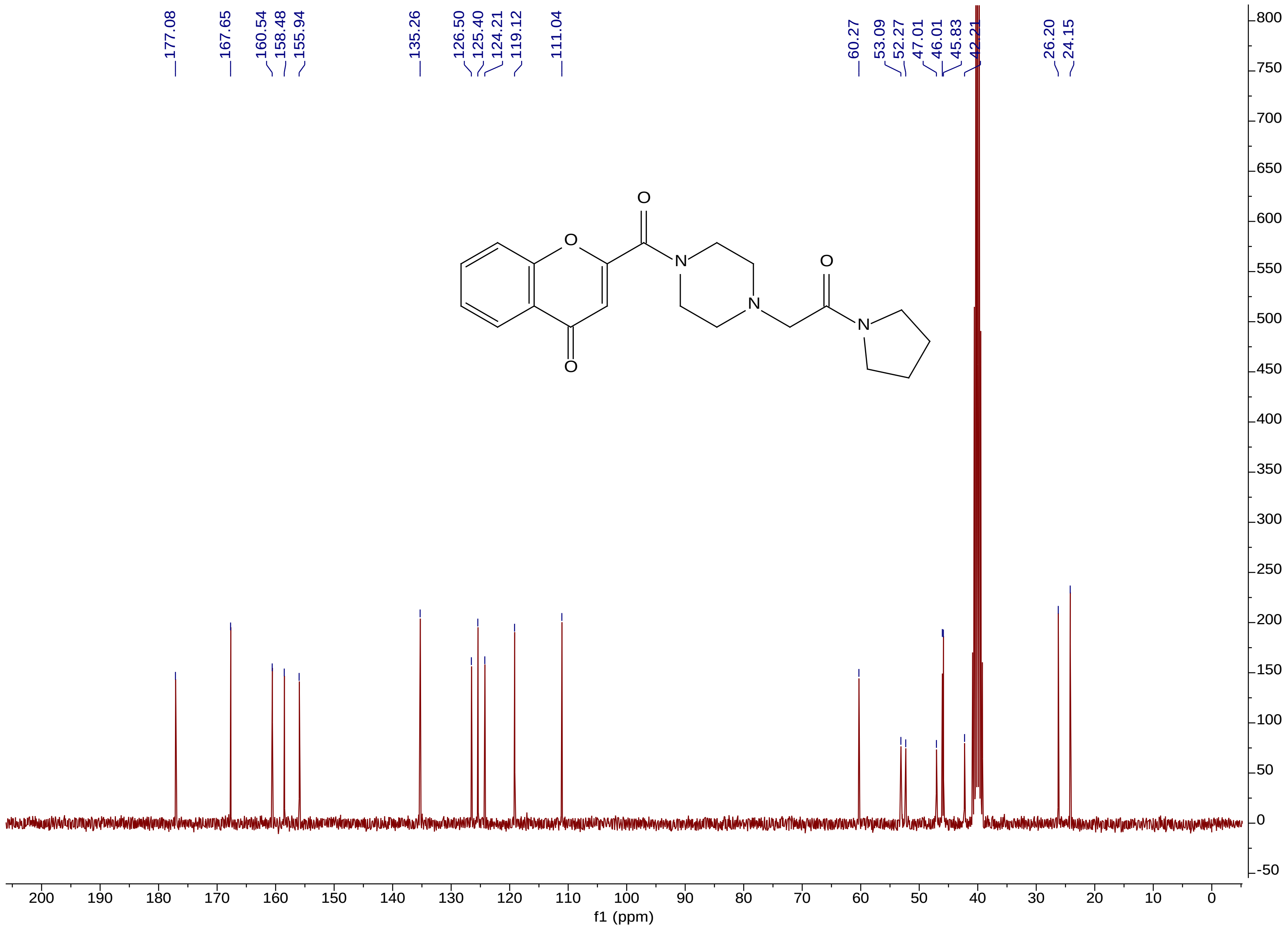

177.08
167.65
160.54
158.48
155.94
135.26
126.50
125.40
124.21
119.12
111.04
60.27
53.09
52.27
47.01
46.01
45.83
42.21
26.20
24.15
f1 (ppm)

tert-Butyl 4-((4-oxo-4*H*-chromene-2-carboxamido)methyl)piperidine-1-carboxylate (3’e)

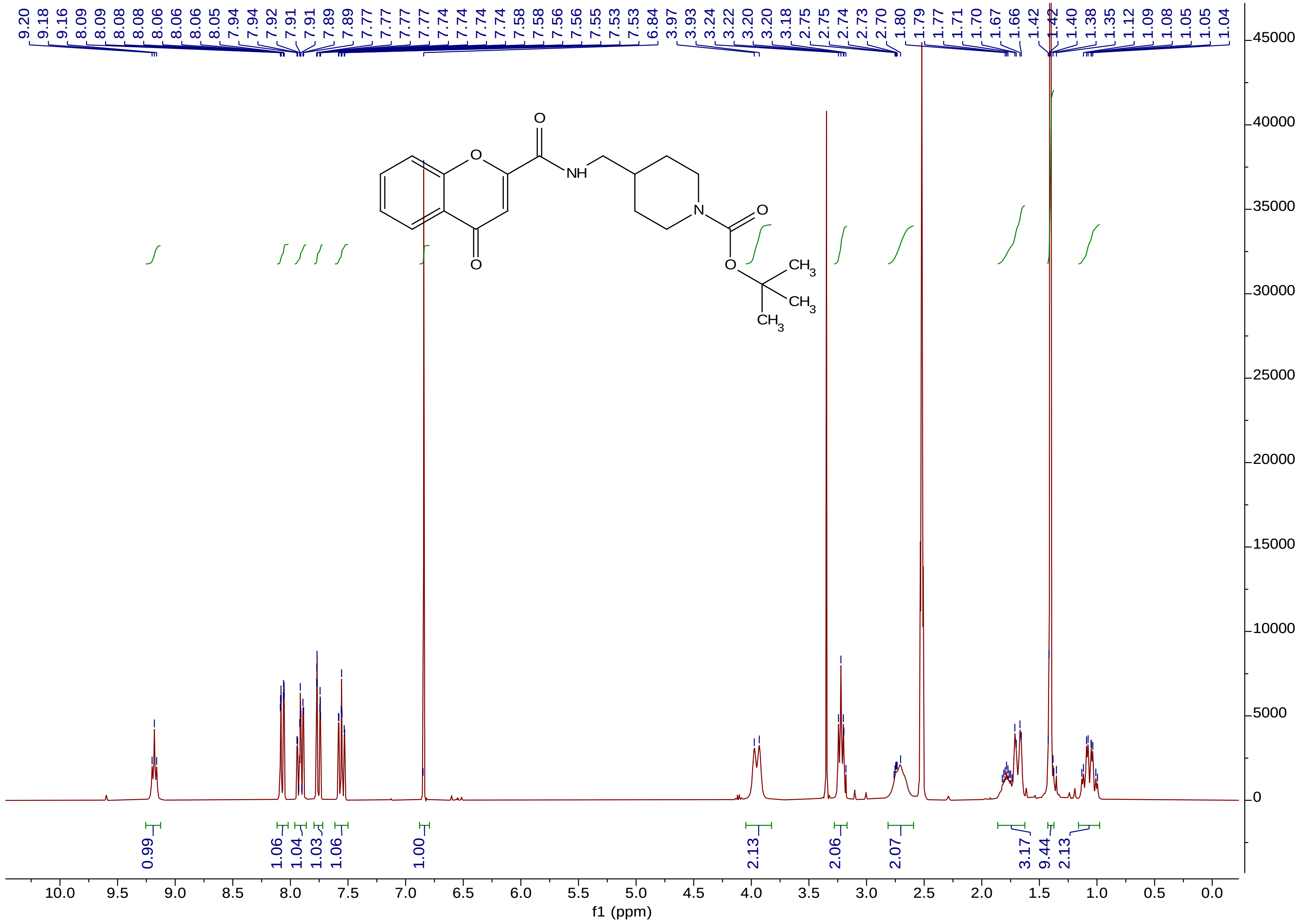

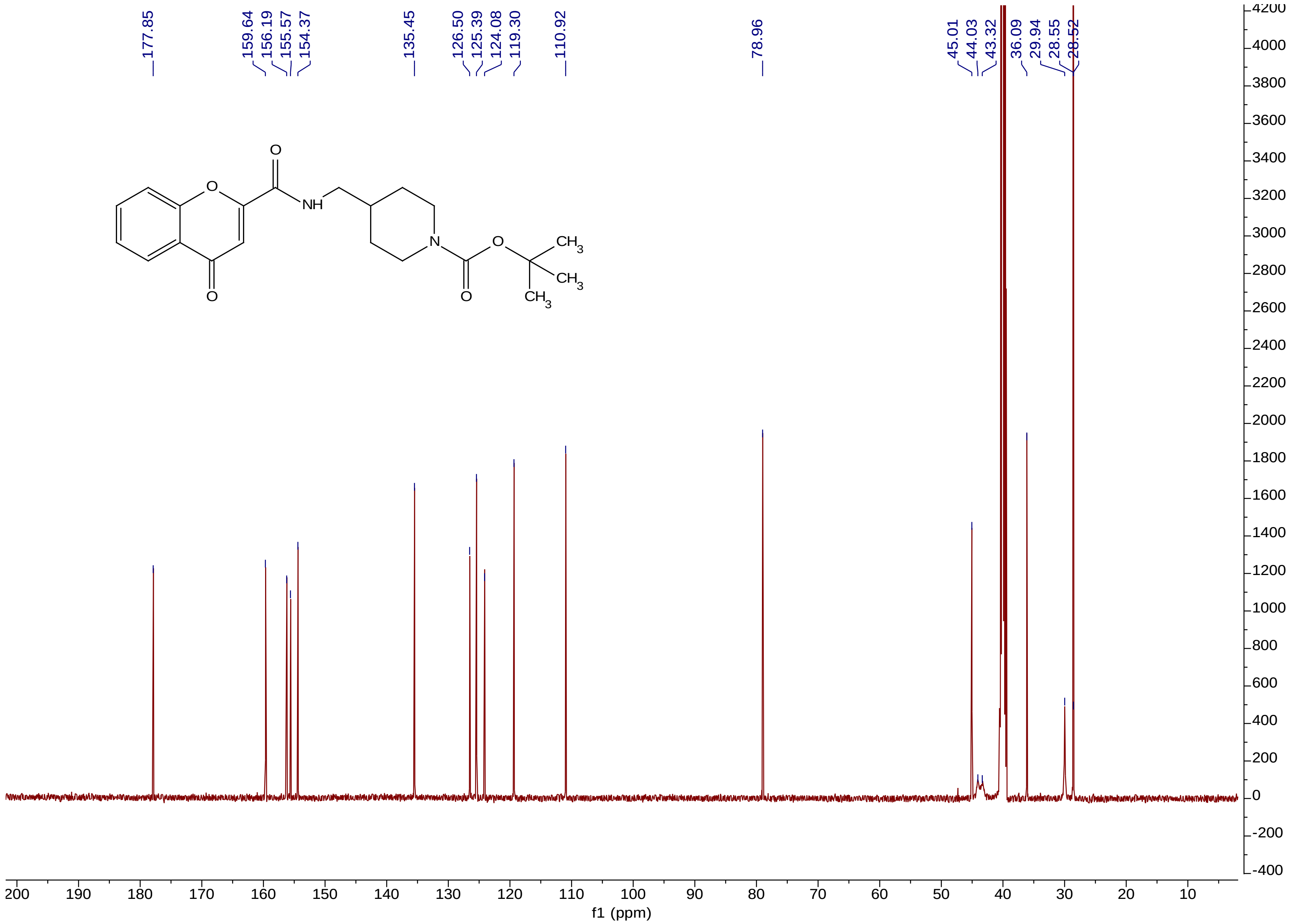
177.85
159.64
156.19
155.57
154.37
135.45
126.50
125.39
124.08
119.30
110.92
78.96
45.01
44.03
43.32
36.09
29.94
28.55
28.52
f1 (ppm)

***N*-(5-(Diethylamino)pentan-2-yl)-4-oxo-4*H*-chromene-2-carboxamide (3’f)**

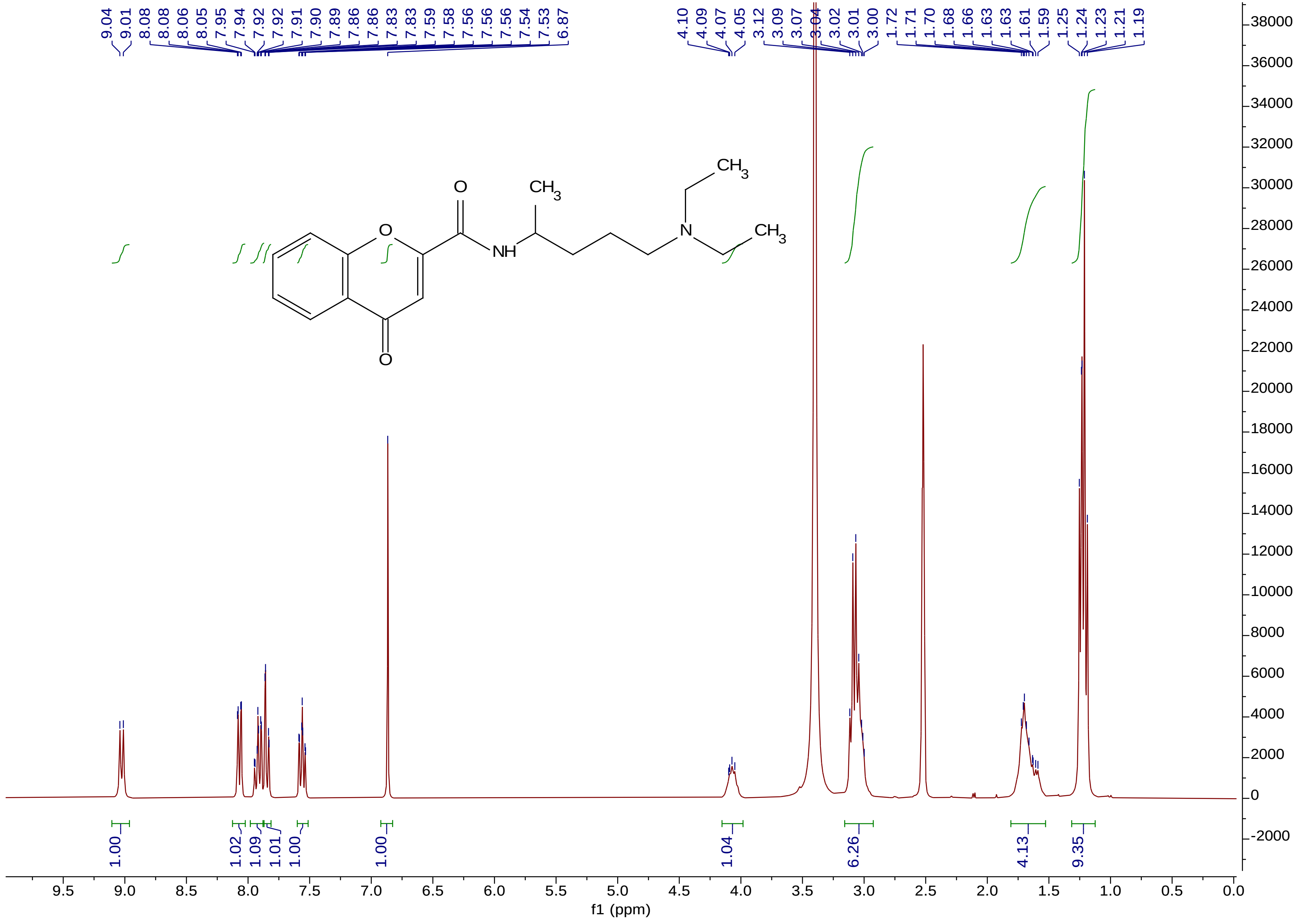

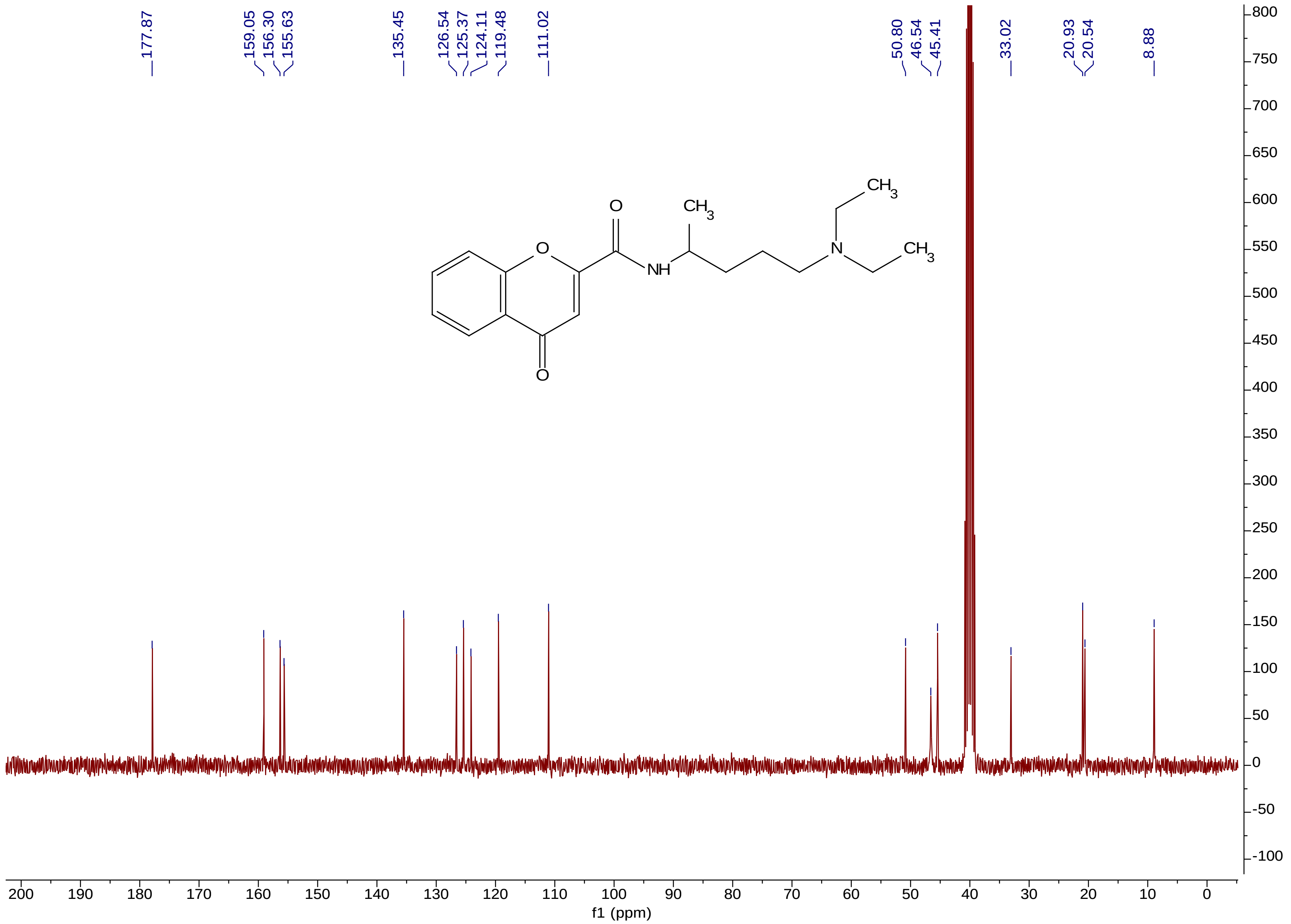
177.87
159.05
156.30
155.63
135.45
126.54
125.37
124.11
119.48
111.02
50.80
46.54
45.41
33.02
20.93
20.54
8.88
O
O
NH
CH3
N
CH3
CH3
O
f1 (ppm)

_N_-(3-(1_H_-Imidazol-1-yl)propyl)-4-oxo-4_H_-chromene-2-carboxamide (3’g)

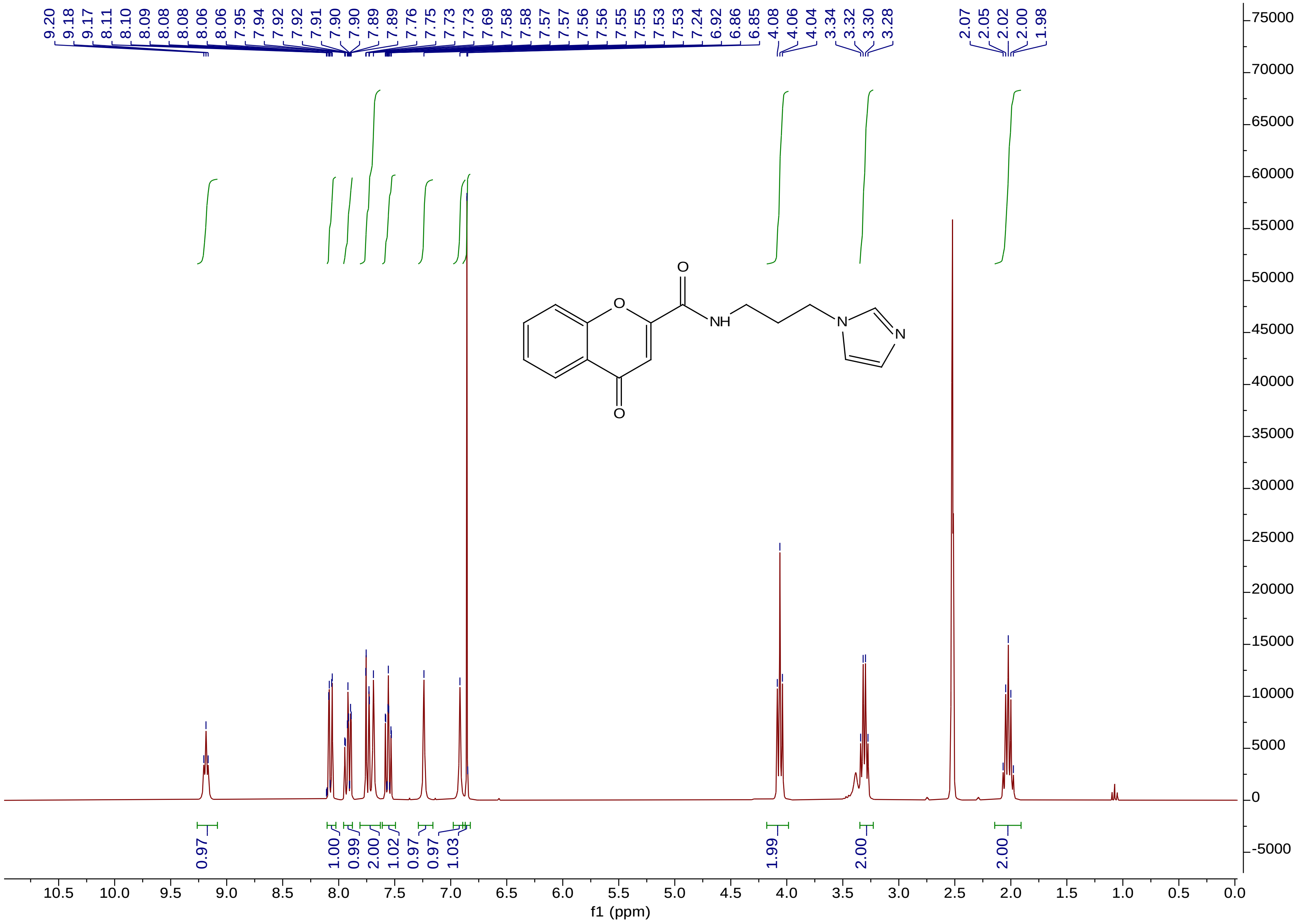

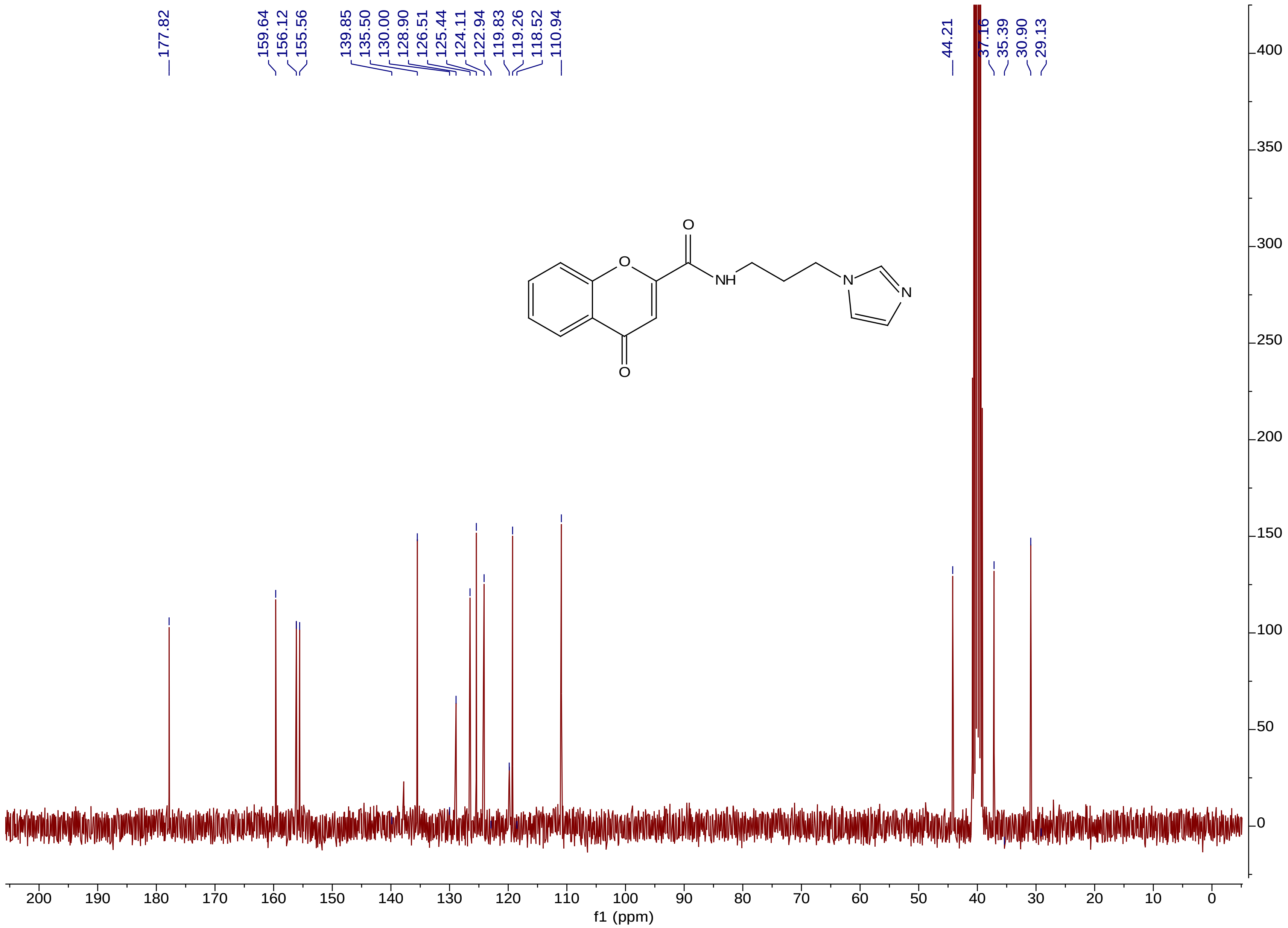
177.82
159.64
156.12
155.56
139.85
135.50
130.00
128.90
126.51
125.44
124.11
122.94
119.83
119.26
118.52
110.94
44.21
37.16
35.39
30.90
29.13
O
O
NH
N
N
O
f1 (ppm)

***N*-(4-Oxo-4*H*-chromen-2-yl)benzamide (6a)**

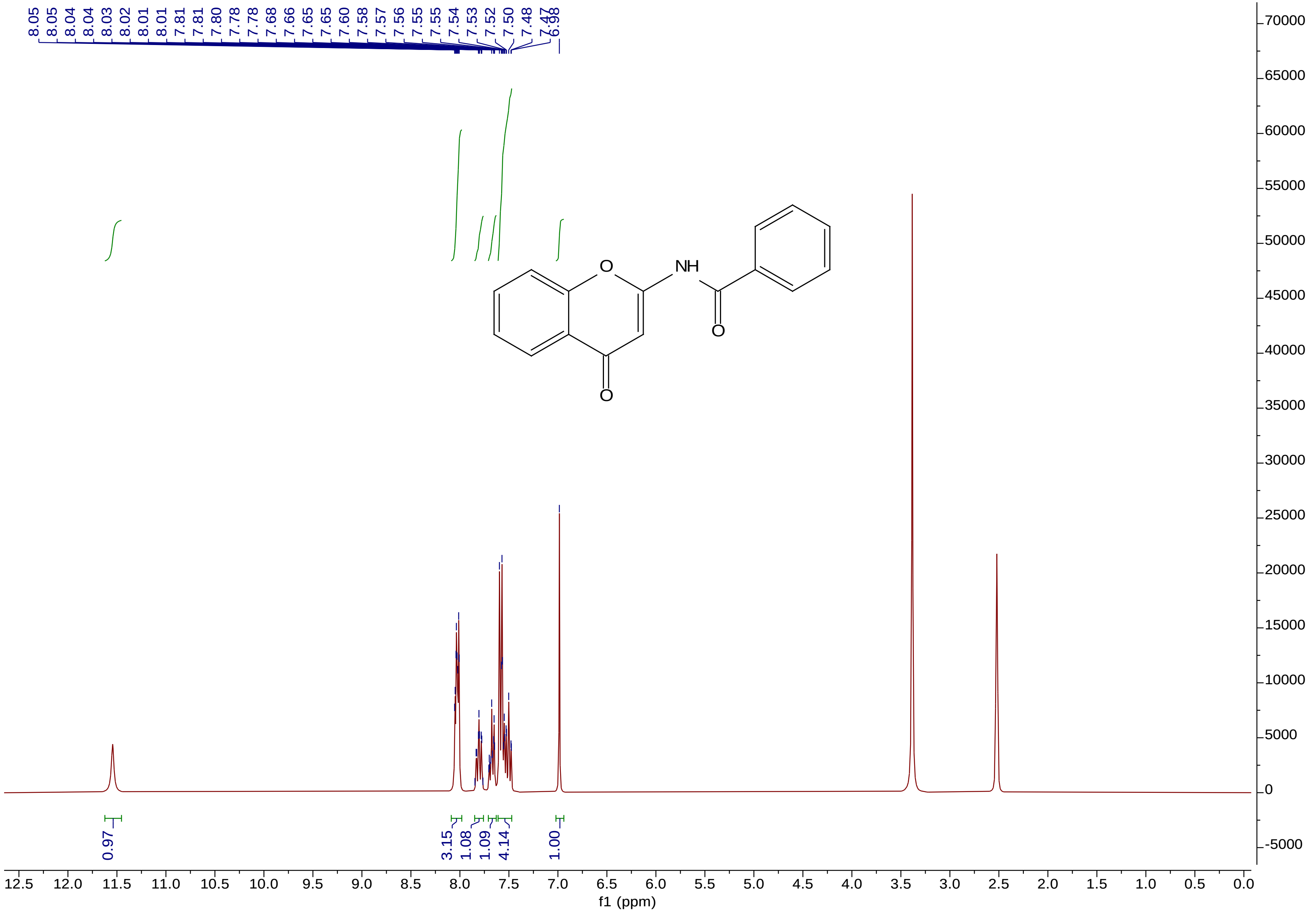

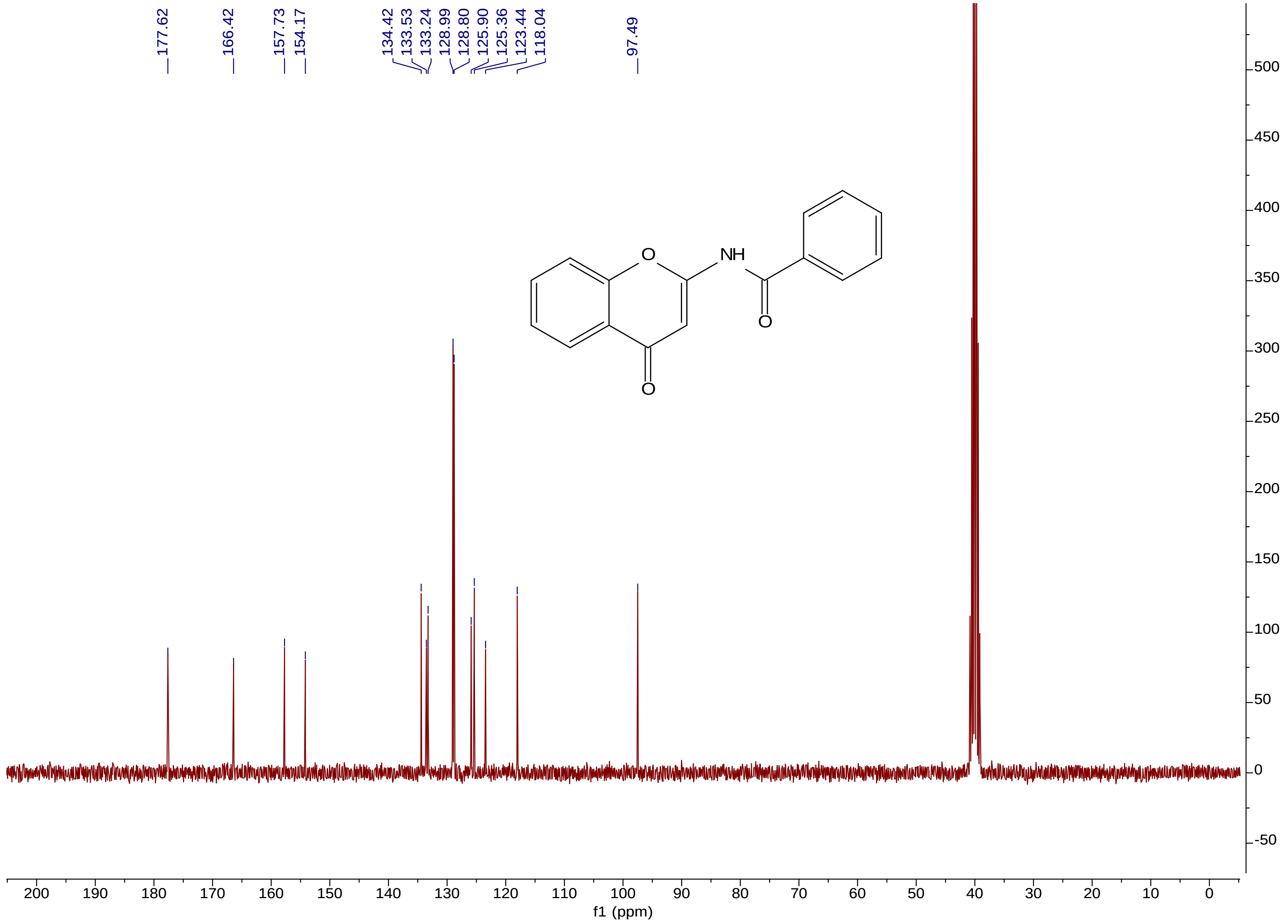
177.62
166.42
157.73
154.17
134.42
133.53
133.24
128.99
128.80
125.90
125.36
123.44
118.04
97.49
O
NH
O
O
f1 (ppm)

4-Methyl-*N*-(4-oxo-4*H*-chromen-2-yl)benzamide (6b)

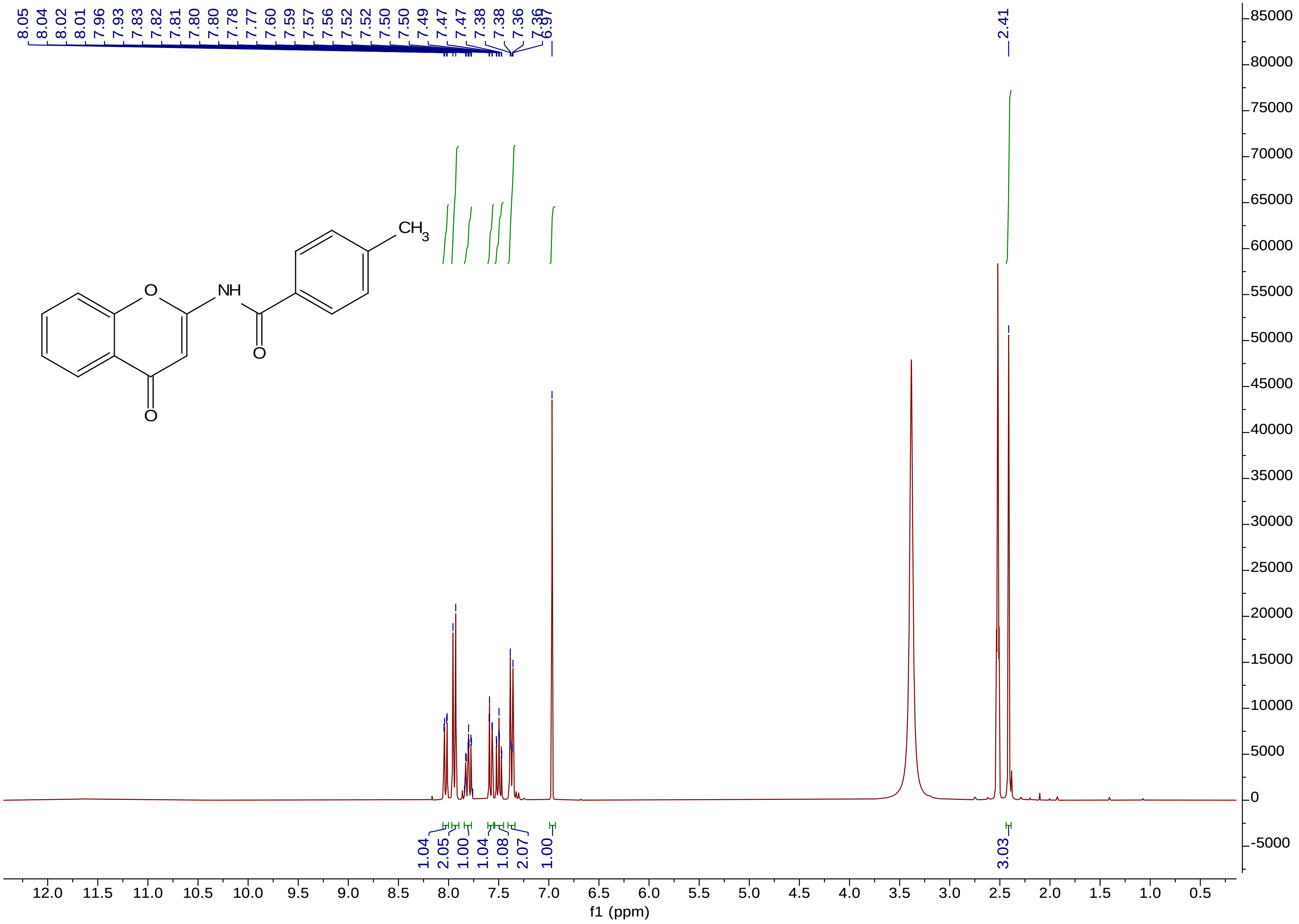

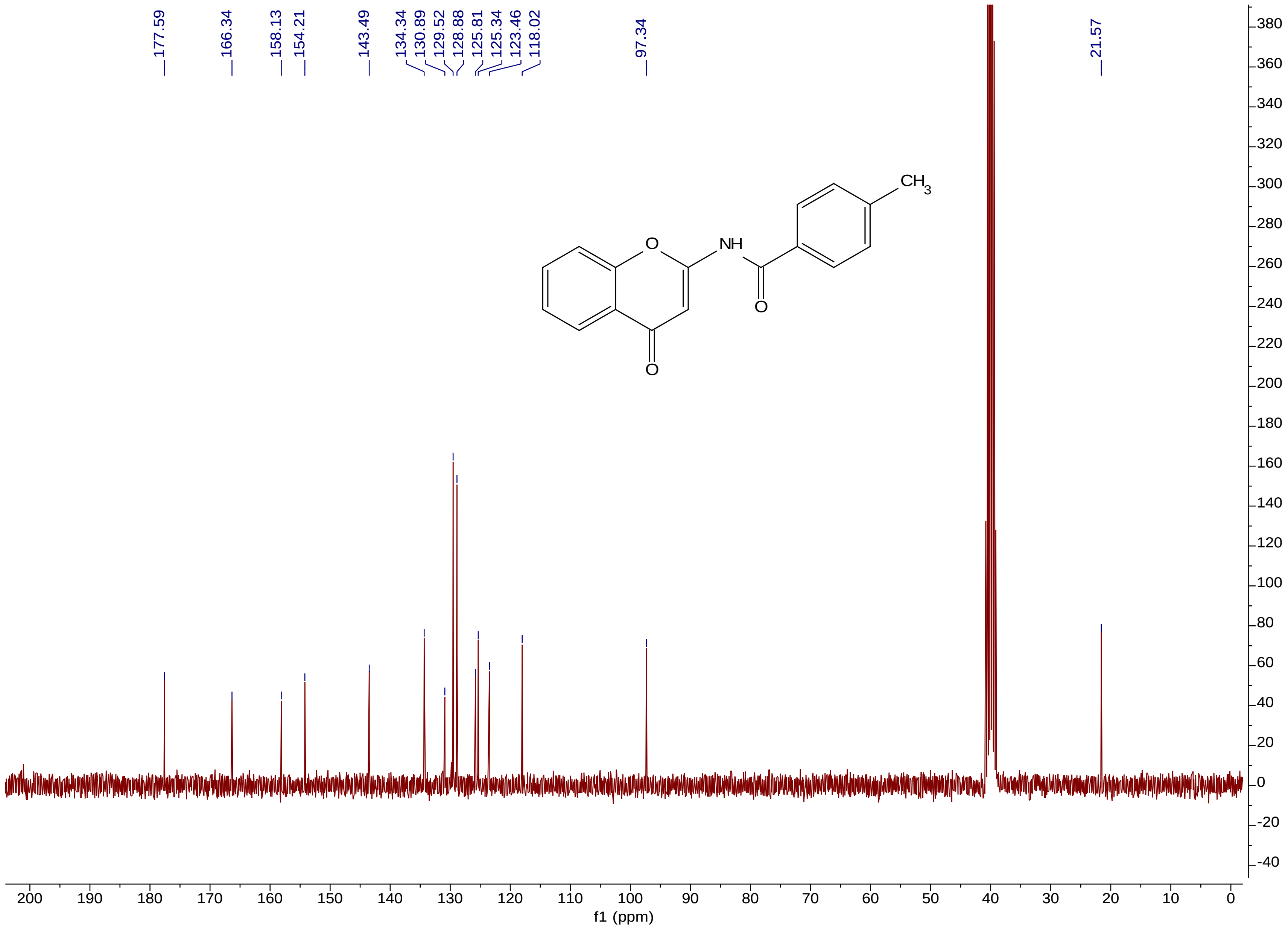
177.59
166.34
158.13
154.21
143.49
134.34
130.89
129.52
128.88
125.81
125.34
123.46
118.02
97.34
21.57
O
NH
O
CH3
O
f1 (ppm)

4-Ethyl-*N*-(4-oxo-4*H*-chromen-2-yl)benzamide (6c)

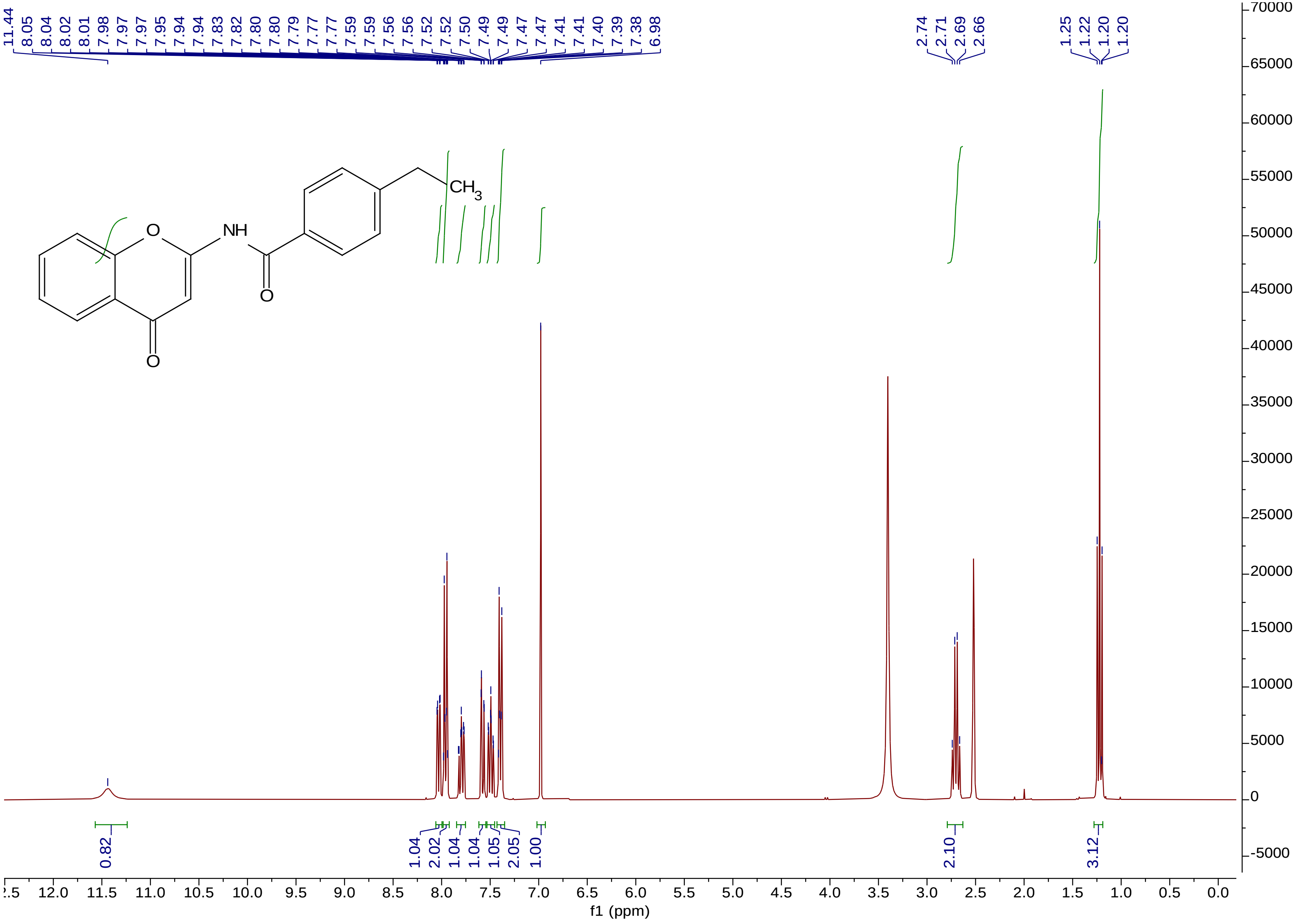

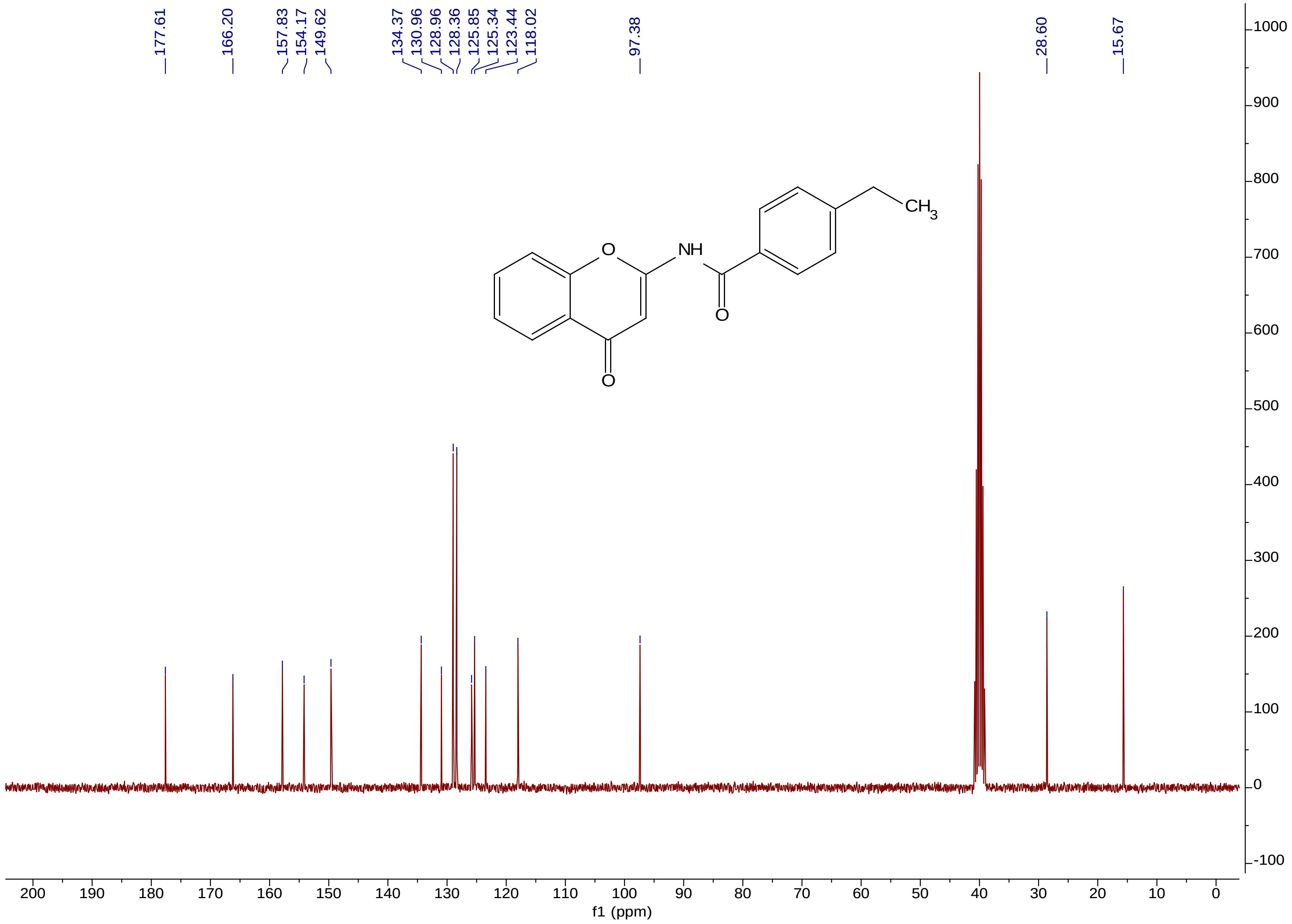
177.61
166.20
157.83
154.17
149.62
134.37
130.96
128.96
128.36
125.85
125.34
123.44
118.02
97.38
28.60
15.67
O
NH
O
O
CH3
f1 (ppm)

N-(4-Oxo-4H-chromen-2-yl)-4-propylbenzamide (6d)

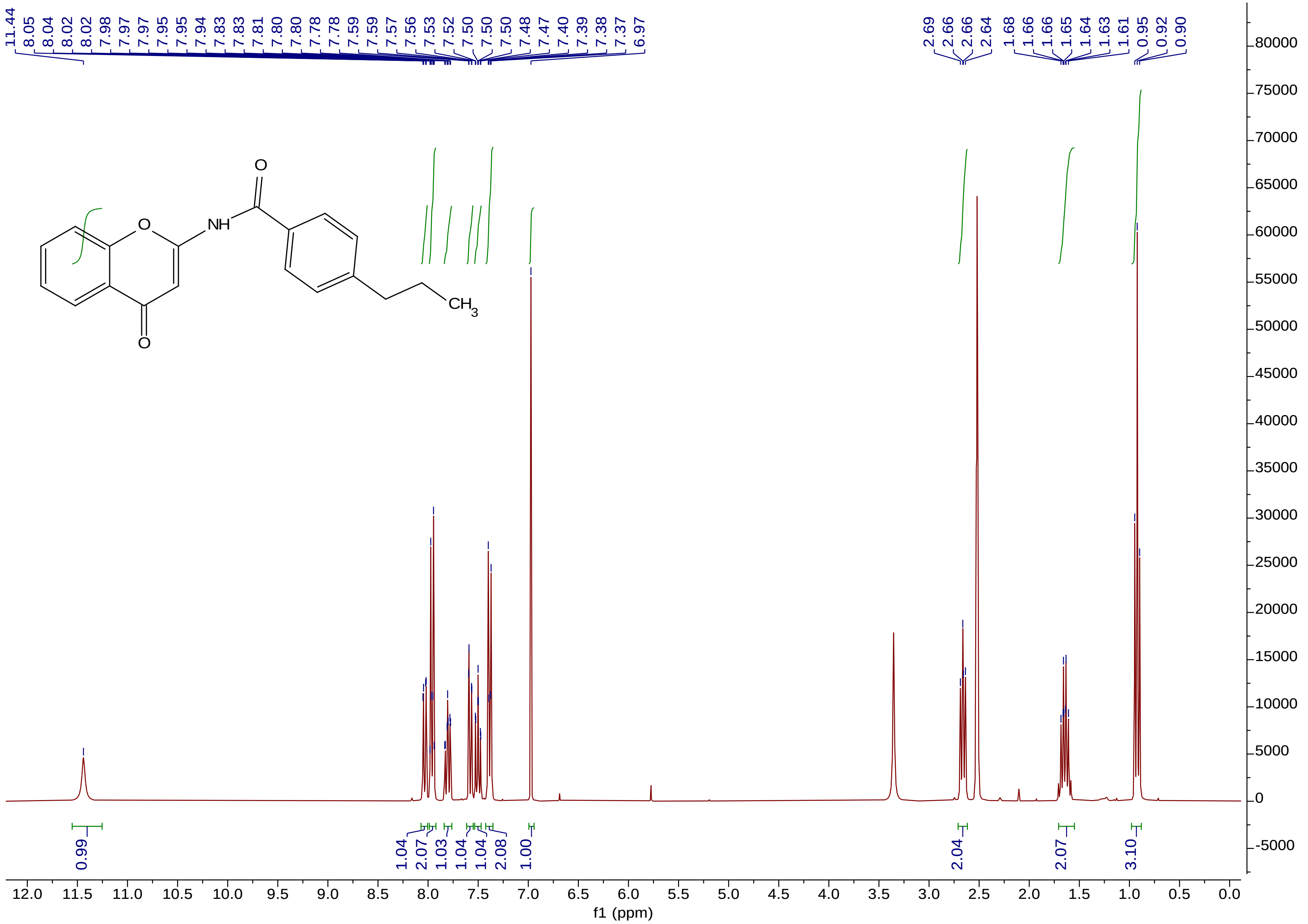

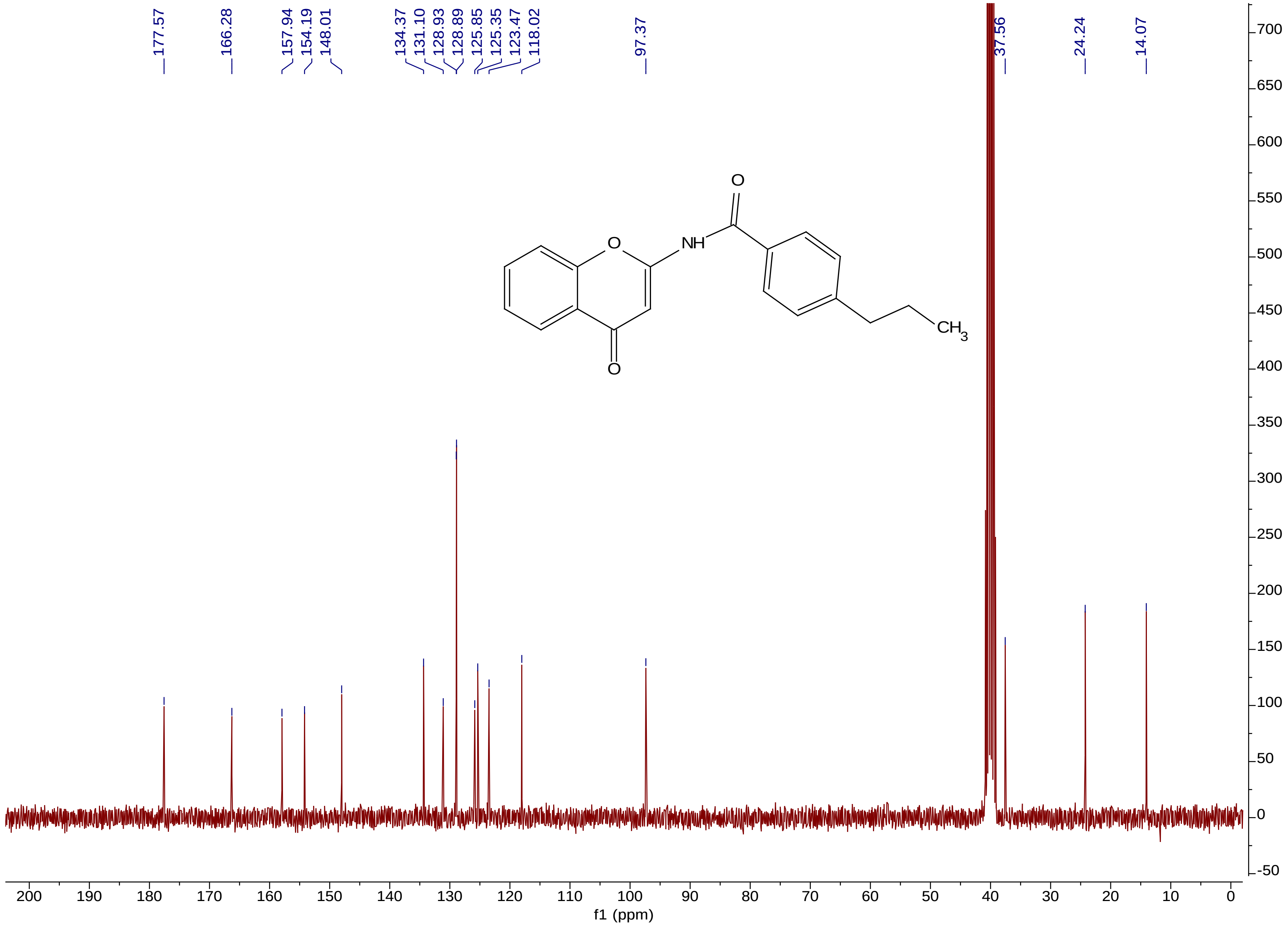
177.57
166.28
157.94
154.19
148.01
134.37
131.10
128.93
128.89
125.85
125.35
123.47
118.02
97.37
37.56
24.24
14.07
O
NH
O
O
CH3
f1 (ppm)

4-Fluoro-*N*-(4-oxo-4*H*-chromen-2-yl)benzamide (6e)

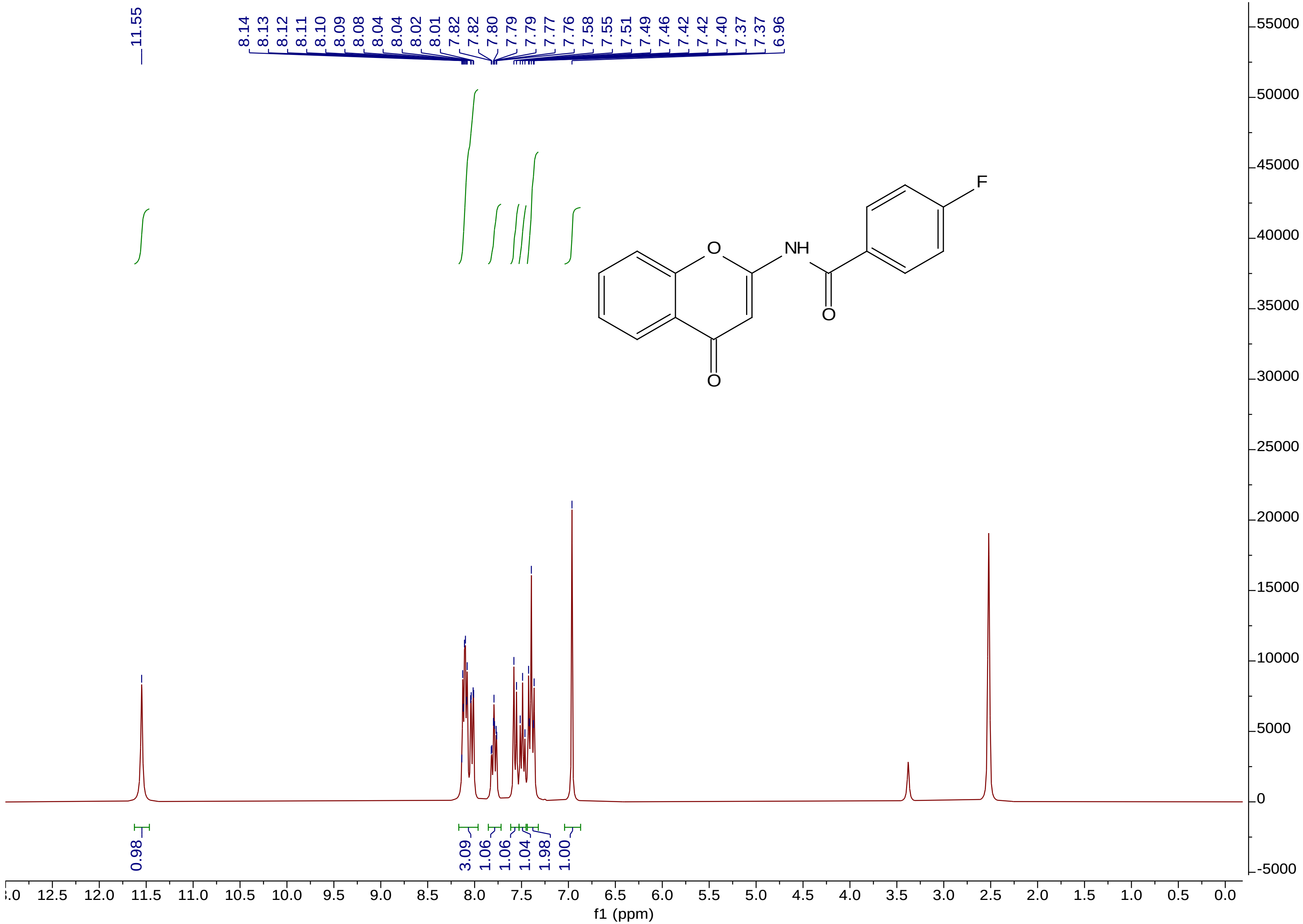

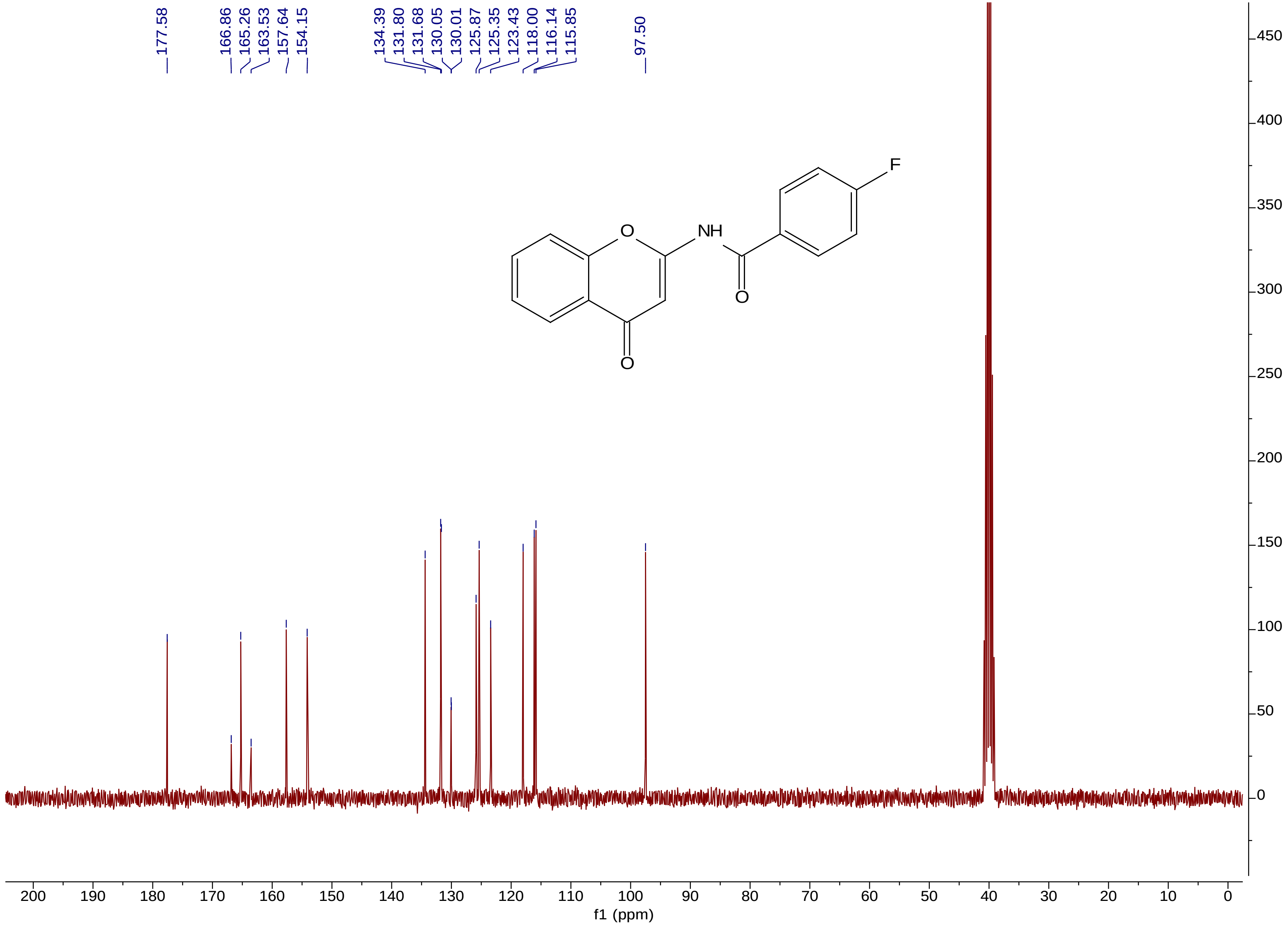
177.58
166.86
165.26
163.53
157.64
154.15
134.39
131.80
131.68
130.05
130.01
125.87
125.35
123.43
118.00
116.14
115.85
97.50
O
NH
F
O
O
f1 (ppm)

4-Chloro-*N*-(4-oxo-4*H*-chromen-2-yl)benzamide (6f)

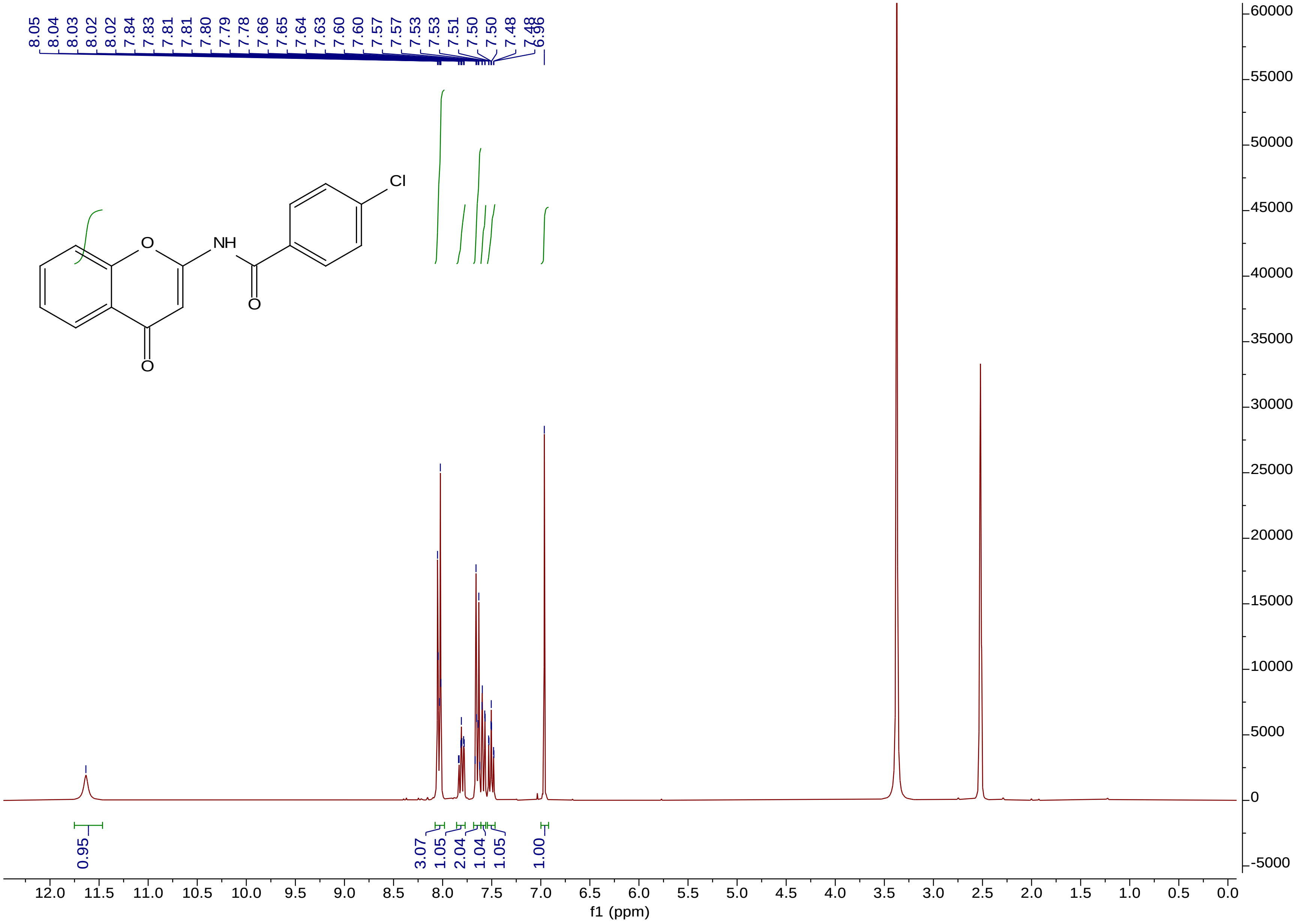

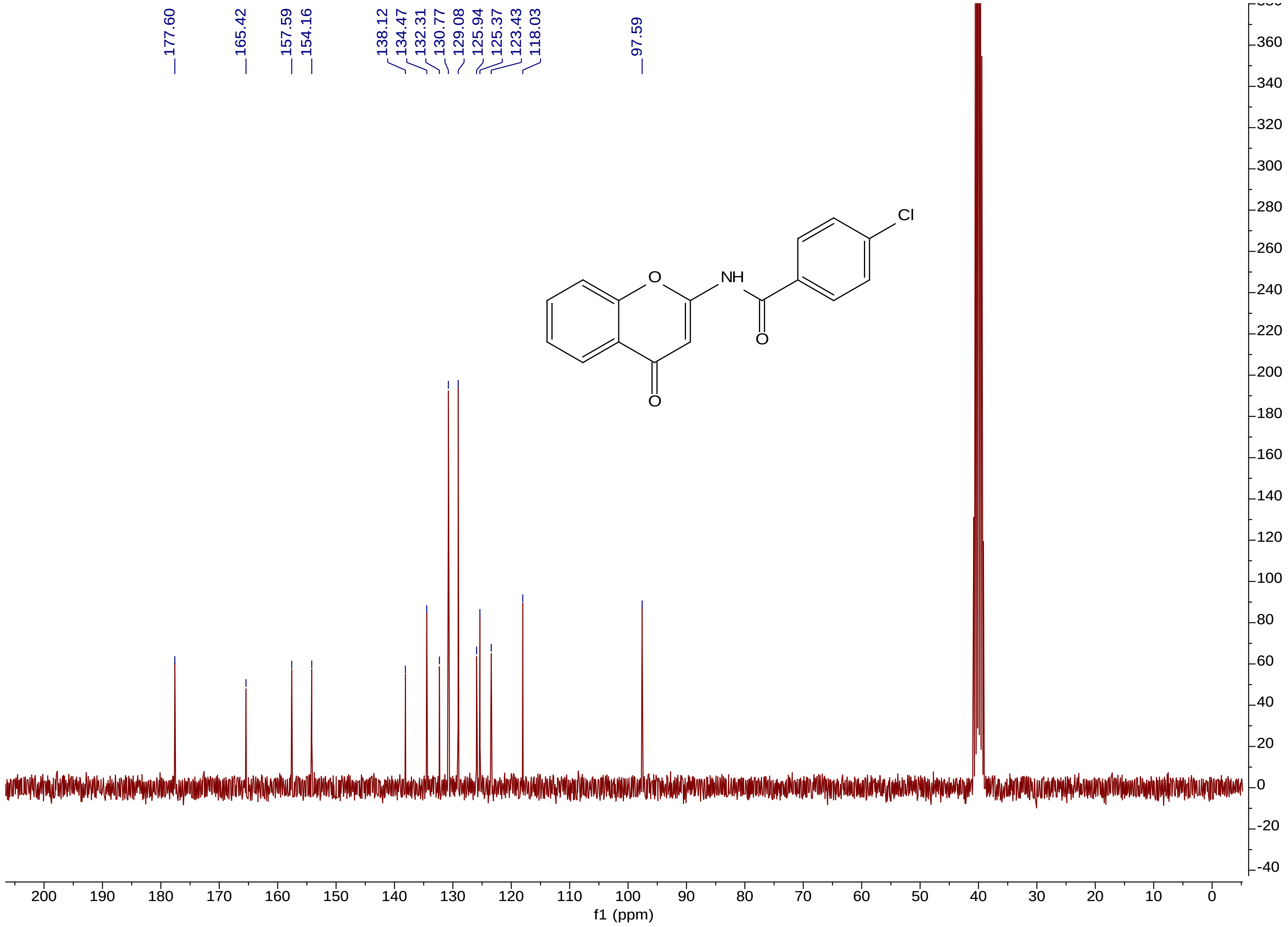
177.60
165.42
157.59
154.16
138.12
134.47
132.31
130.77
129.08
125.94
125.37
123.43
118.03
97.59
O
NH
Cl
O
O
f1 (ppm)

4-Bromo-*N*-(4-oxo-4*H*-chromen-2-yl)benzamide (6g)

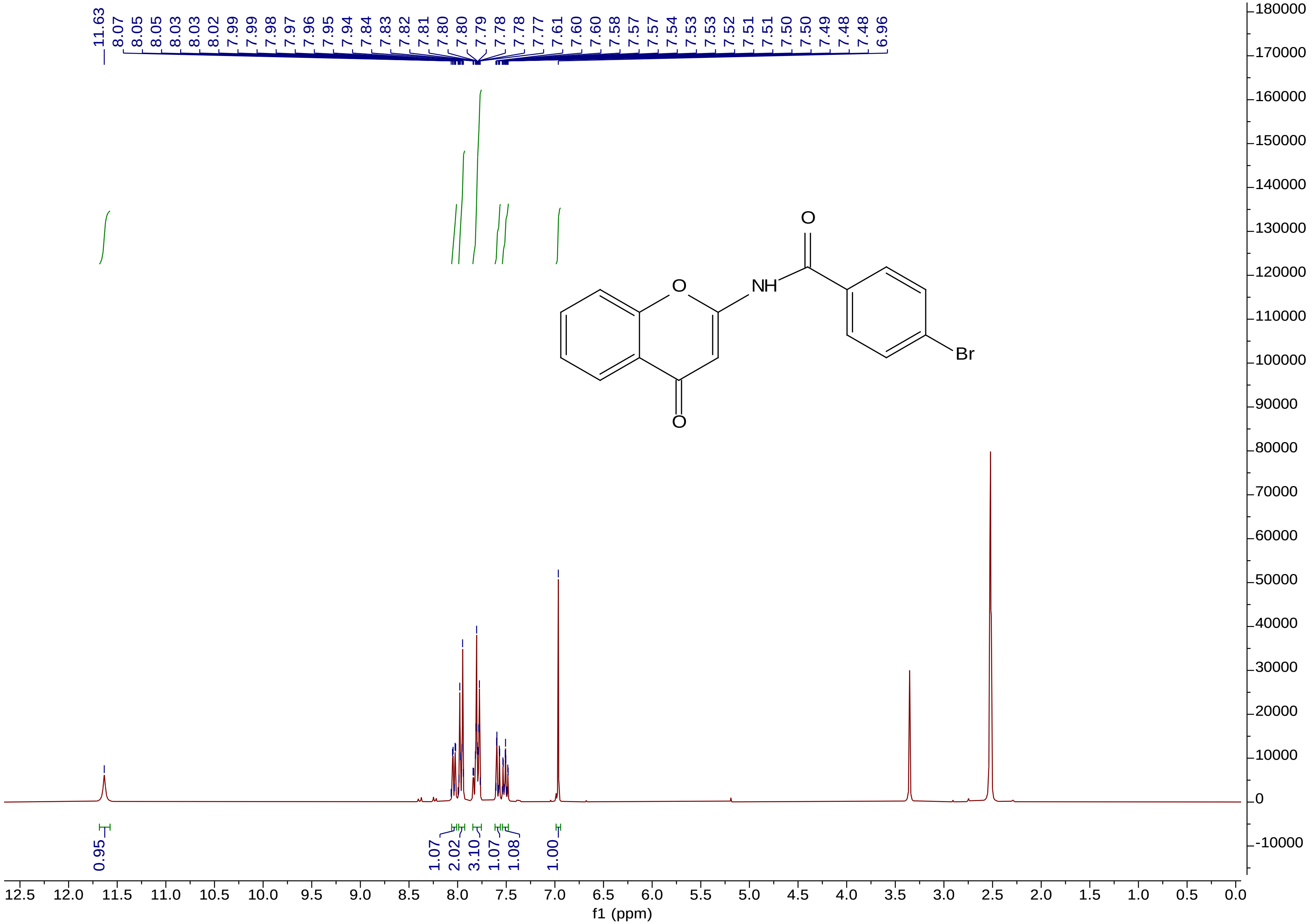

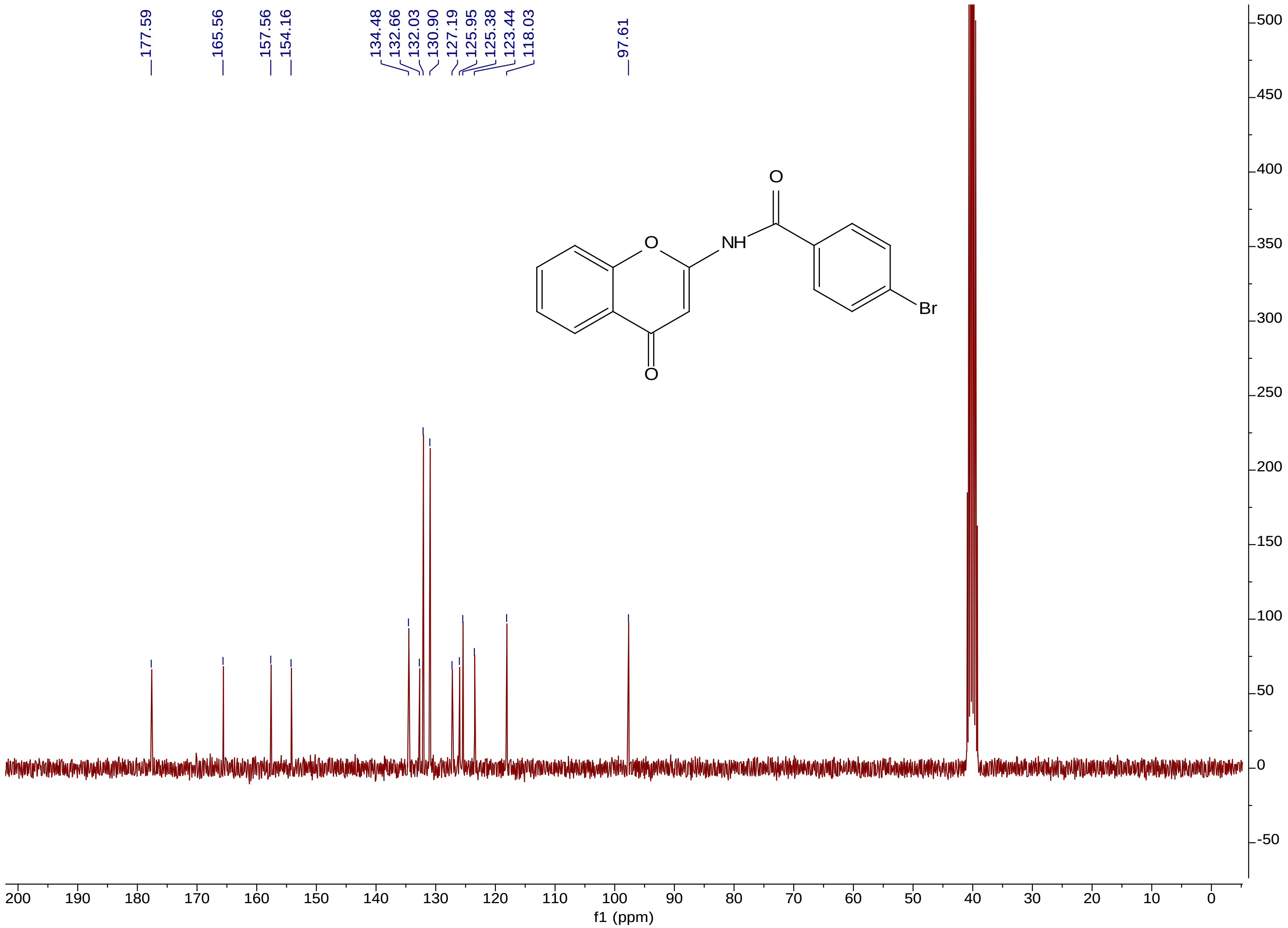
177.59
165.56
157.56
154.16
134.48
132.66
132.03
130.90
127.19
125.95
125.38
123.44
118.03
97.61
O
NH
O
Br
O
f1 (ppm)

2-Fluoro-*N*-(4-oxo-4*H*-chromen-2-yl)benzamide (6h)

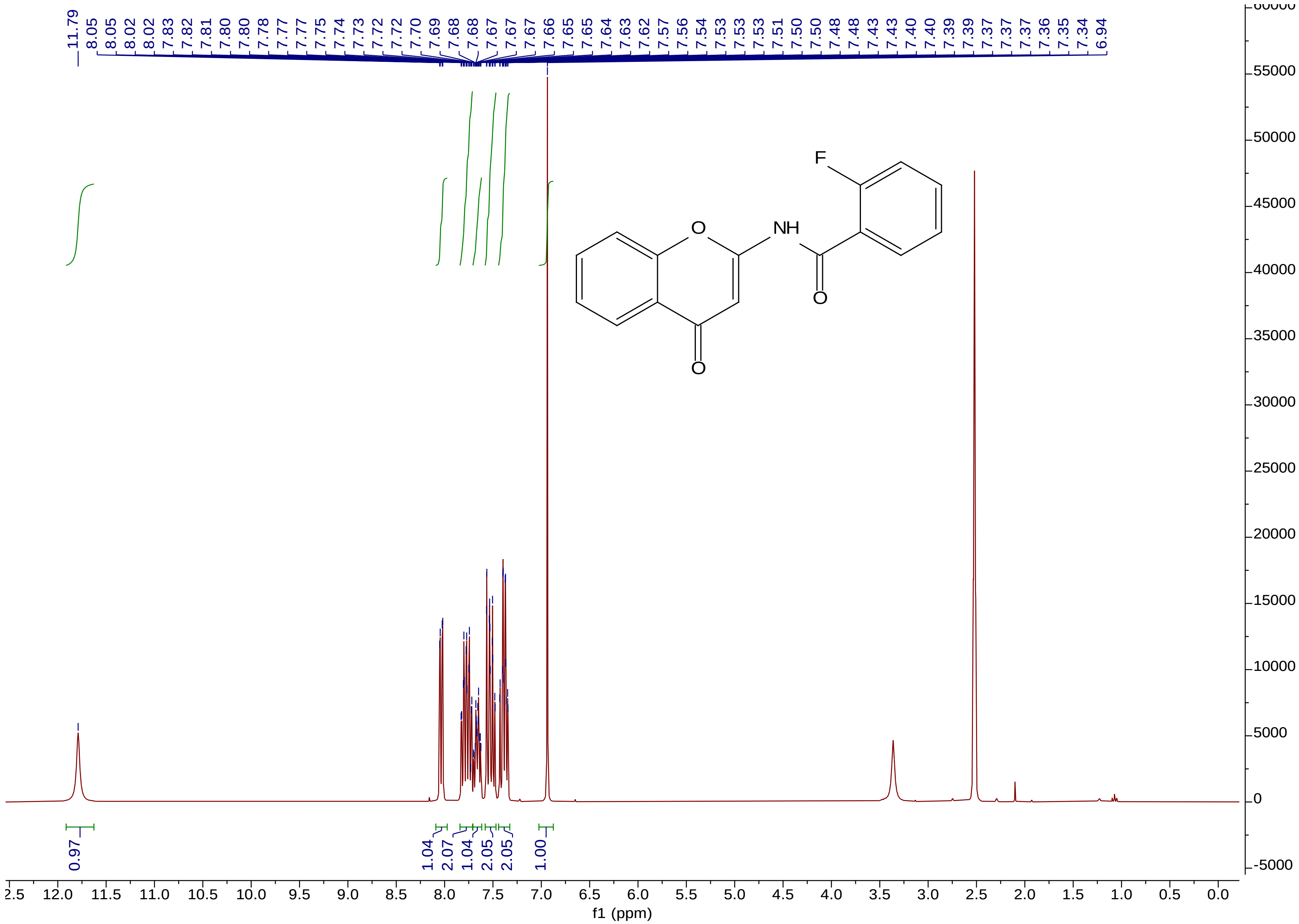

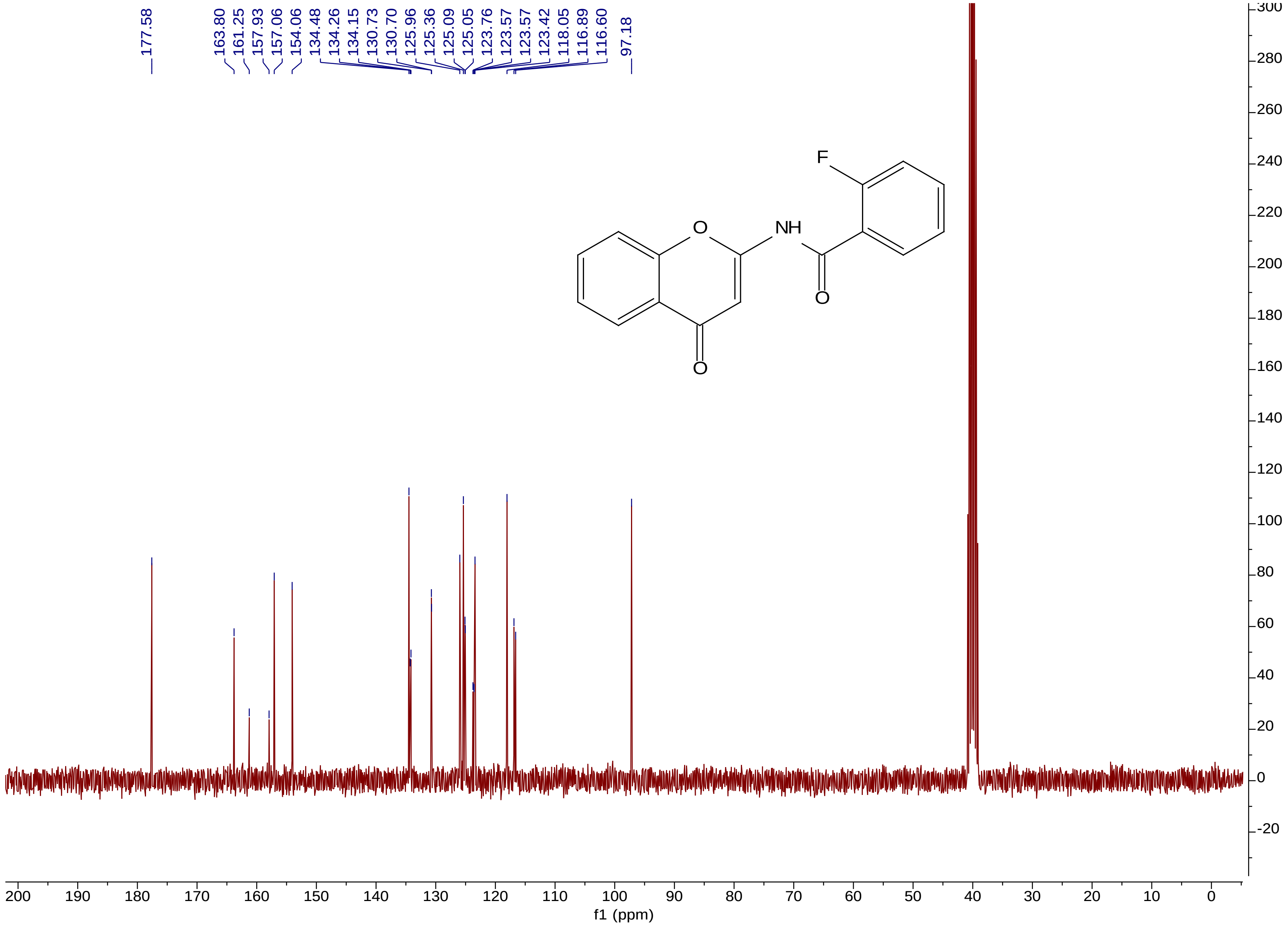
177.58
163.80
161.25
157.93
157.06
154.06
134.48
134.26
134.15
130.73
130.70
125.96
125.36
125.09
125.05
123.76
123.57
123.57
123.42
118.05
116.89
116.60
97.18
F
NH
O
O
O
f1 (ppm)

2-Chloro-*N*-(4-oxo-4*H*-chromen-2-yl)benzamide (6i)

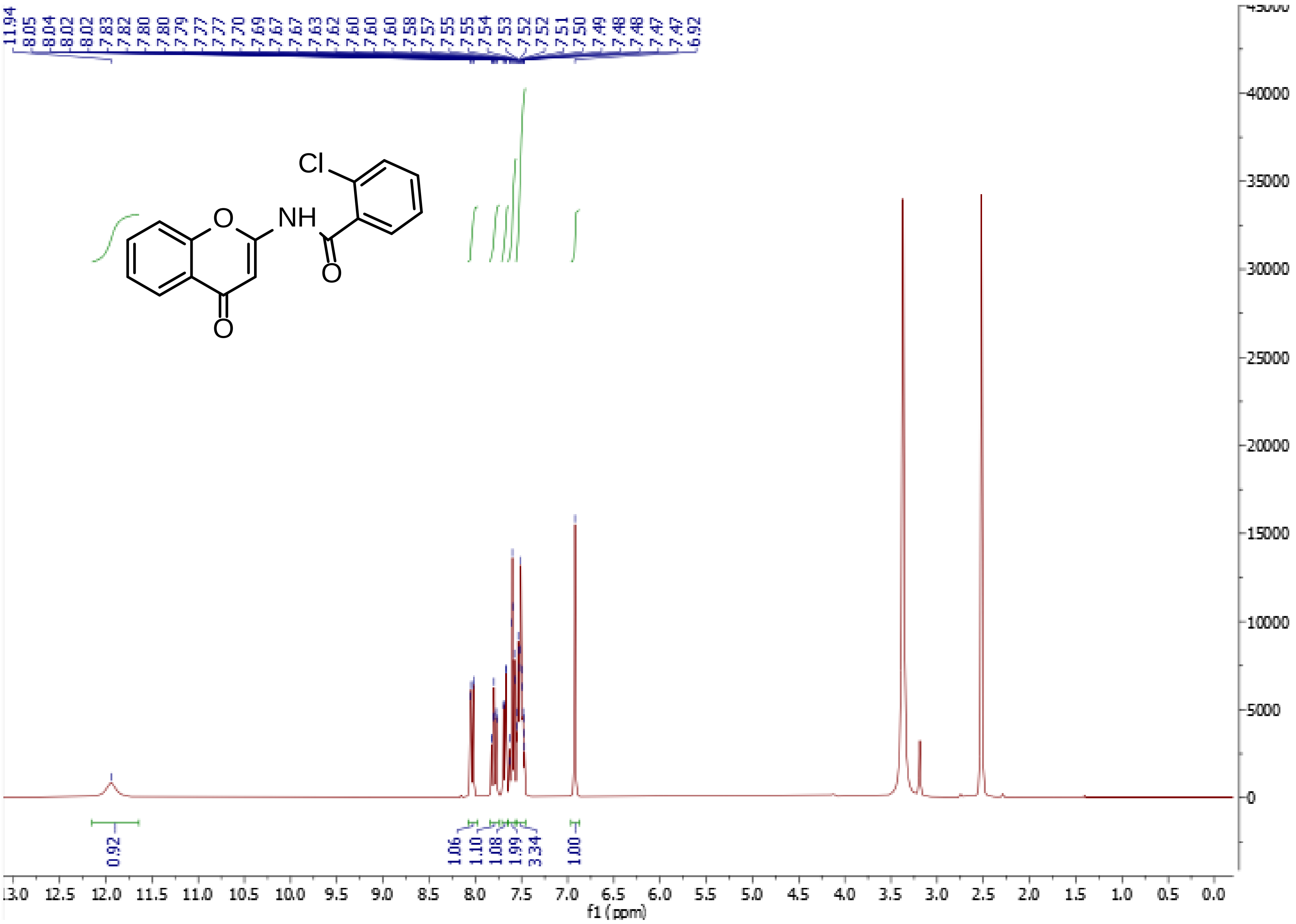

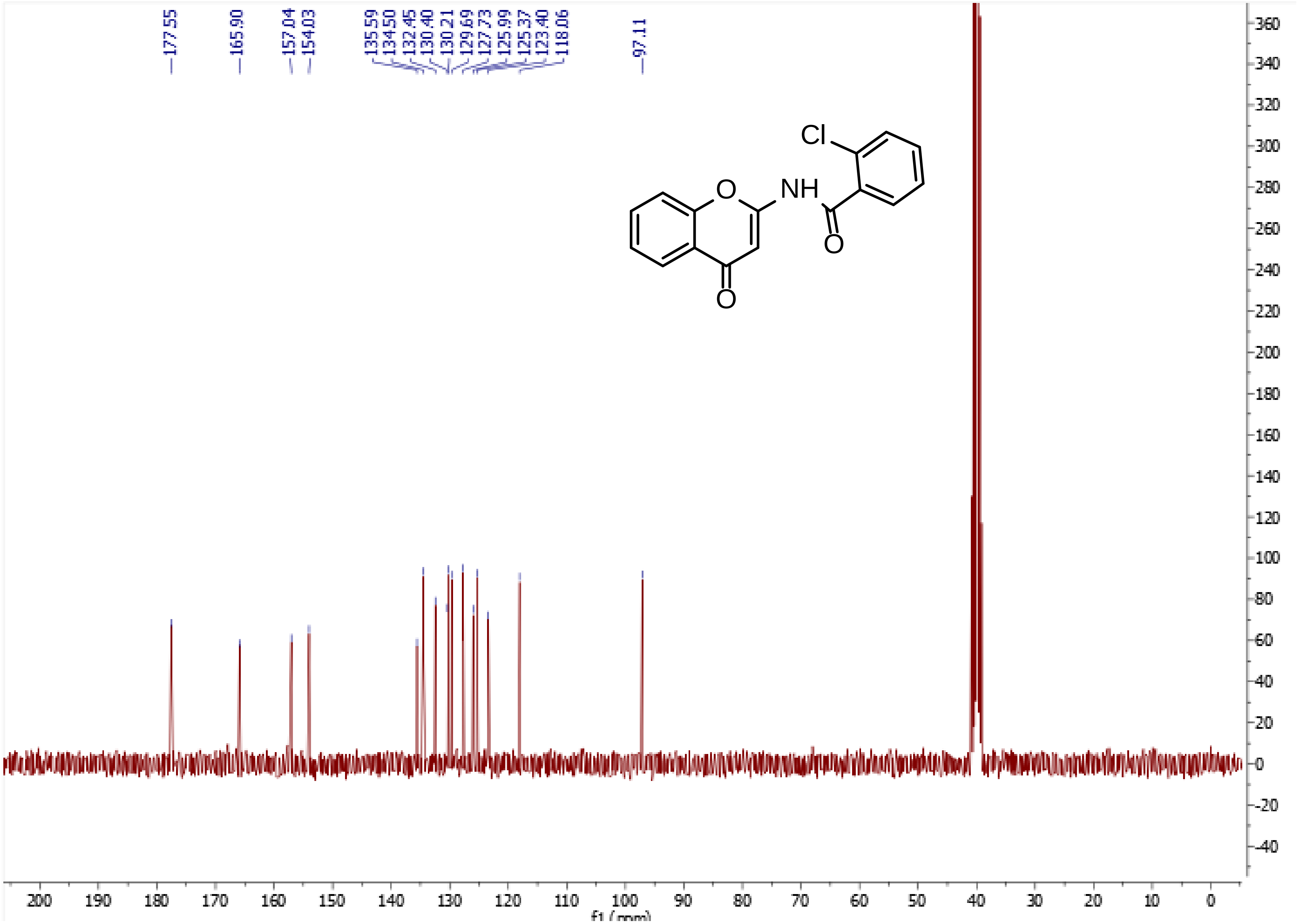
177.55
165.90
157.04
154.03
135.59
134.50
132.45
130.40
130.21
129.69
127.73
125.99
125.37
123.40
118.06
97.11
Cl
O
NH
O
O
f1 (ppm)

2-Bromo-*N*-(4-oxo-4*H*-chromen-2-yl)benzamide (6j)

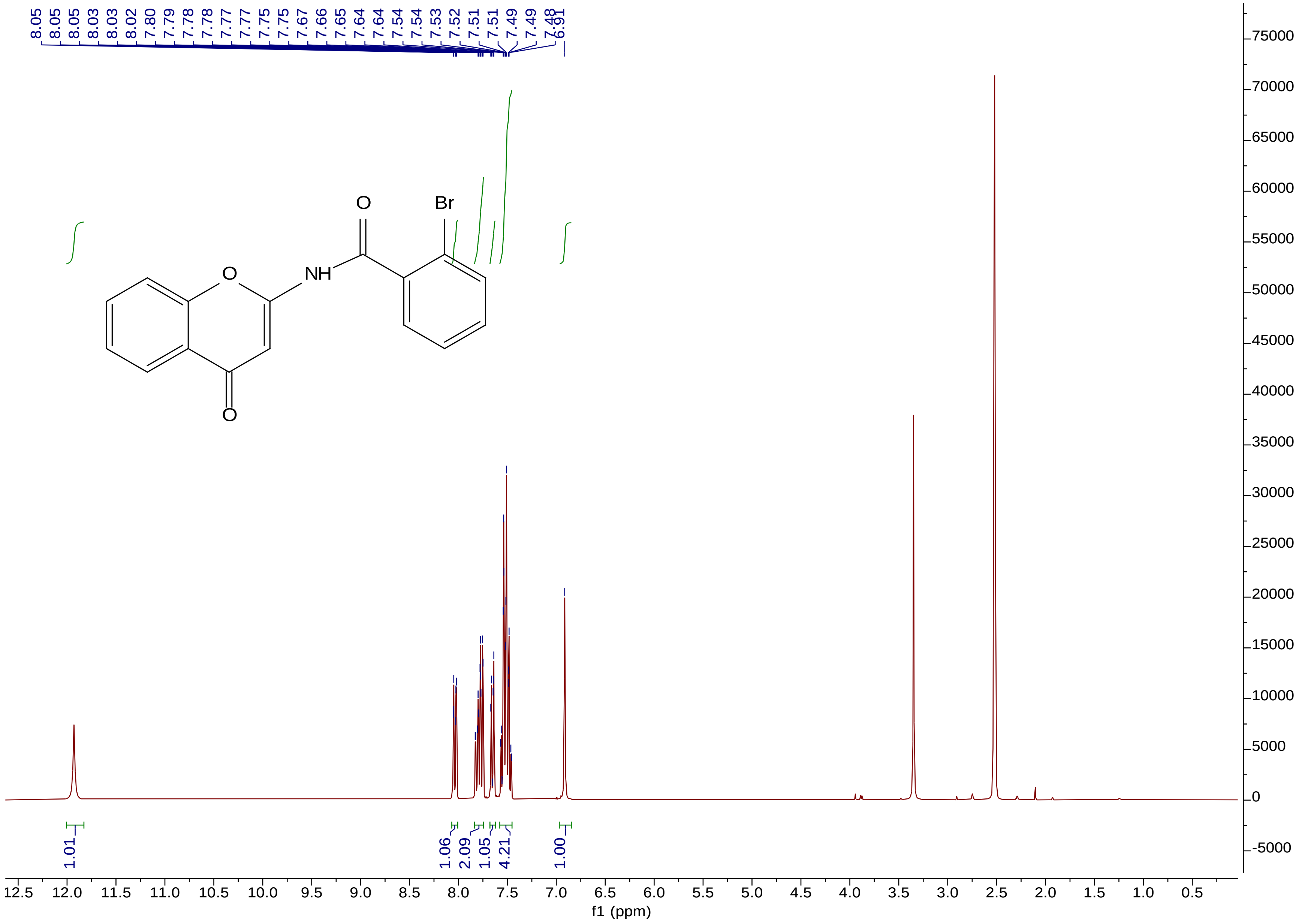

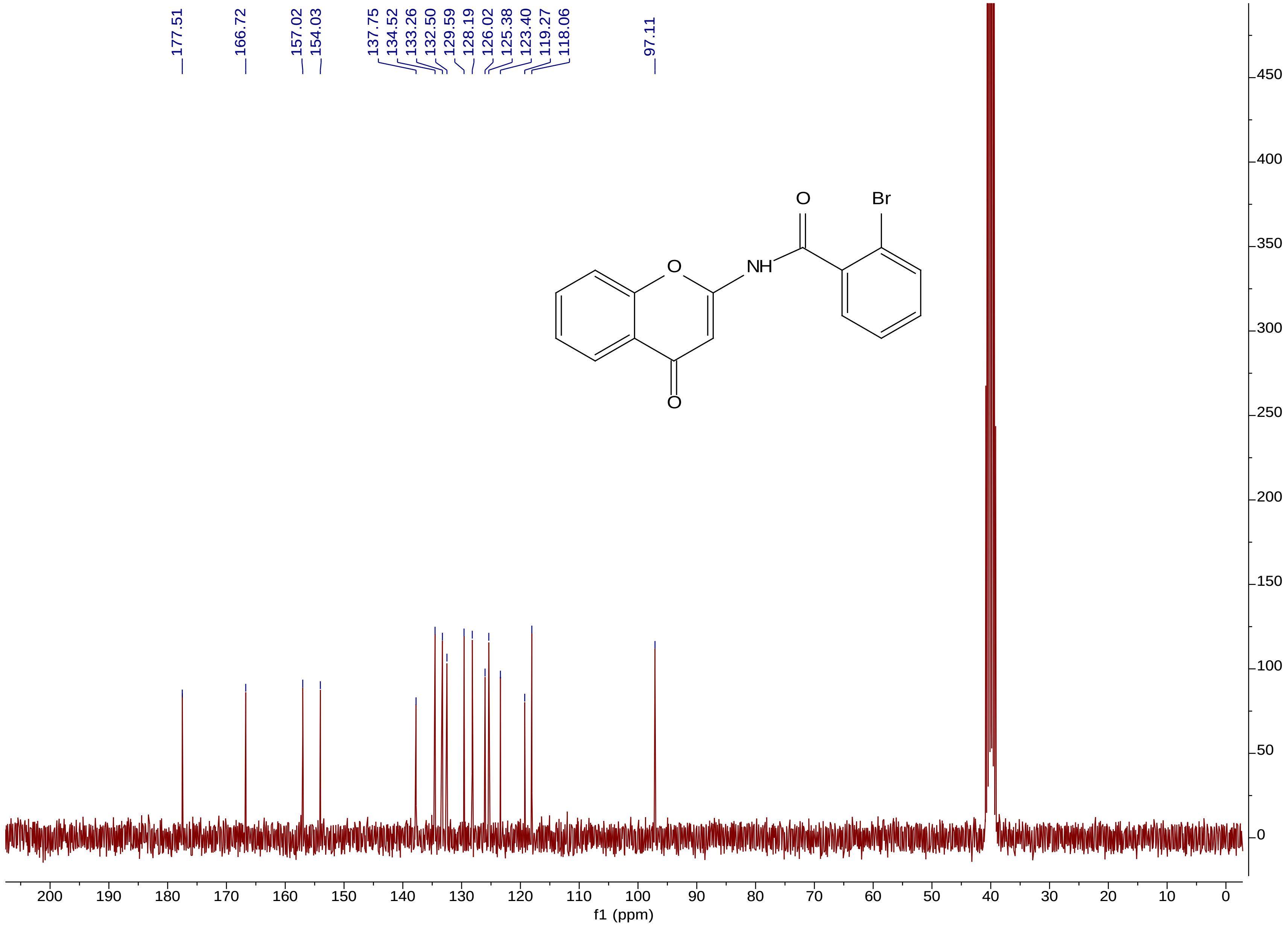
177.51
166.72
157.02
154.03
137.75
134.52
133.26
132.50
129.59
128.19
126.02
125.38
123.40
119.27
118.06
97.11
O
Br
O
NH
O
f1 (ppm)

2-Chloro-4-fluoro-*N*-(4-oxo-4*H*-chromen-2-yl)benzamide (6k)

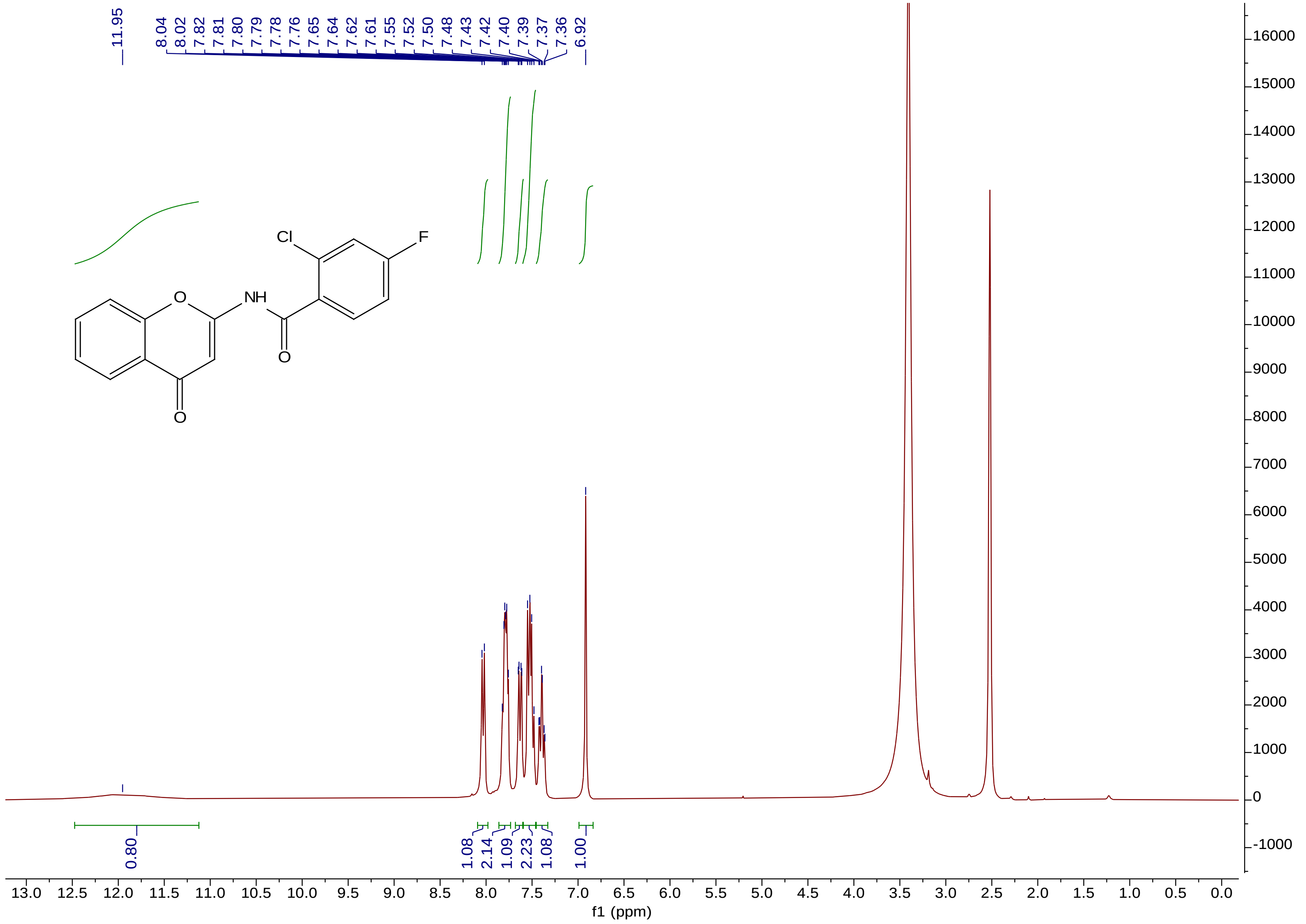

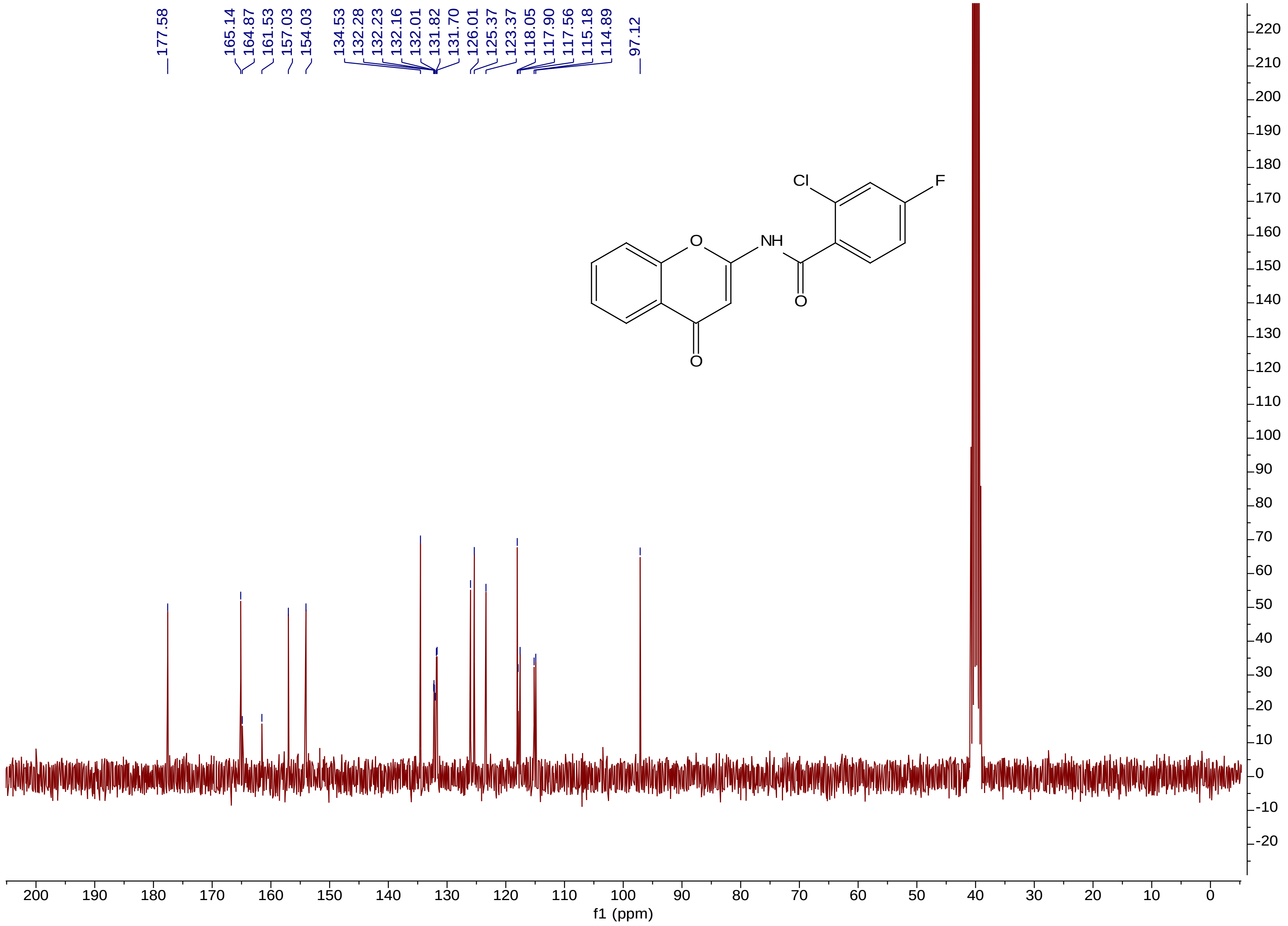
177.58
165.14
164.87
161.53
157.03
154.03
134.53
132.28
132.23
132.16
132.01
131.82
131.70
126.01
125.37
123.37
118.05
117.90
117.56
115.18
114.89
97.12
Cl
F
O
NH
O
O
f1 (ppm)

3-Chloro-4-fluoro-*N*-(4-oxo-4*H*-chromen-2-yl)benzamide (6l)

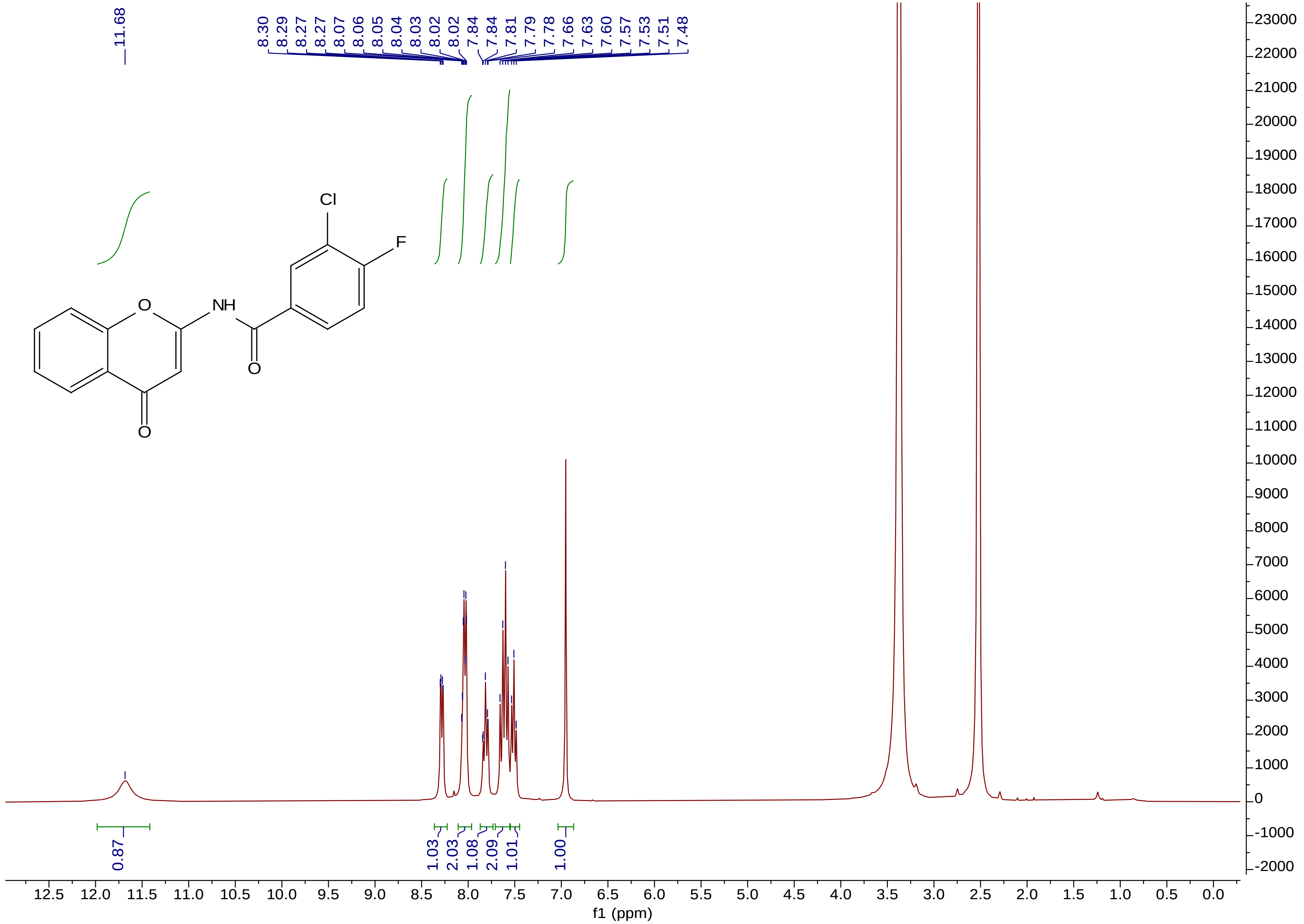

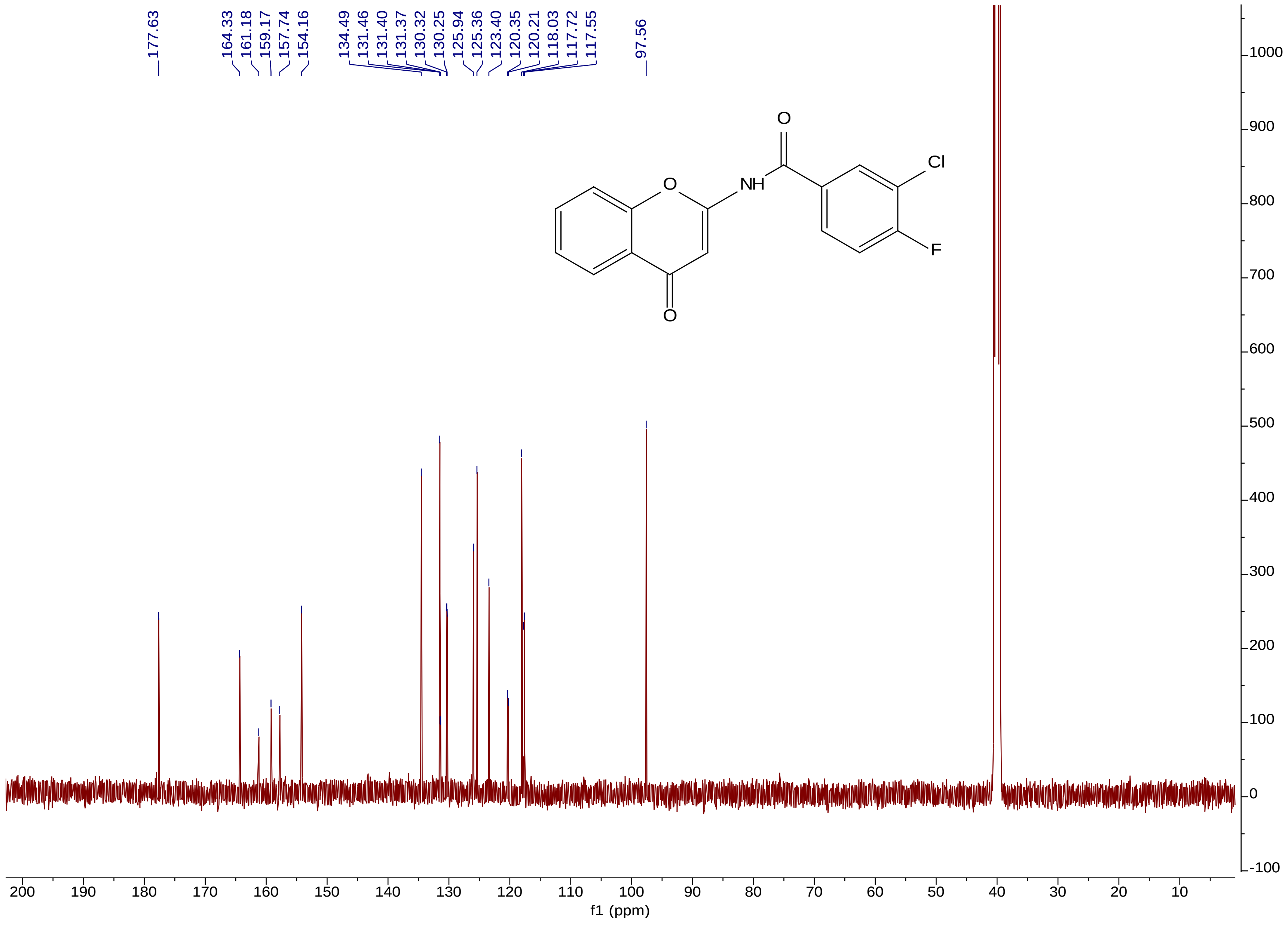
177.63
164.33
161.18
159.17
157.74
154.16
134.49
131.46
131.40
131.37
130.32
130.25
125.94
125.36
123.40
120.35
120.21
118.03
117.72
117.55
97.56
O
NH
Cl
F
f1 (ppm)

4-Nitro-*N*-(4-oxo-4*H*-chromen-2-yl)benzamide (6m)

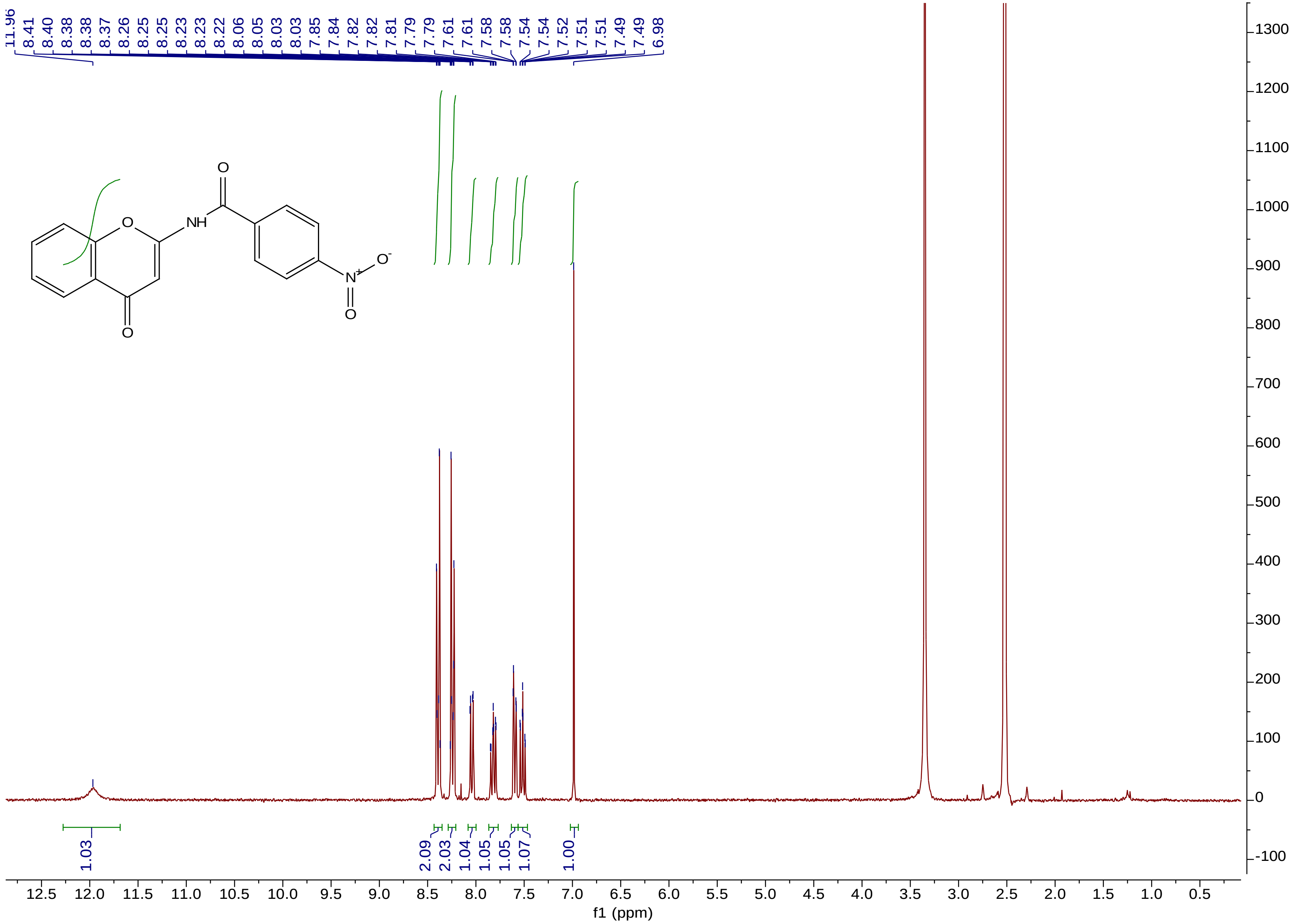

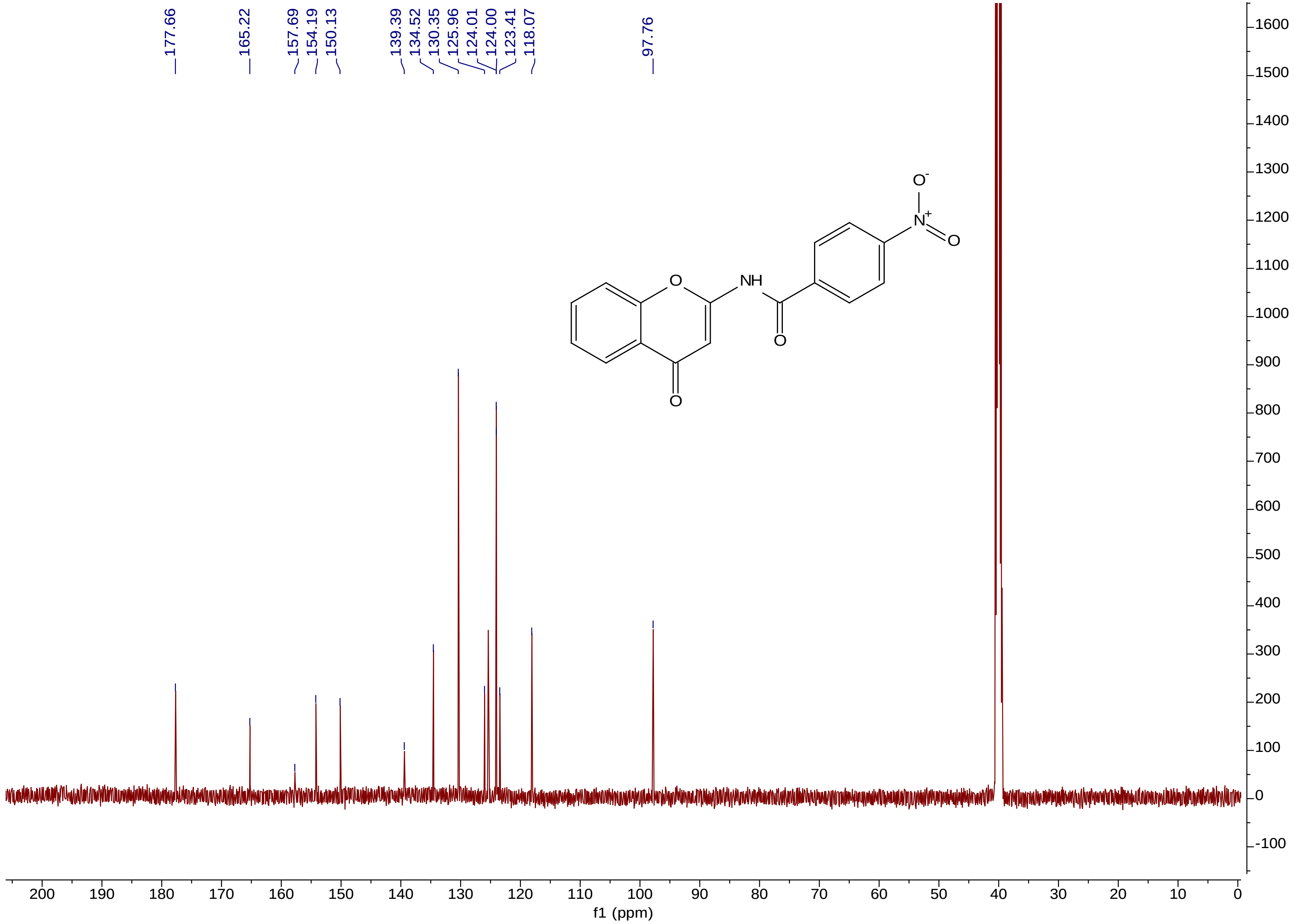

177.66
165.22
157.69
154.19
150.13
139.39
134.52
130.35
125.96
124.01
124.00
123.41
118.07
97.76
O
NH
O
O
N+
O-
O
1600
1500
1400
1300
1200
1100
1000
900
800
700
600
500
400
300
200
100
0
-100
200 190 180 170 160 150 140 130 120 110 100 90 80 70 60 50 40 30 20 10 0
f1 (ppm)

2,4-Dinitro-*N*-(4-oxo-4*H*-chromen-2-yl)benzamide (6n)

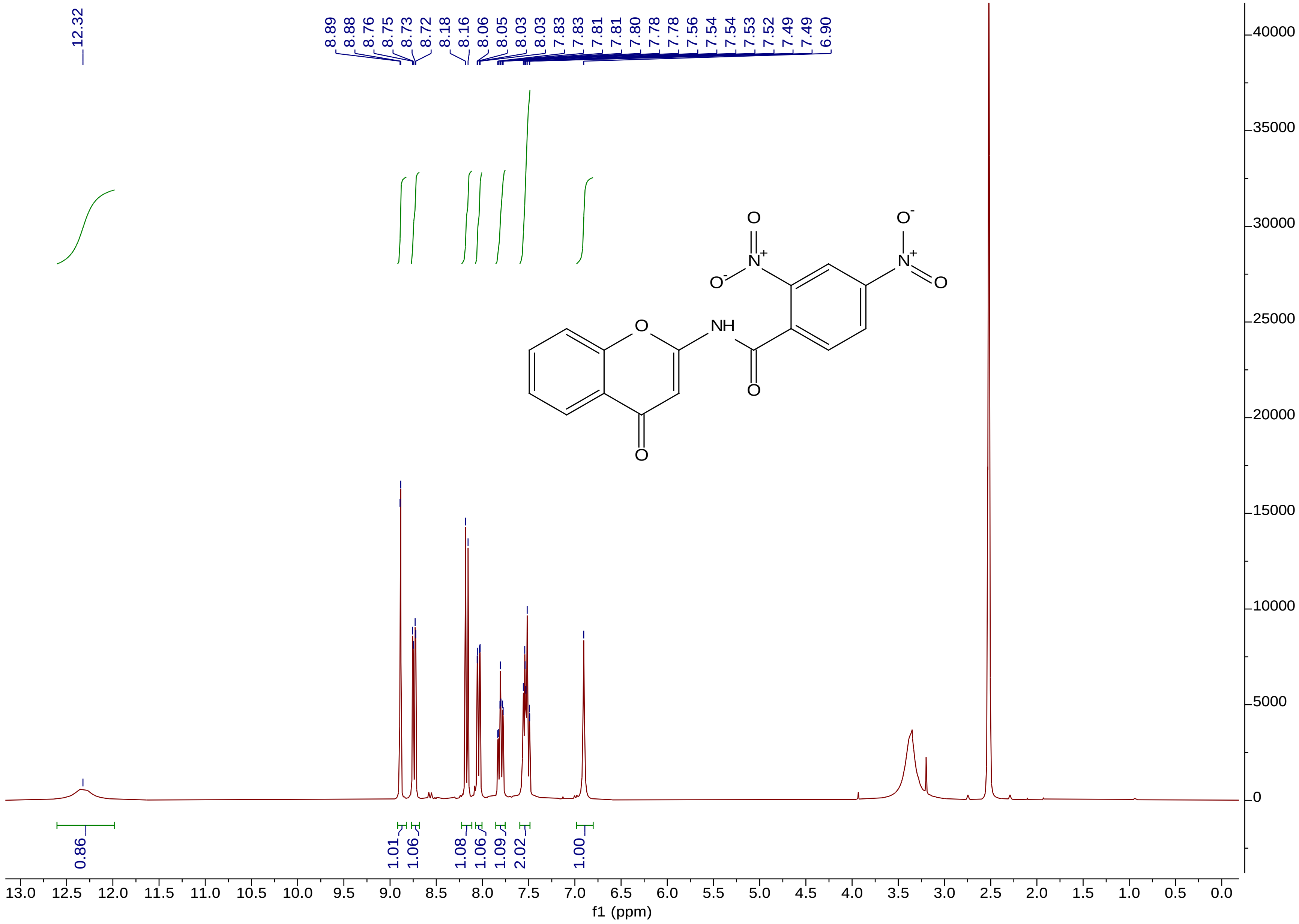

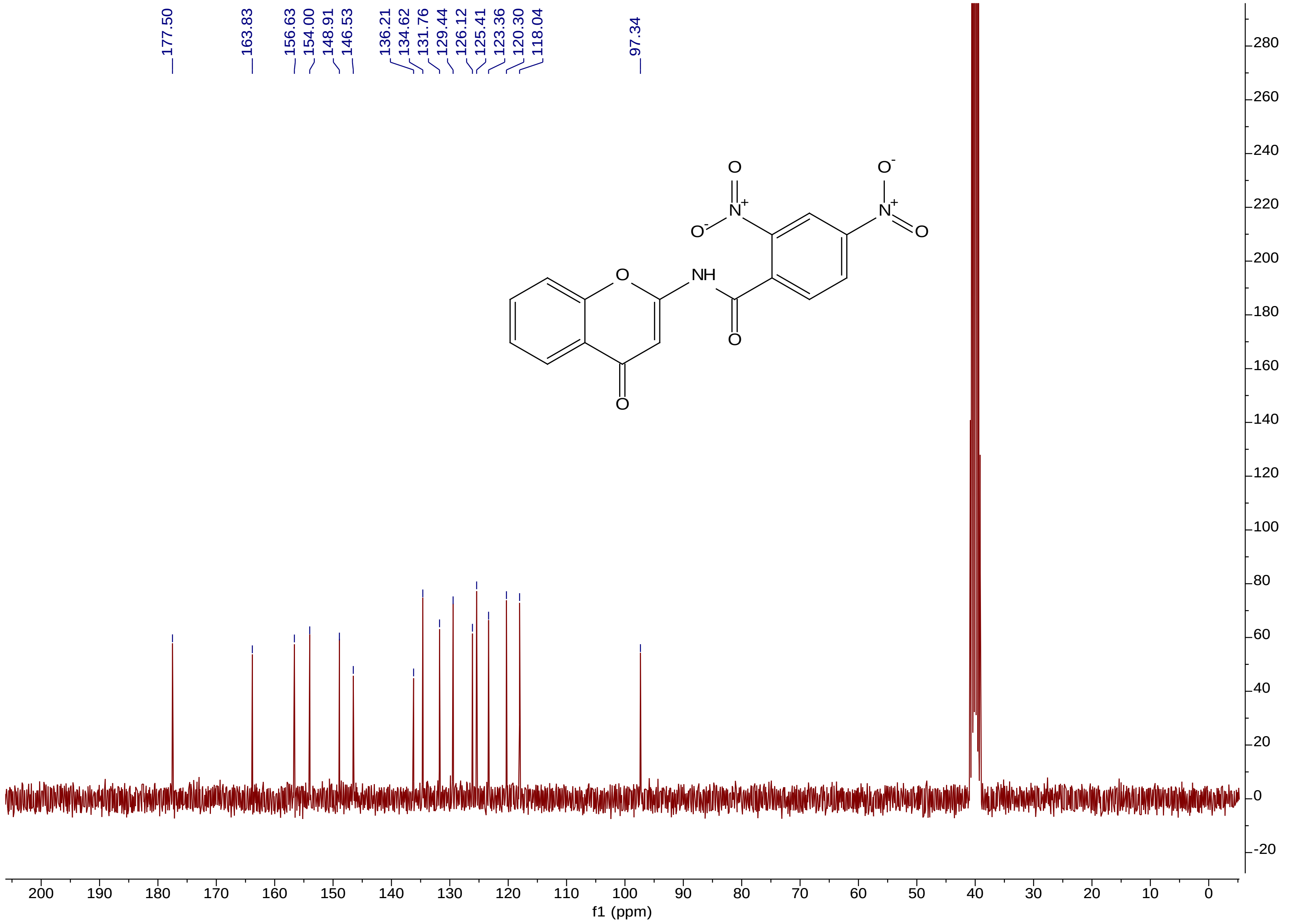
177.50
163.83
156.63
154.00
148.91
146.53
136.21
134.62
131.76
129.44
126.12
125.41
123.36
120.30
118.04
97.34
f1 (ppm)

2,4-Dinitro-N-(4-oxo-4H-chromen-2-yl)benzamide (6n)

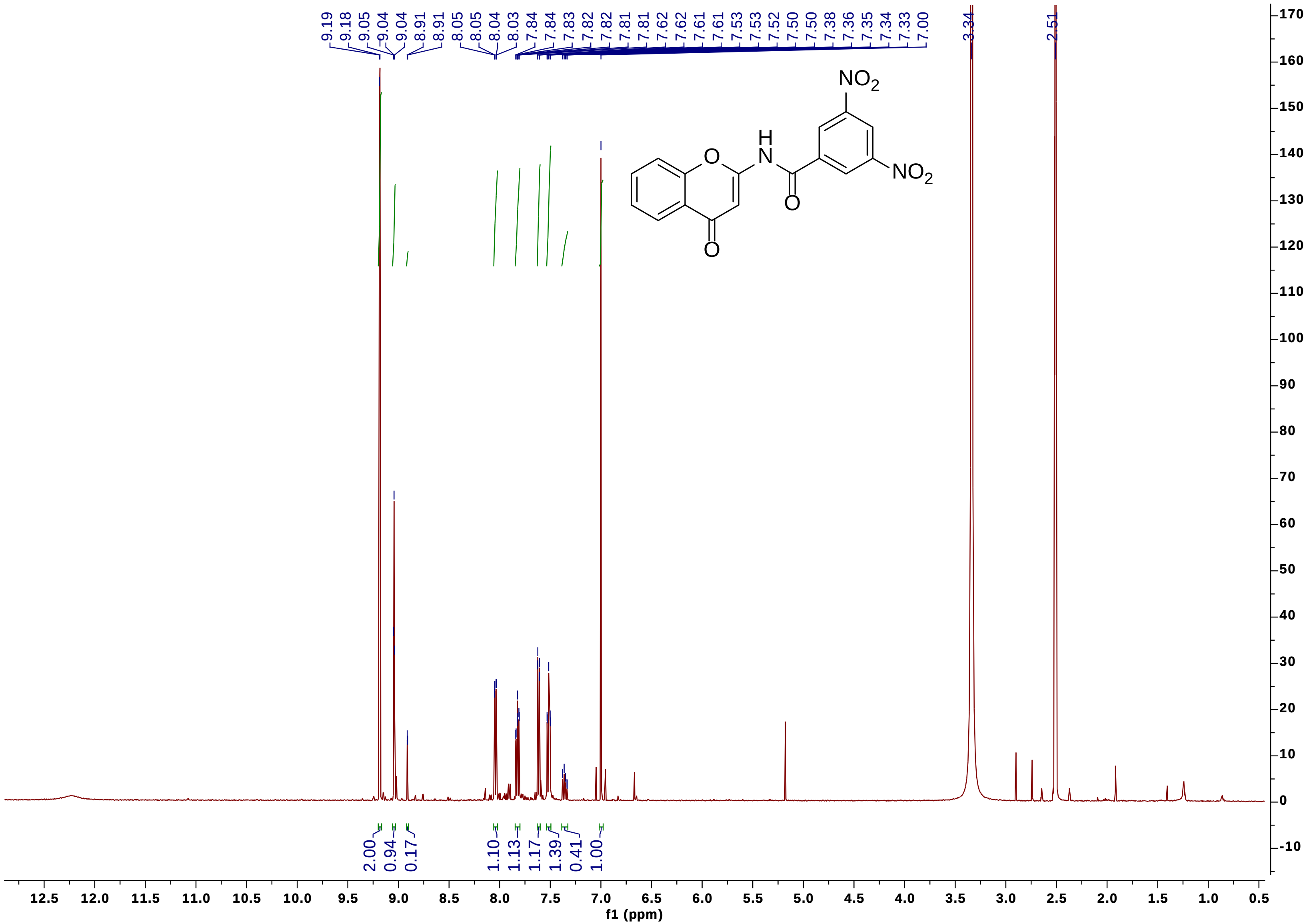

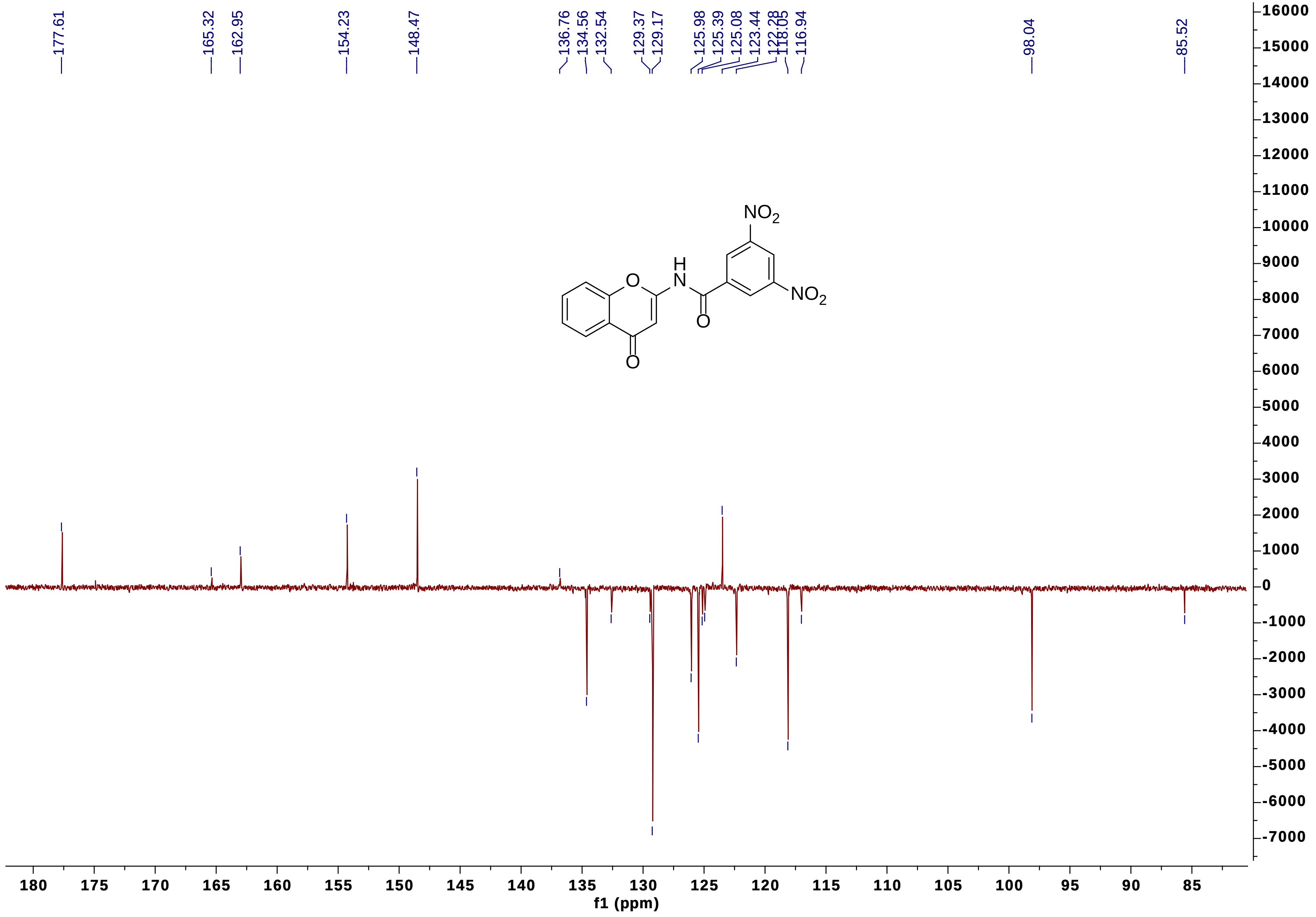
177.61
165.32
162.95
154.23
148.47
136.76
134.56
132.54
129.37
129.17
125.98
125.39
125.08
123.44
122.28
118.05
116.94
98.04
85.52
NO2
H
N
O
O
O
NO2
f1 (ppm)

4-Methoxy-*N*-(4-oxo-4*H*-chromen-2-yl)benzamide (6p)

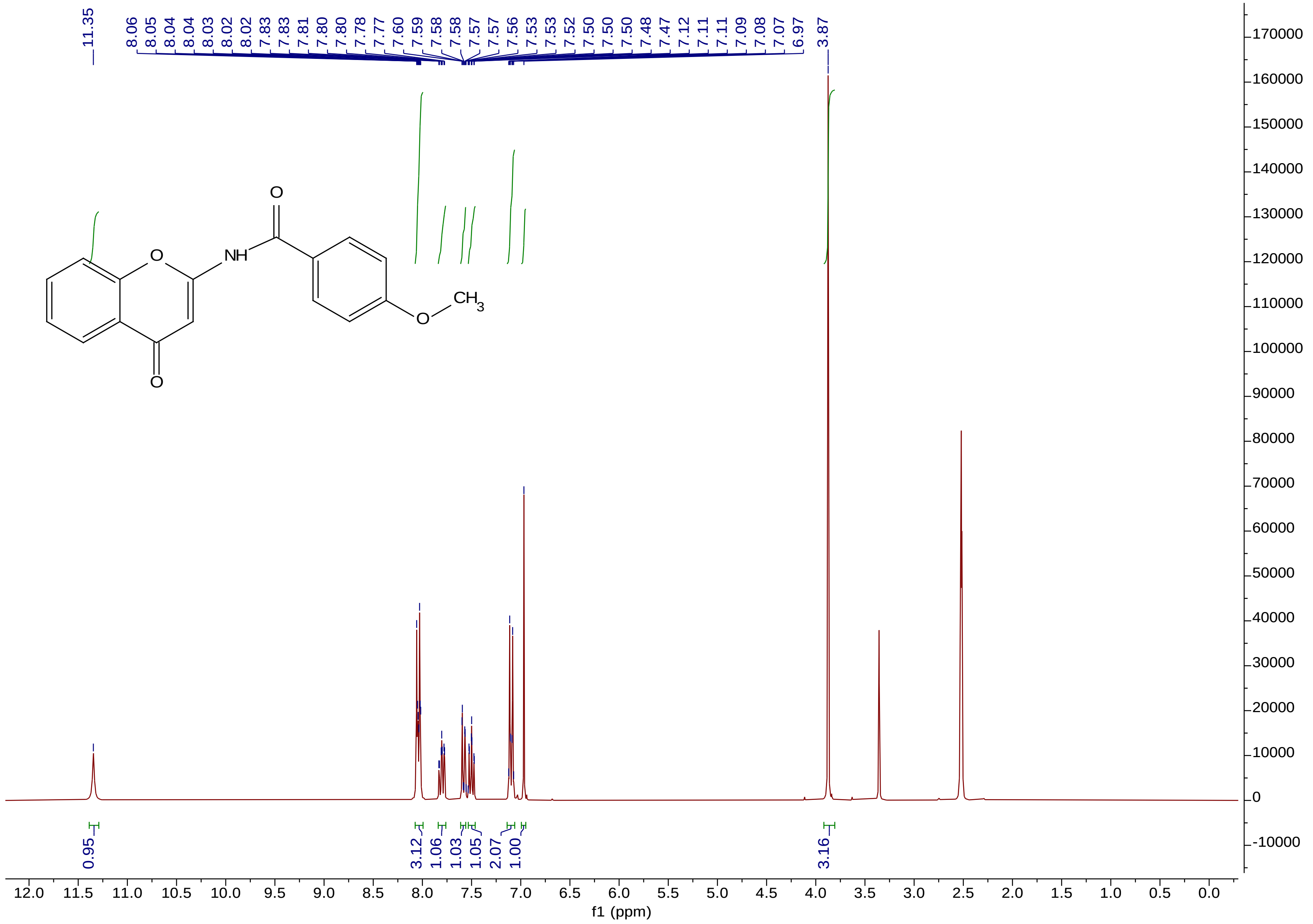

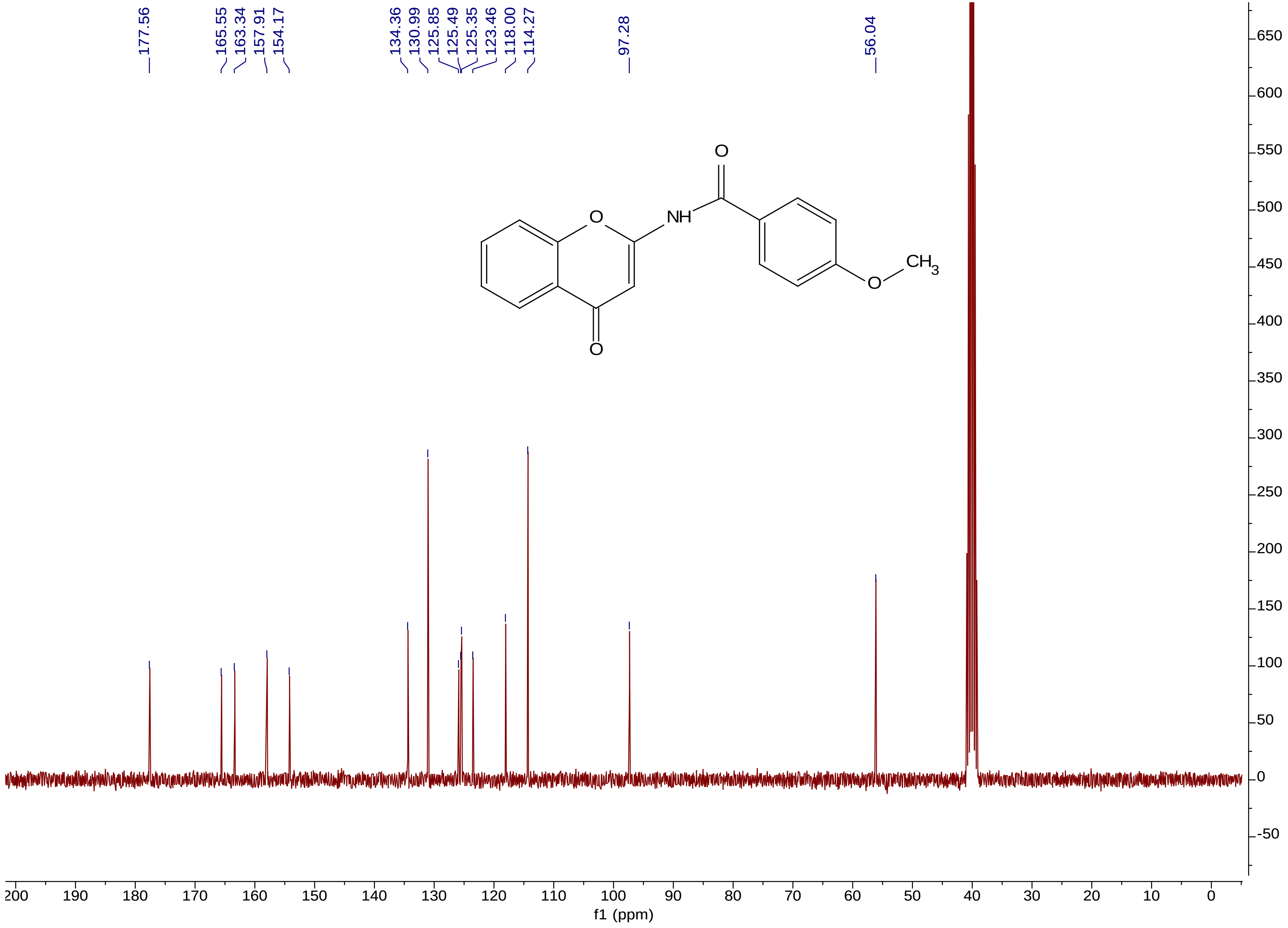
177.56
165.55
163.34
157.91
154.17
134.36
130.99
125.85
125.49
125.35
123.46
118.00
114.27
97.28
56.04
O
NH
O
CH3
O
f1 (ppm)

2-Methoxy-*N*-(4-oxo-4*H*-chromen-2-yl)benzamide (6q)

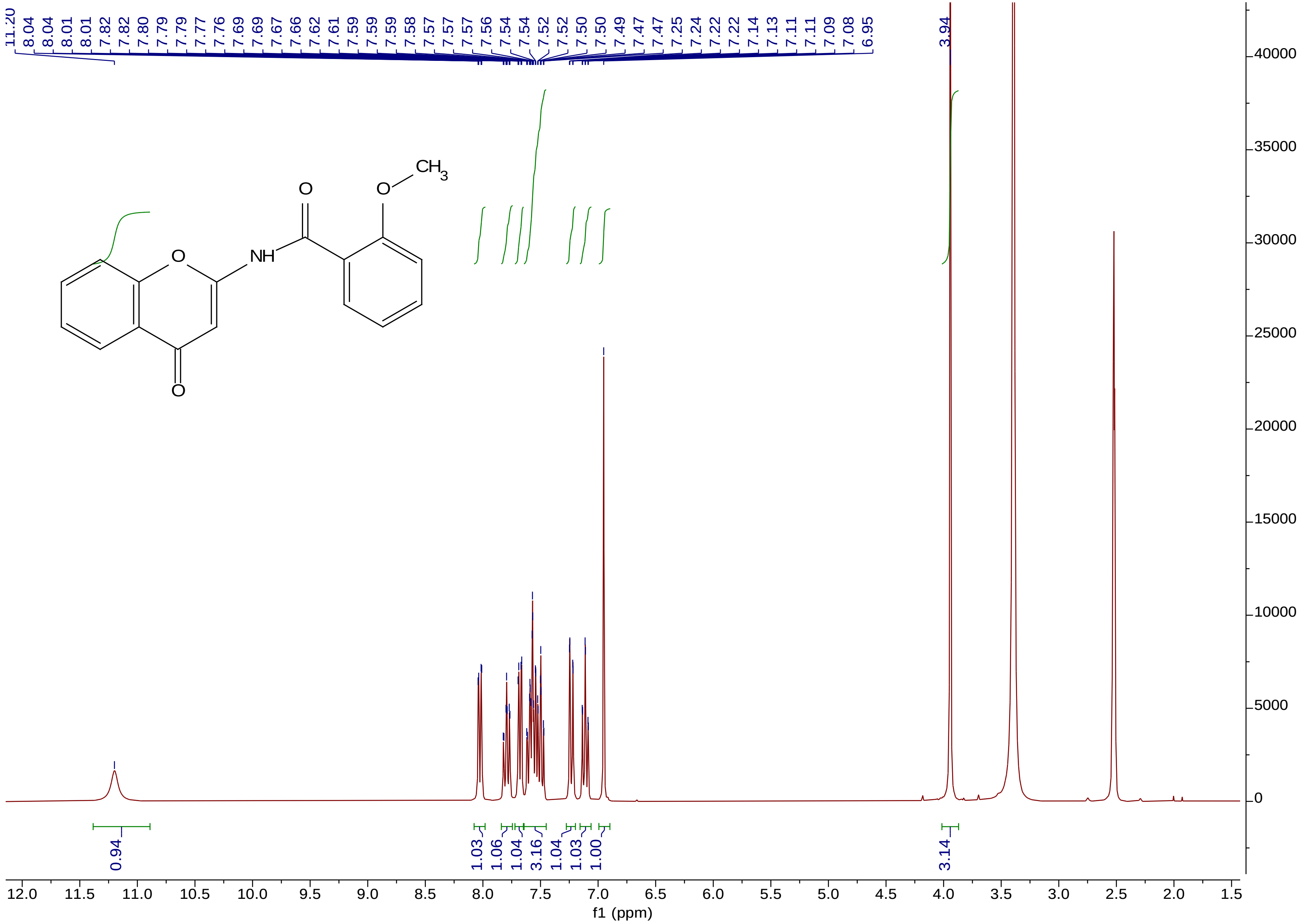

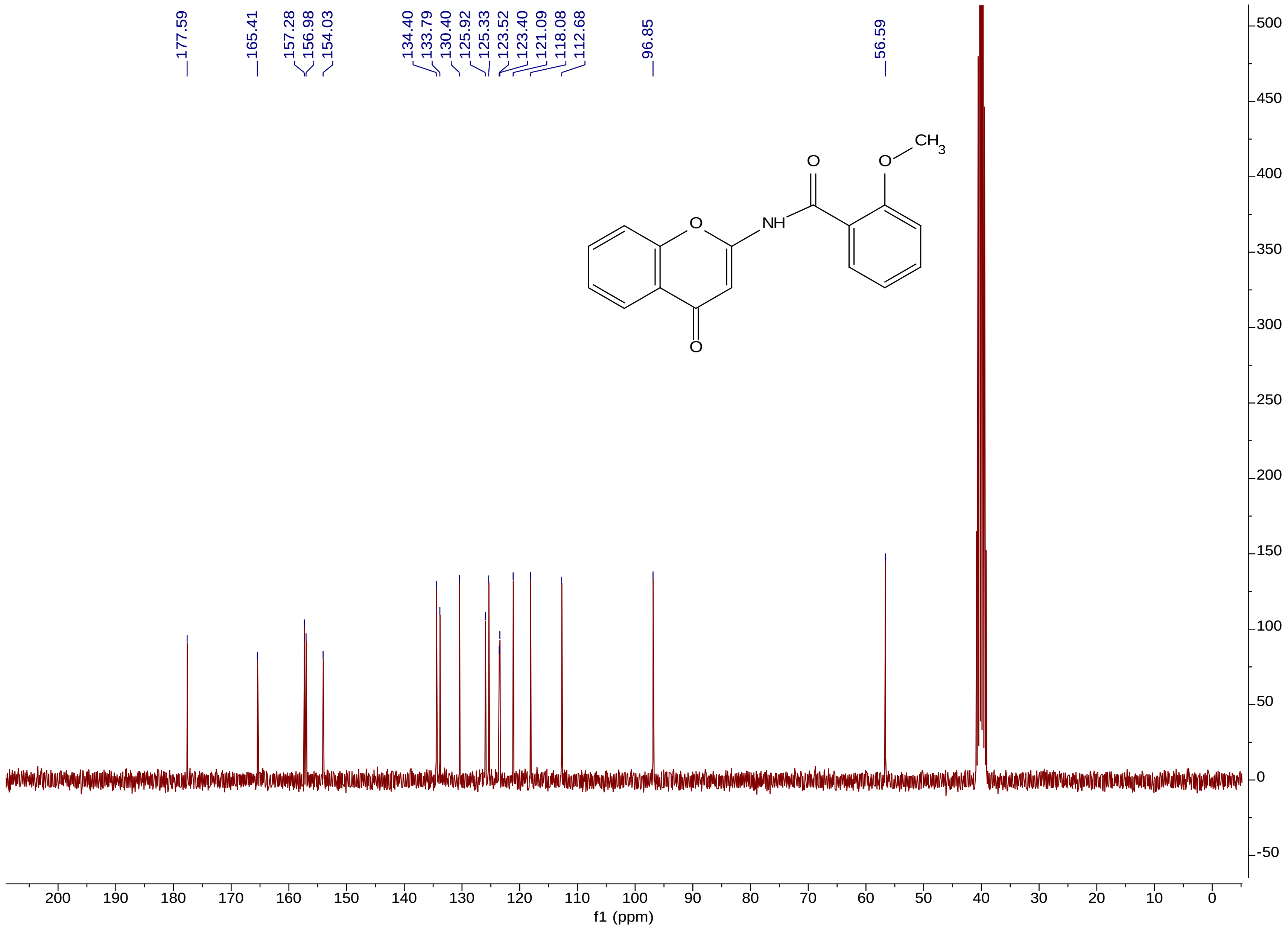
177.59
165.41
157.28
156.98
154.03
134.40
133.79
130.40
125.92
125.33
123.52
123.40
121.09
118.08
112.68
96.85
56.59
O
NH
O
O
CH3
O
f1 (ppm)

2-(Methylthio)-*N*-(4-oxo-4*H*-chromen-2-yl)benzamide (6r)

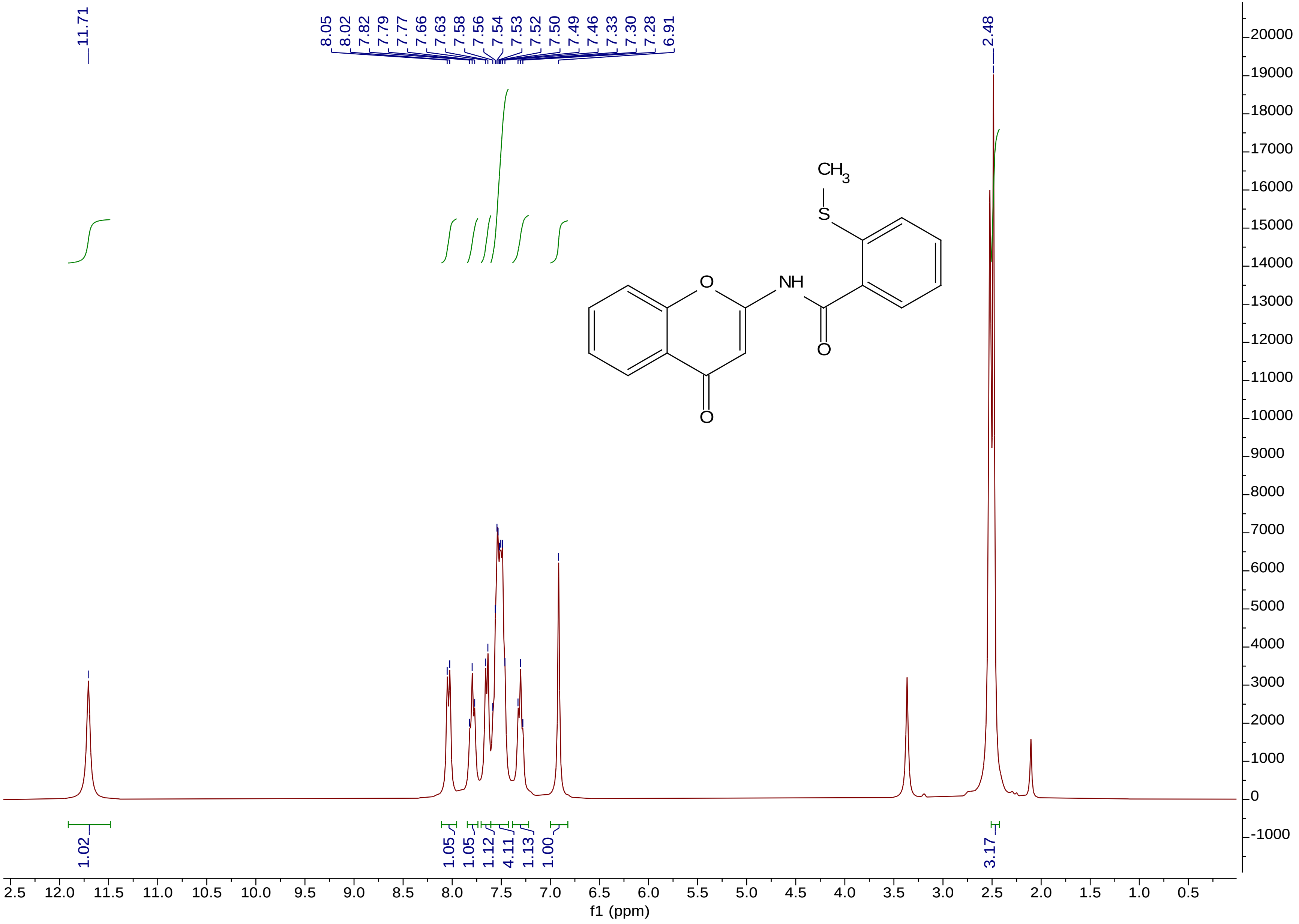

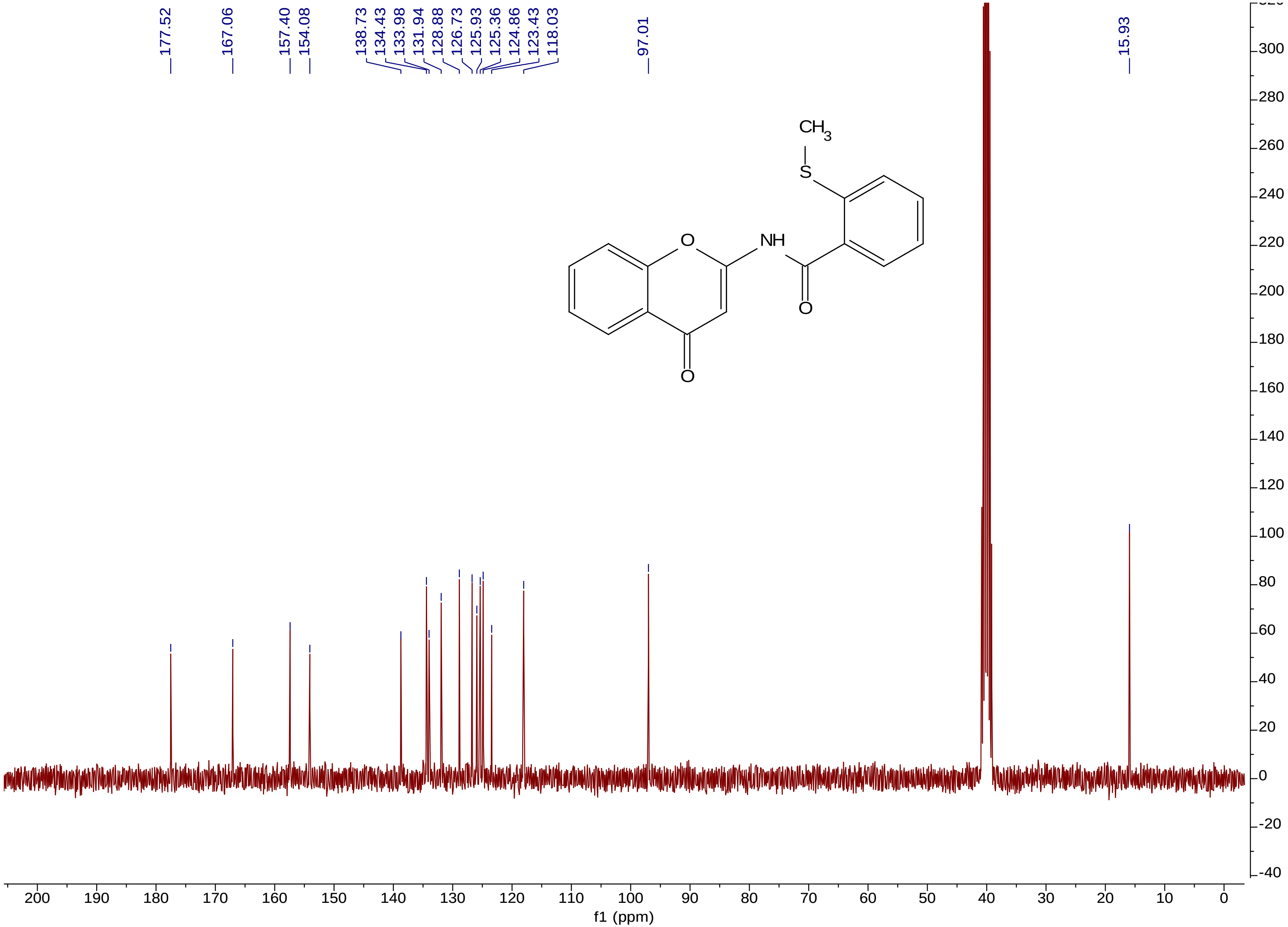
177.52
167.06
157.40
154.08
138.73
134.43
133.98
131.94
128.88
126.73
125.93
125.36
124.86
123.43
118.03
97.01
15.93
CH3
S
O
NH
O
O
f1 (ppm)

3,4-Dimethoxy-*N*-(4-oxo-4*H*-chromen-2-yl)benzamide (6s)

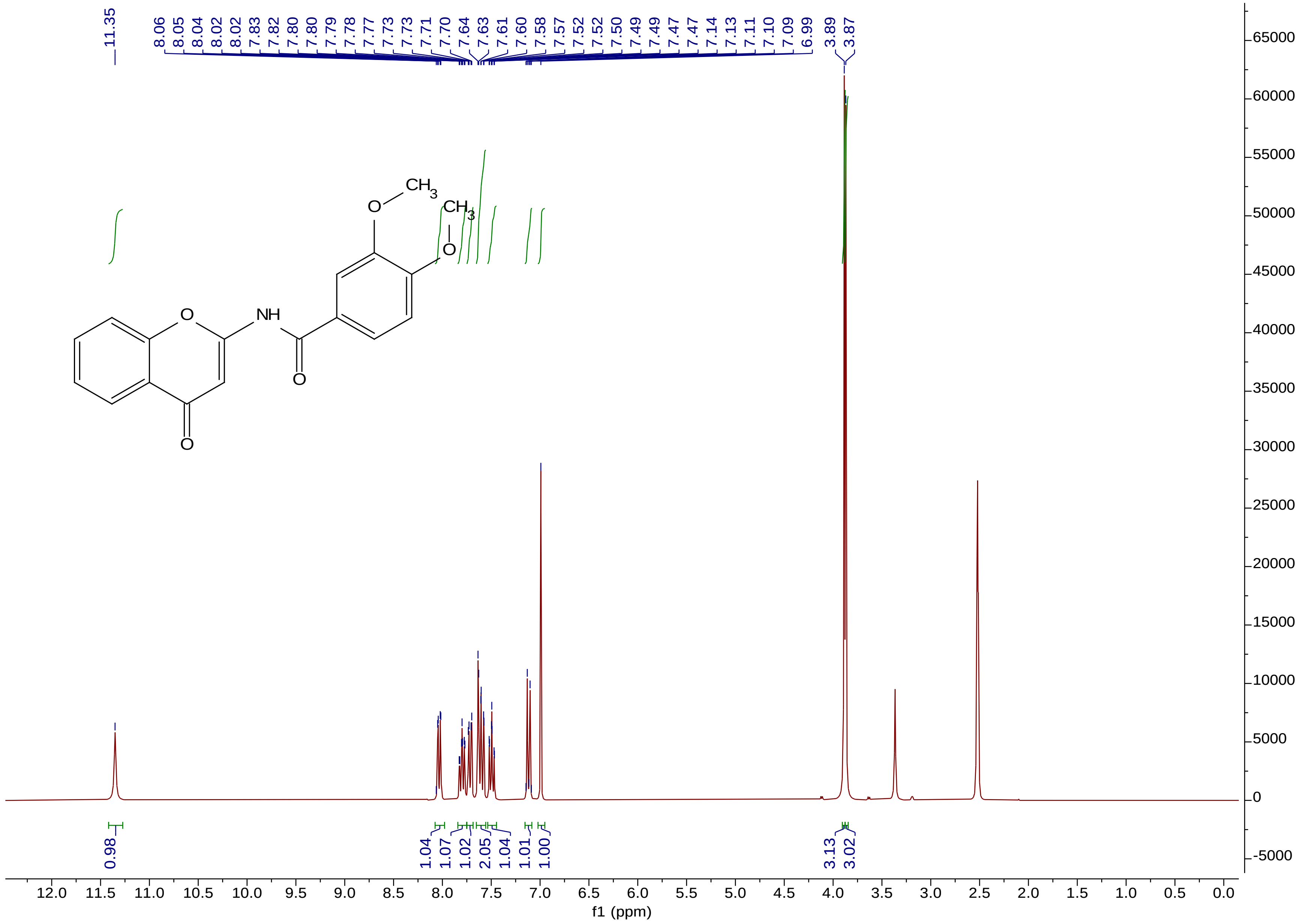

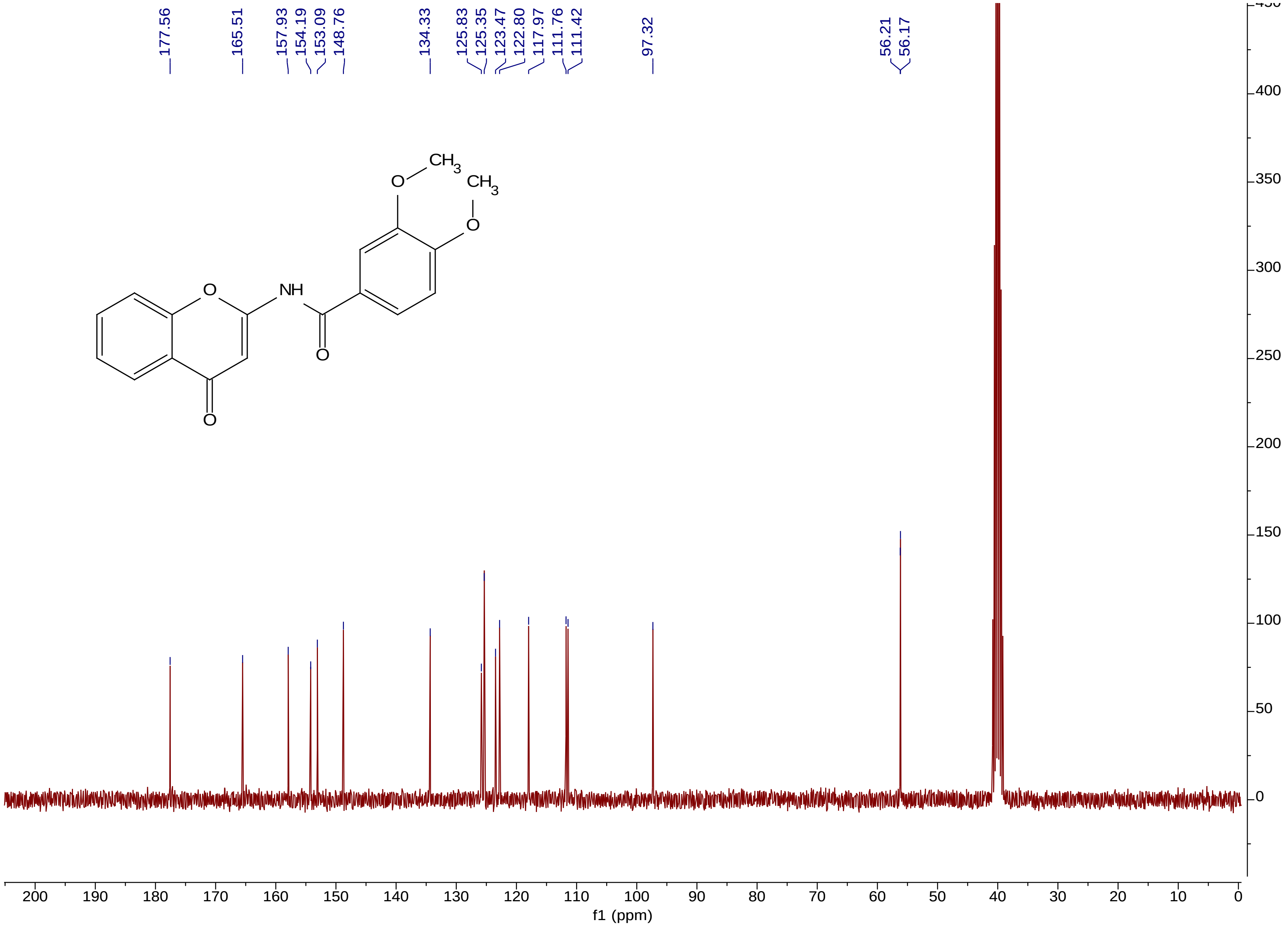
177.56
165.51
157.93
154.19
153.09
148.76
134.33
125.83
125.35
123.47
122.80
117.97
111.76
111.42
97.32
56.21
56.17
O
NH
O
O
O
CH3
O
CH3
200
190
180
170
160
150
140
130
120
110
100
90
80
70
60
50
40
30
20
10
0
f1 (ppm)
400
350
300
250
200
150
100
50
0

3,5-Dimethoxy-*N*-(4-oxo-4*H*-chromen-2-yl)benzamide (6t)

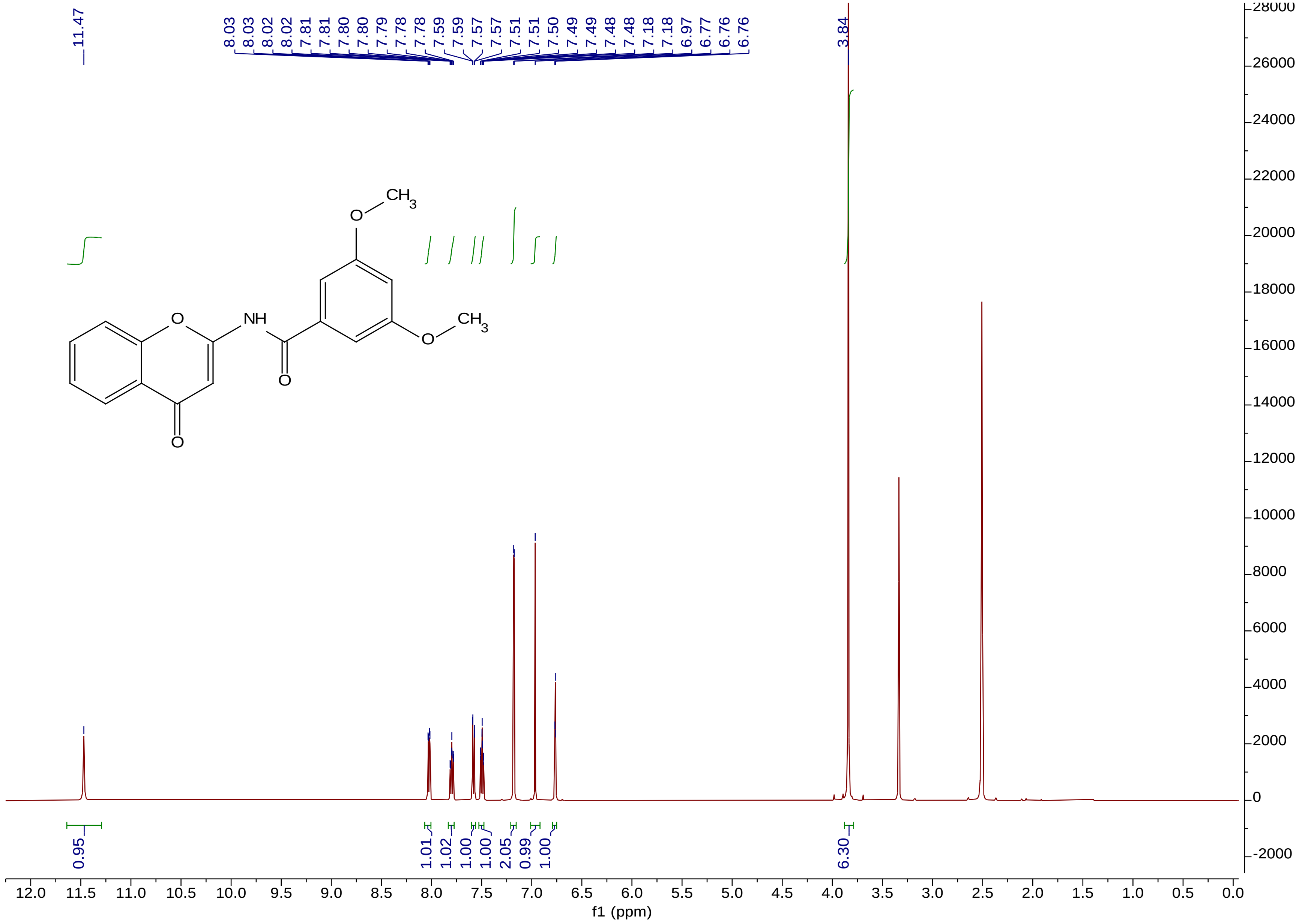

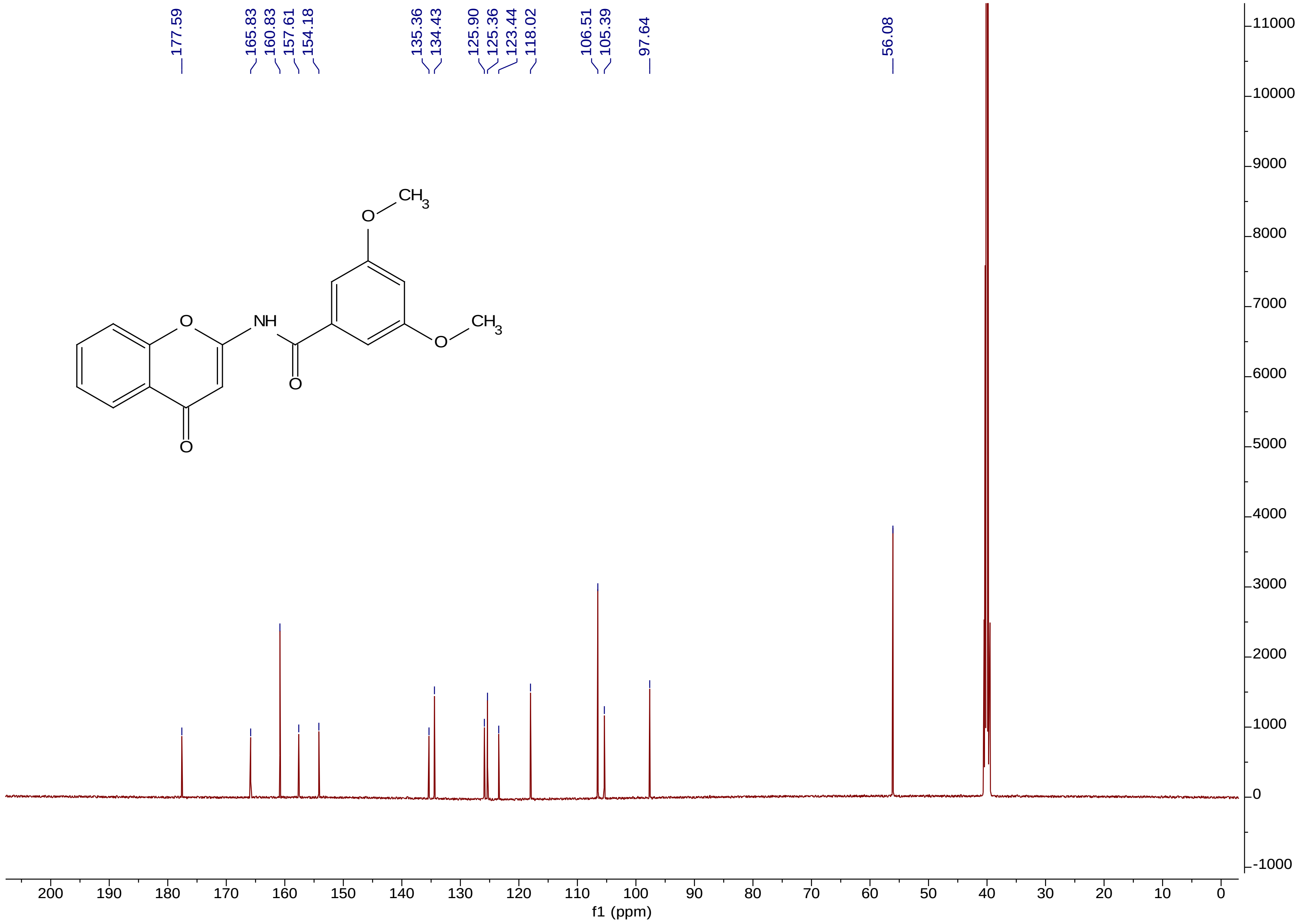
177.59
165.83
160.83
157.61
154.18
135.36
134.43
125.90
125.36
123.44
118.02
106.51
105.39
97.64
56.08
CH3
O
NH
O
CH3
O
O
O
11000
10000
9000
8000
7000
6000
5000
4000
3000
2000
1000
0
-1000
200 190 180 170 160 150 140 130 120 110 100 90 80 70 60 50 40 30 20 10 0
f1 (ppm)

4-Morpholino-*N*-(4-oxo-4*H*-chromen-2-yl)benzamide (6u)

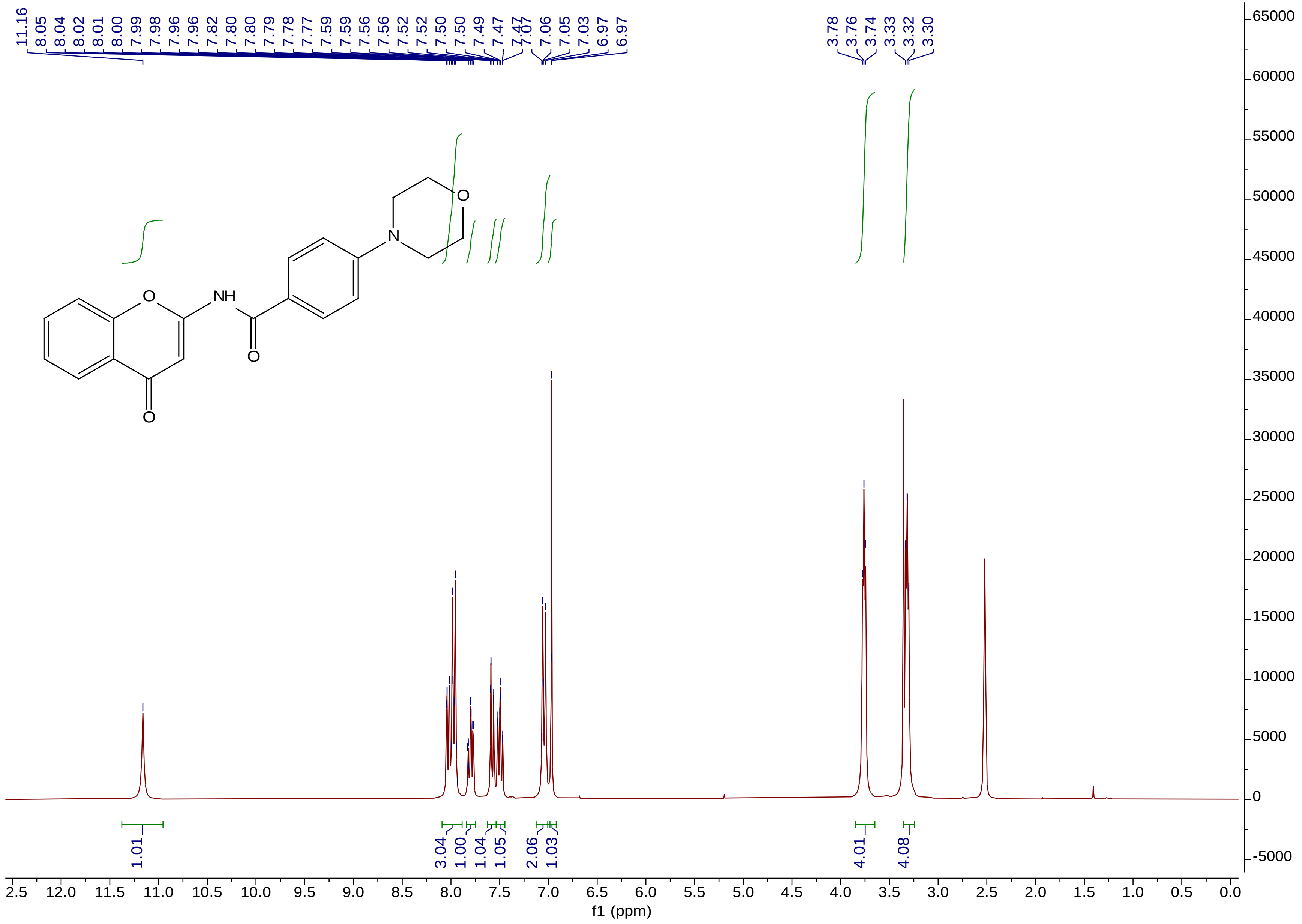

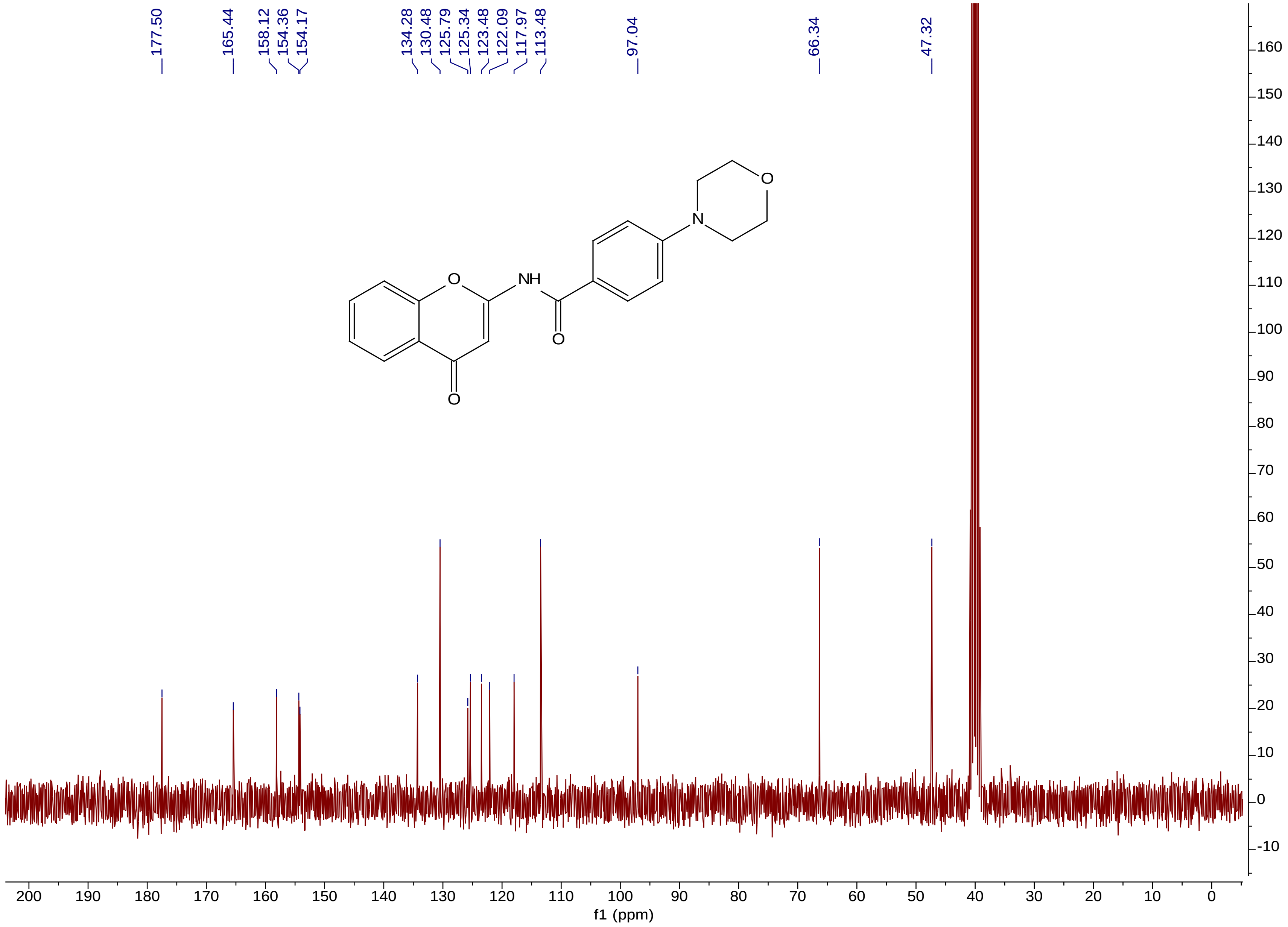
177.50
165.44
158.12
154.36
154.17
134.28
130.48
125.79
125.34
123.48
122.09
117.97
113.48
97.04
66.34
47.32
O
NH
O
O
N
O
f1 (ppm)

N-(4-Oxo-4H-chromen-2-yl)pyrimidine-2-carboxamide (6v)

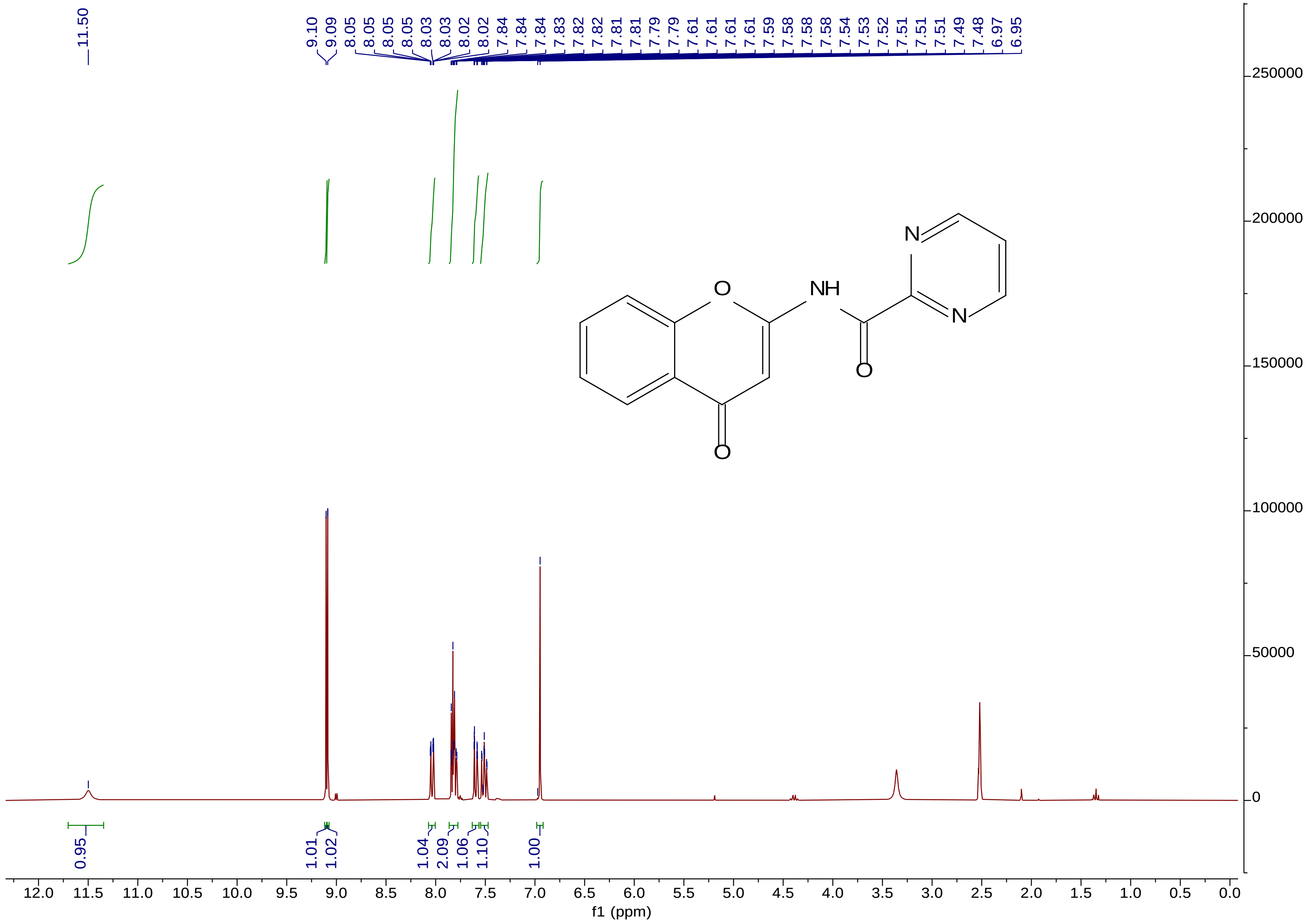

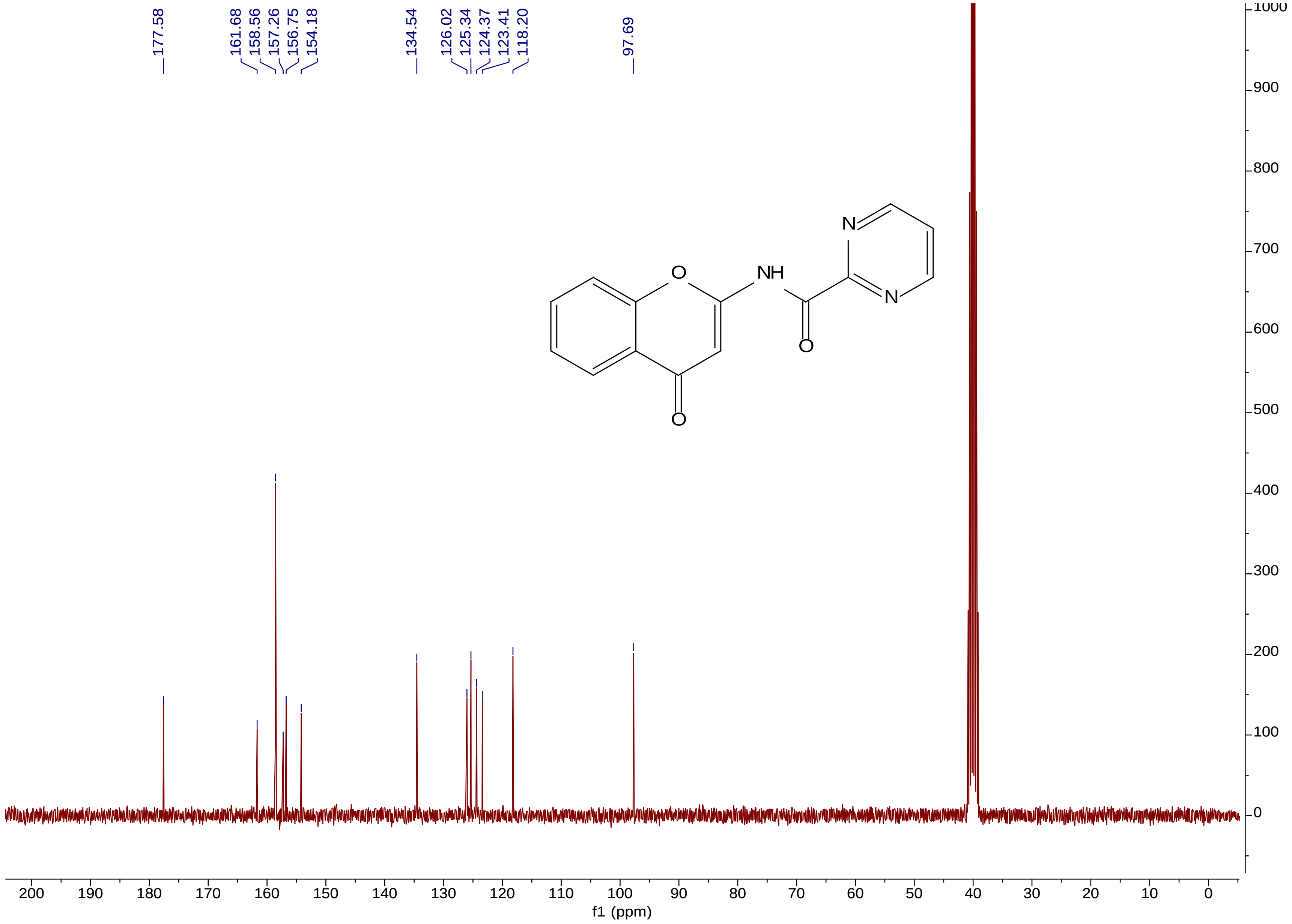

177.58
161.68
158.56
157.26
156.75
154.18
134.54
126.02
125.34
124.37
123.41
118.20
97.69
O
NH
N
N
O
O
1000
900
800
700
600
500
400
300
200
100
0
200
190
180
170
160
150
140
130
120
110
100
90
80
70
60
50
40
30
20
10
0
f1 (ppm)

N-(4-Oxo-4*H*-chromen-2-yl)butyramide (6w)

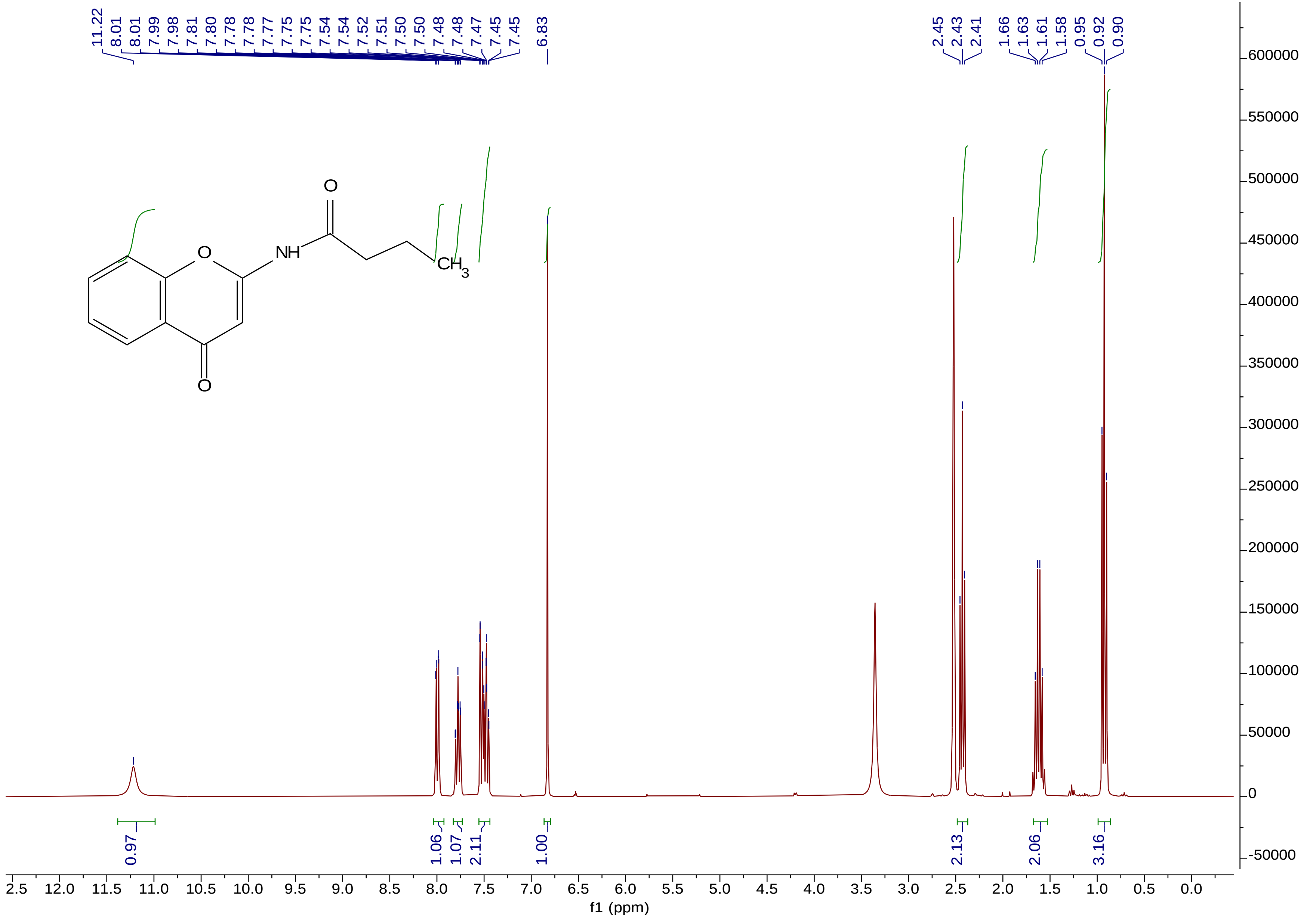

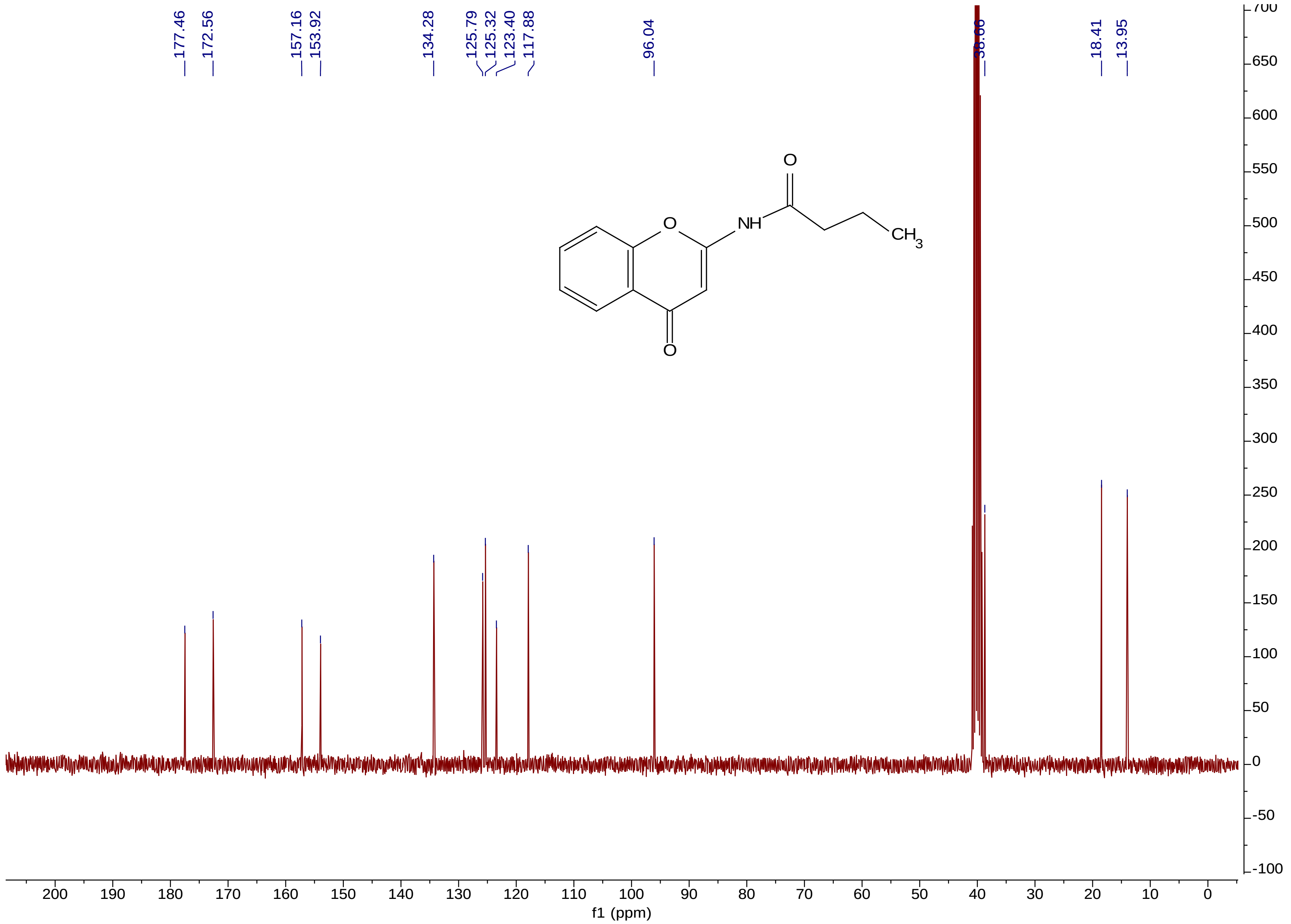
177.46
172.56
157.16
153.92
134.28
125.79
125.32
123.40
117.88
96.04
38.66
18.41
13.95
O
NH
CH3
O
O
f1 (ppm)

_N_-(4-Oxo-4_H_-chromen-2-yl)cyclohexanecarboxamide (6x)

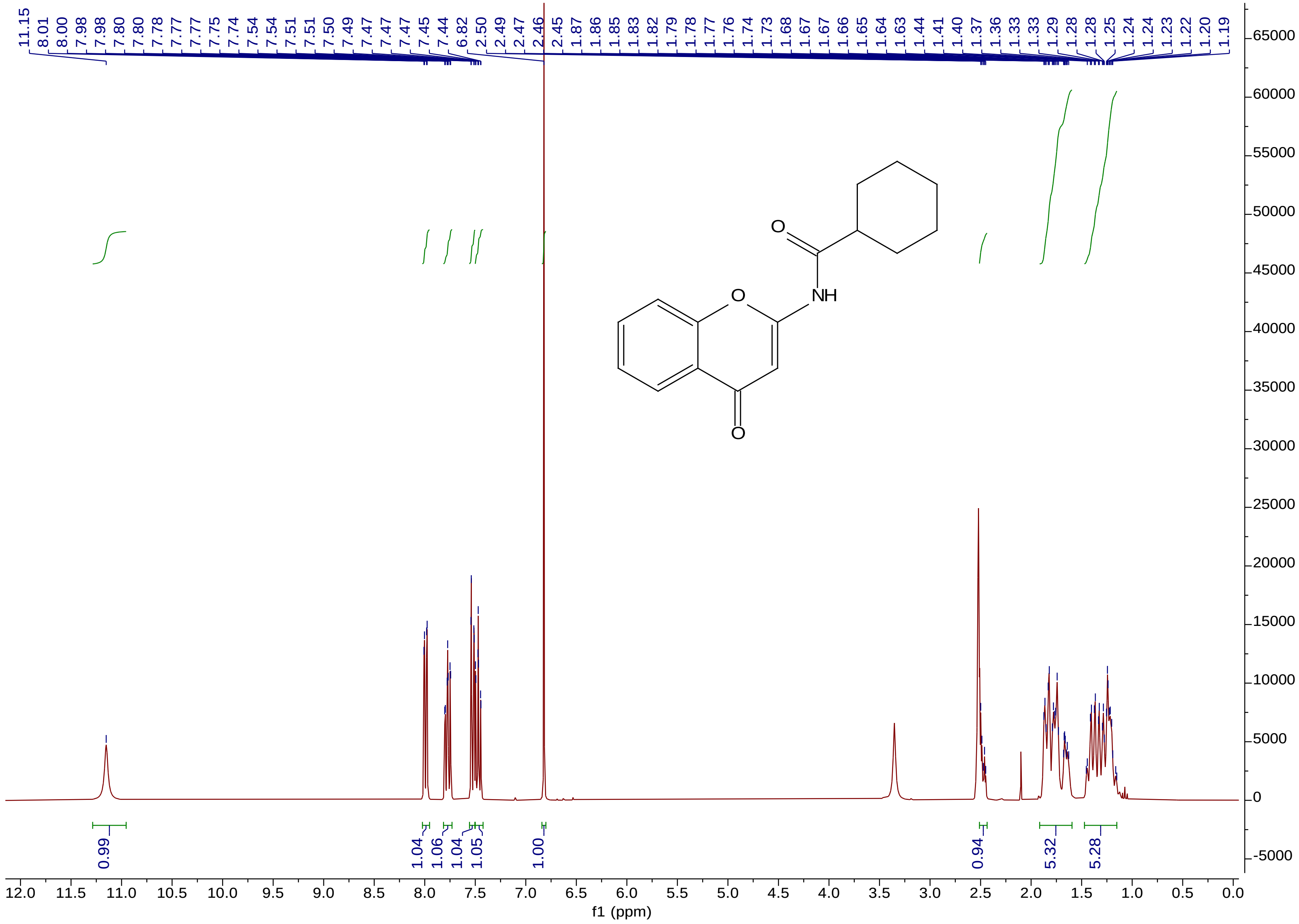

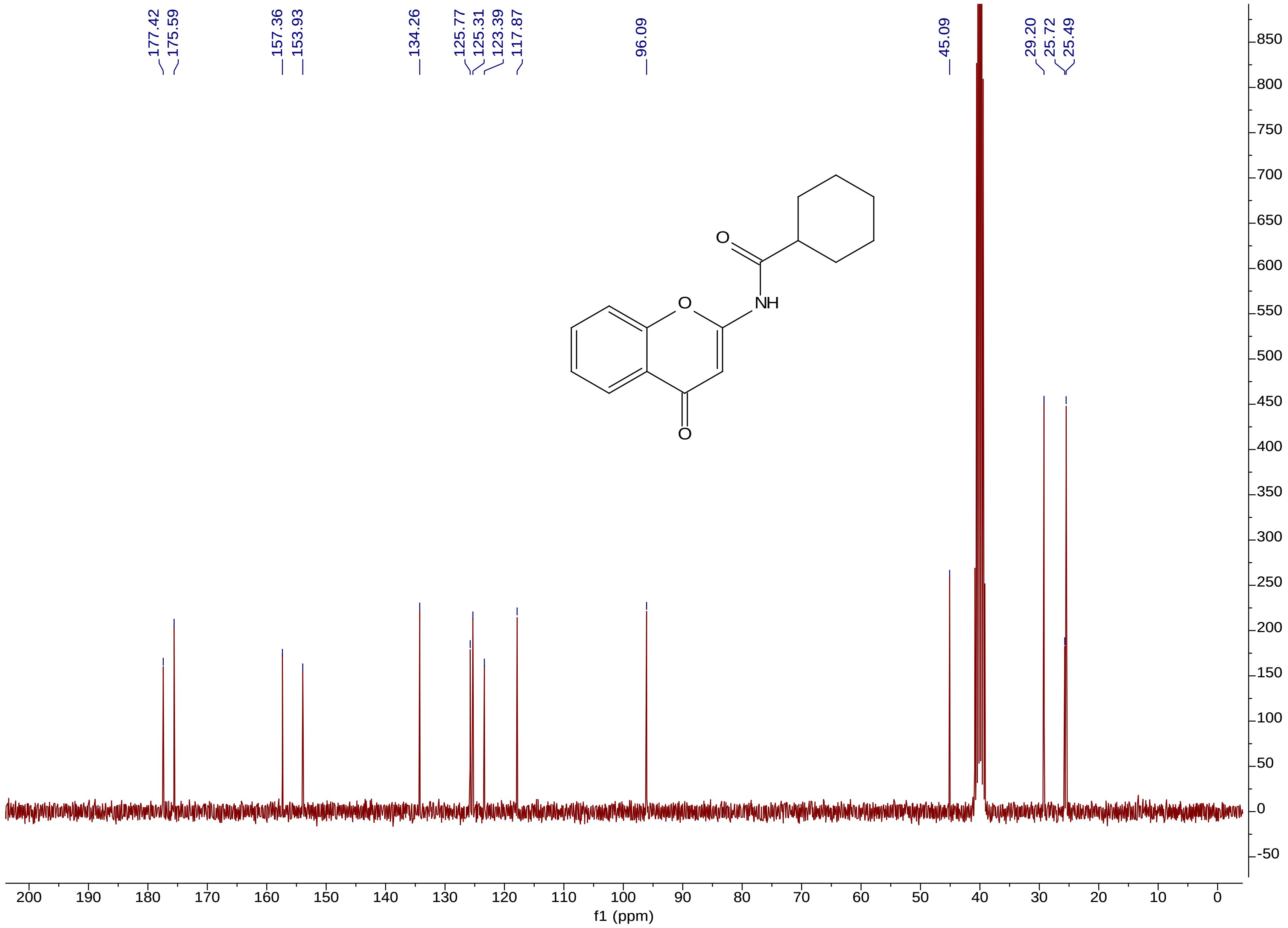

177.42
175.59
157.36
153.93
134.26
125.77
125.31
123.39
117.87
96.09
45.09
29.20
25.72
25.49
O
NH
O
O
f1 (ppm)

(3R,5R,7R)-*N*-(4-Oxo-4*H*-chromen-2-yl)adamantane-1-carboxamide (6y)

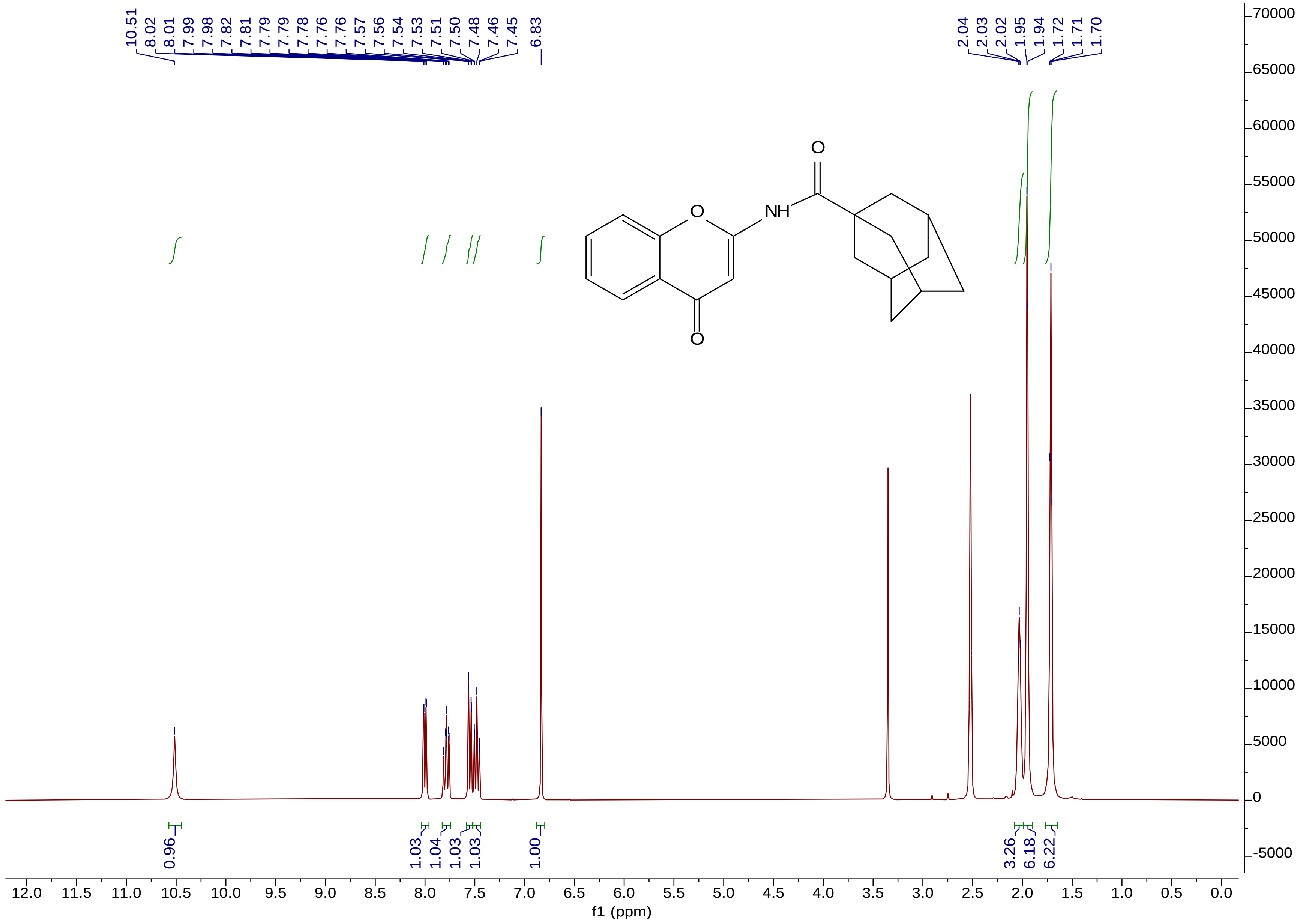

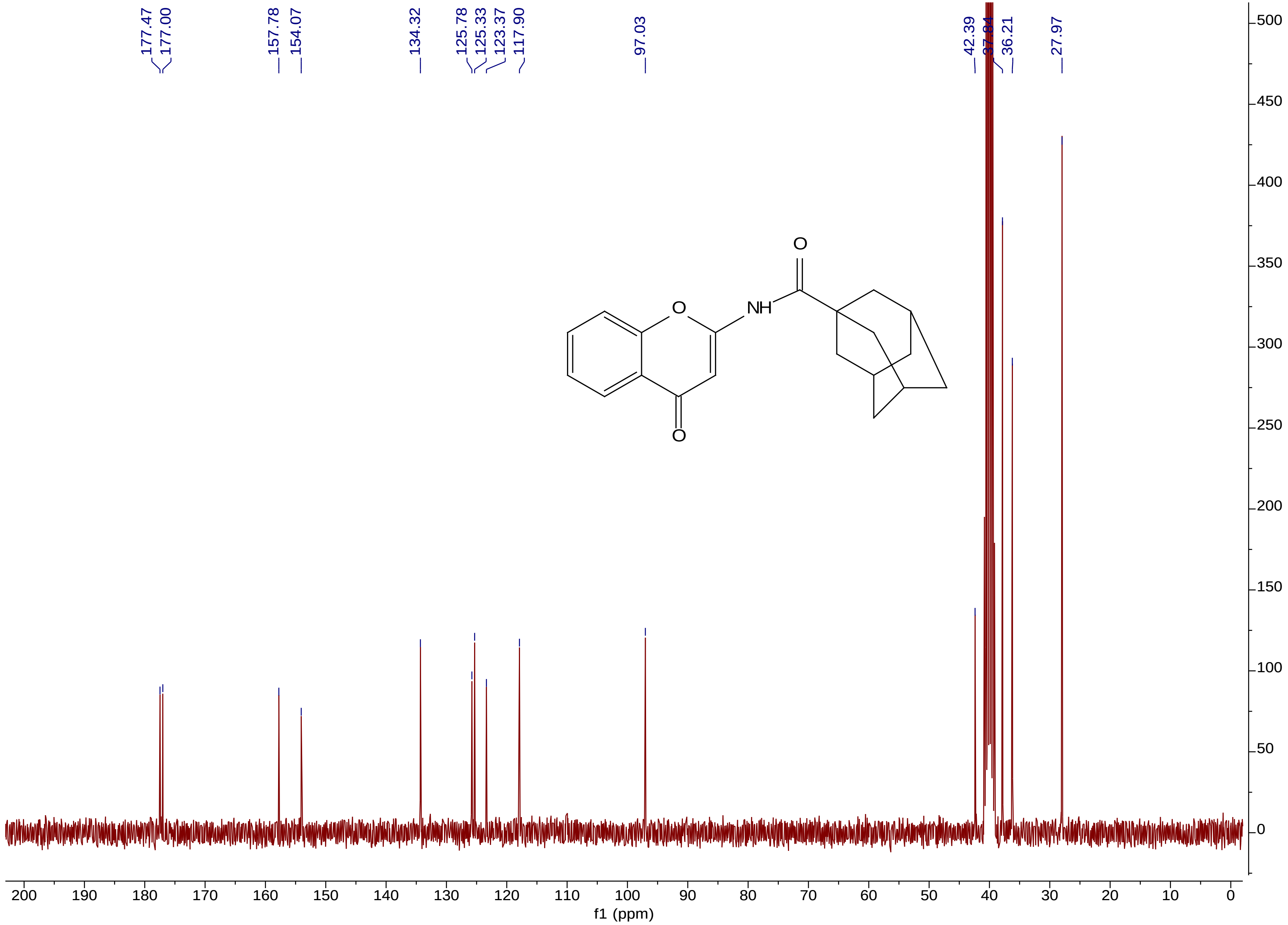
177.47
177.00
157.78
154.07
134.32
125.78
125.33
123.37
117.90
97.03
42.39
36.21
27.97
O
NH
O
O
500
450
400
350
300
250
200
150
100
50
0
200 190 180 170 160 150 140 130 120 110 100 90 80 70 60 50 40 30 20 10 0
f1 (ppm)

4-Oxo-*N*-(4-oxo-4*H*-chromen-2-yl)-4*H*-chromene-2-carboxamide (6z)

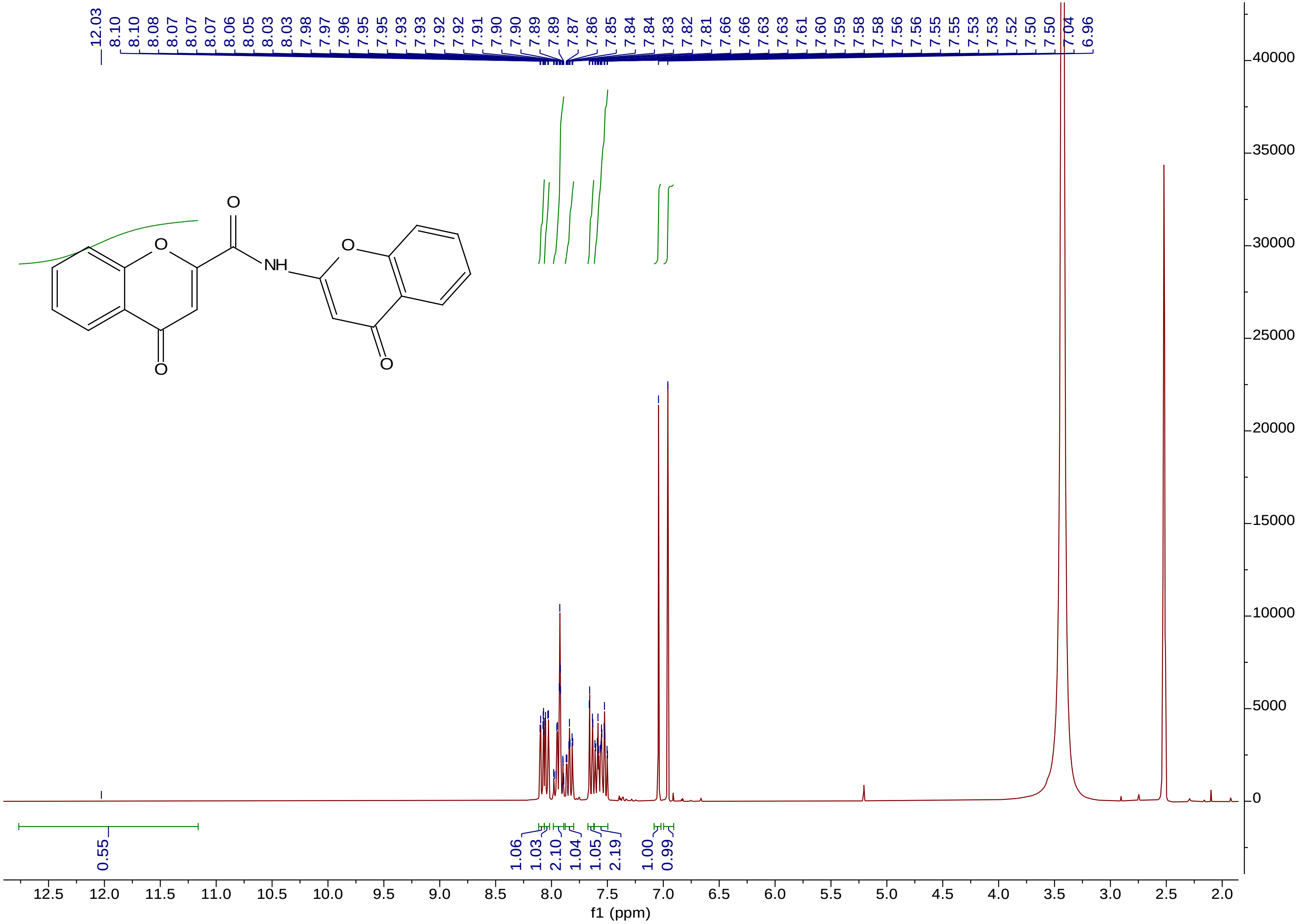

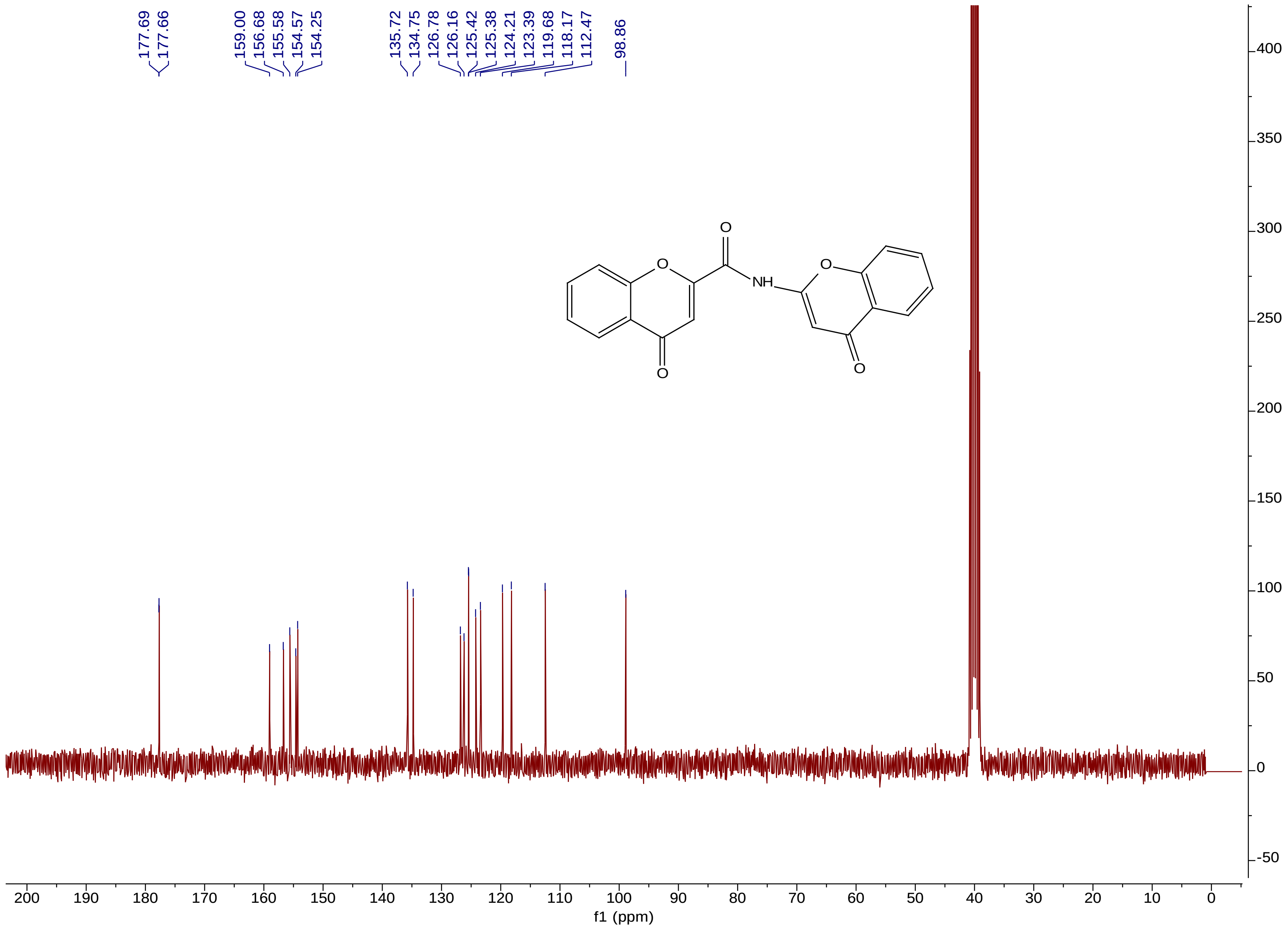
177.69
177.66
159.00
156.68
155.58
154.57
154.25
135.72
134.75
126.78
126.16
125.42
125.38
124.21
123.39
119.68
118.17
112.47
98.86
O
O
NH
O
O
O
O
f1 (ppm)

3-(4-Bromophenyl)-2-isocyanato-4*H*-chromen-4-one (7)

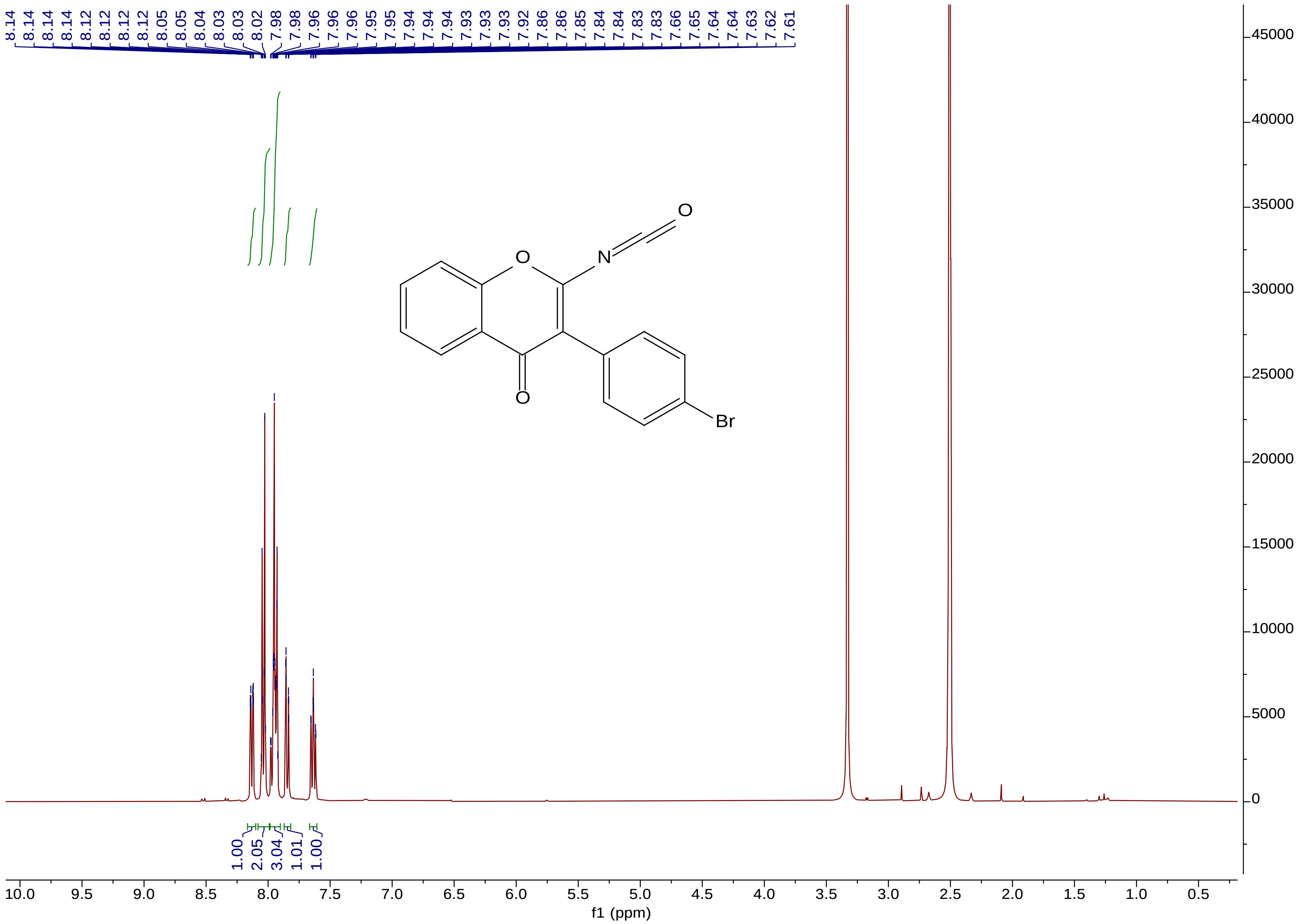

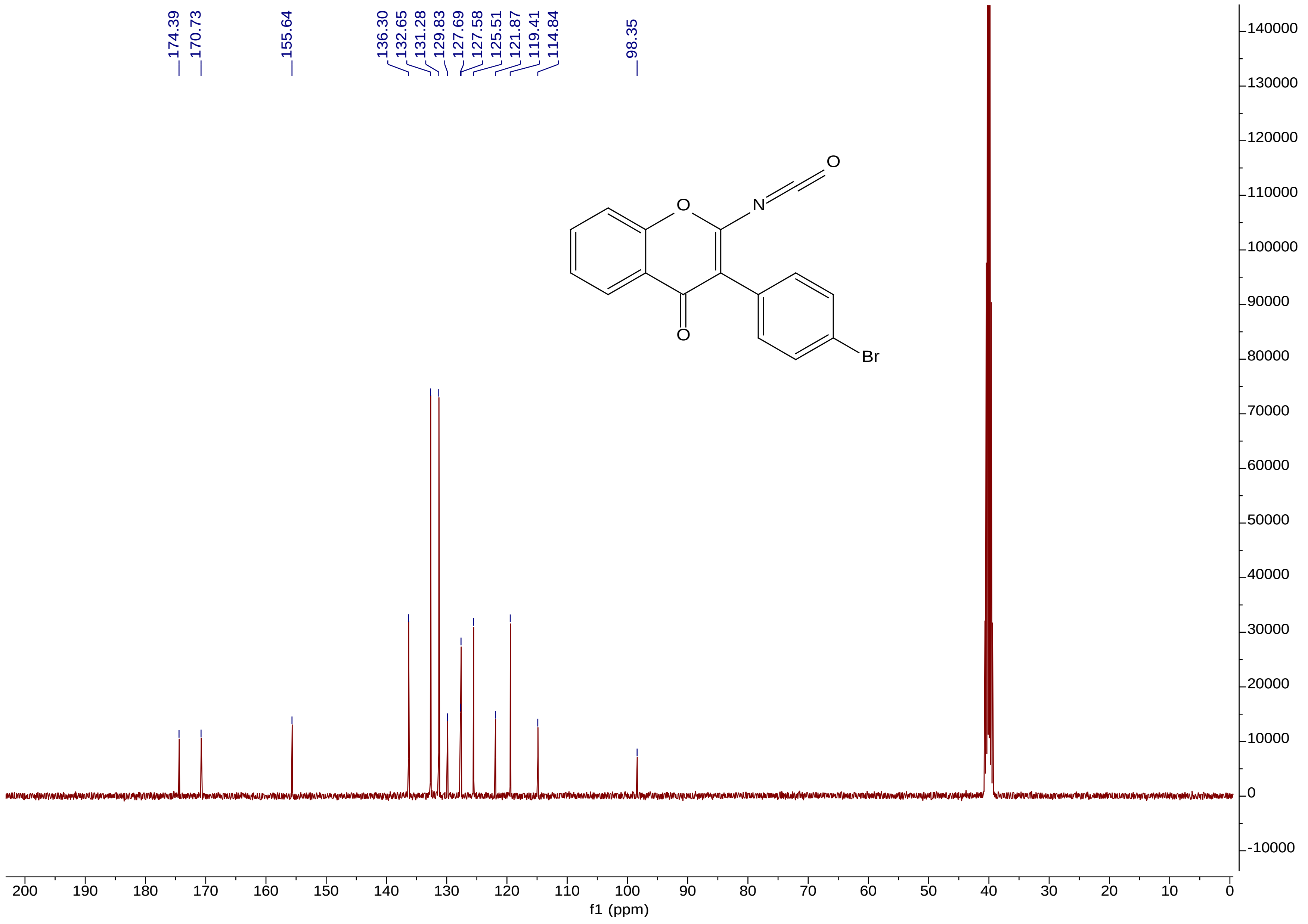
174.39
170.73
155.64
136.30
132.65
131.28
129.83
127.69
127.58
125.51
121.87
119.41
114.84
98.35
O
N
O
O
Br
f1 (ppm)
